# Supplementary material for: A comprehensive synthetic library of poly-N-acetyl glucosamines enabled vaccine against lethal challenges of Staphylococcus aureus
Source: Nat Commun. 2024 Apr 24;15:3420. doi: 10.1038/s41467-024-47457-4 (PMC11043332; doi:10.1038/s41467-024-47457-4)
Supplement: Supplementary file 1 — Supplementary Information [file 41467_2024_47457_MOESM1_ESM.pdf]

Supplementary Information for

A comprehensive synthetic library of poly-*N*-acetyl glucosamines enabled vaccine against lethal challenges of *Staphylococcus aureus*

Authors

Zibin Tan[1,2,3]†, Weizhun Yang[4,1,2]†, Nicholas A. O'Brien[5], Xingling Pan[1,2], Sherif Ramadan[1,2,6], Terence Marsh[7], Neal Hammer[7], Colette Cywes-Bentley[8], Mariana Vinacur[8], Gerald B. Pier[8], Jeffrey C. Gildersleeve[5], Xuefei Huang[1,2,9]*

†Equal contribution

*Corresponding author is Xuefei Huang: huangxu2@msu.edu

Affiliations

[1]Department of Chemistry, Michigan State University, 578 S. Shaw Lane, East Lansing, Michigan 48824, USA

[2]Institute for Quantitative Health Science and Engineering, Michigan State University, East Lansing, Michigan 48824, USA

[3]Center for Cancer Immunology, Faculty of Pharmaceutical Sciences, Shenzhen Institute of Advanced Technology, Chinese Academy of Sciences (CAS), Shenzhen, Guangdong, 518000, China.

[4]School of Chemistry and Materials Science, Hangzhou Institute for Advanced Study, University of Chinese Academy of Sciences, Hangzhou, Zhejiang, 310024, China

[5]Chemical Biology Laboratory, Center for Cancer Research, National Cancer Institute, Frederick, MD 21702, USA

[6]Chemistry Department, Faculty of Science, Benha University, Benha, Qaliobiya 13518, Egypt

[7]Department of Microbiology, Genetics & Immunology, Michigan State University, East Lansing, Michigan 48824, USA

[8]Division of Infectious Diseases, Department of Medicine, Brigham and Women's Hospital, Harvard Medical School, Boston, MA 02115, USA

[9]Department of Biomedical Engineering, Michigan State University, East Lansing, Michigan 48824, USA

Inventory of supporting information:

Supplementary Methods 2

Supplementary Table 1 32

Supplementary Figures 1-117 33

Supplementary References 151

Supplementary Methods

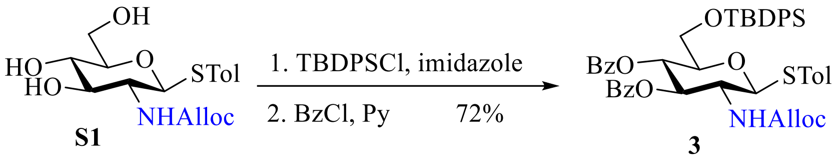

***p*-Tolyl 2-allyloxycarbonylamino-3,4-di-*O*-benzoyl-6-*O*-*tert*-butyldiphenylsilyl-2-deoxy-1-thio-β-D-glucopyranoside (3)**

To a solution of triol **S1**[1] (11.1 g, 30.0 mmol) in DCM (90 ml) was added imidazole (4.8 g, 2.5 equiv) and TBDPSCl (8.6 ml, 1.1 equiv). The reaction was stirred at room temperature for 6 h, after which it was extracted with ethyl acetate and washed with 10% HCl, sat. $NaHCO_3$ and brine. The organic phase was dried (Na_2SO_4) followed by filtration, and the filtrate was concentrated under reduced pressure. The residue was purified by flash column chromatography (hexanes/ethyl acetate = 1/1) to afford the 3,4-diol compound (15.5g, 80% yield). The resulting diol was dissolved in pyridine (50 ml) followed by the addition of benzoyl chloride (6.1 ml, 2.2 equiv) under ice bath. The reaction was stirred at room temperature for 12 h, after which it was extracted with ethyl acetate and washed with 10% HCl, sat. $NaHCO_3$ and brine. The organic phase was dried (Na_2SO_4) followed by filtration, and the filtrate was concentrated under reduced pressure. The residue was purified by flash column chromatography (hexanes/ethyl acetate = 5/1) to afford compound **3** (17.4 g, 91% yield). $[\alpha]_D^{20}$ = -21.2 (*c* 0.9, CH_2Cl_2); ^{1}H-NMR (500 MHz, $CDCl_3$): *δ*. 7.93 (d, *J* =7.5 Hz, 2 H), 7.84 (d, *J* =8.0 Hz, 2 H), 7.72 (d, *J* =7.5 Hz, 2 H), 7.58 (d, *J* =7.5 Hz, 2 H), 7.52-7.47 (m, 4 H), 7.35-7.28 (m, 8 H), 7.18-7.15 (m, 2 H), 7.07 (d, *J* =7.5 Hz, 2 H), 5.80-5.73 (m, 1 H, -$CH_2C\boldsymbol{H}CH_2$), 5.69-5.65 (m, 1 H, **H-3**), 5.59 (t, *J* =9.5 Hz, 1 H, **H-4**), 5.20 (d, *J* =17.5 Hz, 1 H, -$CH_2CHC\boldsymbol{H_2}$), 5.07-4.97 (m, 3 H, -$CH_2CHC\boldsymbol{H_2}$, **H-1** and -N***H***Alloc), 4.49 (bs, 2 H, -$C\boldsymbol{H_2}CHCH_2$), 3.92-3.79 (m, 4 H, **H-2, H-5** and **H-6**), 2.33 (s, 3 H, SPh-$C\boldsymbol{H_3}$), 1.03 (s, 9 H, -$C(C\boldsymbol{H_3})_3$). ^{13}C-NMR (125 MHz, $CDCl_3$): *δ*. 165.0, 163.2, 155.5, 133.3 (2 C), 133.2, 132.9, 130.2, 130.0, 129.8, 129.7, 129.6 (2 C), 129.2, 129.0, 128.5, 128.3 (2 C), 127.7, 127.6, 87.1, 74.4, 68.8, 68.7, 62.8, 55.4, 26.6, 21.2, 19.1. HRMS: $C_{47}H_{49}NNaO_8SSi$ $[M + Na]^+$ calcd: 838.2840, obsd: 838.2830.

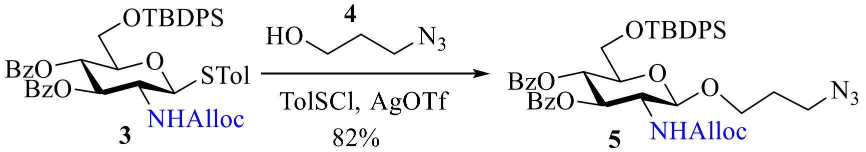

3-Azidopropyl 2-allyloxycarbonylamino-3,4-di-*O*-benzoyl-6-*O*-*tert*-butyldiphenylsilyl-2-deoxy-β-D-glucopyranoside (5)

A solution of donor **3** (1.63 g, 2 mmol), acceptor **4** (0.18 ml, 2 mmol) and freshly activated molecular sieve MS 4 Å in CH_2Cl_2 (DCM) (15 mL) was stirred at room temperature for 30 minutes, and cooled to −78 °C, which was followed by the addition of AgOTf (6 mmol) dissolved in Et_2O/DCM without touching the wall of the flask. After 5 minutes, orange colored *p*-TolSCl (290 μL, 2 mmol) was added to the solution through a microsyringe. The reaction mixture was

warmed to -20 °C under stirring in 1 h. Then the mixture was diluted with DCM and filtered over Celite. The filtrate was extracted with ethyl acetate and washed with $NaHCO_3$ and brine. The organic phase was dried (Na_2SO_4) followed by filtration, and the filtrate was concentrated under reduced pressure and purified by silica gel flash chromatography (hexanes/ethyl acetate = 5/1) to provide compound **5** (1.3 g, 82% yield). $[\alpha]_D^{20}$ = -47.3 (*c* 1.0, CH_2Cl_2); ^{1}H-NMR (500 MHz, $CDCl_3$): *δ*. 7.96 (d, *J* =8.0 Hz, 2 H), 7.86 (d, *J* =8.0 Hz, 2 H), 7.70 (d, *J* =8.0 Hz, 2 H), 7.59 (d, *J* =7.5 Hz, 2 H), 7.52-7.48 (m, 2 H), 7.39-7.31 (m, 8 H), 7.23 (t, *J* =7.5 Hz, 2 H), 5.82-5.76 (m, 1 H, -$CH_2C\boldsymbol{H}CH_2$), 5.66-5.64 (m, 1 H, **H-3**), 5.61 (t, *J* =9.5 Hz, 1 H, **H-4**), 5.21 (d, *J* =17.0 Hz, 1 H, -$CH_2CHC\boldsymbol{H}_2$), 5.10-5.05 (m, 2 H), 4.71 (bs, 1 H), 4.53-4.50 (m, 2 H, -$C\boldsymbol{H_2}CHCH_2$), 4.02-3.98 (m, 1 H, -$OC\boldsymbol{H}_2CH_2CH_2N_3$), 3.93-3.79 (m, 4 H), 3.65-3.61 (m, 1 H-$OC\boldsymbol{H}_2CH_2CH_2N_3$), 3.45-3.36 (m, 2 H, -$OCH_2CH_2C\boldsymbol{H}_2N_3$), 1.96-1.84 (m, 2 H, -$OCH_2CH_2C\boldsymbol{H}_2N_3$), 1.04 (s, 9 H, -$C(C\boldsymbol{H}_3)_3$). ^{13}C-NMR (125 MHz, $CDCl_3$): *δ*. 166.6, 165.0, 155.7, 135.6, 135.5, 133.3, 133.2, 133.1, 133.0, 132.5, 130.0, 129.7 (2 C), 129.6, 129.2, 129.0, 128.3, 127.6 (2 C), 117.6, 101.4, 75.0, 73.0, 69.2, 66.1, 65.7, 62.8, 56.4, 48.2, 29.1, 26.6, 19.2. HRMS: $C_{43}H_{52}N_5O_9Si$ $[M + NH_4]^+$ calcd: 810.3529, obsd: 810.3518.

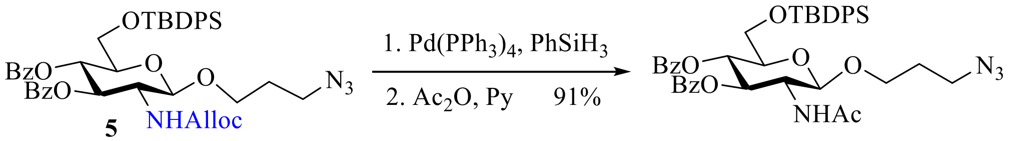

5 **6**

3-Azidopropyl 2-acetamido-3,4-di-*O*-benzoyl-6-*O*-*tert*-butyldiphenylsilyl-2-deoxy-β-D-glucopyranoside (6)

To a solution of compound **5** (1.6 g, 2 mmol) in DCM (20 mL) was added $Pd(PPh_3)_4$ (0.1 equiv) and phenylsilane (1 equiv). The reaction was stirred at room temperature for 5 hours, after which it was extracted with ethyl acetate and washed with $NaHCO_3$ and brine. The organic phase was dried (Na_2SO_4) followed by filtration, and the filtrate was concentrated under reduced pressure. The residue was dissolved in pyridine (5 mL) followed by addition of acetic anhydride (2 ml). The reaction was stirred at room temperature for 1 hour, after which it was quenched by methanol and the mixture was concentrated under reduced pressure and purified by silica gel flash chromatography (hexanes/ethyl acetate = 2/1) to provide compound **6** (1.37 g, 91% yield). $[\alpha]_D^{20}$ = -101.9 (*c* 1.0, CH_2Cl_2); ^{1}H-NMR (500 MHz, $CDCl_3$): *δ*. 7.96 (d, *J* =8.0 Hz, 2 H), 7.86 (d, *J* =8.5 Hz, 2 H), 7.71 (d, *J* =8.0 Hz, 2 H), 7.58 (d, *J* =8.0 Hz, 2 H), 7.51-7.48 (m, 2 H), 7.40-7.29 (m, 8 H), 7.21 (t, *J* =7.5 Hz, 2 H), 5.86-5.82 (br, 1 H, -N***H***Ac), 5.67-5.61 (m, 2 H, **H-3** and **H-4**), 4.75 (d, *J* =8.0 Hz, 1 H, **H-1**), 4.25-4.20 (m, 1 H, **H-2**), 4.02-3.98 (m, 1 H, -$OC\boldsymbol{H}_2CH_2CH_2N_3$), 3.86-3.82 (m, 3 H), 3.65-3.60 (m, 1 H, -$OC\boldsymbol{H}_2CH_2CH_2N_3$), 3.46-3.36 (m, 2 H, -$OCH_2CH_2C\boldsymbol{H}_2N_3$), 1.97-1.82 (m, 2 H, -$OCH_2C\boldsymbol{H}_2CH_2N_3$), 1.03 (s, 9 H, -$C(C\boldsymbol{H}_3)_3$). ^{13}C-NMR (125 MHz, $CDCl_3$): *δ*. 170.2, 167.0, 165.0, 135.7, 135.5, 133.5, 133.2, 133.1, 133.0, 129.9, 129.7, 129.6, 129.2, 128.8, 128.5, 128.4, 127.6 (2 C), 101.3, 75.0, 73.4, 69.1, 66.1, 65.7, 62.7, 54.8, 48.2, 29.1, 26.6, 23.4, 19.2. HRMS: $C_{41}H_{47}N_4O_8Si$ $[M + H]^+$ calcd: 751.3158, obsd: 751.3130.

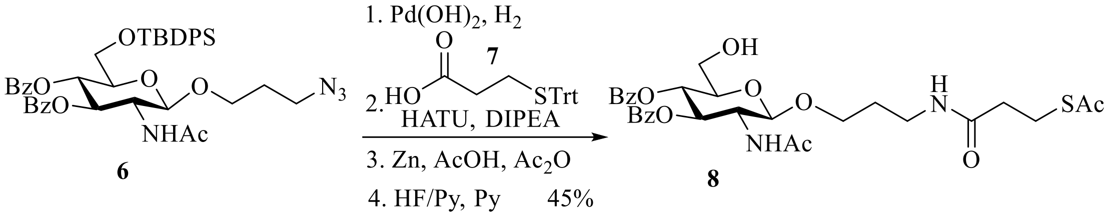

3-(3-(Acetylthio)propionylamino)propyl 2-acetamido-3,4-di-*O*-benzoyl-2-deoxy-β-D-glucopyranoside (8)

To a solution of compound **6** (1.5 g, 2 mmol) in MeOH/DCM (1:1, 16 mL) was added $Pd(OH)_2$ (300 mg). The mixture was hydrogenated with a hydrogen balloon for 5 hours. The reaction mixture was filtered through cotton and concentrated. The resultant amine and *S*-trityl-3-mercaptopropionic acid **7** (765 mg, 2.2 mmol) were dissolved in DMF (8 ml). Upon addition of HATU (910 mg, 1.2 equiv) and DIPEA (1 ml, 3 equiv), the reaction was stirred at room temperature for 1 hour or until TLC indicated the reaction was complete. The reaction mixture was extracted by ethyl acetate and washed by 10% HCl, $NaHCO_3$ (sat) and brine. The organic phase was concentrated, and the mixture was purified by silica gel column chromatography (hexanes/ethyl acetate = 1/1) to afford the desired amide product. To a solution of the amide in THF (9 ml), acetic anhydride (3 ml) and acetic acid (6 mL) was added zinc dust (1.3 g). The reaction was stirred at room temperature for 5 hours, after which it was filtered over Celite and the filtrate was concentrated under reduced pressure. The residue was dissolved in pyridine (5 mL) and 70% HF•Py (1 mL) was added at 0 °C. The reaction was stirred at room temperature for 1 h, after which it was neutralized by sat. $NaHCO_3$. The mixture was extracted with ethyl acetate and washed with 10% HCl, sat. $NaHCO_3$ and brine. The organic phase was dried (Na_2SO_4) followed by filtration, and the filtrate was concentrated under reduced pressure. The residue was purified by flash column chromatography (toluene/acetone = 20/1) to afford compound **8** (554 mg, 45% yield). $[\alpha]_D^{20}$ = -78.9 (*c* 1.0, CH_2Cl_2); ^{1}H-NMR (500 MHz, $CDCl_3$): *δ*. 7.90-7.88 (m, 4 H), 7.48-7.43 (m, 2 H), 7.37-7.26 (m, 4 H), 6.96 (d, *J* =8.5 Hz, 1 H, -N***H***Ac), 6.74-6.71 (m, 1 H, -N***H***C=O), 5.74 (t, *J* =10.0 Hz, 1 H, **H-3**), 5.44 (t, *J* =9.5 Hz, 1 H, **H-4**), 4.79 (d, *J* =8.0 Hz, 1 H, **H-1**), 4.30-4.24 (m, 1 H), 4.02-4.01 (m, 1 H), 3.83-3.69 (m, 3 H), 3.60-3.57 (m, 2 H, $-OCH_2CH_2C\boldsymbol{H}_2N_3$), 3.32 (bs, 1 H), 3.15-3.11 (m, 3 H), 2.51-2.49 (m, 2 H, $-NHC{=}OC\boldsymbol{H}_2-$), 2.23 (s, 3 H, S***Ac***), 1.88-1.70 (m, 5 H, NH***Ac*** and $-OCH_2C\boldsymbol{H}_2CH_2NH-$). ^{13}C-NMR (125 MHz, $CDCl_3$): *δ.* 196.2, 171.3, 171.0, 166.5, 166.0, 133.6, 133.4, 129.8, 129.0, 128.7, 128.5, 128.4, 101.5, 74.5, 73.2, 69.7, 67.6, 61.4, 54.4, 36.4, 36.0, 30.6, 29.4, 25.1, 23.2. HRMS: $C_{30}H_{37}N_2O_{10}S$ $[M + H]^+$ calcd: 617.2169, obsd: 617.2190.

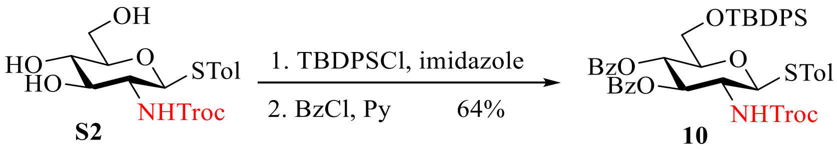

***p*-Tolyl 3,4-di-*O*-benzoyl-6-*O*-*tert*-butyldiphenylsilyl-2-deoxy-1-thio-2-(2,2,2-trichloroethyloxycarbonylamino)-β-D-glucopyranoside (10)**

To a solution of triol **S2**[1] (9.2 g, 20.0 mmol) in DCM (60 ml) was added imidazole (3.2 g, 2.5 equiv) and TBDPSCl (5.7 ml, 1.1 equiv). The reaction was stirred at room temperature for 6 h, after which it was extracted with ethyl acetate and washed with 10% HCl, sat. $NaHCO_3$ and brine. The organic phase was dried (Na_2SO_4) followed by filtration, and the filtrate was concentrated under reduced pressure. The residue was purified by flash column chromatography (hexanes/ethyl acetate = 1/1) to afford diol compound (11.9 g, 80% yield). The resulting compound was dissolved in pyridine (40 ml) followed by addition of benzoyl chloride (4.1 ml). The reaction was stirred at room temperature for 8 h, after which it was extracted with ethyl acetate and washed with 10% HCl, sat. $NaHCO_3$ and brine. The organic phase was dried (Na_2SO_4) followed by filtration, and the filtrate was concentrated under reduced pressure. The residue was purified by flash column chromatography (hexanes/ethyl acetate = 5/1) to afford compound **10** (11.6 g, 80% yield). $[\alpha]_D^{20}$ = -47.5 (*c* 1.5, CH_2Cl_2); ^{1}H-NMR (500 MHz, $CDCl_3$): δ 7.93 (d, *J* =8.0 Hz, 2 H), 7.84 (d, *J* =7.5 Hz, 2 H), 7.72 (d, *J* =7.0 Hz, 2 H), 7.58 (d, *J* =7.0 Hz, 2 H), 7.53-7.47 (m, 4 H), 7.35-7.28 (m, 8 H), 7.18-7.15 (m, 2 H), 7.08 (d, *J* =7.5 Hz, 2 H), 5.72 (t, *J* =10.0 Hz, 1 H, **H-3**), 5.62 (t, *J* =9.0 Hz, 1 H, **H-4**), 5.41 (d, *J* =9.0 Hz, 1 H, -N***H***Troc), 4.97 (d, *J* =10.5 Hz, 1 H, **H-1**), 4.74 (d, *J* =12.0 Hz, 1 H, -C***H***$_2$CCl$_3$), 4.62 (d, *J* =12.0 Hz, 1 H, -C***H***$_2$CCl$_3$), 3.99-3.93 (m, 1 H, **H-2**), 3.84-3.82 (m, 3 H, **H-5** and **H-6**), 2.33 (s, 3 H, SPh-C***H***$_3$), 1.04 (s, 9 H, -C(C***H***$_3$)$_3$). ^{13}C-NMR (125 MHz, $CDCl_3$): δ. 166.5, 164.9, 154.0, 138.4, 135.7, 135.5, 133.6, 133.4, 133.2, 132.8, 130.2, 130.0, 129.8, 129.7 (2 C), 129.6, 129.1, 128.8, 128.5, 128.4, 127.7, 127.6, 95.3, 87.1, 79.2, 74.4, 74.3, 68.7, 62.7, 55.5, 26.6, 21.2, 19.1. HRMS: $C_{46}H_{50}Cl_3N_2O_8SSi$ $[M + NH_4]^+$ calcd: 923.2123, obsd: 923.2120.

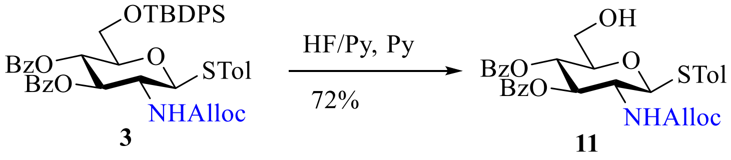

***p*-Tolyl 2-allyloxycarbonylamino-3,4-di-*O*-benzoyl-2-deoxy-1-thio-β-D-glucopyranoside (11)**
To compound **3** (1.6 g, 2 mmol) dissolved in pyridine (5 mL) was added 70% HF•Py (1 mL) at 0 ^{o}C. The reaction was stirred at room temperature for 1 h, after which it was neutralized by sat. $NaHCO_3$. The mixture was extracted with ethyl acetate and washed with 10% HCl, sat. $NaHCO_3$ and brine. The organic phase was dried (Na_2SO_4) followed by filtration, and the filtrate was concentrated under reduced pressure. The residue was purified by flash column chromatography (hexanes/ethyl acetate = 5/2) to afford compound **11** (831 mg, 72% yield). $[\alpha]_D^{20}$ = -52.9 (*c* 1.0, CH_2Cl_2); ^{1}H-NMR (500 MHz, $CDCl_3$): δ. 7.90 (d, *J* =7.0 Hz, 4 H), 7.49-7.45 (m, 4 H), 7.34-7.21 (m, 4 H), 7.14-7.11 (m, 2 H), 5.81-5.70 (m, 2 H, N***H***Alloc and -CH$_2$C***H***CH$_2$), 5.43 (t, *J* =9.5 Hz, 1 H, **H-3**), 5.34 (d, *J* =9.5 Hz, 1 H, **H-4**), 5.16 (d, *J* =17.0 Hz, 1 H, -CH$_2$CHC***H***$_2$), 5.05-5.01 (m, 2 H, **H-1** and -CH$_2$C***H***CH$_2$), 4.47 (bs, 2 H, -C***H***$_2$CHCH$_2$), 3.96-3.88 (m, 1 H), 3.82-3.68 (m, 3 H), 2.59 (bs, 1 H, -O**H**), 2.33 (s, 3 H, SPhC***H***$_3$). ^{13}C-NMR (125 MHz, $CDCl_3$): δ. 166.5, 165.9, 155.6, 138.5, 133.6, 133.4, 132.5, 129.9 (2 C), 129.8, 128.9, 128.6, 128.5, 128.4, 128.3, 117.4, 86.9, 78.5, 73.9, 69.4, 65.7, 61.7, 55.3, 21.2, 19.1. HRMS: $C_{31}H_{35}N_2O_8S$ $[M + NH_4]^+$ calcd: 595.2109, obsd: 595.2090.

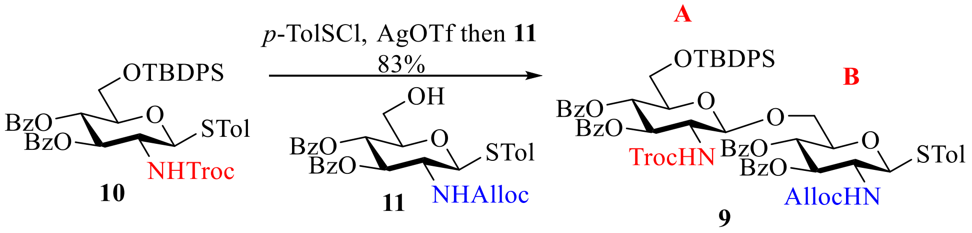

p-Tolyl 3,4-di-O-benzoyl-6-O-tert-butyldiphenylsilyl-2-deoxy-2-(2,2,2-trichloroethyloxycarbonylamino)-β-D-glucopyranosyl-(1→6)-2-allyloxycarbonylamino-3,4-di-O-benzoyl-2-deoxy-1-thio-β-D-glucopyranoside (9)

A solution of donor **10** (905 mg, 1 mmol) and freshly activated molecular sieve MS 4 Å (100 mg) in DCM (5 mL) was stirred at room temperature for 30 minutes, and cooled to −78 °C, which was followed by addition of AgOTf (3 mmol) dissolved in Et_2O/DCM without touching the wall of the flask. After 5 minutes, orange colored *p*-TolSCl (144 µL, 1 mmol) was added to the solution through a microsyringe. After the donor was completely consumed according to TLC analysis (~ 10 minutes), a solution of acceptor **11** (519 mg, 0.9 mmol) in DCM (0.5 mL) was slowly added dropwise via a syringe. The reaction mixture was warmed to -20 °C under stirring in 1 h. Then the mixture was diluted with DCM and filtered over Celite. The filtrate was extracted with ethyl acetate and washed with $NaHCO_3$ and brine. The organic phase was dried (Na_2SO_4) followed by filtration, and the filtrate was concentrated under reduced pressure and purified by silica gel flash chromatography (hexanes/ethyl acetate = 4/1) to afford compound **9** (1 g, 83% yield). $[\alpha]_D^{20}$ = -59.1 (*c* 2.0, CH_2Cl_2); ^{1}H-NMR (500 MHz, $CDCl_3$): *δ*. 7.96-7.87 (m, 8 H), 7.68 (d, *J* =6.5 Hz, 2 H), 7.56-7.47 (m, 8 H), 7.39-7.28 (m, 14 H), 7.20 (t, *J* =7.5 Hz, 2 H), 5.79-5.73 (m, 2 H, **BH-3** and -$CH_2C\boldsymbol{H}CH_2$), 5.57-5.47 (m, 2 H, **AH-3** and **AH-4**), 5.39-5.36 (m, 1 H, **BH-4**), 5.20-5.03 (m, 5 H, **BH-1**, -N**H**Alloc, -N**H**Troc and -$CH_2CHC\boldsymbol{H}_2$), 4.69 (d, *J* =12.0 Hz, 1 H, -$C\boldsymbol{H}_2CCl_3$), 4.62 (d, *J* =8.5 Hz, 1 H, **AH-1**), 4.56-4.50 (m, 3 H, -$C\boldsymbol{H}_2CCl_3$ and -$C\boldsymbol{H}_2CHCH_2$), 4.04-3.98 (m, 2 H), 3.94-3.91 (m, 1 H), 3.81-3.73 (m, 4 H), 3.70-3.67 (m, 1 H), 2.36 (s, 3 H, SPhC$\boldsymbol{H}_3$), 1.01 (s, 9 H, -C(C$\boldsymbol{H}_3$)$_3$). ^{13}C-NMR (125 MHz, $CDCl_3$): *δ*. 166.3, 166.2, 165.5, 165.0, 155.3, 154.5, 138.9, 135.7, 135.5, 133.7 (2 C), 133.4, 133.3, 133.2, 133.1, 132.9, 132.4, 130.3, 130.0, 129.9, 129.8, 129.7, 129.6, 129.3, 129.0, 128.8, 128.6, 128.5, 128.4, 128.3, 128.0, 127.6 (2 C), 117.5, 101.5, 95.5, 86.8, 78.5, 74.9, 74.3, 73.8, 73.2, 69.3, 69.2, 67.5, 65.8, 62.6, 56.5, 55.3, 26.6, 21.2, 19.1. HRMS: $C_{70}H_{73}Cl_3N_3O_{16}SSi$ $[M + NH_4]^+$ calcd: 1376.3541, obsd: 1376.3501.

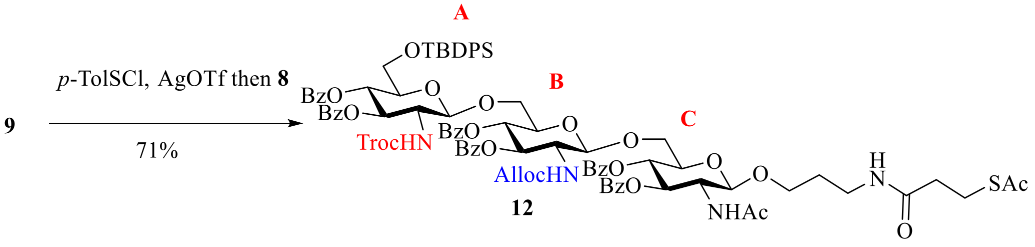

3-(3-(Acetylthio)propionylamino)propyl 3,4-di-*O*-benzoyl-6-*O*-*tert*-butyldiphenylsilyl-2-deoxy-2-(2,2,2-trichloroethyloxycarbonylamino)-β-D-glucopyranosyl-(1→6)-2-allyloxycarbonylamino-3,4-di-*O*-benzoyl-2-deoxy-β-D-glucopyranosyl-(1→6)-2-acetamido-3,4-di-*O*-benzoyl-2-deoxy-β-D-glucopyranoside (12)

Following the general procedure for pre-activation based glycosylation, compound **12** was prepared from donor **9** and acceptor **8** (purified using toluene/acetone = 20/1, 71% yield). $[\alpha]_D^{20}$ = -3.5 (*c* 0.2, CH_2Cl_2); ^{1}H-NMR (500 MHz, $CDCl_3$): *δ*. 8.00-7.86 (m, 12 H), 7.54-7.11 (m, 28 H), 6.53 (d, *J* =6.5 Hz, 1 H, -N***H***C=O), 6.45 (bs, 1 H, -N***H***C=O), 6.19 (d, *J* =9.0 Hz, 1 H, -N***H***C=O), 5.69-5.59 (m, 5 H), 5.47-5.45 (m, 1 H), 5.29 (t, *J* =9.0 Hz, 1 H), 5.16 (d, *J* =14.5 Hz, 1 H, -CH_2CHC***H***$_2$), 5.03 (d, *J* =10.0 Hz, 1 H, -CH_2CHC***H***$_2$), 4.75-4.73 (m, 2 H, **AH-1** and -C***H***$_2$$CCl_3$), 4.66 (d, *J* =7.0 Hz, 1 H, **BH-1**), 4.60 (d, *J* =7.0 Hz, 1 H, **CH-1**), 4.52-4.48 (m, 3 H, -C***H***$_2$$CCl_3$ and -C***H***$_2$CHCH_2), 4.24 (d, *J* =7.5 Hz, 1 H), 4.15-4.09 (m, 2 H), 4.04-4.00 (m, 3 H), 3.97-3.88 (m, 3 H), 3.82-3.70 (m, 4 H), 3.61 (bs, 3 H), 3.21-3.18 (m, 3 H, -C***H***$_2$NH=O and -C***H***$_2$SAc), 2.60-2.55 (m, 2 H, -NHC=OC***H***$_2$-), 2.31 (s, 3 H, **-SAc**), 1.84-1.76 (m, 5 H, -NH**Ac** and -OCH_2C***H***$_2$$CH_2$NH-), 0.96 (s, 9 H, -C(C***H***$_3$)$_3$). ^{13}C-NMR (125 MHz, $CDCl_3$): *δ*. 196.8, 171.1, 170.7, 166.6, 166.3, 165.5, 164.9, 155.9, 154.7, 135.5, 135.4, 133.7, 133.6, 133.4, 133.3, 133.2, 133.0 (2 C), 132.7, 132.6, 130.0, 129.9 (2 C), 129.7 (2 C), 129.6, 129.3, 129.0 (2 C), 128.9, 128.7, 128.6 (2 C), 128.5, 128.4 (2 C), 128.3 (2 C), 127.6, 127.5, 117.4, 102.4, 102.1, 101.2, 95.6, 75.3, 74.1, 73.5, 73.2, 73.1, 70.4, 69.8, 69.3, 69.2, 67.9, 65.7, 64.0, 62.9, 56.6, 56.3, 54.6, 36.8, 35.9, 30.6, 29.7, 26.6, 25.3, 23.2, 19.0. HRMS: $C_{93}H_{98}Cl_3N_4O_{26}SSi$ $[M + H]^+$ calcd: 1851.5019, obsd: 1851.4938.

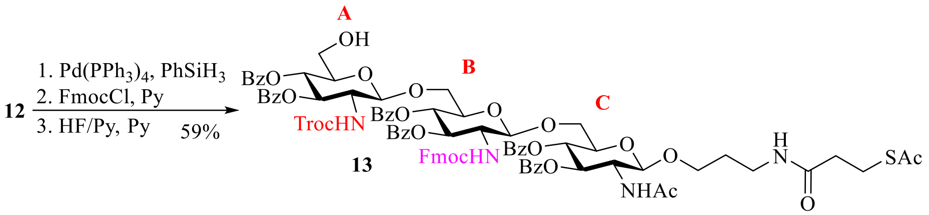

3-(3-(Acetylthio)propionylamino)propyl 3,4-di-*O*-benzoyl-2-deoxy-2-(2,2,2-trichloroethyloxycarbonylamino)-β-D-glucopyranosyl-(1→6)-3,4-di-*O*-benzoyl-2-deoxy-2-fluorenylmethyloxycarbonylamino-β-D-glucopyranosyl-(1→6)-2-acetamido-3,4-di-*O*-benzoyl-2-deoxy-β-D-glucopyranoside (13)

To a solution of compound **12** (555 mg, 0.3 mmol) in DCM (5 mL) was added $Pd(PPh_3)_4$ (0.1 equiv) and phenylsilane (1 equiv). The filtrate was extracted with ethyl acetate and washed with $NaHCO_3$ and brine. The organic phase was dried (Na_2SO_4), filtered, and the filtrate was concentrated under reduced pressure. The residue was dissolved in pyridine (5 mL) followed by the addition of FmocCl (512 mg, 0.9 mmol) at 0°C. The reaction was stirred at room temperature for 5 hours, after which it was quenched by methanol. The mixture was extracted with ethyl acetate and washed with $NaHCO_3$ and brine. The organic phase was dried (Na_2SO_4) followed by filtration. The filtrate was concentrated under reduced pressure and purified by silica gel flash chromatography. The resultant compound was dissolved in pyridine (2 mL) followed by the addition of 70% HF•Py (0.5 mL) at 0 °C. The reaction was stirred at room temperature for 3 h,

after which it was neutralized by sat. $NaHCO_3$. The mixture was extracted with ethyl acetate and washed with 10% HCl, sat. $NaHCO_3$ and brine. The organic phase was dried (Na_2SO_4) followed by filtration, and the filtrate was concentrated under reduced pressure. The residue was purified by flash column chromatography (toluene/acetone = 12/1) to afford compound **13** (310 mg, 59% yield). $[\alpha]_D^{20}$ = -8.0 (*c* 0.3, CH_2Cl_2); ^{1}H-NMR (500 MHz, $CDCl_3$): *δ*. 7.99-7.86 (m, 12 H), 7.72 (d, *J* =7.5 Hz, 2 H), 7.68 (dd, *J* =7.5, 12.0 Hz, 1 H), 7.55-7.27 (m, 21 H), 7.20 (bs, 1 H), 7.12 (bs, 1 H), 6.66 (bs, 1 H, -N***H***C=O), 6.45 (bs, 1 H, -N***H***C=O), 6.13 (d, *J* =7.5 Hz, 1 H, -N***H***C=O), 5.82-5.68 (m, 4 H), 5.42-5.38 (m, 3 H), 4.92 (d, *J* =6.5 Hz, 1 H, **AH-1**), 4.77-4.70 (m, 2 H, **BH-1** and **CH-1**), 4.65 (d, *J* =12.0 Hz, 1 H, -C$\boldsymbol{H}_2$$CCl_3$), 4.57 (d, *J* =12.0 Hz, 1 H, -C$\boldsymbol{H}_2$$CCl_3$), 4.51 (t, *J* =4.5 Hz, 1 H), 4.28-4.20 (m, 3 H), 4.12-3.88 (m, 8 H), 3.79-3.58 (m, 8 H), 3.20-3.14 (m, 3 H, -C$\boldsymbol{H}_2$NH=O and -C$\boldsymbol{H}_2$SAc), 2.52 (bs, 2 H, -NHC=OC$\boldsymbol{H}_2$-), 2.26 (s, 3 H, **-SAc**), 1.84 (s, 3 H, -NH**Ac**), 1.77 (bs, 1 H, -OCH_2C$\boldsymbol{H}_2$$CH_2$NH-), 1.71 (bs, 1 H, -$OCH_2C\boldsymbol{H}_2$$CH_2$NH-). ^{13}C-NMR (125 MHz, $CDCl_3$): *δ*. 196.7, 171.1, 170.9, 166.6, 166.1, 166.0, 165.7, 155.9, 154.6, 143.8, 141.1, 133.6, 133.4, 133.3, 133.2, 133.0, 132.1, 130.0 (3 C), 129.9 (2 C), 129.8, 129.7, 128.9 (2 C), 128.8, 128.6, 128.5 (2 C), 128.4 (2 C), 128.3, 127.6, 127.0, 119.8, 101.7, 101.5, 101.3, 95.5, 74.7, 74.1, 73.4, 73.2, 72.6, 69.7, 66.8, 64.0, 61.2, 56.4, 54.4, 47.0, 36.8, 35.9, 30.6, 29.3, 25.2, 23.2. HRMS: $C_{88}H_{86}Cl_3N_4O_{26}S$ $[M + H]^+$ calcd: 1751.4311, obsd: 1751.4387.

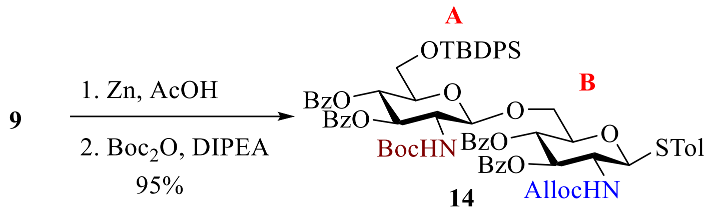

***p*-Tolyl 3,4-di-*O*-benzoyl-6-*O*-*tert*-butyldiphenylsilyl-2-*tert*-butyloxycarbonylamino-2-deoxy-β-D-glucopyranosyl-(1→6)-2-allyloxycarbonylamino-3,4-di-*O*-benzoyl-2-deoxy-1-thio-β-D-glucopyranoside (14)**

To a solution of disaccharide **9** (1.36 g, 1 mmol) in THF and acetic acid (3:1, 12 mL) was added zinc dust (1.3 g, 20 mmol). The reaction was stirred at room temperature for 6 hours, after which it was filtered by celite. The filtrate was extracted with ethyl acetate and washed with sat. $NaHCO_3$ (two times) and brine. The organic phase was dried (Na_2SO_4) followed by filtration, and the filtrate was concentrated under reduced pressure. The resultant residue and Boc_2O (536 mg, 2 mmol) were dissolved in DCM (6 ml) followed by addition of DIPEA (0.2 ml) at 0°C. The reaction was stirred at room temperature for 5 hours. Then the solvent was removed and the resultant residue was purified by flash column chromatography (hexanes/ethyl acetate = 4/1) to afford compound **14** (1.2 g, 95% yield). $[\alpha]_D^{20}$ = -60.9 (*c* 1.5, CH_2Cl_2); ^{1}H-NMR (500 MHz, $CDCl_3$): *δ*. 8.00 (d, *J* =7.0 Hz, 2 H), 7.94-7.89 (m, 6 H), 7.69-7.67 (m, 2 H), 7.58-7.48 (m, 8 H), 7.40-7.26 (m, 14 H), 7.22 (t, *J* =7.5 Hz, 2 H), 5.80-5.74 (m, 2 H, **BH-3** and -CH_2C***H***CH_2), 5.56-5.47 (m, 2 H, **AH-3** and **AH-4**), 5.39 (t, *J* =9.5 Hz, 1 H, **BH-4**), 5.21 (d, *J* =17.5 Hz, 1 H, -CH_2CHC$\boldsymbol{H}_2$), 5.12-5.07 (m, 3 H, **BH-1**, -N***H***Alloc and -CH_2CHC$\boldsymbol{H}_2$), 4.66 (d, *J* =7.5 Hz, 1 H, **AH-1**), 4.54-4.51 (m, 3 H, -N***H***Boc and -C$\boldsymbol{H}_2$CHCH_2), 4.04 (d, *J* =11.5 Hz, 1 H), 3.97-3.79 (m, 6 H), 3.71 (d, *J* =9.0 Hz, 1 H), 2.36

(s, 3 H, SPhC***H***$_3$), 1.30 (s, 9 H, -C(C***H***$_3$)$_3$), 1.02 (s, 9 H, -C(C***H***$_3$)$_3$). ^{13}C-NMR (125 MHz, $CDCl_3$): *δ*. 166.3, 165.4, 165.1, 155.5, 155.4, 138.8, 135.7, 135.5, 133.7, 133.6, 133.4, 133.2, 133.1 (2 C), 132.9, 132.5, 130.2, 130.0, 129.9 (2 C), 129.8, 129.7, 129.6, 129.4 (2 C), 128.9, 128.7, 128.5, 128.3 (2 C), 128.1, 127.6 (2 C), 117.4, 101.7, 87.0, 79.6, 78.7, 74.9, 73.9, 73.7, 69.5, 69.4, 67.7, 65.8, 62.7, 55.8, 55.3, 28.2, 26.6, 21.2, 19.2. HRMS: $C_{72}H_{80}N_3O_{16}SSi$ $[M + NH_4]^+$ calcd: 1302.5023, obsd: 1302.5016.

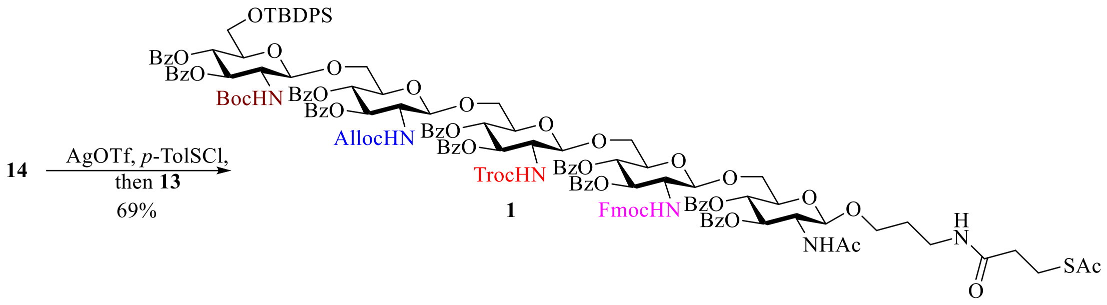

3-(3-(Acetylthio)propionylamino)propyl 3,4-di-*O*-benzoyl-6-*O*-*tert*-butyldiphenylsilyl-2-*tert*-butyloxycarbonylamino-2-deoxy-β-D-glucopyranosyl-(1→6)-2-allyloxycarbonylamino-3,4-di-*O*-benzoyl-2-deoxy-β-D-glucopyranosyl-(1→6)-3,4-di-*O*-benzoyl-2-deoxy-2-(2,2,2-trichloroethyloxycarbonylamino)-β-D-glucopyranosyl-(1→6)-3,4-di-*O*-benzoyl-2-deoxy-2-fluorenylmethyloxycarbonylamino-β-D-glucopyranosyl-(1→6)-2-acetamido-3,4-di-*O*-benzoyl-2-deoxy-β-D-glucopyranoside (1)

Following the general procedure for pre-activation based glycosylation, compound **1** was prepared from donor **14** and acceptor **13** (purified with toluene/acetone = 10/1, 69% yield). $[\alpha]_D^{20}$ = -53.1 (*c* 0.8, CH_2Cl_2); ^{1}H-NMR (500 MHz, $CDCl_3$): *δ*. 8.32 (d, *J* =7.5 Hz, 2 H), 8.27-8.24 (m, 4 H), 8.12-7.92 (m, 12 H), 7.87 (d, *J* =7.5 Hz, 1 H), 7.70-6.85 (m, 46 H), 6.80 (t, *J* =7.5 Hz, 1 H), 6.64 (bs, 1 H, -N***H***C=O), 6.01-5.89 (m, 3 H), 5.83 (t, *J* =9.5 Hz, 1 H), 5.71 (t, *J* =9.5 Hz, 1 H), 5.44-5.37 (m, 3 H, -CH$_2$C***H***CH$_2$), 5.25 (t, *J* =10.0 Hz, 1 H), 5.18 (t, *J* =9.5 Hz, 1 H), 5.09 (d, *J* =8.5 Hz, 1 H, anomeric H), 4.96-4.73 (m, 7 H, two anomeric H and -CH$_2$CHC***H***$_2$), 4.59-4.50 (m, 2 H), 4.43-4.11 (m, 10 H, anomeric H), 3.96-3.87 (m, 3 H, anomeric H), 3.79-3.67 (m, 3 H), 3.62-3.55 (m, 3 H), 3.41-3.17 (m, 4 H), 2.68-2.64 (m, 2 H, -NHC=OC***H***$_2$-), 2.28 (s, 3 H, **-SAc**), 1.90-1.87 (m, 5 H, -NH**Ac** and -OCH$_2$C***H***$_2$CH$_2$NH-), 1.17 (s, 9 H, -C(C***H***$_3$)$_3$), 1.07 (s, 9 H, -C(C***H***$_3$)$_3$). ^{13}C-NMR (125 MHz, $CDCl_3$): *δ*. 195.9, 171.2 (2 C), 166.9, 166.8, 166.7 (2 C), 166.4, 166.3, 166.0, 165.7, 165.0, 164.6, 156.6, 156.3, 156.0, 155.6, 144.2, 143.5, 141.0, 140.6, 135.6, 135.3, 133.4 (2 C), 133.3, 133.2, 133.1, 133.0, 132.9, 132.8, 132.1 (2 C), 132.0, 131.9, 131.8, 131.4, 130.5, 130.4, 130.3, 130.2, 130.0, 129.9, 129.8 (2 C), 129.7, 129.6 (2 C), 129.2 (2 C), 129.0, 128.9, 128.7 (2 C), 128.6, 128.5 (2 C), 128.4, 128.3 (2 C), 128.2, 128.1, 28.0, 127.7, 127.3, 127.2, 127.1, 126.7, 125.3, 125.1, 119.4, 119.3, 117.7, 105.4, 105.3, 103.0, 102.3, 100.5, 95.4, 80.7, 74.6, 74.5, 74.2, 73.9, 73.4, 73.2, 72.7, 72.3, 72.1, 72.0, 71.7, 70.8, 69.6, 69.2, 67.7, 66.5, 66.3, 64.6, 64.0, 57.4, 56.6, 56.3, 55.8, 54.2, 47.0, 37.0, 35.5, 30.5, 29.1, 28.0, 26.8, 25.2, 22.7. HRMS: $C_{153}H_{155}Cl_3N_6O_{42}SSi$ $[M + 2H]^{2+}$ calcd: 1456.4361, obsd: 1456.4336.

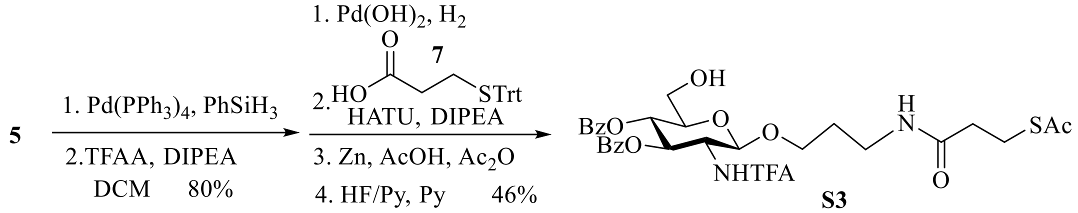

3-(3-(Acetylthio)propionylamino)propyl 3,4-di-*O*-benzoyl-2-deoxy-2-trifluoroacetamido-β-D-glucopyranoside (S3)

Compound **S3** was synthesized analogously as compound **8**. To a solution of compound **5** (1.6 g, 2 mmol) in DCM (20 mL) was added $Pd(PPh_3)_4$ (0.1 equiv) and phenylsilane (1 equiv). The reaction was stirred at room temperature for 5 hours, after which it was extracted with ethyl acetate and washed with $NaHCO_3$ and brine. The organic phase was dried (Na_2SO_4) followed by filtration, and the filtrate was concentrated under reduced pressure. The residue was dissolved in DCM (10 mL) and DIPEA (1.7 ml) followed by addition of trifluoroacetic anhydride (0.3 ml, 2.2 mmol) at -78°C. The reaction was stirred at room temperature for 1 hour, after which it was quenched by methanol and the mixture was concentrated under reduced pressure and purified by silica gel flash chromatography (hexanes/ethyl acetate = 2/1) to provide the desired product. To a solution of above compound (1.6 g, 2 mmol) in MeOH/DCM (1:1, 16 mL) was added $Pd(OH)_2$ (300 mg). The mixture was hydrogenated with a hydrogen balloon for 5 hours. The reaction mixture was filtered through cotton and concentrated. The resultant amine and S-trityl-3-mercaptopropionic acid **7** (765 mg, 2.2 mmol) were dissolved in DMF (8 ml). Upon addition of HATU (912 mg, 1.2 equiv) and DIPEA (1 ml, 3 equiv). The reaction was stirred at room temperature for 1 hour or until TLC indicated the reaction was complete. The reaction mixture was extracted by ethyl acetate and washed by 10% HCl, $NaHCO_3$ (sat) and brine. The organic phase was concentrated, and the mixture was purified by silica gel column chromatography (hexanes/ethyl acetate = 1/1) to afford the desired product. To a solution of the above compound in THF (9 ml), acetic anhydride (3 ml) and acetic acid (6 mL) was added zinc dust (1.3 g). The reaction was stirred at room temperature for 5 hours, after which it was filtered over Celite, and the filtrate was concentrated under reduced pressure. The residue was dissolved in pyridine (5 mL) followed by the addition of 70% HF•Py (1 mL) at 0 °C. The reaction was stirred at room temperature for 1 h, after which it was neutralized by sat. $NaHCO_3$. The mixture was extracted with ethyl acetate and washed with 10% HCl, sat. $NaHCO_3$ and brine. The organic phase was dried (Na_2SO_4) followed by filtration, and the filtrate was concentrated under reduced pressure. The residue was purified by flash column chromatography (toluene/acetone = 20/1) to afford compound **S3** (493 mg, 45% yield). $[\alpha]_D^{20}$ = -55.4 (*c* 1.0, CH_2Cl_2); ^{1}H-NMR (500 MHz, $CDCl_3$): *δ*. 8.21 (d, *J* =8.5 Hz, 1 H), 7.93-7.90 (m, 4 H), 7.53-7.47 (m, 2 H), 7.38-7.33 (m, 4 H), 6.18 (bs, 1 H, -N**H**TFA), 5.79 (t, *J* =10.0 Hz, 1 H, **H-3**), 5.49 (t, *J* =9.5 Hz, 1 H, **H-4**), 4.84 (d, *J* =7.5 Hz, 1 H, **H-1**), 4.34-4.29 (m, 1 H), 4.07-4.03 (m, 1 H), 3.86-3.71 (m, 3 H), 3.67-3.60 (m, 1 H), 3.55-3.51 (m, 1 H, -C***H***$_2$NH=O), 3.19-3.10 (m, 3 H, -C***H***$_2$NH=O and -C***H***$_2$SAc), 2.56-2.44 (m, 2 H, -NHC=OC***H***$_2$-), 2.31 (s, 3 H, -S**Ac**), 1.85-1.82 (m, 1 H, -OCH$_2$C***H***$_2$CH$_2$NH-), 1.67-1.63 (m, 1 H, -OCH$_2$C***H***$_2$CH$_2$NH-). ^{13}C-NMR (125 MHz, $CDCl_3$): *δ*. 196.7, 171.3, 166.5, 166.1, 158.4 (q, *J* =37.5 Hz, C=O of TFA), 133.8, 133.5, 129.9, 129.8,

128.6, 128.5 (2 C), 128.4, 119.1 (q, J =286 Hz, CF_3 of TFA), 101.0, 74.7, 72.7, 69.4, 67.9, 61.2, 55.0, 36.4, 36.1, 30.6, 29.6, 25.2. HRMS: $C_{30}H_{34}F_3N_2O_{10}S$ $[M + H]^+$ calcd: 671.1881, obsd: 671.1889.

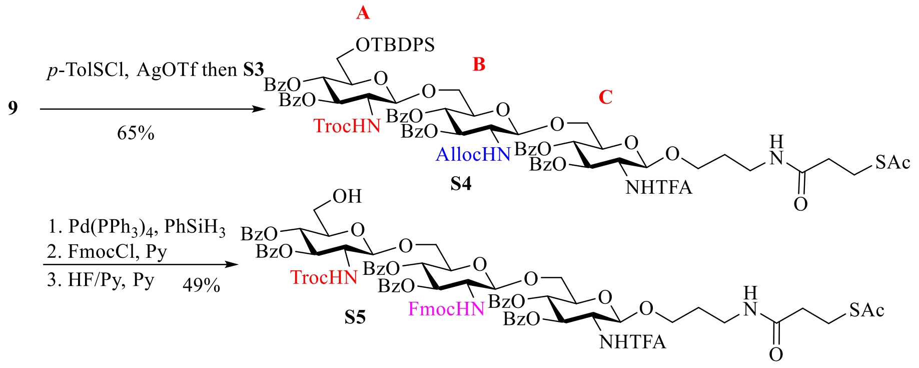

3-(3-(Acetylthio)propionylamino)propyl 3,4-di-*O*-benzoyl-6-*O*-*tert*-butyldiphenylsilyl-2-deoxy-2-(2,2,2-trichloroethyloxycarbonylamino)-β-D-glucopyranosyl-(1→6)-2-allyloxycarbonylamino-3,4-di-*O*-benzoyl-2-deoxy-β-D-glucopyranosyl-(1→6)-3,4-di-*O*-benzoyl-2-deoxy-2-trifluoroacetamido-β-D-glucopyranoside (S4)

Following the general procedure for pre-activation based glycosylation, compound **S4** was prepared from donor **9** and acceptor **S3** (purified with toluene/acetone = 20/1, 65% yield). $[\alpha]_D^{20}$ = -2.9 (c 0.2, CH_2Cl_2); ^{1}H-NMR (500 MHz, $CDCl_3$): δ. 8.00-7.86 (m, 12 H), 7.56-7.10 (m, 28 H), 6.17 (bs, 1 H, -N*H*C=O), 5.95 (bs, 1 H, -N*H*C=O), 5.80-5.43 (m, 5 H), 5.28-5.04 (m, 4 H), 4.84 (d, J =8.0 Hz, 1 H, **CH-1**), 4.71 (d, J =12.0 Hz, 1 H, -C*H*$_2$CCl$_3$), 4.64 (d, J =8.0 Hz, 1 H, **AH-1**), 4.55-4.48 (m, 4 H, **BH-1**, -C*H*$_2$CCl$_3$ and -C*H*$_2$CHCH$_2$), 4.37-4.31 (m, 1 H), 4.21-4.16 (m, 2 H), 4.05-3.86 (m, 5 H), 3.76-3.57 (m, 5 H), 3.21-3.18 (m, 3 H, -C*H*$_2$NH=O and -C*H*$_2$SAc), 2.58-2.52 (m, 2 H, -NHC=OC*H*$_2$-), 2.32 (s, 3 H, **-SAc**), 1.86 (bs, 1 H, -OCH$_2$C*H*$_2$CH$_2$NH-), 1.73 (bs, 1 H, -OCH$_2$C*H*$_2$CH$_2$NH-), 0.99 (s, 9 H, -C(C*H*$_3$)$_3$). ^{13}C-NMR (125 MHz, $CDCl_3$): δ. 196.8, 171.1, 166.4, 166.2, 166.1, 165.5, 164.9, 158.3 (q, J =37.5 Hz, C=O of TFA), 155.9, 154.8, 135.5, 135.4, 133.8, 133.7, 133.4 (2 C), 133.3 (2 C), 133.0, 132.6, 130.0, 129.9 (2 C), 129.7 (2 C), 129.6, 129.2, 128.9 (2 C), 128.6 (2 C), 128.5, 128.4 (2 C), 128.3 (2 C), 127.6, 127.5, 119.1 (q, J =286.5 Hz, CF_3 of TFA), 117.4, 102.8, 102.4, 100.6, 95.4, 75.7, 74.2, 73.5, 73.3, 73.0, 72.6, 70.4, 69.9, 69.3, 68.2, 65.7, 63.2, 56.7, 56.2, 54.9, 36.5, 36.1, 30.6, 29.4, 26.6, 25.2, 19.0. HRMS: $C_{93}H_{95}Cl_3F_3N_4O_{26}SSi$ $[M + H]^+$ calcd: 1905.4742, obsd: 1905.4798.

3-(3-(Acetylthio)propionylamino)propyl 3,4-di-*O*-benzoyl-2-deoxy-2-(2,2,2-trichloroethyloxycarbonylamino)-β-D-glucopyranosyl-(1→6)-3,4-di-*O*-benzoyl-2-deoxy-2-fluorenylmethyloxycarbonylamino-β-D-glucopyranosyl-(1→6)-3,4-di-*O*-benzoyl-2-deoxy-2-trifluoroacetamido-β-D-glucopyranoside (S5)

Following the procedure for synthesis of compound **13**, compound **S5** (toluene/acetone = 15/1, 49% yield) was prepared from compound **S4**. $[\alpha]_D^{20}$ = -1.0 (c 0.1, CH_2Cl_2); ^{1}H-NMR (500 MHz, $CDCl_3$): δ. 8.27 (bs, 1 H), 8.01-7.87 (m, 12 H), 7.72 (d, J =7.5 Hz, 2 H), 7.53-7.27 (m, 22 H), 7.21

(bs, 1 H), 7.12 (bs, 1 H), 6.25 (bs, 1 H, -N***H***C=O from linker), 5.97 (d, *J* =8.5 Hz, 1 H), 5.86-5.57 (m, 4 H), 5.44-5.40 (m, 3 H), 4.94 (d, *J* =5.5 Hz, 1 H, **CH-1**), 4.79 (bs, 2 H, **AH-1** and **BH-1**), 4.60-4.55 (m, 2 H), 4.39-4.22 (m, 4 H), 4.07-3.98 (m, 6 H), 3.85-3.59 (m, 7 H), 3.17-3.14 (m, 3 H, -C***H***$_2$NH=O and -C***H***$_2$SAc), 2.50 (bs, 2 H, -NHC=OC***H***$_2$-), 2.25 (s, 3 H, **-SAc**), 1.79 (bs, 1 H, -OCH$_2$C***H***$_2$CH$_2$NH-), 1.68 (bs, 1 H, -OCH$_2$C***H***$_2$CH$_2$NH-). ^{13}C-NMR (125 MHz, CDCl$_3$): *δ*. 196.8, 171.1, 166.4, 166.2, 158.3 (q, *J* =37.5 Hz, C=O of TFA), 155.9, 154.6, 143.9, 143.6, 141.1, 133.7 (2 C), 133.5, 133.4, 133.3, 129.9 (2 C), 129.8, 128.9, 128.8, 128.7, 128.6, 128.5, 128.4, 128.3, 127.6, 127.0, 125.1, 119.9, 119.2 (q, *J* =286.0 Hz, CF$_3$ of TFA), 101.9, 101.6, 100.7, 95.4, 74.6, 74.1, 73.6, 73.0, 72.8, 72.4, 70.4, 69.8, 68.1, 66.8, 61.3, 56.8, 56.4, 54.7, 47.0, 36.4, 36.0, 30.6, 29.4, 25.1. HRMS: $C_{88}H_{83}Cl_3F_3N_4O_{26}S$ [M + H]$^+$ calcd: 1805.4034, obsd: 1805.4103.

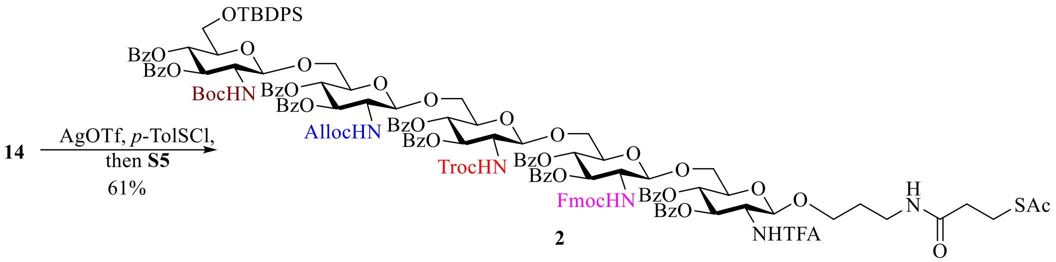

3-(3-(Acetylthio)propionylamino)propyl 3,4-di-*O*-benzoyl-6-*O*-*tert*-butyldiphenylsilyl-2-*tert*-butyloxycarbonylamino-2-deoxy-β-D-glucopyranosyl-(1→6)-2-allyloxycarbonylamino-3,4-di-*O*-benzoyl-2-deoxy-β-D-glucopyranosyl-(1→6)-2-deoxy-3,4-di-*O*-benzoyl-2-(2,2,2-trichloroethyloxycarbonylamino)-β-D-glucopyranosyl-(1→6)-3,4-di-*O*-benzoyl-2-fluorenylmethyloxycarbonylamino-2-deoxy-β-D-glucopyranosyl-(1→6)-3,4-di-*O*-benzoyl-2-deoxy-2-trifluoroacetamido-β-D-glucopyranoside (2)

Following the general procedure for pre-activation based glycosylation, compound **2** (toluene/acetone = 12/1, 61% yield) was prepared from donor **14** and acceptor **S5**. $[\alpha]_D^{20}$ = -4.0 (*c* 0.2, CH$_2$Cl$_2$); ^{1}H-NMR (500 MHz, CDCl$_3$): *δ*. 8.63 (d, *J* =9.5 Hz, 1 H, -N***H***C=O), 8.28-8.24 (m, 6 H), 8.11-8.05 (m, 6 H), 7.96-7.91 (m, 6 H), 7.87 (d, *J* =7.0 Hz, 2 H), 7.68-6.85 (m, 47 H), 6.78 (t, *J* =8.0 Hz, 1 H), 6.39 (bs, 1 H, -N***H***C=O), 6.05-5.99 (m, 2 H), 5.92-5.86 (m, 2 H), 5.73 (t, *J* =9.5 Hz, 1 H), 5.67 (t, *J* =9.5 Hz, 1 H), 5.44-5.38 (m, 3 H, -CH$_2$C***H***CH$_2$), 5.24-5.18 (m, 2 H, anomeric H), 5.14 (t, *J* =10.0 Hz, 1 H), 4.96-4.64 (m, 9 H, two anomeric H and -CH$_2$CHC***H***$_2$), 4.53-4.19 (m, 11 H, anomeric H), 4.13-4.04 (m, 3 H), 3.95 (t, *J* =9.5 Hz, 1 H), 3.87-3.84 (m, 2 H, anomeric H), 3.79-3,50 (m, 7 H), 3.37-3.23 (m, 4 H, -C***H***$_2$NH=O and -C***H***$_2$SAc), 2.65-2.57 (m, 2 H, -NHC=OC***H***$_2$-), 2.28 (s, 3 H, **-SAc**), 1.91-1.86 (m, 2 H, -OCH$_2$C***H***$_2$CH$_2$NH-), 1.15 (s, 9 H, -C(C***H***$_3$)$_3$), 1.06 (s, 9 H, -C(C***H***$_3$)$_3$). ^{13}C-NMR (125 MHz, CDCl$_3$): *δ*. 195.9, 171.0, 166.9, 166.8, 166.7, 166.6, 166.4, 166.1, 166.0, 165.7, 164.7, 164.5, 158.5 (q, *J* =37.5 Hz, C=O of TFA), 156.6, 156.2, 156.0, 155.5, 144.2, 143.5, 141.0, 140.6, 135.5, 135.3, 133.4 (2 C), 133.3, 133.2, 133.1 (2 C), 133.0, 131.9, 131.4, 130.5, 130.4, 130.3, 130.2, 130.0, 129.9 (2 C), 129.7, 129.6, 129.4, 129.2, 129.0 (2 C), 128.8 (2 C), 128.7 (2 C), 128.5, 128.4 (2 C), 128.2 (2 C), 128.1 (2 C), 127.7, 127.3, 127.2, 127.1, 126.7, 125.3, 125.1, 119.4, 119.3(2 C) (q, *J* =286.0 Hz, CF$_3$ of TFA), 117.6, 105.2, 103.0, 102.4, 99.7, 95.2, 80.7, 74.5, 74.1, 73.8, 73.4, 73.2, 73.1, 72.7, 72.5, 72.4, 72.3 (2 C), 71.9,

71.8, 70.8, 69.6, 68.0, 66.5, 66.2, 64.6, 57.5, 56.6, 56.3, 55.8, 54.9, 47.0, 36.8, 35.6, 30.5, 29.0, 27.9, 26.7, 25.1, 18.8. HRMS: $C_{153}H_{151}Cl_3F_3N_6O_{42}SSi$ [M + H]$^+$ calcd: 2965.8367, obsd: 2965.8389.

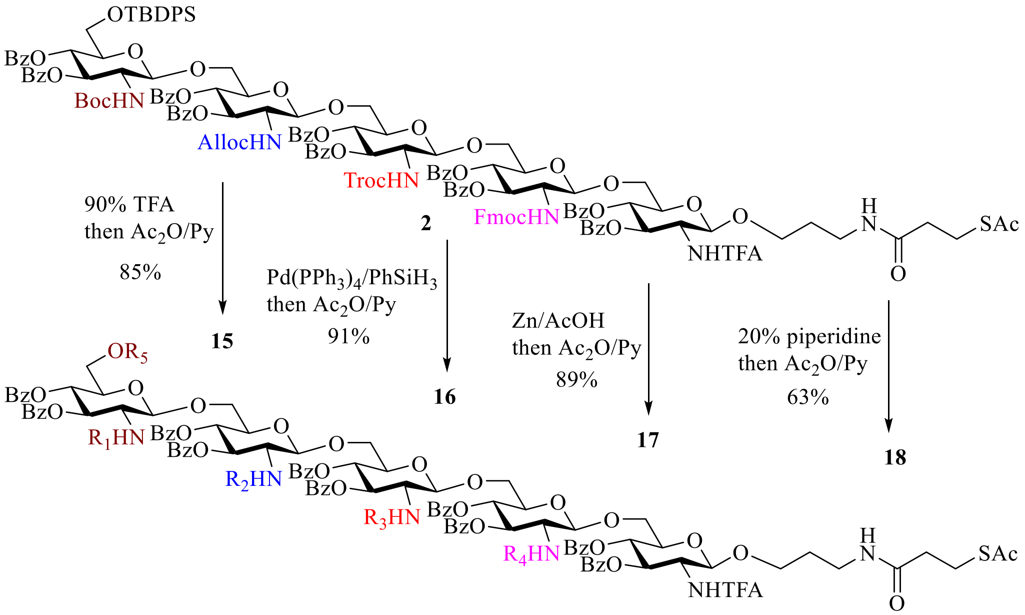

15: R_1 = Ac; R_2 = Alloc; R_3 = Troc; R_4 = Fmoc; R_5 = Ac
16: R_1 = Boc; R_2 = Ac; R_3 = Troc; R_4 = Fmoc; R_5 = TBDPS
17: R_1 = Boc; R_2 = Alloc; R_3 = Ac; R_4 = Fmoc; R_5 = TBDPS
18: R_1 = Boc; R_2 = Alloc; R_3 = Troc; R_4 = Ac; R_5 = TBDPS

3-(3-(Acetylthio)propionylamino)propyl 6-*O*-acetyl-2-acetamido-3,4-di-*O*-benzoyl-2-deoxy-β-D-glucopyranosyl-(1→6)-2-allyloxycarbonylamino-3,4-di-*O*-benzoyl-2-deoxy-β-D-glucopyranosyl-(1→6)- 3,4-di-*O*-benzoyl-2-deoxy-2-(2,2,2-trichloroethyloxycarbonylamino)-β-D-glucopyranosyl-(1→6)-3,4-di-*O*-benzoyl-2-deoxy-2-fluorenylmethyloxycarbonylamino-β-D-glucopyranosyl-(1→6)-3,4-di-*O*-benzoyl-2-deoxy-2-trifluoroacetamido-β-D-glucopyranoside (15)

Following the general procedure for Boc removal and acetylation, compound **15** was prepared in 85% yield for two steps. ^{1}H-NMR (500 MHz, $CDCl_3$): *δ.* 8.67 (m, 1 H), 8.27-7.89 (m, 20 H), 7.62-7.17 (m, 32 H), 7.11-7.03 (m, 3 H), 6.94-6.89 (m, 1 H), 6.82-6.78 (m, 1 H), 6.60-6.57 (m, 1 H, -N***H***C=O), 6.13-5.95 (m, 4 H), 5.84-5.30 (m, 12 H), 5.21-5.15 (m, 2 H), 4.97-4.75 (m, 8 H), 4.70-4.39 (m, 10 H), 4.32-4.08 (m, 11 H), 4.04-3.91 (m, 4 H), 3.83-3.55 (m, 9 H), 3.52-3.29 (m, 2 H), 3.24-3.21 (m, 2 H, -C***H***$_2$SAc), 2.67-2.56 (m, 2 H, -NHC=OC***H***$_2$-), 2.27 (s, 3 H, -S**Ac**), 1.96 (s, 3 H, -O**Ac**), 1.94-1.83 (m, 2 H, -OCH$_2$C***H***$_2$CH$_2$NH-), 1.72 (s, 3 H, -NH**Ac**). ^{13}C-NMR (125 MHz, $CDCl_3$): *δ.* 171.1, 170.6 (2 C), 170.4, 167.2, 167.0, 166.7, 166.6, 166.4, 166.1, 166.0, 165.8, 165.5, 165.0 (2 C), 156.6, 156.2, 156.0, 143.9, 143.6, 143.5, 141.0, 140.7, 133.7, 133.6 (2 C), 133.5, 133.3, 133.2, 133.1 (2 C), 132.1, 131.9, 131.8, 130.3 (2 C), 130.2, 130.1 (2 C), 130.0, 129.9 (2 C), 129.8 (2 C), 129.7, 129.6, 129.5, 129.4, 129.1, 128.9, 128.6, 128.5, 128.3, 128.2 (2 C), 128.1, 127.3, 127.1, 126.7, 125.2, 125.1, 119.5, 119.4, 117.8, 117.7, 117.6, 105.1, 103.7, 103.5, 102.2, 100.0, 95.5, 74.5, 74.0, 73.6, 73.4, 73.0, 72.7, 72.6, 72.5, 72.4, 72.3 (2 C), 72.1, 71.8, 69.6, 68.5,

66.6, 66.1 (2 C), 57.1, 56.7, 56.1, 56.0, 54.8, 46.9, 36.8, 35.7, 30.5, 29.1, 25.8, 25.7, 25.1, 20.9. HRMS: $C_{136}H_{129}Cl_3F_3N_6O_{42}S$ $[M + H]^+$ calcd: 2711.6876, obsd: 2711.6920.

3-(3-(Acetylthio)propionylamino)propyl 3,4-di-*O*-benzoyl-6-*O*-*tert*-butyldiphenylsilyl-2-*tert*-butyloxycarbonylamino-2-deoxy-β-D-glucopyranosyl-(1→6)-2-acetamido-3,4-di-*O*-benzoyl-2-deoxy-β-D-glucopyranosyl-(1→6)-3,4-di-*O*-benzoyl-2-deoxy-2-(2,2,2-trichloroethyloxycarbonylamino)-β-D-glucopyranosyl-(1→6)-3,4-di-*O*-benzoyl-2-deoxy-2-fluorenylmethyloxycarbonylamino-β-D-glucopyranosyl-(1→6)-3,4-di-*O*-benzoyl-2-deoxy-2-trifluoroacetamido-β-D-glucopyranoside (16)

Following the general procedure for Alloc removal and acetylation, compound **16** was prepared in 91% yield for two steps. ^{1}H-NMR (500 MHz, $CDCl_3$): *δ*. 8.64 (d, *J* =9.5 Hz, 1 H), 8.30-8.25 (m, 6 H), 8.12-8.05 (m, 6 H), 7.99-7.86 (m, 8 H), 7.73-7.70 (m, 1 H), 7.65-7.21 (m, 30 H), 7.16-7.01 (m, 8 H), 6.90-6.87 (m, 2 H), 6.83 (t, *J* =7.5 Hz, 1 H), 6.47-6.44 (m, 1 H, -N***H***C=O), 6.09-5.91 (m, 4 H), 5.80 (t, *J* =9.5 Hz, 1 H), 5.65 (t, *J* =9.5 Hz, 1 H), 5.46-5.37 (m, 2 H), 5.22 (d, *J* =9.0 Hz, 1 H), 5.16-5.12 (m, 1 H), 4.97-4.84 (m, 4 H), 4.76-4.64 (m, 3 H), 4.53-4.50 (m, 2 H), 4.42-4.28 (m, 5 H), 4.18-4.03 (m, 5 H), 3.97 (d, *J* =8.5 Hz, 1 H), 3.92-3.90 (m, 1 H), 3.88-3.84 (m, 1 H), 3.81-3.74 (m, 2 H), 3.71-3.50 (m, 5 H), 3.37-3.31 (m, 2 H), 3.28-3.25 (m, 2 H, -C$\boldsymbol{H}_2$SAc), 2.69-2.58 (m, 2 H, -NHC=OC$\boldsymbol{H}_2$-), 2.29 (s, 3 H, -S**Ac**), 1.91-1.86 (m, 2 H, -$OCH_2C\boldsymbol{H}_2CH_2NH$-),1.62 (s, 3 H, -NH**Ac**), 1.16 (s, 9 H, -C(C$\boldsymbol{H}_3)_3$), 1.07 (s, 9 H, -C(C$\boldsymbol{H}_3)_3$). ^{13}C-NMR (125 MHz, $CDCl_3$): *δ*. 195.8, 171.1, 170.2, 167.0, 166.9, 166.5, 166.4, 166.1 (2 C), 165.7, 164.9, 164.5, 156.8, 156.1, 155.5, 144.3, 143.8, 141.0, 140.8, 135.5, 135.3, 133.7, 133.5, 133.4, 133.2, 133.0, 132.9, 131.2, 130.6, 130.4 (2 C), 130.3, 130.2, 130.0 (2 C), 129.9, 129.7 (2 C), 129.4 (2 C), 129.3, 129.2, 129.0, 128.8, 128.7 (2 C), 128.5 (2 C), 128.4, 128.3 (2 C), 128.2, 127.5, 127.4, 127.2, 127.1, 126.6 (2 C), 125.5, 125.2, 119.4, 119.3, 105.2, 105.1, 103.0, 102.3, 99.6, 95.2, 80.8, 77.2, 74.7, 74.5, 74.2, 74.0, 73.4, 73.1, 72.8, 72.6, 72.4, 72.2, 71.9, 71.8, 70.7, 69.5, 69.2, 67.9, 66.5, 64.0, 57.5, 56.6, 55.9, 55.0, 54.3, 47.0, 36.7, 35.7, 30.5, 27.9, 26.7, 25.1, 22.9, 18.8. HRMS: $C_{151}H_{149}Cl_3F_3N_6O_{41}SSi$ $[M + H]^+$ calcd: 2923.8266, obsd: 2953.8305.

3-(3-(Acetylthio)propionylamino)propyl 3,4-di-*O*-benzoyl-6-*O*-*tert*-butyldiphenylsilyl-2-*tert*-butyloxycarbonylamino-2-deoxy-β-D-glucopyranosyl-(1→6)-2-allyloxycarbonylamino-3,4-di-*O*-benzoyl-2-deoxy-β-D-glucopyranosyl-(1→6)-2-acetamido-3,4-di-*O*-benzoyl-2-deoxy-β-D-glucopyranosyl-(1→6)-3,4-di-*O*-benzoyl-2-deoxy-2-fluorenylmethyloxycarbonylamino-β-D-glucopyranosyl-(1→6)-3,4-di-*O*-benzoyl-2-deoxy-2-trifluoroacetamido-β-D-glucopyranoside (17)

Following the general procedure for Troc removal and acetylation, compound **17** was prepared in 89% yield for two steps. ^{1}H-NMR (500 MHz, $CDCl_3$): *δ*. 9.52 (d, *J* =9.5 Hz, 1 H, -N***H***C=O), 8.28-7.85 (m, 20 H), 7.66-6.98 (m, 44 H), 6.93-6.90 (m, 1 H), 6.87-6.84 (m, 2 H), 6.76-6.73 (m, 1 H), 6.43-6.41 (m, 1 H, -N***H***C=O), 6.11 (t, *J* =10.0 Hz, 1 H), 5.96-5.84 (m, 3 H), 5.76-5.66 (m, 2 H), 5.45-5.37 (m, 3 H, -$CH_2C\boldsymbol{H}CH_2$), 5.27-5.19 (m, 2 H, anomeric H), 5.13-5.09 (m, 1 H), 4.95-4.61 (m, 9 H, two anomeric H and -$CH_2CHC\boldsymbol{H}_2$), 4.51-4.44 (m, 2 H), 4.36-4.03 (m, 10 H, anomeric H),

3.95-3.90 (m, 1 H), 3.87-3.83 (m, 1 H), 3.80-3.75 (m, 2 H), 3.60-3.50 (m, 5 H), 3.40-3.31 (m, 3 H), 3.25-3.22 (m, 2 H, -C***H***$_2$SAc), 2.62-2.58 (m, 2 H, -NHC=OC***H***$_2$-), 2.28 (s, 3 H, -S**Ac**), 1.91-1.86 (m, 2 H, -OCH$_2$C***H***$_2$CH$_2$NH-), 1.75 (s, 3 H, -NH**Ac**), 1.17 (s, 9 H, -C(C***H***$_3$)$_3$), 1.06 (s, 9 H, -C(C***H***$_3$)$_3$). ^{13}C-NMR (125 MHz, CDCl$_3$): *δ*. 195.9, 171.0, 170.7, 166.7 (2 C), 166.6, 166.4, 166.2, 166.0, 165.8, 164.9, 164.5, 158.7, 158.4, 156.6, 156.2, 155.6, 144.2, 143.5, 141.0, 140.6, 135.5, 135.3, 133.5, 133.4 (2 C), 133.3, 133.2 (2 C), 133.1, 132.9 (2 C), 131.9, 131.4, 130.5, 130.4, 130.3, 130.2, 130.1, 130.0, 129.9, 129.7, 129.6, 129.5, 129.2 (2 C), 129.0 (2 C), 128.9, 128.8 (2 C), 128.7, 128.5 (2 C), 128.3, 128.2, 127.7, 127.3, 127.2, 127.0, 126.7, 125.3, 125.1, 119.4, 119.3, 117.5, 105.3, 105.2, 103.3, 102.4, 99.7, 80.4, 77.5, 76.8, 74.5, 73.6, 73.1, 73.0, 72.8, 72.7, 72.5, 72.4, 72.3, 72.2, 72.1, 71.7, 70.8, 69.4, 67.9, 66.5, 66.1, 64.5, 56.6, 56.2, 56.0 (2 C), 54.9, 54.5, 53.8, 47.0, 36.9, 35.6, 30.5, 29.3, 28.8, 28.2, 27.8, 26.5, 25.1, 22.8, 18.8. HRMS: $C_{152}H_{152}F_3N_6O_{41}SSi$ $[M + H]^+$ calcd: 2833.9430, obsd: 2833.9499.

3-(3-(Acetylthio)propionylamino)propyl 3,4-di-*O*-benzoyl-6-*O*-*tert*-butyldiphenylsilyl-2-*tert*-butyloxycarbonylamino-2-deoxy-β-D-glucopyranosyl-(1→6)-2-allyloxycarbonylamino-3,4-di-*O*-benzoyl-2-deoxy-β-D-glucopyranosyl-(1→6)-3,4-di-*O*-benzoyl-2-deoxy-2-(2,2,2-trichloroethyloxycarbonylamino)-β-D-glucopyranosyl-(1→6)-2-acetamido-3,4-di-*O*-benzoyl-2-deoxy-β-D-glucopyranosyl-(1→6)-3,4-di-*O*-benzoyl-2-deoxy-2-trifluoroacetamido-β-D-glucopyranoside (18)

Following the general procedure for Fmoc removal and procedure for acetylation, compound **18** was prepared in 63% yield for two steps. ^{1}H-NMR (500 MHz, CDCl$_3$): *δ*. 8.58 (d, *J* =9.5 Hz, 1 H, -N***H***C=O), 8.26-8.20 (m, 6 H), 8.16 (d, *J* =8.0 Hz, 2 H), 8.09 (d, *J* =8.0 Hz, 2 H), 8.03 (d, *J* =7.5 Hz, 2 H), 7.96-7.92 (m, 6 H), 7.87 (d, *J* =7.5 Hz, 2 H), 7.68-7.55 (m, 6 H), 7.51-7.11 (m, 28 H), 7.05-7.02 (m, 3 H), 6.97-6.94 (m, 1 H), 6.87-6.84 (m, 2 H), 6.42-6.39 (m, 1 H, -N***H***C=O), 6.03-5.96 (m, 2 H), 5.90 (t, *J* =9.5 Hz, 1 H), 5.78-5.67 (m, 2 H), 5.64-5.60 (m, 1 H), 5.54-5.46 (m, 1 H, -CH$_2$C***H***CH$_2$), 5.41-5.34 (m, 2 H), 5.18-5.09 (m, 3 H, anomeric H), 4.97-4.74 (m, 6 H, two anomeric H and -CH$_2$CHC***H***$_2$), 4.68-4.56 (m, 3 H), 4.51-4.41 (m, 2 H), 4.35-4.04 (m, 10 H, anomeric H), 3.85-3.83 (m, 1 H), 3.76-3.63 (m, 3 H, anomeric H), 3.58-3.49 (m, 3 H), 3.35-3.23 (m, 3 H, -C***H***$_2$NH=O and -C***H***$_2$SAc), 2.68-2.56 (m, 2 H, -NHC=OC***H***$_2$-), 2.33 (s, 3 H, -C***H***$_2$SAc), 1.90-1.83 (m, 2 H, -OCH$_2$C***H***$_2$CH$_2$NH-), 1.71 (s, 3 H, -NH**Ac**), 1.12 (s, 9 H, -C(C***H***$_3$)$_3$), 1.06 (s, 9 H, -C(C***H***$_3$)$_3$). ^{13}C-NMR (125 MHz, CDCl$_3$): *δ*. 195.9, 171.1, 170.5, 166.9, 166.7 (2 C), 166.5 (2 C), 166.1, 166.0, 165.7, 164.9, 164.5, 156.2, 156.0, 135.5, 135.3, 133.5 (2 C), 133.4, 133.2, 133.1 (2 C), 133.0, 131.8, 131.3, 130.4 (2 C), 130.3 (3 C), 130.0, 129.9, 129.8 (2 C), 129.7 (2 C), 129.6, 129.4, 129.2, 129.0 (4 C), 128.8, 128.7, 128.6, 128.5, 128.4 (2 C), 128.3 (2 C), 128.2, 128.1, 127.6, 127.3, 117.1 (2 C), 105.0, 104.7, 102.9, 102.2, 99.5, 95.2, 80.8, 74.5, 74.1, 73.9, 73.4, 73.2, 73.0, 72.7, 72.4 (2 C), 72.2, 72.1, 72.0, 71.8, 71.7, 70.7, 69.6, 67.8, 65.9, 64.6, 57.4, 56.4, 55.8, 54.9, 54.0, 36.7, 35.7, 30.6, 29.1, 27.9, 26.7, 25.1, 22.7, 18.8. HRMS: $C_{140}H_{143}Cl_3F_3N_6O_{41}SSi$ $[M + H]^+$ calcd: 2785.7791, obsd: 2785.7714.

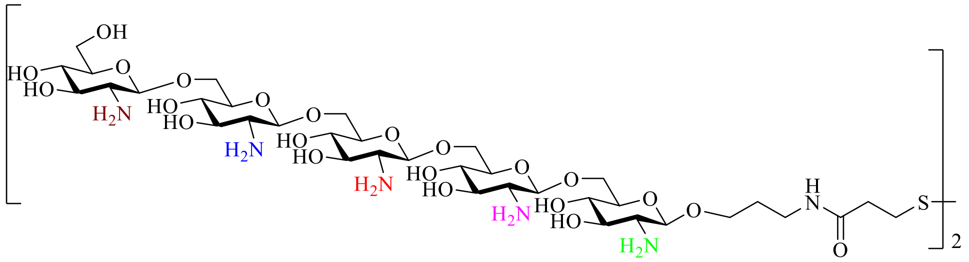

PNAG0 (00000): Compound **2** was subjected to Boc, Alloc, Troc, Fmoc, Bz and TFA removals according to the general procedures to afford **PNAG0** (10 mg, 43% yield for 5 steps) $[\alpha]_D^{20}$ = -5.3 (*c* 0.4, MeOH). ^{1}H-NMR (500 MHz, D_2O): *δ.* 4.67-4.62 (m, 4 H, anomeric H, obtained from HSQC NMR), 4.59 (d, *J* =8.5 Hz, 1 H, anomeric H), 4.14-4.08 (m, 4 H), 3.82-3.72 (m, 6 H), 3.63-3.51 (m, 10 H), 3.44-3.23 (m, 7 H), 3.21-3.16 (m, 1 H, -$OCH_2CH_2C\boldsymbol{H}_2NH$-), 3.10-3.04 (m, 1 H, -$OCH_2CH_2C\boldsymbol{H}_2NH$-), 2.97-2.88 (m, 5 H, -C***H***-NH_2), 2.80 (t, *J* =7.0 Hz, 2 H, -$C\boldsymbol{H}_2S$-), 2.52 (t, *J* =7.0 Hz, 2 H, -NHC=$OC\boldsymbol{H}_2$-), 1.70-1.64 (m, 2 H, -$OCH_2C\boldsymbol{H}_2CH_2NH$-). ^{13}C-NMR (125 MHz, D_2O): *δ.* 99.1 (4 C), 98.8, 76.1, 74.6, 71.6, 69.5, 68.3, 68.1, 67.5, 60.3, 60.1, 55.4, 35.9, 34.7, 33.0, 28.3 (obtained from HSQC NMR). HRMS: $C_{72}H_{136}N_{12}O_{44}S_2$ $[M + 2 H]^{2+}$ calcd: 968.4102, obsd: 968.4108.

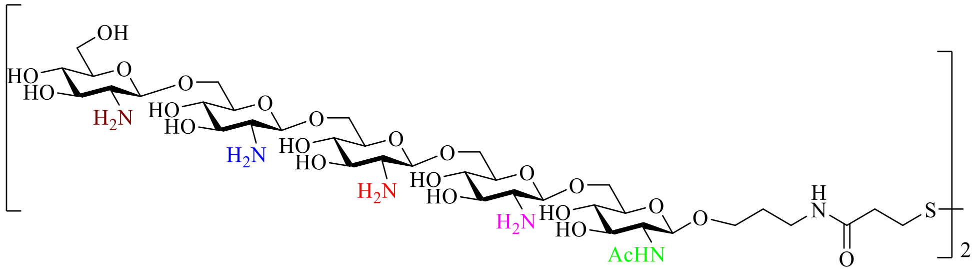

PNAG1 (00001): Compound **1** was subjected to Boc, Alloc, Troc, Fmoc, Bz removals according to the general procedures to afford **PNAG1** (3.5 mg, 41% yield for 5 steps). $[\alpha]_D^{20}$ = -4.4 (*c* 0.3, MeOH). ^{1}H-NMR (500 MHz, D_2O): *δ.* 4.67-4.63 (m, 4 H, anomeric H, obtained from HSQC NMR), 4.37 (d, *J* =8.5 Hz, 1 H, anomeric H), 4.14-4.08 (m, 4 H), 3.83-3.71 (m, 6 H), 3.63-3.30 (m, 18 H), 3.09-2.91 (m, 6 H, -$OCH_2CH_2C\boldsymbol{H}_2NH$- and -C***H***-$NH_2$), 2.80 (t, *J* =6.5 Hz, 2 H, -$C\boldsymbol{H}_2S$-), 2.51 (t, *J* =6.5 Hz, 2 H, -NHC=$OC\boldsymbol{H}_2$-), 1.88 (s, 3 H, -NH**Ac**), 1.62-1.57 (m, 2H, -$OCH_2C\boldsymbol{H}_2CH_2NH$-). ^{13}C-NMR (125 MHz, D_2O): *δ.* 101.3, 99.2 (4 C), 76.1, 74.6, 74.4, 73.4, 71.6, 69.7, 69.5, 68.3, 67.5, 60.3, 55.4, 36.3, 34.9, 33.2, 28.3, 22.0 (obtained from HSQC NMR). HRMS: $C_{76}H_{140}N_{12}O_{46}S_2$ $[M + 2 H]^{2+}$ calcd: 1010.4208, obsd: 1010.4211.

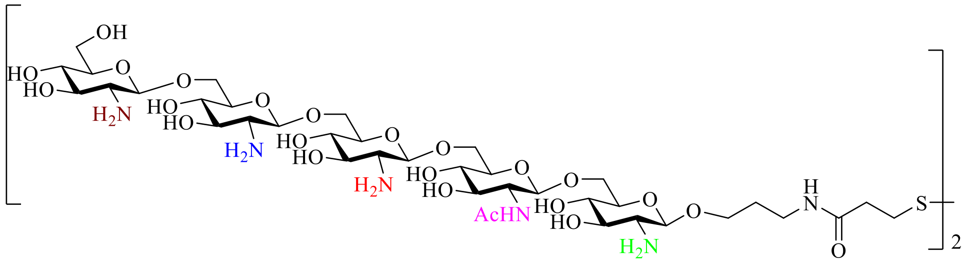

PNAG2 (00010): Compound **2** was subjected to Fmoc removal followed by acetylation, removal of Boc, Alloc, Troc, Bz and TFA according to the general procedures to afford **PNAG2** (2.8 mg, 53% yield for 6 steps). $[\alpha]_D^{20}$ = -3.6 (*c* 0.3, MeOH). ^{1}H-NMR (500 MHz, D_2O): *δ*. 4.66-4.62 (m, 3 H, anomeric H obtained from HSQC NMR), 4.54 (d, *J* =8.0 Hz, 1 H, anomeric H), 4.39 (d, *J* =8.0 Hz, 1 H, anomeric H), 4.11-4.04 (m, 4 H), 3.81-3.76 (m, 5 H), 3.61-3.17 (m, 20 H), 3.09-3.03 (m, 1 H, $-OCH_2CH_2C\boldsymbol{H}_2NH-$), 2.96-2.90 (m, 3 H, -C***H***-NH_2), 2.86 (t, *J* =9.5 Hz, 1 H, -C***H***-NH_2), 2.79 (bs, 2H, $-C\boldsymbol{H}_2S-$), 2.52 (t, *J* =6.0 Hz, 2 H, $-NHC{=}OC\boldsymbol{H}_2-$), 1.87 (s, 3 H, -NH**Ac**), 1.69-1.64 (m, 2H, $-OCH_2C\boldsymbol{H}_2CH_2NH-$). ^{13}C-NMR (125 MHz, D_2O): *δ*. 101.5, 99.1 (2 C), 99.0, 98.6, 76.1, 74.6, 74.4, 73.8, 71.8, 69.7, 69.5, 68.3, 68.1, 60.3, 55.4, 34.9, 33.0, 28.3, 22.2 (obtained from HSQC NMR). HRMS: $C_{76}H_{140}N_{12}O_{46}S_2$ $[M + 2 H]^{2+}$ calcd: 1010.4208, obsd: 1010.4200.

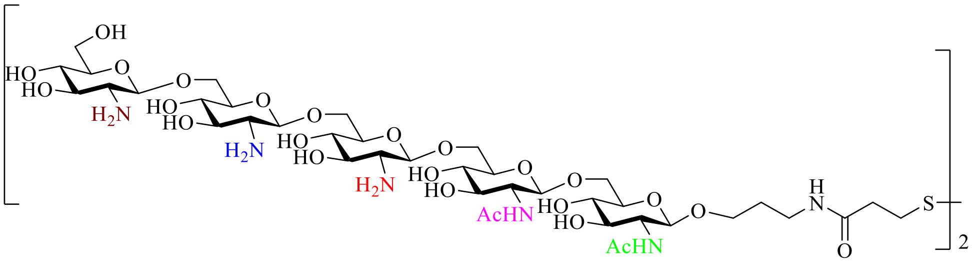

PNAG3 (00011): Compound **1** was subjected to Fmoc removal followed by acetylation, removal of Boc, Alloc, Troc and Bz according to the general procedures to afford **PNAG3** (1.1 mg, 37% yield for 6 steps). $[\alpha]_D^{20}$ = -2.6 (*c* 0.3, MeOH). ^{1}H-NMR (500 MHz, D_2O): *δ*. 4.67-4.62 (m, 3 H, anomeric H obtained from HSQC NMR), 4.42 (d, *J* =8.5 Hz, 1 H, anomeric H), 4.32 (d, *J* =8.5 Hz, 1 H, anomeric H), 4.13-4.00 (m, 4 H), 3.83-3.63 (m, 5 H), 3.63-3.21 (m, 16 H), 3.14-3.08 (m, 1 H), 3.04-2.91 (m, 4 H, $-OCH_2CH_2C\boldsymbol{H}_2NH-$ and -C***H***-NH_2), 2.81 (m, 1 H, $-C\boldsymbol{H}_2S-$), 2.64-2.59 (m, 1 H, $-C\boldsymbol{H}_2S-$), 2.53 (t, *J* =6.5 Hz, 2 H, $-NHC{=}OC\boldsymbol{H}_2-$), 1.88 (s, 6 H, -NH**Ac**), 1.63-1.58 (m, 2 H, -$OCH_2C\boldsymbol{H}_2CH_2NH-$). ^{13}C-NMR (125 MHz, D_2O): *δ*. 101.5, 100.9, 99.1 (2 C), 99.0, 76.1, 74.6, 74.2, 73.8, 71.8, 71.6, 69.9, 69.7, 69.5, 68.7, 68.5, 68.3, 67.5, 60.3, 60.1, 55.4, 55.0, 36.1, 28.1, 22.0 (obtained from HSQC NMR). HRMS: $C_{80}H_{144}N_{12}O_{48}S_2$ $[M + 2 H]^{2+}$ calcd: 1052.4313, obsd: 1052.4316.

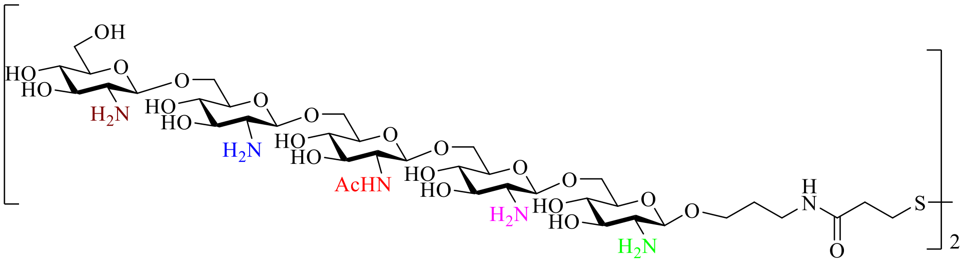

PNAG4 (00100): Compound **2** was subjected to Troc removal followed by acetylation, removal of Boc, Alloc, Fmoc, Bz and TFA according to the general procedures to afford **PNAG4** (2.0 mg, 48% yield for 6 steps). $[\alpha]_D^{20}$ = -1.2 (*c* 0.2, MeOH). ^{1}H-NMR (500 MHz, D_2O): *δ*. 4.64-4.62 (m, 2 H, anomeric H obtained from HSQC NMR), 4.60-4.57 (m, 2 H, anomeric H), 4.39 (d, *J* =8.5 Hz, 1 H, anomeric H), 4.11-4.04 (m, 4 H), 3.82-3.72 (m, 5 H), 3.62-3.30 (m, 18 H), 3.27 (t, *J* =9.0

Hz, 1 H), 3.20-3.16 (m, 1 H, -OCH$_2$CH$_2$C***H***$_2$NH-), 3.10-3.04 (m, 1 H, -OCH$_2$CH$_2$C***H***$_2$NH-), 2.96-2.87 (m, 4 H, -C***H***-NH$_2$), 2.80 (t, *J* =7.0 Hz, 2 H, -C***H***$_2$S-), 2.52 (t, *J* =7.0 Hz, 2 H, -NHC=OC***H***$_2$-), 1.89 (s, 3 H, -NH**Ac**), 1.69-1.64 (m, 2H, -OCH$_2$C***H***$_2$CH$_2$NH-). ^{13}C-NMR (125 MHz, D$_2$O): δ. 101.7, 99.2 (2 C), 99.0 (2 C), 76.1, 74.8 (2 C), 74.4, 74.2, 71.8, 69.7, 69.5 (2 C), 68.3, 68.1, 67.9, 67.7, 60.3, 55.4, 34.9, 33.0, 28.5, 24.8, 22.2 (obtained from HSQC NMR). HRMS: $C_{76}H_{140}N_{12}O_{46}S_2$ [M + 2 H]$^{2+}$ calcd: 1010.4208, obsd: 1010.4198.

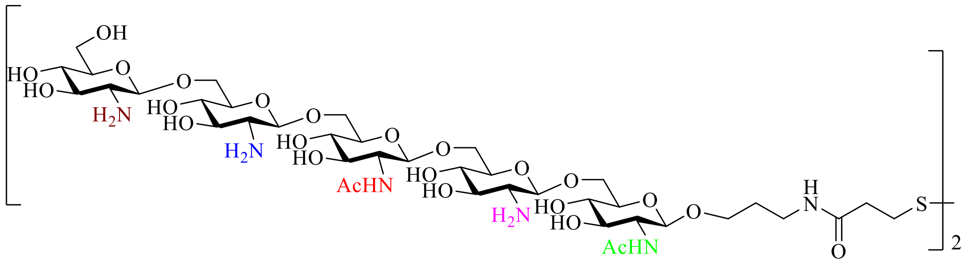

PNAG5 (00101): Compound **1** was subjected to Troc removal followed by acetylation, removal of Boc, Alloc, Fmoc and Bz according to the general procedures to afford **PNAG5** (3.1 mg, 53% yield for 6 steps). $[\alpha]_D^{20}$ = -4.5 (*c* 0.3, MeOH). ^{1}H-NMR (500 MHz, D$_2$O): δ. 4.66-4.59 (m, 3 H, anomeric H obtained from HSQC NMR), 4.39 (d, *J* =8.0 Hz, 1 H, anomeric H), 4.36 (d, *J* =8.5 Hz, 1 H, anomeric H), 4.10-4.03 (m, 4 H), 3.80-3.68 (m, 5 H), 3.61-3.24 (m, 20 H), 3.11-3.07 (m, 1 H), 3.02-2.87 (m, 5 H, -OCH$_2$CH$_2$C***H***$_2$NH- and -C***H***-NH$_2$), 2.62-2.56 (m, 2 H, -C***H***$_2$S-), 2.49 (t, *J* =7.0 Hz, 2 H, -NHC=OC***H***$_2$-), 1.88 (s, 3 H, -NH**Ac**), 1.87 (s, 3 H, -NH**Ac**), 1.61-1.56 (m, 2 H, -OCH$_2$C***H***$_2$CH$_2$NH-). ^{13}C-NMR (125 MHz, D$_2$O): δ. 101.4, 101.3, 99.0 (2 C), 98.9, 76.1, 74.6, 74.4, 73.4, 71.8, 69.5 (2 C), 68.5, 68.1, 67.7, 60.1, 55.4 (2 C), 55.2, 46.6, 36.5, 36.3, 31.0, 29.1, 28.3, 22.2 (obtained from HSQC NMR). HRMS: $C_{80}H_{144}N_{12}O_{48}S_2$ [M + 2 H]$^{2+}$ calcd: 1052.4313, obsd: 1052.4304.

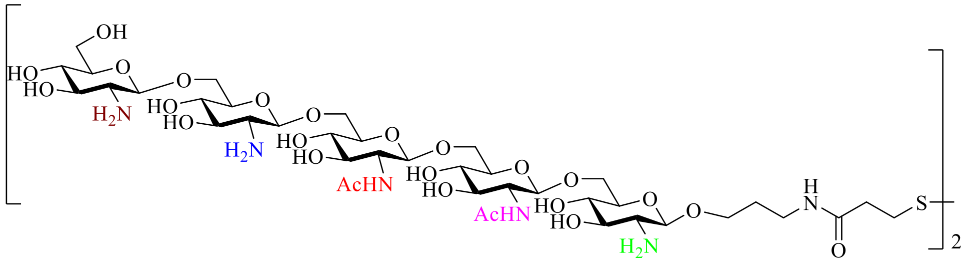

PNAG6 (00110): Compound **2** was subjected to Troc and Fmoc removals followed by acetylation, removal of Boc, Alloc, Bz and TFA according to the general procedures to afford **PNAG6** (2.3 mg, 51% yield for 6 steps). $[\alpha]_D^{20}$ = -2.6 (*c* 0.2, MeOH). ^{1}H-NMR (500 MHz, D$_2$O): δ. 4.67-4.62 (m, 2H, anomeric H obtained from HSQC NMR), 4.54 (d, *J* =9.0 Hz, 1 H, anomeric H), 4.41 (d, *J* =8.5 Hz, 1 H, anomeric H), 4.33 (d, *J* =9.0 Hz, 1 H, anomeric H), 4.11-3.99 (m, 4 H), 3.81-3.74 (m, 4 H), 3.62-3.17 (m, 22 H), 3.09-2.78 (m, 6 H, -OCH$_2$CH$_2$C***H***$_2$NH-, -C***H***-NH$_2$ and -C***H***$_2$S-), 2.52-2.48 (m, 2 H, -NHC=OC***H***$_2$-), 1.88 (s, 3 H, -NH**Ac**), 1.87 (s, 3 H, -NH**Ac**), 1.69-1.64 (m, 2 H, -OCH$_2$C***H***$_2$CH$_2$NH-). ^{13}C-NMR (125 MHz, D$_2$O): δ. 101.5 (2 C), 99.2 (2 C), 98.6, 76.1, 74.6, 74.3, 74.0, 71.8, 69.7 (2 C), 69.5 (2 C), 68.5, 68.3, 68.1, 67.3, 60.1, 55.4, 55.3, 36.1, 34.9, 33.2,

28.3, 22.2 (obtained from HSQC NMR). HRMS: $C_{80}H_{144}N_{12}O_{48}S_2$ $[M + 2 H]^{2+}$ calcd: 1052.4313, obsd: 1052.4304.

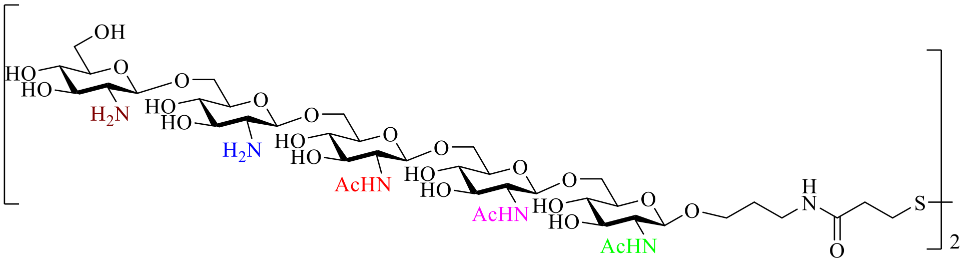

PNAG7 (00111): Compound **1** was subjected to Troc and Fmoc removals followed by acetylation, removal of Boc, Alloc and Bz according to the general procedures to afford **PNAG7** (2.8 mg, 44% yield for 6 steps). $[\alpha]_D^{20}$ = -4.5 (*c* 0.3, MeOH). ^{1}H-NMR (500 MHz, D_2O): *δ*. 4.67-4.62 (m, 2 H, anomeric H obtained from HSQC NMR), 4.42 (d, *J* =8.5 Hz, 1 H, anomeric H), 4.35 (d, *J* =8.0 Hz, 1 H, anomeric H), 4.31 (d, *J* =8.5 Hz, 1 H, anomeric H), 4.11-3.98 (m, 4 H), 3.81-3.70 (m, 5 H), 3.62-3.20 (m, 21 H), 3.13-3.07 (m, 1 H, -OCH$_2$CH$_2$C***H***$_2$NH-), 3.03-2.91 (m, 3 H, -C***H***-NH$_2$ and -OCH$_2$CH$_2$C***H***$_2$NH-), 2.80 (t, *J* =7.0 Hz, 2 H, -C***H***$_2$S-), 2.51 (t, *J* =6.5 Hz, 2 H, -NHC=OC***H***$_2$-), 1.89 (s, 3 H, -NH**Ac**), 1.87 (s, 6 H, -NH**Ac**), 1.62-1.57 (m, 2 H, -OCH$_2$C***H***$_2$CH$_2$NH-). ^{13}C-NMR (125 MHz, D_2O): *δ*. 101.4, 101.2, 100.9, 99.0, 98.8, 76.1, 73.8, 71.8, 69.7, 69.5, 68.5, 68.3, 60.3, 55.4 (2 C), 55.2, 36.3, 36.2, 34.9, 33.2, 28.3, 22.2 (obtained from HSQC NMR). HRMS: $C_{84}H_{148}N_{12}O_{50}S_2$ $[M + 2 H]^{2+}$ calcd: 1094.4419, obsd: 1094.4407.

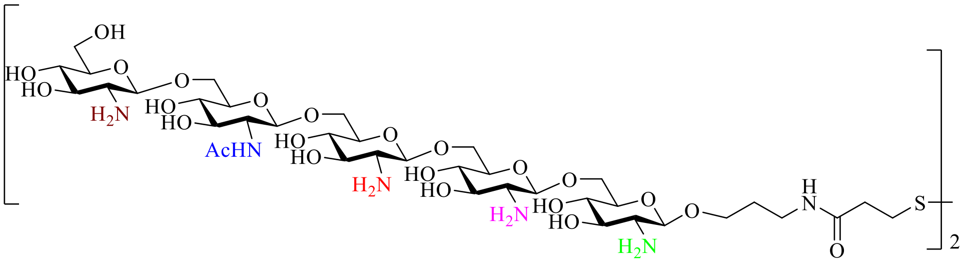

PNAG8 (01000): Compound **2** was subjected to Alloc removal followed by acetylation, removal of Boc, Troc, Fmoc, Bz and TFA according to the general procedures to afford **PNAG8** (3.1 mg, 40% yield for 6 steps). $[\alpha]_D^{20}$ = -2.4 (*c* 0.3, MeOH). ^{1}H-NMR (500 MHz, D_2O): *δ*. 4.65-4.57 (m, 4 H, anomeric H obtained from HSQC NMR), 4.38 (d, *J* =8.5 Hz, 1 H, anomeric H), 4.09-3.98 (m, 4 H), 3.78-3.71 (m, 5 H), 3.61-3.17 (m, 20 H), 3.08-3.04 (m, 1 H, -OCH$_2$CH$_2$C***H***$_2$NH-), 2.95-2.87 (m, 4 H, -C***H***-NH$_2$), 2.78 (bs, 2 H, -C***H***$_2$S-), 2.50 (bs, 2 H, -NHC=OC***H***$_2$-), 1.88 (s, 3 H, -NH**Ac**), 1.67 (bs, 2H, -OCH$_2$C***H***$_2$CH$_2$NH-). ^{13}C-NMR (125 MHz, D_2O): *δ*. 101.8, 98.9, 98.8, 76.1, 74.6, 74.4 (2 C), 73.8, 71.8, 69.7, 69.5 (2 C), 68.3, 68.1 (2 C), 67.7, 60.1, 55.4 (2 C), 34.7, 33.0, 28.3, 22.2 (obtained from HSQC NMR). HRMS: $C_{76}H_{140}N_{12}O_{46}S_2$ $[M + 2 H]^{2+}$ calcd: 1010.4208, obsd: 1010.4200.

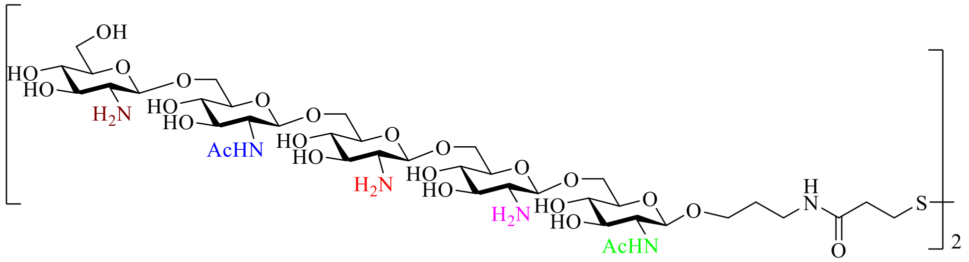

PNAG9 (01001): Compound **1** was subjected to Alloc removal followed by acetylation, removal of Boc, Troc, Fmoc and Bz according to the general procedures to afford **PNAG9** (2.9 mg, 41% yield for 6 steps). $[\alpha]_D^{20}$ = -4.4 (*c* 0.3, MeOH). ^{1}H-NMR (500 MHz, D_2O): *δ*. 4.69-4.63 (m, 3 H, anomeric H obtained from HSQC NMR), 4.42-4.39 (m, 2 H, anomeric H), 4.13-4.06 (m, 4 H), 3.83-3.74 (m, 5 H), 3.65-3.28 (m, 21 H), 3.15-3.09 (m, 1 H, -OCH$_2$CH$_2$C***H***$_2$NH-), 3.06-3.02 (m, 1 H, -OCH$_2$CH$_2$C***H***$_2$NH-), 3.00-2.91 (m, 3 H, -C***H***-NH$_2$), 2.83 (t, *J* =7.0 Hz, 2 H, -C***H***$_2$S-), 2.54 (t, *J* =6.5 Hz, 2 H, -NHC=OC***H***$_2$-), 1.92 (s, 3 H, -NH**Ac**), 1.91 (s, 3 H, -NH**Ac**), 1.66-1.60 (m, 2H, -OCH$_2$C***H***$_2$CH$_2$NH-). ^{13}C-NMR (125 MHz, D_2O): *δ*. 101.7, 101.3, 99.0 (3 C), 75.7, 74.6, 74.4, 73.6, 71.8, 69.7 (2 C), 69.5, 68.3 (2 C), 68.1, 60.7, 60.3, 55.4, 55.2, 36.3 (2 C), 34.7, 33.2, 28.3, 22.2 (obtained from HSQC NMR). HRMS: $C_{80}H_{144}N_{12}O_{48}S_2$ $[M + 2 H]^{2+}$ calcd: 1052.4313, obsd: 1052.4308.

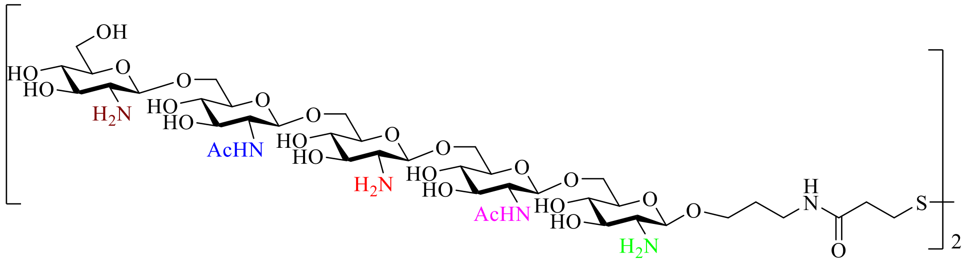

PNAG10 (01010): Compound **2** was subjected to Alloc and Fmoc removals followed by acetylation, removal of Boc, Troc, Bz and TFA according to the general procedures to afford **PNAG10** (3.2 mg, 53% yield for 6 steps). $[\alpha]_D^{20}$ = -5.0 (*c* 0.3, MeOH). ^{1}H-NMR (500 MHz, D_2O): *δ*. 4.64-4.62 (m, 1 H, anomeric H obtained from HSQC NMR), 4.61 (d, *J* =8.5 Hz, 1 H, anomeric H), 4.54 (d, *J* =8.5 Hz, 1 H, anomeric H), 4.39 (d, *J* =8.5 Hz, 2 H, anomeric H), 4.09-4.02 (m, 4 H), 3.79-3.70 (m, 4 H), 3.62-3.17 (m, 22 H), 3.09-3.04 (m, 1 H, -OCH$_2$CH$_2$C***H***$_2$NH-), 2.95-2.77 (m, 5 H, -C***H***-NH$_2$ and -C***H***$_2$S-), 2.52 (t, *J* =6.5 Hz, 2 H, -NHC=OC***H***$_2$-), 1.89 (s, 3 H, -NH**Ac**), 1.87 (s, 3 H, -NH**Ac**), 1.69-1.64 (m, 2 H, -OCH$_2$C***H***$_2$CH$_2$NH-). ^{13}C-NMR (125 MHz, D_2O): *δ*. 101.6 (2 C), 99.0 (2 C), 98.6, 76.1, 74.6, 74.5, 74.4, 73.6, 71.8 (2 C), 69.7 (2 C), 69.5, 69.4, 68.3, 68.1, 67.9, 67.5, 60.1, 55.6, 55.4, 55.2, 34.9, 33.0, 28.3, 22.2 (obtained from HSQC NMR). HRMS: $C_{80}H_{144}N_{12}O_{48}S_2$ $[M + 2 H]^{2+}$ calcd: 1052.4313, obsd: 1052.4318.

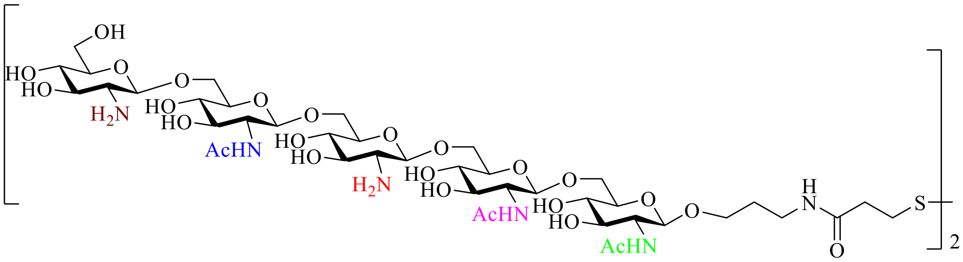

PNAG11 (01011): Compound **1** was subjected to Alloc and Fmoc removals followed by acetylation, removal of Boc, Troc and Bz according to the general procedures to afford **PNAG11** (2.8 mg, 50% yield for 6 steps). $[\alpha]_D^{20}$ = -3.7 (*c* 0.3, MeOH). ^{1}H-NMR (500 MHz, D_2O): *δ*. 4.65-4.61 (m, 2 H, anomeric H obtained from HSQC NMR), 4.42-4.39 (m, 2 H, anomeric H), 4.32 (d, *J* =8.5 Hz, 1 H, anomeric H), 4.11-4.02 (m, 4 H), 3.81-3.71 (m, 5 H), 3.62-3.20 (m, 22 H), 3.13-3.08 (m, 1 H, -OCH$_2$CH$_2$C***H***$_2$NH-), 3.04-2.99 (m, 1 H, -OCH$_2$CH$_2$C***H***$_2$NH-), 2.96-2.89 (m, 2 H, -C***H***-NH$_2$), 2.81 (t, *J* =7.0 Hz, 2 H, -C***H***$_2$S-), 2.52 (t, *J* =6.0 Hz, 2 H, -NHC=OC***H***$_2$-), 1.90 (s, 3 H, -NH**Ac**), 1.89 (s, 3 H, -NH**Ac**), 1.88 (s, 3 H, -NH**Ac**), 1.64-1.59 (m, 2 H, -OCH$_2$C***H***$_2$CH$_2$NH-). ^{13}C-NMR (125 MHz, D_2O): *δ*. 101.5, 101.4, 100.9, 98.9 (2 C), 76.1, 74.4, 74.1, 74.0, 73.5, 71.8, 69.7, 69.5, 69.4, 68.5, 68.3, 67.9, 60.1, 55.6, 55.5, 55.3, 36.3, 34.7, 33.2, 28.3, 22.0 (obtained from HSQC NMR). HRMS: $C_{84}H_{148}N_{12}O_{50}S_2$ $[M + 2 H]^{2+}$ calcd: 1094.4419, obsd: 1094.4407.

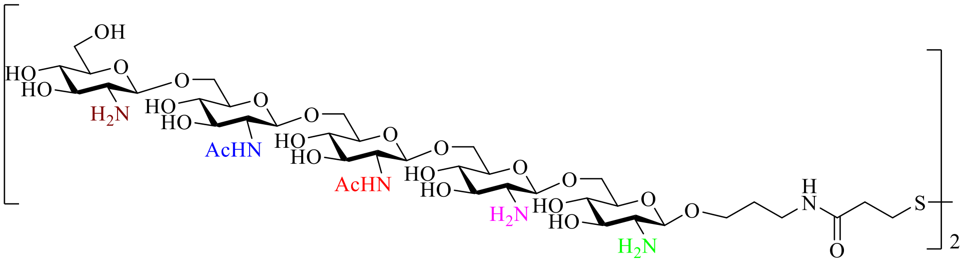

PNAG12 (01100): Compound **2** was subjected to Alloc and Troc removals followed by acetylation, removal of Boc, Fmoc, Bz and TFA according to the general procedures to afford **PNAG12** (2.7 mg, 49% yield for 6 steps). $[\alpha]_D^{20}$ = -1.4 (*c* 0.3, MeOH). ^{1}H-NMR (500 MHz, D_2O): *δ*. 4.64-4.56 (m, 3 H, anomeric H obtained from HSQC NMR), 4.39 (d, *J* =8.5 Hz, 1 H, anomeric H), 4.33 (d, *J* =8.5 Hz, 1 H, anomeric H), 4.09-3.98 (m, 4 H), 3.81-3.70 (m, 5 H), 3.60-3.47 (m, 11 H), 3.41-3.16 (m, 10 H), 3.09-3.04 (m, 1 H, -OCH$_2$CH$_2$C***H***$_2$NH-), 2.94-2.86 (m, 3 H, -C***H***-NH$_2$), 2.80 (t, *J* =6.5 Hz, 2 H, -C***H***$_2$S-), 2.52 (t, *J* =7.0 Hz, 2 H, -NHC=OC***H***$_2$-), 1.88 (s, 3 H, -NH**Ac**), 1.87 (s, 3 H, -NH**Ac**), 1.69-1.64 (m, 2 H, -OCH$_2$C***H***$_2$CH$_2$NH-). ^{13}C-NMR (125 MHz, D_2O): *δ*. 101.5 (2 C), 98.8, 98.7 (2 C), 75.9, 74.4, 74.2, 74.0, 73.6, 71.6, 69.7, 69.5, 69.3, 68.3, 68.1, 67.9, 67.7, 60.1, 36.1, 34.7, 33.2, 28.3, 22.2 (obtained from HSQC NMR). HRMS: $C_{80}H_{144}N_{12}O_{48}S_2$ $[M + 2 H]^{2+}$ calcd: 1052.4313, obsd: 1052.4304.

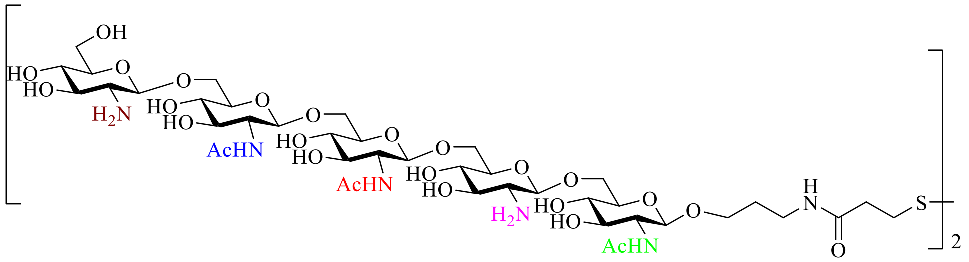

PNAG13 (01101): Compound **1** was subjected to Alloc and Troc removals followed by acetylation, removal of Boc, Fmoc and Bz according to the general procedures to afford **PNAG13** (2.1 mg, 48% yield for 6 steps). $[\alpha]_D^{20}$ = -4.7 (*c* 0.3, MeOH). ^{1}H-NMR (500 MHz, D_2O): *δ*. 4.66-4.60 (m, 2 H, anomeric H obtained from HSQC NMR), 4.41 (d, *J* =8.5 Hz, 1 H, anomeric H), 4.37-4.33 (m, 2 H, anomeric H), 4.11-4.00 (m, 4 H), 3.80-3.69 (m, 4 H), 3.63-3.22 (m, 22 H), 3.12-3.09 (m, 1 H, $-OCH_2CH_2C\boldsymbol{H}_2NH-$), 3.04-2.99 (m, 1 H, $-OCH_2CH_2C\boldsymbol{H}_2NH-$), 2.96-2.89 (m, 2 H, -C***H***-NH_2), 2.81 (t, *J* =6.5 Hz, 2 H, $-C\boldsymbol{H}_2S-$), 2.52 (t, *J* =6.5 Hz, 2 H, $-NHC{=}OC\boldsymbol{H}_2-$), 1.90 (s, 3 H, -NH**Ac**), 1.89 (s, 3 H, -NH**Ac**), 1.88 (s, 3 H, -NH**Ac**), 1.63-1.58 (m, 2 H, $-OCH_2C\boldsymbol{H}_2CH_2NH-$). ^{13}C-NMR (125 MHz, D_2O): *δ*. 101.6, 101.5 (2 C), 99.0, 98.9, 76.1, 74.5, 74.3, 74.0, 73.8, 73.7, 73.5, 71.8, 69.7, 69.6, 68.5, 68.1, 60.3, 55.4, 55.3, 36.3, 34.7, 33.2, 28.3, 22.2 (obtained from HSQC NMR). HRMS: $C_{84}H_{148}N_{12}O_{50}S_2$ $[M + 2 H]^{2+}$ calcd: 1094.4419, obsd: 1094.4402.

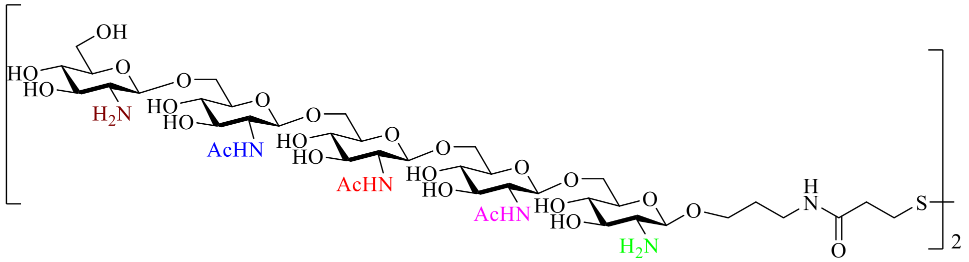

PNAG14 (01110): Compound **2** was subjected to Alloc, Troc and Fmoc removals followed by acetylation, removal of Boc, Bz and TFA according to the general procedures to afford **PNAG14** (3.2 mg, 44% yield for 6 steps). $[\alpha]_D^{20}$ = -5.0 (*c* 0.3, MeOH). ^{1}H-NMR (500 MHz, D_2O): *δ*. 4.64-4.62 (m, 1 H, anomeric H obtained from HSQC NMR), 4.54 (d, *J* =8.0 Hz, 1 H, anomeric H), 4.41 (d, *J* =8.5 Hz, 1 H, anomeric H), 4.35-4.32 (m, 2 H, anomeric H), 4.10-3.98 (m, 4 H), 3.78-3.73 (m, 4 H), 3.61-3.20 (m, 24 H), 3.09-3.04 (m, 1 H, $-OCH_2CH_2C\boldsymbol{H}_2NH-$), 2.95 (t, *J* =9.0 Hz, 1 H, -C***H***-NH_2), 2.87 (t, *J* =9.0 Hz, 1 H, -C***H***-NH_2), 2.81 (t, *J* =6.0 Hz, 2 H, $-C\boldsymbol{H}_2S-$), 2.52 (t, *J* =6.5 Hz, 2 H, $-NHC{=}OC\boldsymbol{H}_2-$), 1.89 (s, 3 H, -NH**Ac**), 1.88 (s, 3 H, -NH**Ac**), 1.87 (s, 3 H, -NH**Ac**), 1.69-1.64 (m, 2 H, $-OCH_2C\boldsymbol{H}_2CH_2NH-$). ^{13}C-NMR (125 MHz, D_2O): *δ*. 101.6, 101.5 (2 C), 99.0, 98.6, 76.1, 74.6, 74.4, 74.3, 74.0, 73.7, 71.9, 71.8, 69.9, 69.7, 69.6, 68.4, 68.1, 68.0, 67.5, 60.1, 55.4 (2 C), 55.2, 35.9, 34.7, 33.2, 28.3, 22.2 (obtained from HSQC NMR). HRMS: $C_{84}H_{148}N_{12}O_{50}S_2$ $[M + 2 H]^{2+}$ calcd: 1094.4419, obsd: 1094.4413.

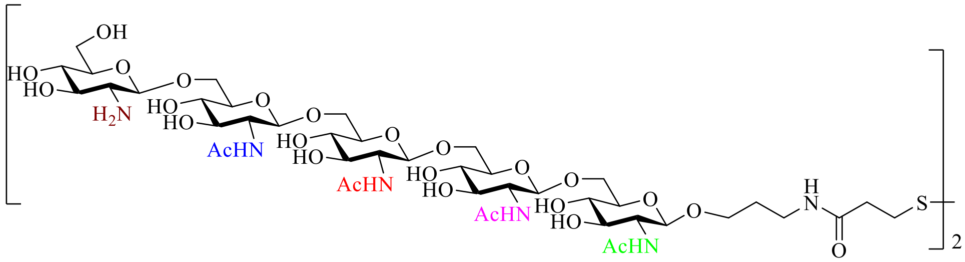

PNAG15 (01111): Compound **1** was subjected to Alloc, Troc and Fmoc removals followed by acetylation, removal of Boc and Bz according to the general procedures to afford **PNAG15** (2.2 mg, 39% yield for 6 steps). $[\alpha]_D^{20}$ = -3.1 (*c* 0.3, MeOH). ^{1}H-NMR (500 MHz, D_2O): *δ*. 4.65-4.63 (m, 1 H, anomeric H obtained from HSQC NMR), 4.43 (d, *J* =8.5 Hz, 1 H, anomeric H), 4.38-4.35 (m, 2 H, anomeric H), 4.30 (d, *J* =8.5 Hz, 1 H, anomeric H), 4.12 (d, *J* =10.5 Hz, 1 H), 4.05-3.99 (m, 3 H), 3.80-3.70 (m, 4 H), 3.62-3.48 (m, 10 H), 3.44-3.34 (m, 10 H), 3.29-3.21 (m, 3 H), 3.16-3.10 (m, 1 H, -$OCH_2CH_2C\boldsymbol{H}_2NH$-), 3.04-2.98 (m, 1 H, -$OCH_2CH_2C\boldsymbol{H}_2NH$-), 2.97 (dd, *J* =8.5, 10.5 Hz, 1 H, -C***H***-NH_2), 2.82 (t, *J* =6.5 Hz, 2 H, -$C\boldsymbol{H}_2S$-), 2.53 (t, *J* =7.0 Hz, 2 H, -NHC=$OC\boldsymbol{H}_2$-), 1.90 (s, 3 H, -NH**Ac**), 1.89 (s, 3 H, -NH**Ac**), 1.88 (s, 6 H, -NH**Ac**), 1.64-1.59 (m, 2 H, -$OCH_2C\boldsymbol{H}_2CH_2NH$-). ^{13}C-NMR (125 MHz, D_2O): *δ*. 101.5, 101.3 (2 C), 100.9, 100.0, 76.1, 74.5, 74.2, 74.0, 73.8, 71.8, 69.9 (2 C), 69.5, 69.3, 68.5, 68.3, 68.2, 67.5, 60.3, 55.6, 55.4, 55.2, 36.1, 34.7, 33.2, 28.1, 22.2 (obtained from HSQC NMR). HRMS: $C_{88}H_{152}N_{12}O_{52}S_2$ $[M + 2 H]^{2+}$ calcd: 1136.4525, obsd: 1136.4507.

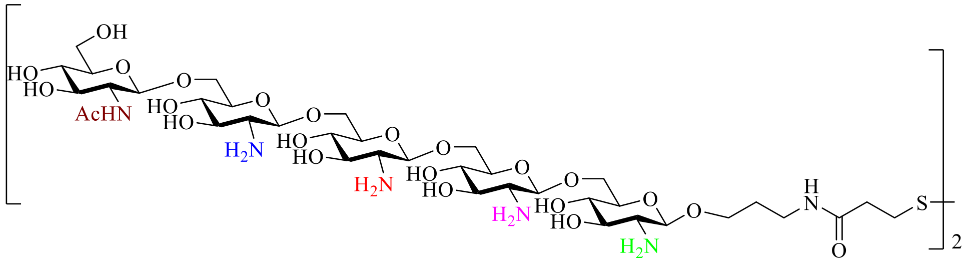

PNAG16 (10000): Compound **2** was subjected to Boc removal followed by acetylation, removal of Alloc, Troc, Fmoc, Bz and TFA according to the general procedures to afford **PNAG16** (4.0 mg, 41% yield for 6 steps). $[\alpha]_D^{20}$ = -2.5 (*c* 0.3, MeOH). ^{1}H-NMR (500 MHz, D_2O): *δ*. 4.65-4.63 (m, 2 H, anomeric H obtained from HSQC NMR), 4.61-4.57 (m, 2 H, anomeric H), 4.35 (d, *J* =8.5 Hz, 1 H, anomeric H), 4.11-4.01 (m, 4 H), 3.80-3.73 (m, 5 H), 3.65-3.46 (m, 12 H), 3.40-3.26 (m, 7 H), 3.21-3.17 (m, 1 H, -$OCH_2CH_2C\boldsymbol{H}_2NH$-), 3.09-3.05 (m, 1 H, -$OCH_2CH_2C\boldsymbol{H}_2NH$-), 2.95-2.87 (m, 4 H, -C***H***-NH_2), 2.79 (t, *J* =6.5 Hz, 1 H, -$C\boldsymbol{H}_2S$-), 2.51 (t, *J* =6.5 Hz, 2 H, -NHC=$OC\boldsymbol{H}_2$-), 1.89 (s, 3 H, -NH**Ac**), 1.67-1.65 (m, 2H, -$OCH_2C\boldsymbol{H}_2CH_2NH$-). ^{13}C-NMR (125 MHz, D_2O): *δ*. 101.7, 99.2 (2 C), 99.0, 98.8, 75.7, 74.6 (2 C), 74.4, 73.8, 71.8, 71.7, 69.7, 69.5, 68.3 (2 C), 68.1, 67.7, 60.5, 55.4, 55.3, 36.1, 34.7, 33.0, 28.3, 22.2 (obtained from HSQC NMR). HRMS: $C_{76}H_{140}N_{12}O_{46}S_2$ $[M + 2 H]^{2+}$ calcd: 1010.4208, obsd: 1010.4202.

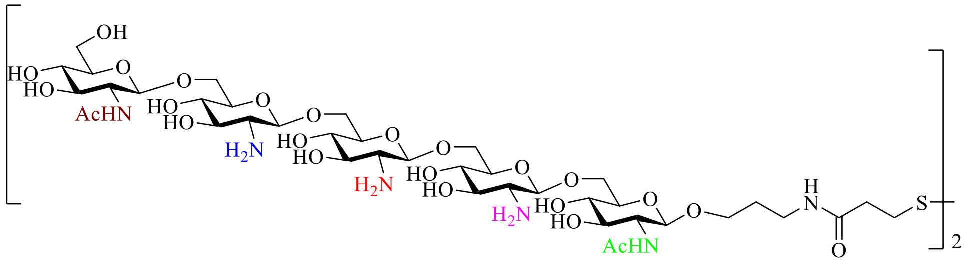

PNAG17 (10001): Compound **1** was subjected to Boc removal followed by acetylation, removal of Alloc, Troc, Fmoc and Bz according to the general procedures to afford **PNAG17** (2.1 mg, 48% yield for 6 steps). $[\alpha]_D^{20}$ = -2.7 (*c* 0.3, MeOH). ^{1}H-NMR (500 MHz, D_2O): *δ*. 4.68-4.64 (m, 2 H, anomeric H obtained from HSQC NMR), 4.62 (d, *J* =9.0 Hz, 1 H, anomeric H), 4.37-35 (m, 2 H, anomeric H), 4.12-4.02 (m, 4 H), 3.78-3.73 (m, 5 H), 3.65-3.26 (m, 21 H), 3.11-2.99 (m, 2 H, -$OCH_2CH_2C\boldsymbol{H}_2NH$-), 2.99-2.88 (m, 3 H, -C***H***-NH_2), 2.63-2.57 (m, 3 H, -C$\boldsymbol{H}_2$S- and -NHC=OC$\boldsymbol{H}_2$-), 1.89 (s, 3 H, -NH**Ac**), 1.88 (s, 3 H, -NH**Ac**), 1.62-1.57 (m, 2 H, -$OCH_2C\boldsymbol{H}_2CH_2NH$-). ^{13}C-NMR (125 MHz, D_2O): *δ*. 101.4, 101.2, 99.1, 99.0 (2 C), 75.7, 74.3 (2 C), 73.4, 71.6 (2 C), 69.7, 69.5 (2 C), 68.3 (2 C), 67.9, 60.5, 55.4, 55.2 (2 C), 36.1, 36.5, 29.1, 28.3, 22.0 (obtained from HSQC NMR). HRMS: $C_{80}H_{144}N_{12}O_{48}S_2$ $[M + 2 H]^{2+}$ calcd: 1052.4313, obsd: 1052.4302.

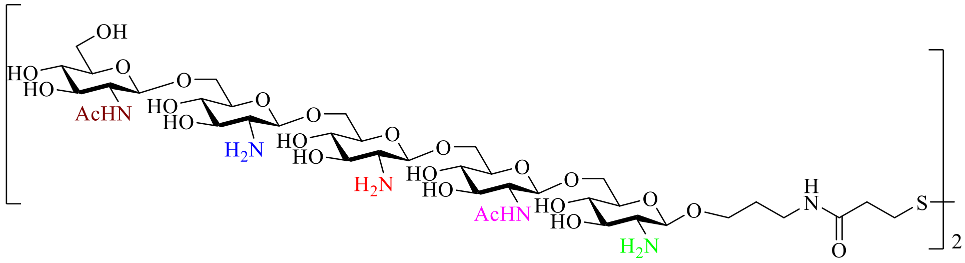

PNAG18 (10010): Compound **2** was subjected to Boc and Fmoc removals followed by acetylation, removal of Alloc, Troc, Bz and TFA according to the general procedures to afford **PNAG18** (2.5 mg, 47% yield for 6 steps). $[\alpha]_D^{20}$ = -2.7 (*c* 0.3, MeOH). ^{1}H-NMR (500 MHz, D_2O): *δ*. 4.67-4.60 (m, 2 H, anomeric H obtained from HSQC NMR), 4.55 (d, *J* =8.5 Hz, 1 H, anomeric H), 4.40-4.35 (m, 2 H, anomeric H), 4.11-4.02 (m, 4 H), 3.78-3.74 (m, 4 H), 3.65-3.17 (m, 22 H), 3.08-2.78 (m, 6 H, -$OCH_2CH_2C\boldsymbol{H}_2NH$-, -C***H***-$NH_2$ and -C$\boldsymbol{H}_2$S-), 2.53-2.48 (m, 2 H, -NHC=OC$\boldsymbol{H}_2$-), 1.89 (s, 3 H, -NH**Ac**), 1.88 (s, 3 H, -NH**Ac**), 1.69-1.64 (m, 2 H, -$OCH_2C\boldsymbol{H}_2CH_2NH$-). ^{13}C-NMR (125 MHz, D_2O): *δ*. 101.7, 101.5, 99.2, 99.0, 98.6, 75.7, 74.8, 74.6, 73.6 (2 C), 71.8, 71.7 (2 C), 69.7, 69.6, 68.2, 68.1, 67.3, 60.7, 55.7, 55.6, 55.2, 36.1, 32.8, 31.0, 28.3, 22.2 (obtained from HSQC NMR). HRMS: $C_{80}H_{144}N_{12}O_{48}S_2$ $[M + 2 H]^{2+}$ calcd: 1052.4313, obsd: 1052.4315.

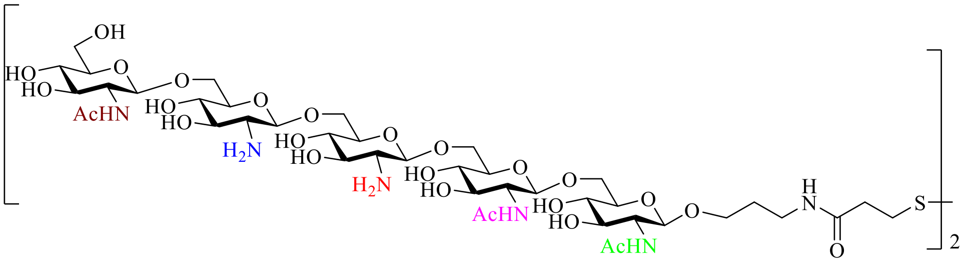

PNAG19 (10011): Compound **1** was subjected to Boc and Fmoc removals followed by acetylation, removal of Alloc, Troc and Bz according to the general procedures to afford **PNAG19** (2.2 mg, 51% yield for 6 steps). $[\alpha]_D^{20}$ = -3.9 (*c* 0.3, MeOH). ^{1}H-NMR (500 MHz, D_2O): *δ*. 4.67-4.65 (m, 1 H, anomeric H obtained from HSQC NMR), 4.61 (d, *J* =8.5 Hz, 1 H, anomeric H), 4.41 (d, *J* =8.0 Hz, 1 H, anomeric H), 4.36 (d, *J* =8.5 Hz, 1 H, anomeric H), 4.31 (d, *J* =8.5 Hz, 1 H, anomeric H), 4.11-4.02 (m, 4 H), 3.88-3.69 (m, 4 H), 3.64-3.20 (m, 24 H), 3.13-3.07 (m, 1 H, -$OCH_2CH_2C\boldsymbol{H}_2NH$-), 3.02-2.88 (m, 3 H, -$OCH_2CH_2C\boldsymbol{H}_2NH$- and -C***H***-$NH_2$), 2.62-2.55 (m, 3 H, -C***H***$_2$S- and -NHC=OC***H***$_2$-), 1.88 (s, 3 H, -NH**Ac**), 1.86 (ss, 6 H, -NH**Ac**), 1.62-1.57 (m, 2 H, -$OCH_2C\boldsymbol{H}_2CH_2NH$-). ^{13}C-NMR (125 MHz, D_2O): *δ*. 101.5 (2 C), 101.1, 99.2, 99.0, 75.7, 74.6, 74.4, 73.7, 73.4, 71.9, 71.8, 69.7 (2 C), 69.6, 68.5, 68.3, 68.1, 67.7, 60.5, 55.4, 55.2 (2 C), 36.7, 36.3, 29.1, 28.1, 22.0 (obtained from HSQC NMR). HRMS: $C_{84}H_{148}N_{12}O_{50}S_2$ $[M + 2 H]^{2+}$ calcd: 1094.4419, obsd: 1094.4404.

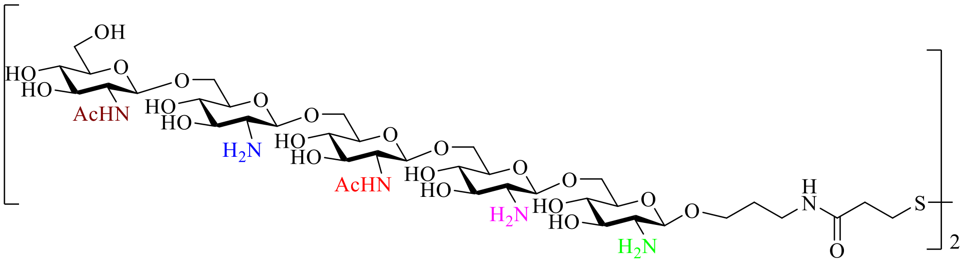

PNAG20 (10100): Compound **2** was subjected to Boc and Troc removals followed by acetylation, removal of Alloc, Fmoc, Bz and TFA according to the general procedures to afford **PNAG20** (3.3 mg, 53% yield for 6 steps). $[\alpha]_D^{20}$ = -2.5 (*c* 0.3, MeOH). ^{1}H-NMR (500 MHz, D_2O): *δ*. 4.62-4.57 (m, 3 H, anomeric H), 4.40- 4.35 (m, 2 H, anomeric H), 4.06-4.03 (m, 4 H), 3.80-3.70 (m, 5 H), 3.62-3.21 (m, 24 H), 3.06-2.99 (m, 2 H, -$OCH_2CH_2C\boldsymbol{H}_2NH$-), 2.92-2.86 (m, 3 H, -C***H***-NH_2), 2.81 (t, *J* =7.0 Hz, 2 H, -C***H***$_2$S-), 2.52-2.48 (m, 2 H, -NHC=OC***H***$_2$-), 1.89 (s, 6 H, -NH**Ac**), 1.70-1.65 (m, 2 H, -$OCH_2C\boldsymbol{H}_2CH_2NH$-). ^{13}C-NMR (125 MHz, D_2O): *δ*. 101.5, 101.2, 98.9, 98.8, 98.7, 75.5, 74.8, 74.2 (2 C), 73.6, 72.2, 71.6, 69.5, 69.3, 69.2, 68.3, 68.1, 67.5, 60.5, 55.4, 55.2, 46.8, 36.1, 31.2, 28.3, 22.4 (obtained from HSQC NMR). HRMS: $C_{80}H_{144}N_{12}O_{48}S_2$ $[M + 2 H]^{2+}$ calcd: 1052.4313, obsd: 1052.4298.

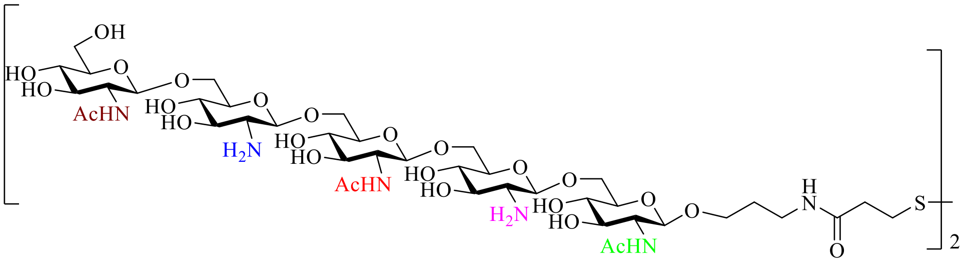

PNAG21 (10101): Compound **1** was subjected to Boc and Troc removals followed by acetylation, removal of Alloc, Fmoc and Bz according to the general procedures to afford **PNAG21** (2.8 mg, 51% yield for 6 steps). $[\alpha]_D^{20}$ = -4.0 (*c* 0.3, MeOH). ^{1}H-NMR (500 MHz, D_2O): *δ*. 4.67-4.65 (m, 2 H, anomeric H obtained from HSQC NMR), 4.40 (d, *J* =8.5 Hz, 1 H, anomeric H), 4.36 (d, *J* =8.5 Hz, 2 H, anomeric H), 4.06-3.99 (m, 4 H), 3.78-3.69 (m, 5 H), 3.64-3.34 (m, 18 H), 3.31-3.25 (m, 4 H), 3.11-3.08 (m, 1 H, $-OCH_2CH_2C\boldsymbol{H}_2NH-$), 3.04-3.00 (m, 1 H, $-OCH_2CH_2C\boldsymbol{H}_2NH-$), 2.93-2.88 (m, 2 H, $-C\boldsymbol{H}-NH_2$), 2.80 (t, *J* =6.5 Hz, 2 H, $-C\boldsymbol{H}_2S-$), 2.51 (t, *J* =6.5 Hz, 2 H, $-NHC{=}OC\boldsymbol{H}_2-$), 1.81 (s, 6 H, -NH**Ac**), 1.88 (s, 3 H, -NH**Ac**), 1.62-1.57 (m, 2 H, $-OCH_2C\boldsymbol{H}_2CH_2NH-$). ^{13}C-NMR (125 MHz, D_2O): *δ*. 101.7, 101.6 (2 C), 99.3, 99.2, 75.9, 74.8, 74.4, 73.8, 71.8, 69.8, 69.7, 68.3 (2 C), 68.1, 60.7, 55.6, 55.4, 36.3, 34.7, 33.2, 28.3, 22.2 (obtained from HSQC NMR). HRMS: $C_{84}H_{148}N_{12}O_{50}S_2$ $[M + 2 H]^{2+}$ calcd: 1094.4419, obsd: 1094.4408.

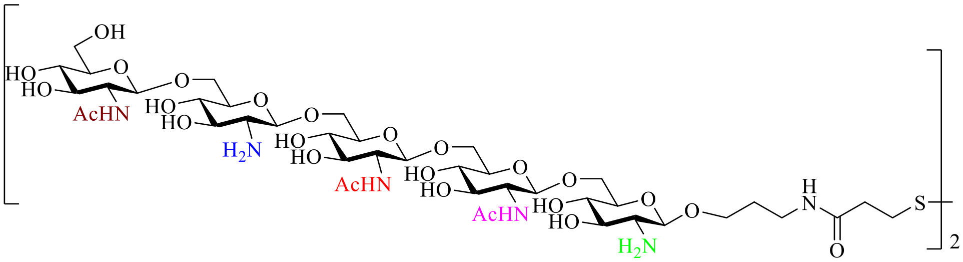

PNAG22 (10110): Compound **2** was subjected to Boc, Troc and Fmoc removals followed by acetylation, removal of Alloc, Bz and TFA according to the general procedures to afford **PNAG22** (2.8 mg, 51% yield for 6 steps). $[\alpha]_D^{20}$ = -2.8 (*c* 0.3, MeOH). ^{1}H-NMR (500 MHz, D_2O): *δ*. 4.61 (d, *J* =8.5 Hz, 1 H, anomeric H), 4.54 (d, *J* =8.5 Hz, 1 H, anomeric H), 4.41 (d, *J* =8.5 Hz, 1 H, anomeric H), 4.36 (d, *J* =8.5 Hz, 1 H, anomeric H), 4.33 (d, *J* =8.5 Hz, 1 H, anomeric H), 4.07-3.99 (m, 4 H), 3.78-3.71 (m, 3 H), 3.63-3.40 (m, 18 H), 3.31-2.18 (m, 6 H), 3.09-3.05 (m, 1 H, $-OCH_2CH_2C\boldsymbol{H}_2NH-$), 2.93-2.78 (m, 4 H, $-C\boldsymbol{H}-NH_2$ and $-C\boldsymbol{H}_2S-$), 2.53 (t, *J* =7.0 Hz, 2 H, $-NHC{=}OC\boldsymbol{H}_2-$), 1.89 (s, 6 H, -NH**Ac**), 1.87 (s, 3 H, -NH**Ac**), 1.70-1.65 (m, 2 H, $-OCH_2C\boldsymbol{H}_2CH_2NH-$). ^{13}C-NMR (125 MHz, D_2O): *δ*. 101.5 (2 C), 101.4, 99.2, 98.8, 75.7, 74.8, 74.2, 73.6, 69.8, 69.6 (2 C), 68.3, 68.1, 67.7, 67.3, 60.5, 55.4, 55.2, 35.9, 34.7, 33.2, 28.3, 22.2 (obtained from HSQC NMR). HRMS: $C_{84}H_{148}N_{12}O_{50}S_2$ $[M + 2 H]^{2+}$ calcd: 1094.4419, obsd: 1092.4414.

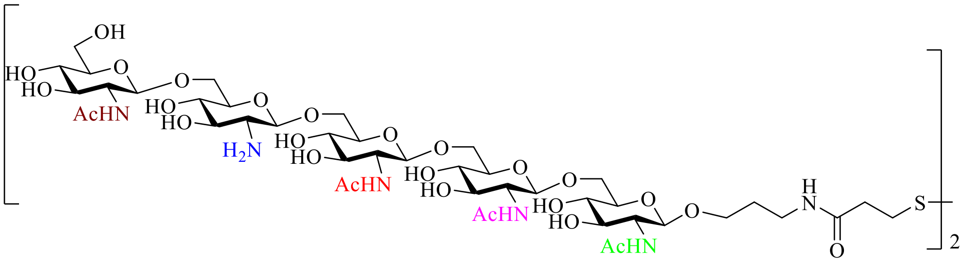

PNAG23 (10111): Compound **1** was subjected to Boc, Troc and Fmoc removals followed by acetylation, removal of Alloc and Bz according to the general procedures to afford **PNAG23** (1.2 mg, 37% yield for 6 steps). $[\alpha]_D^{20}$ = -2.8 (*c* 0.3, MeOH). ^{1}H-NMR (500 MHz, D_2O): *δ*. 4.62 (d, *J* =9.0 Hz, 1 H, anomeric H), 4.42 (d, *J* =8.5 Hz, 1 H, anomeric H), 4.37-4.30 (m, 3 H, anomeric H), 4.07-3.98 (m, 4 H), 3.78-3.72 (m, 4 H), 3.63-3.24 (m, 23 H), 3.13-3.07 (m, 1 H, -$OCH_2CH_2C\boldsymbol{H}_2NH$-), 3.04-2.98 (m, 1 H, -$OCH_2CH_2C\boldsymbol{H}_2NH$-), 2.81 (t, *J* =6.0 Hz, 1 H, -C***H***-NH_2), 2.64-2.48 (m, 3 H, -$C\boldsymbol{H}_2S$- and -NHC=$OC\boldsymbol{H}_2$-), 1.89-1.87 (m, 12 H, -NH**Ac**), 1.63-1.58 (m, 2 H, -$OCH_2C\boldsymbol{H}_2CH_2NH$-). ^{13}C-NMR (125 MHz, D_2O): *δ*. 101.5, 101.4 (2 C), 101.1, 99.0, 75.7, 74.8, 74.2, 73.8, 71.9, 71.8, 69.7, 69.6, 68.5, 68.1 (2 C), 67.5, 60.5, 55.4, 55.2, 36.1, 32.8, 31.0, 28.1, 22.0 (obtained from HSQC NMR). HRMS: $C_{88}H_{152}N_{12}O_{52}S_2$ $[M + 2 H]^{2+}$ calcd: 1136.4525, obsd: 1136.4517.

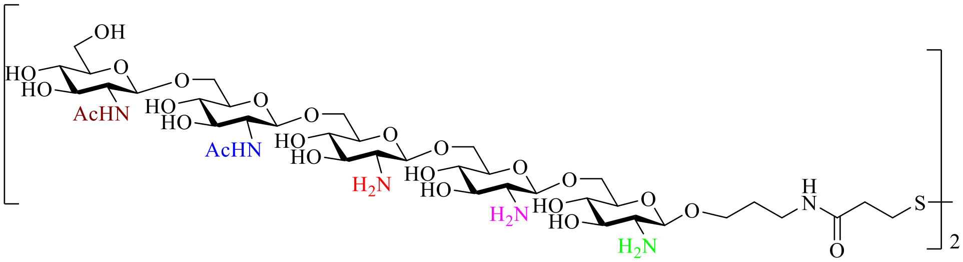

PNAG24 (11000): Compound **2** was subjected to Boc, Alloc removals followed by acetylation, removal of Troc, Fmoc, Bz and TFA according to the general procedures to afford **PNAG24** (2.4 mg, 41% yield for 6 steps). $[\alpha]_D^{20}$ = -0.7 (*c* 0.3, MeOH). ^{1}H-NMR (500 MHz, D_2O): *δ*. 4.65-4.63 (m, 1 H, anomeric H obtained from HSQC NMR), 4.60-4.56 (m, 2 H, anomeric H), 4.36 (d, *J* =9.0 Hz, 1 H, anomeric H), 4.33 (d, *J* =8.5 Hz, 1 H, anomeric H), 4.08-3.97 (m, 4 H), 3.82-3.71 (m, 4 H), 3.63-3.45 (m, 11 H), 3.42-3.34 (m, 5 H), 3.30-3.15 (m, 6 H), 3.09-3.03 (m, 1 H, -$OCH_2CH_2C\boldsymbol{H}_2NH$-), 2.95-2.86 (m, 3 H, -C***H***-NH_2), 2.79 (t, *J* =7.0 Hz, 2 H, -$C\boldsymbol{H}_2S$-), 2.51 (t, *J* =6.5 Hz, 2 H, -NHC=$OC\boldsymbol{H}_2$-), 1.88 (s, 3 H, -NH**Ac**), 1.87 (s, 3 H, -NH**Ac**), 1.69-1.64 (m, 2 H, -$OCH_2C\boldsymbol{H}_2CH_2NH$-). ^{13}C-NMR (125 MHz, D_2O): *δ*. 101.8, 101.6, 99.1, 99.0, 98.8, 75.7, 74.6, 74.4 (2 C), 73.5, 71.8, 69.7 (2 C), 69.5, 68.3 (2 C), 67.7 (2 C), 60.5, 55.4, 55.2, 36.1, 34.7, 33.0, 28.3, 22.2 (obtained from HSQC NMR). HRMS: $C_{80}H_{144}N_{12}O_{48}S_2$ $[M + 2 H]^{2+}$ calcd: 1052.4313, obsd: 1052.4309.

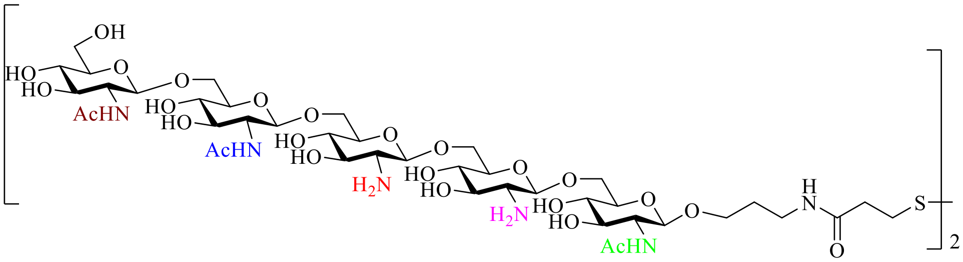

PNAG25 (11001): Compound **1** was subjected to Boc and Alloc removals followed by acetylation, removal of Troc, Fmoc and Bz according to the general procedures to afford **PNAG25** (2.2 mg, 58% yield for 6 steps). $[\alpha]_D^{20}$ = -1.6 (*c* 0.3, MeOH). ^{1}H-NMR (500 MHz, D_2O): *δ*. 4.67-4.65 (m, 1 H, anomeric H obtained from HSQC NMR), 4.62 (d, *J* =8.5 Hz, 1 H, anomeric H), 4.39-4.34 (m, 3 H, anomeric H), 4.10-3.99 (m, 4 H), 3.79-3.73 (m, 4 H), 3.65-3.23 (m, 22 H), 3.13-3.08 (m, 1 H, -$OCH_2CH_2C\boldsymbol{H}_2NH$-), 3.05-3.00 (m, 1 H, -$OCH_2CH_2C\boldsymbol{H}_2NH$-), 2.97-2.89 (m, 2 H, -C***H***-NH_2), 2.81 (t, *J* =6.5 Hz, 2 H, -$C\boldsymbol{H}_2S$-), 2.52 (t, *J* =6.5 Hz, 2 H, -NHC=$OC\boldsymbol{H}_2$-), 1.90 (s, 6 H, -NH**Ac**), 1.89 (s, 3 H, -NH**Ac**), 1.63-1.58 (m, 2 H, -$OCH_2C\boldsymbol{H}_2CH_2NH$-). ^{13}C-NMR (125 MHz, D_2O): *δ*. 101.7, 101.5 (2 C), 99.0 (2 C), 75.7, 74.6, 74.4 (2 C), 74.1 (2 C), 73.6, 71.9, 71.8, 69.9 (2 C), 69.7 (2 C), 68.5, 68.4, 68.3, 60.7, 55.6, 55.4, 36.3, 34.9, 33.4, 28.5, 22.2 (obtained from HSQC NMR). HRMS: $C_{84}H_{148}N_{12}O_{50}S_2$ $[M + 2 H]^{2+}$ calcd: 1094.4419, obsd: 1092.4402.

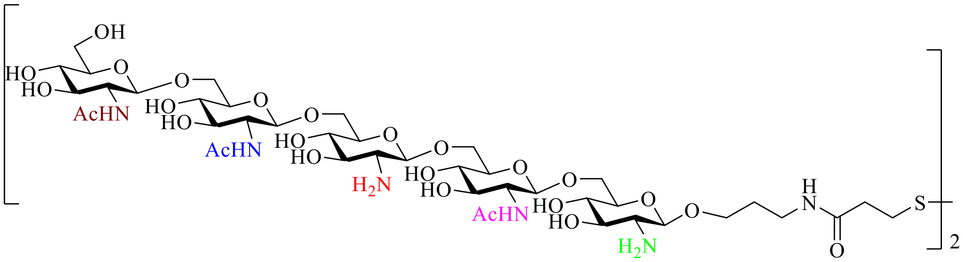

PNAG26 (11010): Compound **2** was subjected to Boc, Alloc and Fmoc removals followed by acetylation, removal of Troc, Bz and TFA according to the general procedures to afford **PNAG26** (2.3 mg, 51% yield for 6 steps). $[\alpha]_D^{20}$ = -2.5 (*c* 0.3, MeOH). ^{1}H-NMR (500 MHz, D_2O): *δ*. 4.61 (d, *J* =8.5 Hz, 1 H, anomeric H), 4.54 (d, *J* =8.5 Hz, 1 H, anomeric H), 4.39-4.32 (m, 3 H, anomeric H), 4.06-3.99 (m, 4 H), 3.77-3.70 (m, 3 H), 3.63-3.18 (m, 24 H), 3.09-3.03 (m, 1 H, -$OCH_2CH_2C\boldsymbol{H}_2NH$-), 2.92-2.78 (m, 4 H, -C***H***-NH_2 and -$C\boldsymbol{H}_2S$-), 2.52 (t, *J* =7.0 Hz, 2 H, -NHC=$OC\boldsymbol{H}_2$-), 1.89 (s, 3 H, -NH**Ac**), 1.88 (ss, 6 H, -NH**Ac**), 1.69-1.64 (m, 2 H, -$OCH_2C\boldsymbol{H}_2CH_2NH$-). ^{13}C-NMR (125 MHz, D_2O): *δ*. 101.4, 101.3 (2 C), 98.8, 98.4, 75.7, 74.5, 74.4, 73.6, 71.6, 69.6 (2 C), 68.3, 68.1, 67.5, 60.5, 55.6, 55.4, 36.1, 34.9, 33.2, 28.5, 22.4 (obtained from HSQC NMR). HRMS: $C_{84}H_{148}N_{12}O_{50}S_2$ $[M + 2 H]^{2+}$ calcd: 1094.4419, obsd: 1094.4406.

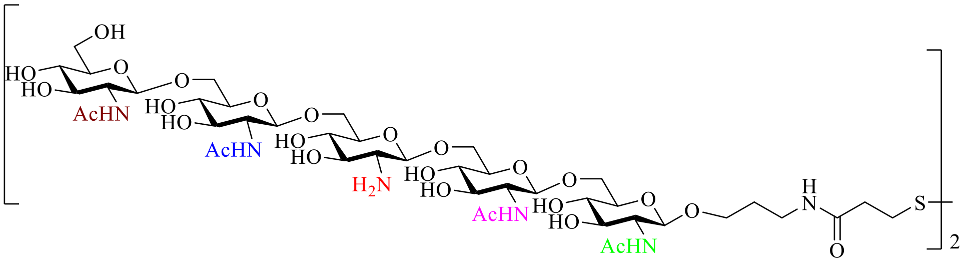

PNAG27 (11011): Compound **1** was subjected to Boc, Alloc and Fmoc removals followed by acetylation, removal of Troc and Bz according to the general procedures to afford **PNAG27** (1.5 mg, 33% yield for 6 steps). $[\alpha]_D^{20}$ = -3.0 (*c* 0.3, MeOH). ^{1}H-NMR (500 MHz, D_2O): *δ.* 4.62-4.60 (m, 1 H, anomeric H obtained from HSQC NMR), 4.41-4.30 (m, 4 H, anomeric H), 4.07-3.99 (m, 4 H), 3.78-3.70 (m, 4 H), 3.62-3.20 (m, 23 H), 3.13-3.07 (m, 1 H, $-OCH_2CH_2C\boldsymbol{H}_2NH-$), 3.03-2.98 (m, 1 H, $-OCH_2CH_2C\boldsymbol{H}_2NH-$), 2.93-2.89 (m, 1 H, $-C\boldsymbol{H}-NH_2$), 2.80 (t, *J* =6.5 Hz, 1 H, $-C\boldsymbol{H}_2S-$), 2.63-2.49 (m, 3 H, $-C\boldsymbol{H}_2S-$ and $-NHC{=}OC\boldsymbol{H}_2-$), 1.89 (s, 3 H, -NH**Ac**), 1.88 (ss, 6 H, -NH**Ac**), 1.87 (s, 3 H, -NH**Ac**), 1.63-1.58 (m, 2 H, $-OCH_2C\boldsymbol{H}_2CH_2NH-$). ^{13}C-NMR (125 MHz, D_2O): *δ.* 101.5, 101.4 (2 C), 101.3, 99.0, 75.7, 74.4, 74.2, 73.8, 71.8, 69.9 (2 C), 69.8, 68.5, 68.4, 68.1, 67.5, 60.5, 55.4, 55.2, 36.3, 34.9, 33.2, 28.1, 22.2 (obtained from HSQC NMR). HRMS: $C_{88}H_{152}N_{12}O_{52}S_2$ [M + 2 H]$^{2+}$ calcd: 1136.4525, obsd: 1136.4498.

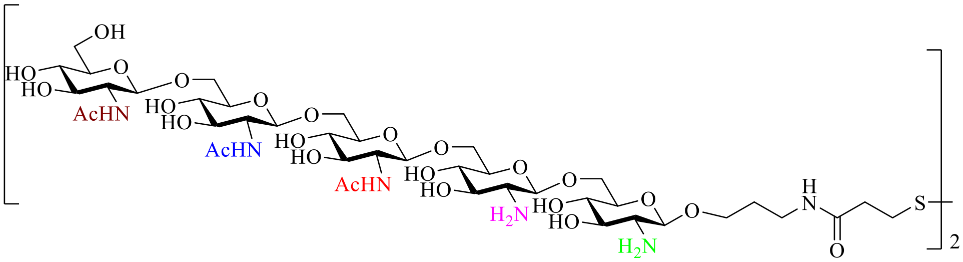

PNAG28 (11100): Compound **2** was subjected to Boc, Alloc and Troc removals followed by acetylation, removal of Fmoc, Bz and TFA according to the general procedures to afford **PNAG28** (3.4 mg, 50% yield for 6 steps). $[\alpha]_D^{20}$ = -1.9 (*c* 0.3, MeOH). ^{1}H-NMR (500 MHz, D_2O): *δ.* 4.60-4.56 (m, 2 H, anomeric H), 4.37-4.33 (m, 3 H, anomeric H), 4.03-3.97 (m, 4 H), 3.80-3.73 (m, 4 H), 3.62-3.35 (m, 17 H), 3.29-3.18 (m, 6 H), 3.10-3.05 (m, 1 H, $-OCH_2CH_2C\boldsymbol{H}_2NH-$), 2.91-2.86 (m, 2 H, $-C\boldsymbol{H}-NH_2$), 2.80 (t, *J* =6.5 Hz, 2 H, $-C\boldsymbol{H}_2S-$), 2.52 (t, *J* =8.5 Hz, 2 H, $-NHC{=}OC\boldsymbol{H}_2-$), 1.88 (s, 9 H, -NH**Ac**), 1.69-1.64 (m, 2 H, $-OCH_2C\boldsymbol{H}_2CH_2NH-$). ^{13}C-NMR (125 MHz, D_2O): *δ.* 101.9 (2 C), 101.7, 99.0, 98.9, 75.9, 74.8, 74.6, 74.5 (2 C), 73.8, 72.0, 71.8, 69.9, 69.7, 68.7 (2 C), 68.5, 67.9, 67.7, 60.5, 55.6, 55.4, 36.1, 34.9, 33.2, 28.3, 22.2 (obtained from HSQC NMR). HRMS: $C_{84}H_{148}N_{12}O_{50}S_2$ [M + 2 H]$^{2+}$ calcd: 1094.4419, obsd: 1094.4414.

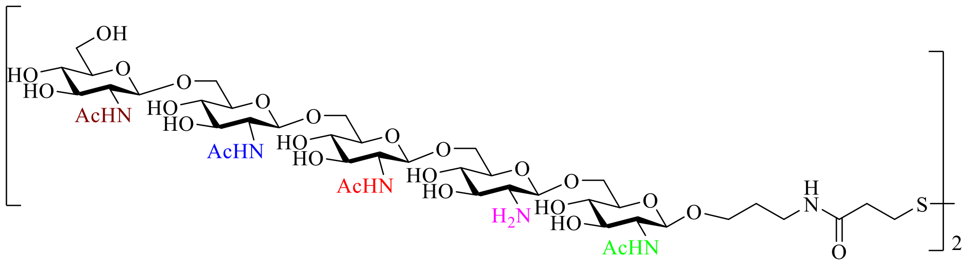

PNAG29 (11101): Compound **2** was subjected to Boc, Alloc and Troc removals followed by acetylation, removal of Fmoc and Bz according to the general procedures to afford **PNAG29** (1.5 mg, 36% yield for 6 steps). $[\alpha]_D^{20}$ = -2.2 (*c* 0.3, MeOH). ^{1}H-NMR (500 MHz, D_2O): *δ*. 4.61 (d, *J* =9.0 Hz, 1 H, anomeric H), 4.38-4.34 (m, 4 H, anomeric H), 4.04-3.98 (m, 4 H), 3.77-3.68 (m, 4 H), 3.62-3.21 (m, 23 H), 3.13-3.07 (m, 1 H, $-OCH_2CH_2C\boldsymbol{H}_2NH-$), 3.02-2.99 (m, 1 H, $-OCH_2CH_2C\boldsymbol{H}_2NH-$), 2.92 (dd, *J* =9.0, 10.0 Hz, 1 H, $-C\boldsymbol{H}-NH_2$), 2.80 (t, *J* =7.0 Hz, 1 H, $-C\boldsymbol{H}_2S-$), 2.62-2.47 (m, 3 H, $-C\boldsymbol{H}_2S-$ and $-NHC{=}OC\boldsymbol{H}_2-$), 1.89-1.88 (m, 12 H, -NH**Ac**), 1.62-1.57 (m, 2 H, $-OCH_2C\boldsymbol{H}_2CH_2NH-$). ^{13}C-NMR (125 MHz, D_2O): *δ*. 101.8, 101.5 (2 C), 101.3, 99.0, 75.7, 74.6, 74.3, 73.8, 72.0, 69.9 (2 C), 69.7, 68.3 (2 C), 67.9, 60.7, 55.6, 55.4, 36.7, 36.3, 29.1, 28.3, 22.2 (obtained from HSQC NMR). HRMS: $C_{88}H_{152}N_{12}O_{52}S_2$ $[M + 2 H]^{2+}$ calcd: 1136.4525, obsd: 1136.4520.

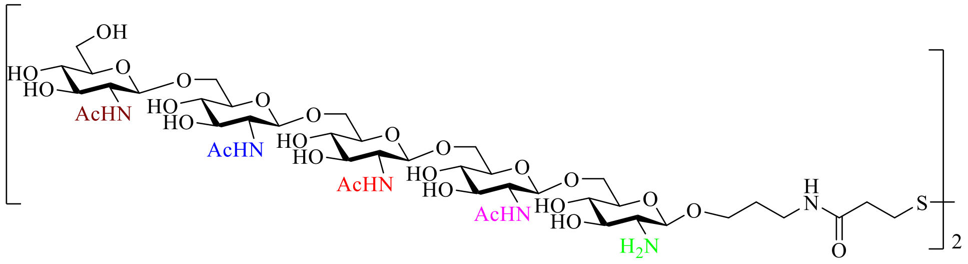

PNAG30 (11110): Compound **2** was subjected to Boc, Alloc, Troc and Fmoc removals followed by acetylation, removal of Bz and TFA according to the general procedures to afford **PNAG30** (1.0 mg, 40% yield for 6 steps). $[\alpha]_D^{20}$ = -2.1 (*c* 0.2, MeOH). ^{1}H-NMR (500 MHz, D_2O): *δ*. 4.54 (d, *J* =8.5 Hz, 1 H, anomeric H), 4.38-4.32 (m, 4 H, anomeric H), 4.4.04-3.98 (m, 4 H), 3.77-3.73 (m, 3 H), 3.61-3.35 (m, 18 H), 3.29-3.18 (m, 7 H), 3.09-3.03 (m, 1 H, $-OCH_2CH_2C\boldsymbol{H}_2NH-$), 2.87 (t, *J* =10.5 Hz, 1 H, $-C\boldsymbol{H}-NH_2$), 2.83-2.78 (m, 2 H, $-C\boldsymbol{H}_2S-$), 2.53-2.49 (m, 2 H, $-NHC{=}OC\boldsymbol{H}_2-$), 1.89 (s, 3 H, -NH**Ac**), 1.88 (s, 6 H, -NH**Ac**), 1.87 (s, 3 H, -NH**Ac**), 1.70-1.64 (m, 2 H, $-OCH_2C\boldsymbol{H}_2CH_2NH-$). ^{13}C-NMR (125 MHz, D_2O): *δ*. 101.5 (2 C), 101.4 (2 C), 98.6, 75.7, 74.7, 74.0, 73.6, 71.8, 69.9, 69.7, 68.2, 67.8, 67.2, 60.5, 55.4, 55.2, 36.1, 34.7, 33.2, 28.3, 22.2 (obtained from HSQC NMR). HRMS: $C_{88}H_{152}N_{12}O_{52}S_2$ $[M + 2 H]^{2+}$ calcd: 1136.4525, obsd: 1136.4508.

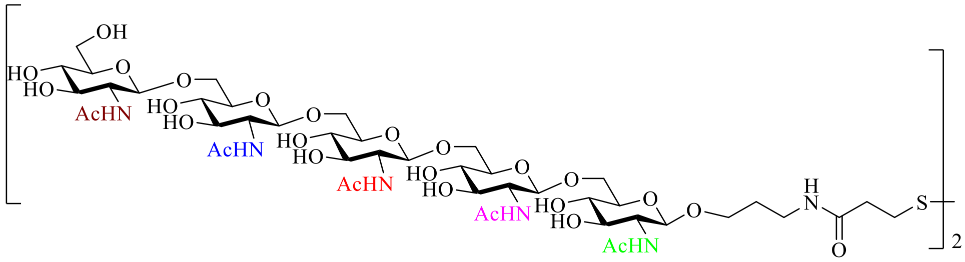

PNAG31 (11111): Compound **1** was subjected to Boc, Alloc, Troc and Fmoc removals followed by acetylation, removal of Bz according to the general procedures to afford **PNAG31** (3.5 mg, 38% yield for 6 steps). $[\alpha]_D^{20}$ = -2.4 (*c* 0.2, MeOH). ^{1}H-NMR (500 MHz, D_2O): *δ*. 4.38-4.33 (m, 4 H, anomeric H), 4.30 (d, *J* =8.0 Hz, 1 H, anomeric H), 4.33 (d, *J* =9.0 Hz, 2 H), 4.04-3.98 (m, 4 H), 3.77-3.68 (m, 3 H), 3.61-3.47 (m, 10 H), 3.43-3.33 (m, 9 H), 3.29-3.19 (m, 6 H), 3.14-3.09 (m, 1 H, $-OCH_2CH_2C\boldsymbol{H}_2NH-$), 3.03-2.97 (m, 1 H, $-OCH_2CH_2C\boldsymbol{H}_2NH-$), 2.80 (t, *J* =7.0 Hz, 2 H, -$C\boldsymbol{H}_2S$-), 2.51 (t, *J* =6.5 Hz, 2 H, -NHC=O$C\boldsymbol{H}_2$-), 1.88-1.86 (m, 15 H, -NH**Ac**), 1.62-1.57 (m, 2 H, $-OCH_2C\boldsymbol{H}_2CH_2NH-$). ^{13}C-NMR (125 MHz, D_2O): *δ*. 101.3 (4 C), 101.1, 75.7, 74.4, 73.5, 69.8, 69.7, 68.4, 68.3, 60.5, 55.4, 55.2, 36.1, 34.7, 33.0, 28.1, 22.0 (obtained from HSQC NMR). HRMS: $C_{92}H_{156}N_{12}O_{54}S_2$ $[M + 2 H]^{2+}$ calcd: 1178.4630, obsd: 1178.4628.

Supplementary Tables

Supplementary Table 1. Staphylococcus strains used in this study.

**strain**	**description**	**reference**
41	JE2; Laboratory derived wild type parental MRSA; USA300_LAC; CC8	2
954	*icaA*::Tn; NTML NE37 *bursa aurealis* transposon (Tn) mutant, Erm^R	2
**clinical isolates**		
1055	MRSA abscess hand cellulitis	3
1056	MRSA abscess left arm	3
1057	MRSA left wrist/ index finger	3
1058	MSSA abscess left foot osteomyelitis	this study
1059	MSSA bone from the coccyx, chronic osteomyelitis	3
1153	MRSA hallux bone	this study

Supplementary Figures.

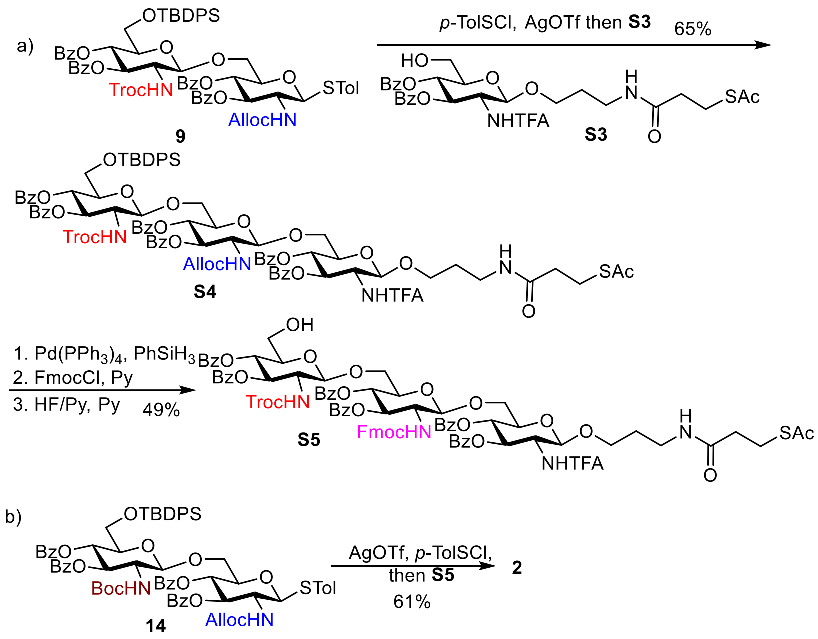

Supplementary Fig. 1. Syntheses of a) compound **S5** and b) compound **2**. Abbreviations: acetyl (Ac), allyloxycarbonyl (Alloc), benzoyl (Bz), tert-butyldiphenylsilyl (TBDPS), fluorenylmethoxycarbonyl (Fmoc), 2,2,2-trichloroethoxycarbonyl (Troc), and trifluoroacetic acid (TFA).

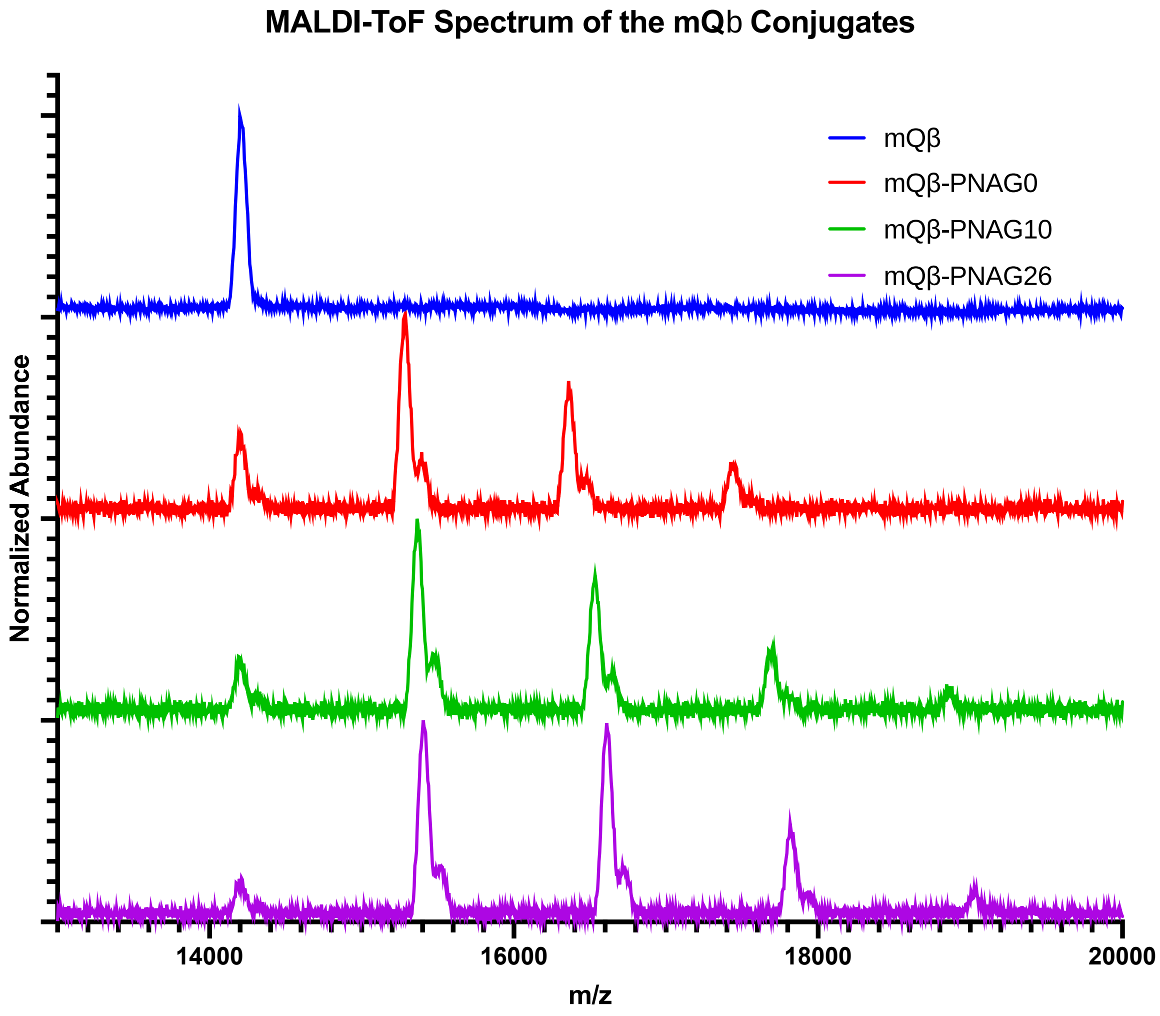
MALDI-ToF Spectrum of the mQb Conjugates
mQβ
mQβ-PNAG0
mQβ-PNAG10
mQβ-PNAG26
Normalized Abundance
14000
16000
18000
20000
m/z

A)

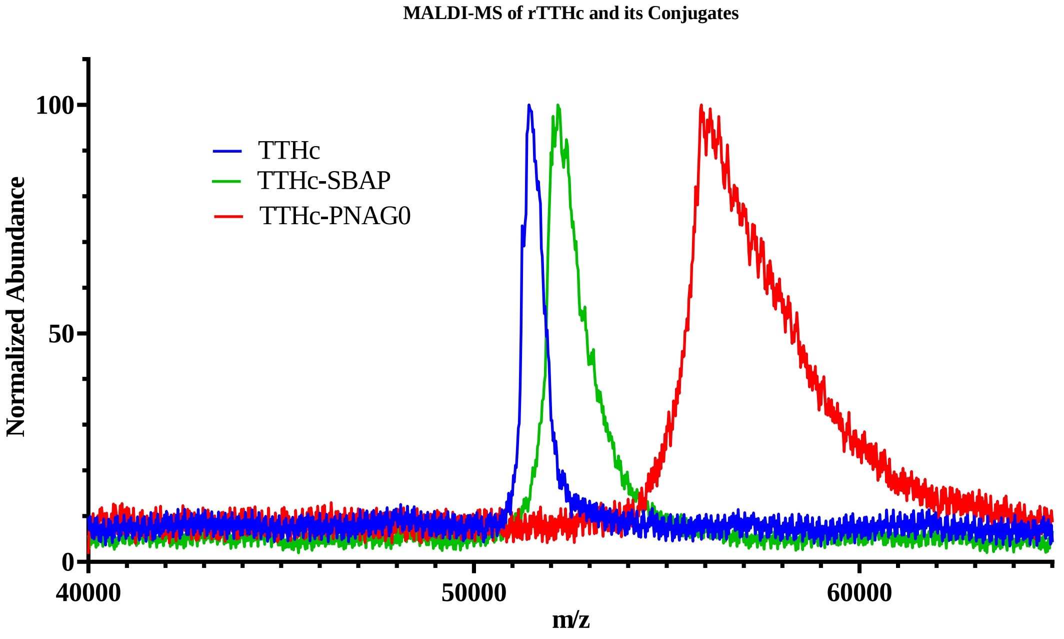
MALDI-MS of rTTHc and its Conjugates
TTHc
TTHc-SBAP
TTHc-PNAG0
Normalized Abundance
100
50
0
40000
50000
60000
m/z

B)

Supplementary Fig. 2. MALDI-TOF MS spectra of PNAG conjugates with mQβ and TTHc. A) MALDI-TOF MS spectrum of the mQβ-PNAG conjugates. The peak assignment was shown in the table below. The average glycan loading per particle was calculated by

$$(1)\ L = 180 \times \frac{\sum N_i A_i}{\sum A_i}$$

where 180 is the number of subunits in each VLP, N is the number of glycan loading in a specific peak, and A is the relative abundance of the corresponding peak. The shoulder peaks showing at +113 position of the major peaks are due to +1 linker as a result of intrasubunit crosslinking. The average loading is around 250 glycans per mQβ particle based on the MS results. B) MALDI-TOF MS characterization of TTHc conjugates. TTHc-SBAP: SBAP functionalized TTHc. The average glycan loading on the PNAG0 conjugates was calculated to be 4.7 glycans per TTHc molecule.

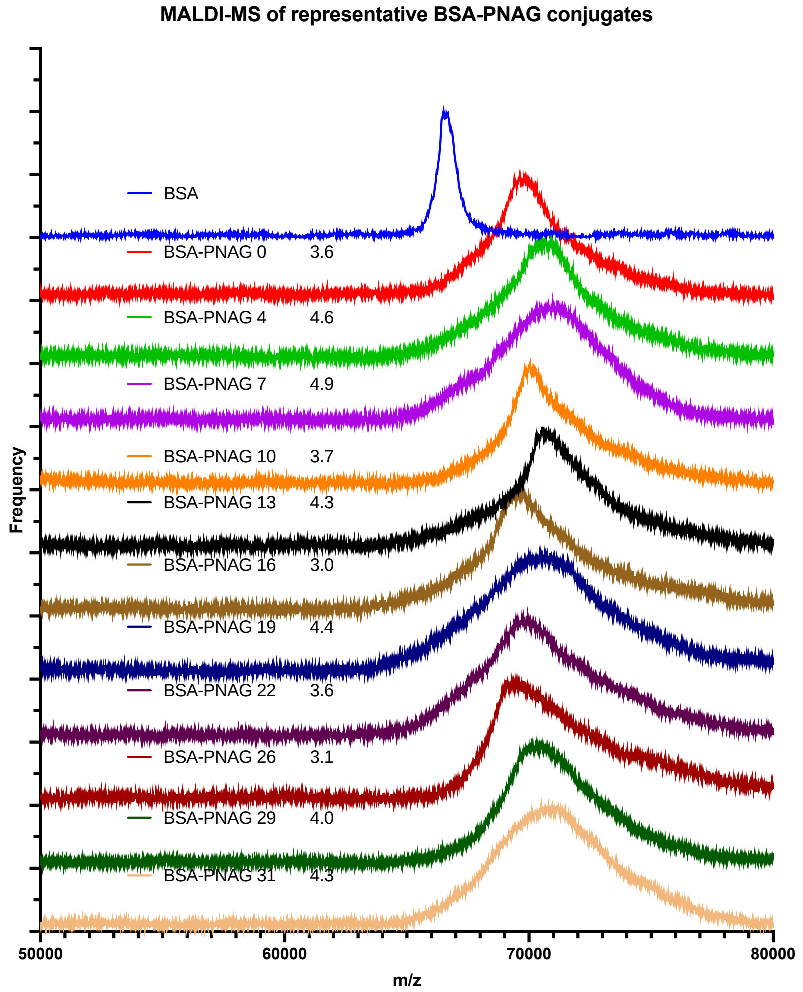

Supplementary Fig. 3. MALDI-TOF mass spectra of representative BSA-PNAG conjugates. Average loadings of glycans per BSA are presented.

CD1 Mouse Antibody Response

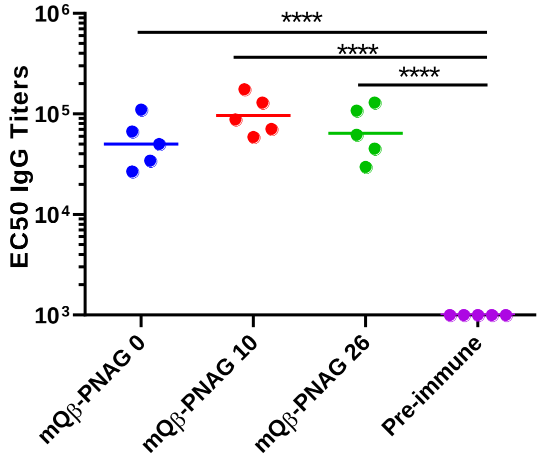

Supplementary Fig. 4. Immunization of CD1 mice (n=5 per group) with mQβ-PNAG led to high levels of anti-PNAG IgG antibodies on day 35 after immunization. The EC50 values (the fold of serum dilution that gives half-maximal binding) of the IgG titers were plotted with each symbol representing one animal and the horizontal line is the geometrical mean value of the titers within the group. The ELISA titers were determined using the BSA-PNAG conjugate containing the same PNAG structure as the immunizing mQβ-PNAG construct. One-way Anova allowed for rejection of the null hypothesis that all groups have the same mean IgG titers (P < 0.0001). **** Statistical significance (P<0.0001) by Dunnett's multiple comparisons post-hoc test. Source data are provided as a Source Data file.

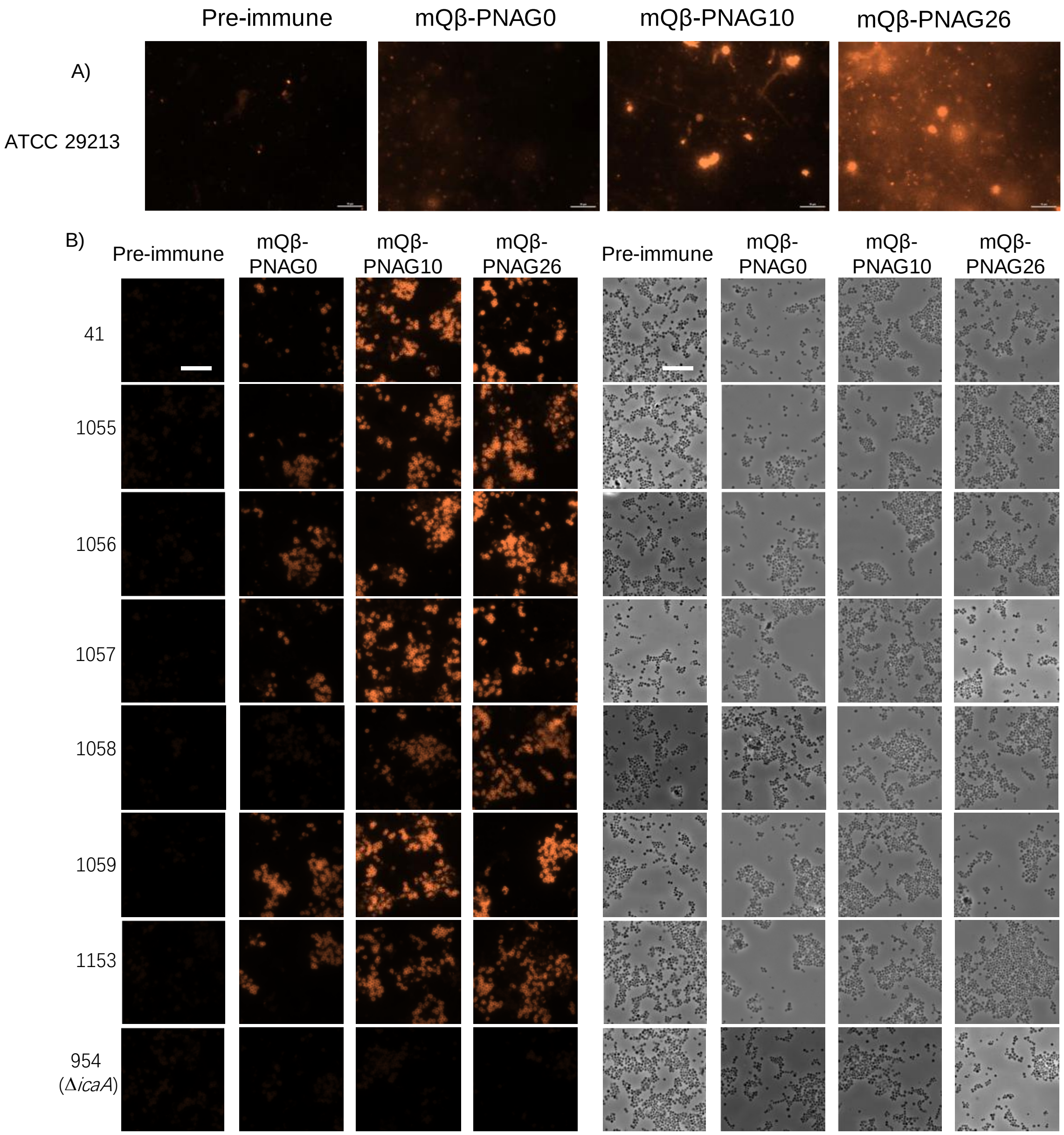

Supplementary Fig. 5. *S. aureus* cells stained with pre-immune and post-immune sera. A) *S. aureus* ATCC 29213 cells were stained by pre-immune, and post-immune sera from rabbits immunized with mQβ-PNAG0, mQβ-PNAG10, and mQβ-PNAG26 respectively followed by the addition of Alexa Fluor 594 labeled goat anti rabbit IgG Fc 2nd antibody. The binding was visualized through fluorescence microscopy. Sera from rabbits immunized with mQβ-PNAG10, and mQβ-PNAG26 showed significant binding to the bacteria. The scale bar is 10 µm. B) Fluorescence images and bright field images of MRSA strains, i.e., 41, 1055, 1056, 1057, 1058, 1059, and 1153 upon staining with pre-immune sera as well as post-immune sera from rabbits immunized with mQβ-PNAG0, mQβ-PNAG10, and mQβ-PNAG26 respectively. The post-immune sera bound with these strains well as detected by the Alexa Fluor 594 labeled goat anti-rabbit IgG Fc 2nd antibody. In comparison, a control strain lacking PNAG expression with *icaA* gene knock out (954) showed negligible binding by the sera, suggesting the recognition is PNAG dependent. Scale bar: 10 µm.

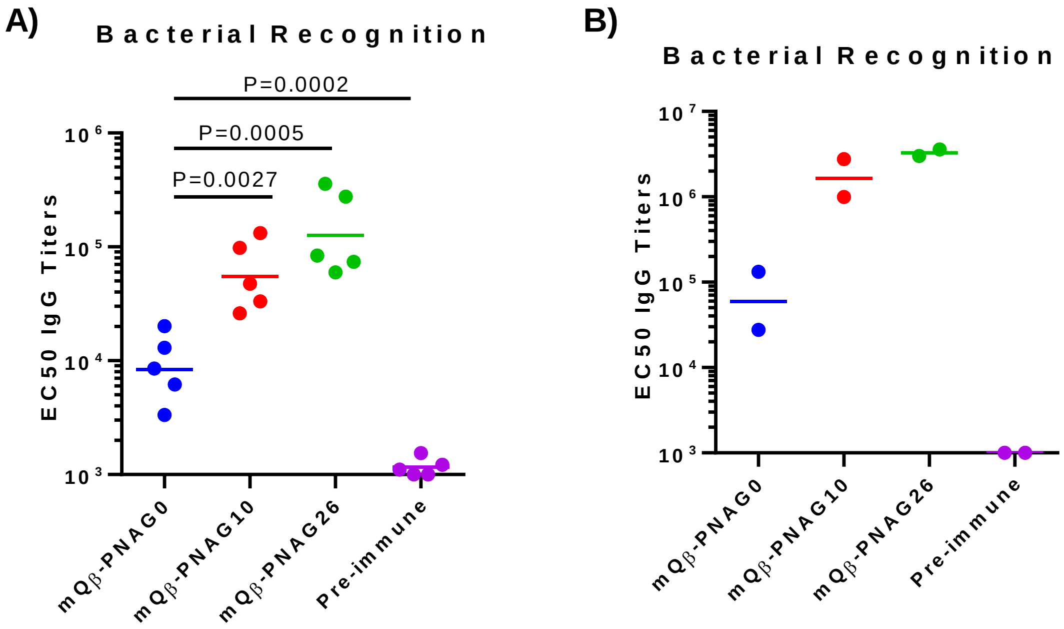

Supplementary Fig. 6. Bacterial recognition by post-immune sera. Post-immune sera of A) mice and B) rabbits immunized with the mQβ-PNAG conjugates recognized the *S. aureus* cells well as determined by ELISA. The EC50 values (the fold of serum dilution that gives half-maximal binding) of the IgG titers were plotted with each symbol representing one animal and the horizontal line is the geometrical mean value of the titers within the group. The ELISA titers were determined against *S. aureus* cell coated ELISA wells. Statistical analysis was performed using one way ANOVA. Source data are provided as a Source Data file.

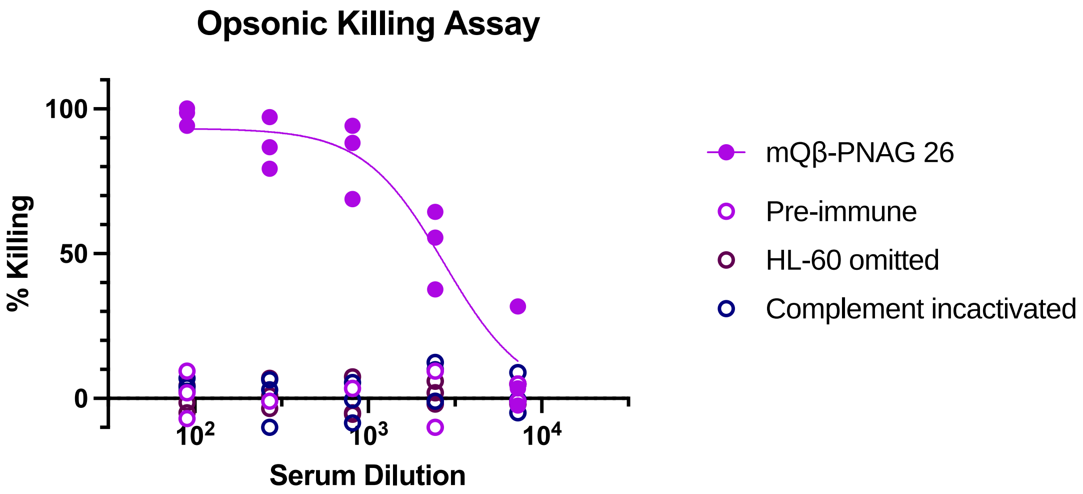

Supplementary Fig. 7. Opsonic killing is dependent on all factors including bacterial specific serum antibody, active complement source, and phagocytic cells. Here is an example showing omitting each factor completely abrogates killing activity. Complement inactivation was performed under 56 °C for 30 mins. HL-60 omitted group and complement inactivated group were carried out with mQβ-PNAG26 serum. The sera used were pooled from rabbits (n =2 per group) immunized with mQβ-PNAG conjugate. Three aliquots were prepared from each pooled serum and the individual values of the three aliquots were presented. Source data are provided as a Source Data file.

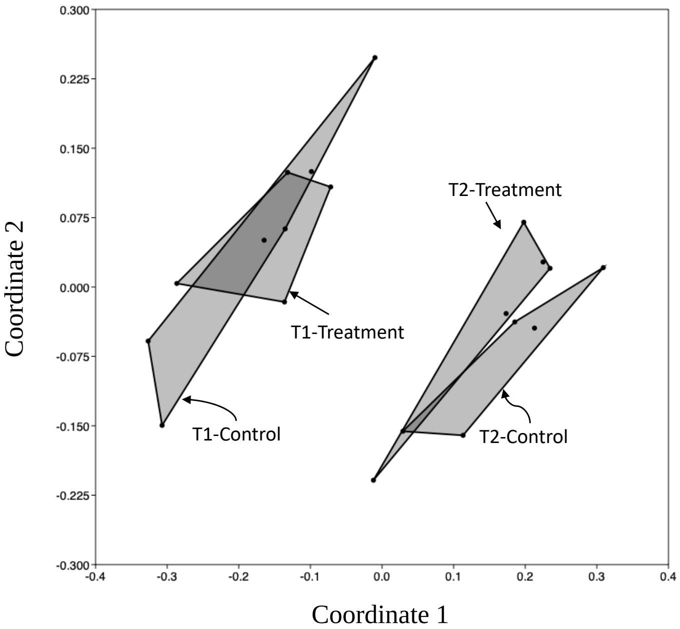

Supplementary Fig. 8. Non-Metric Multidimensional Scaling (NMDS) analysis of bacterial communities of controls and treatments showing no significant changes in the gut microbial community following mQβ-PNAG26 immunization. T1 and T2 refer to the two sampling times. T1 = first sampling right before immunization, T2 = control and treatment samples at 42 days after the prime immunization. Each of the four groups is delineated by a convex hull describing a minimum area. The stress test values for 2D and 3D NMDS were 0.141 and 0.109, respectively. Amplicon sequence variants (ASV) datasets revealed similar patterns of ordination. When comparing treatment versus control within a timepoint, ANalysis Of SIMilarity (ANOSIM) and Permutational multivariate analysis of variance (PERMANOVA) values of -0.04 (T1) and 1.518 (T2) indicated high similarity in the phylogenetic structure of the bacterial communities. Source data are provided as a Source Data file.

Supplementary Fig. 9. ^{1}H-NMR spectrum of compound **3**.

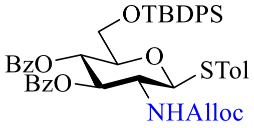

^{1}H-NMR ($CDCl_3$, 500 MHz) of **3**

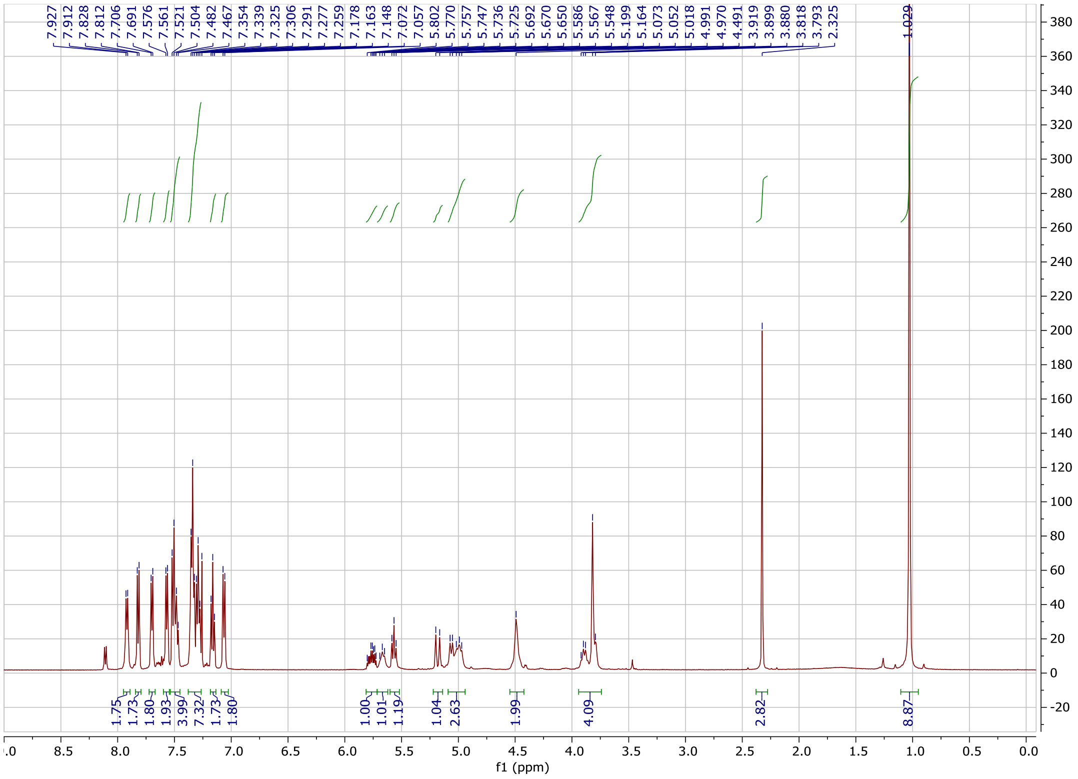

Supplementary Fig. 10. ^{13}C-NMR spectrum of compound **3**.

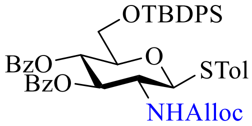

^{13}C-NMR ($CDCl_3$, 125 MHz) of **3**

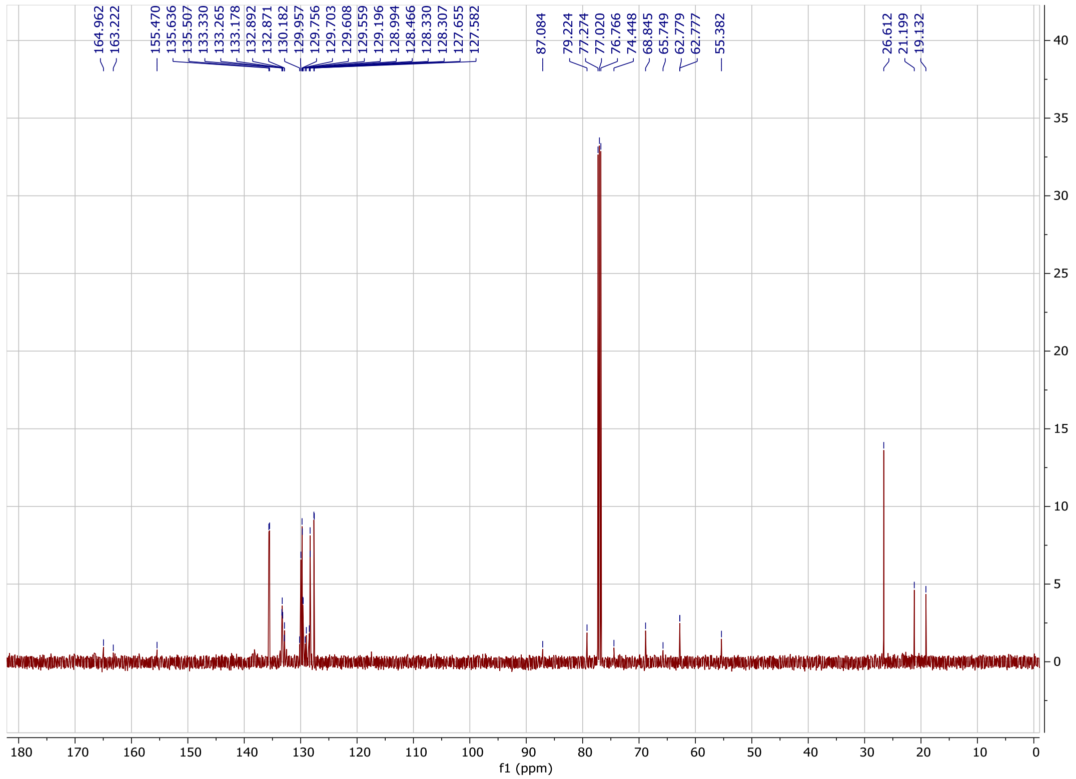

Supplementary Fig. 11. ^{1}H-NMR spectrum of compound **5**.

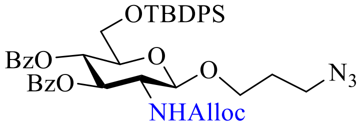

^{1}H-NMR ($CDCl_3$, 500 MHz) of **5**

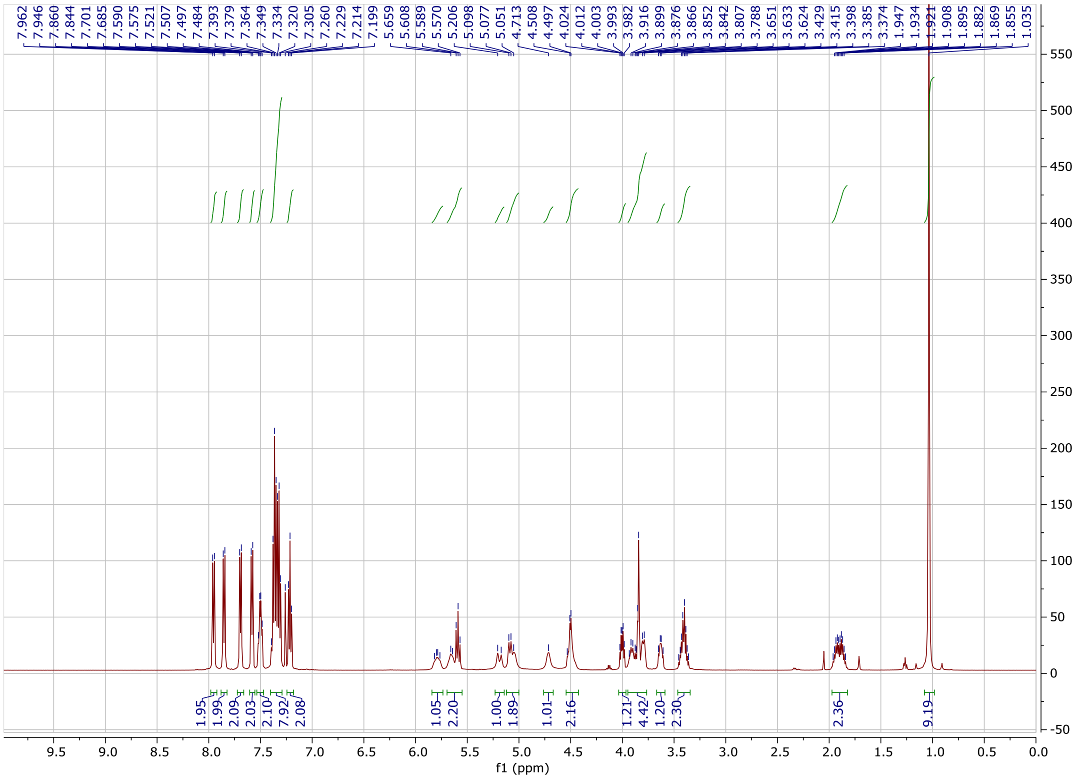

Supplementary Fig. 12. ^{13}C-NMR spectrum of compound **5**.

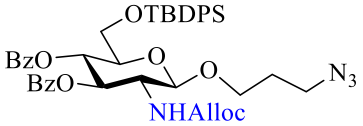

^{13}C-NMR ($CDCl_3$, 125 MHz) of **5**

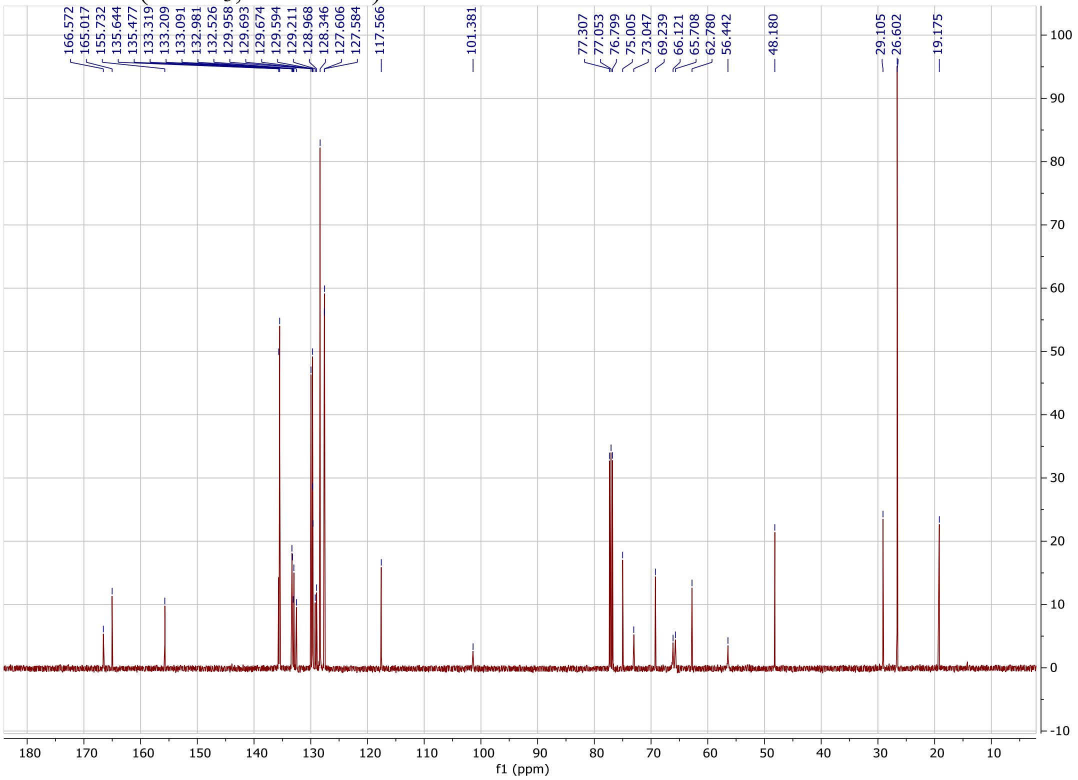

Supplementary Fig. 13. ^{1}H-NMR spectrum of compound **6**.

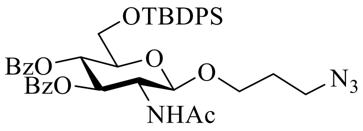

^{1}H-NMR ($CDCl_3$, 500 MHz) of **6**

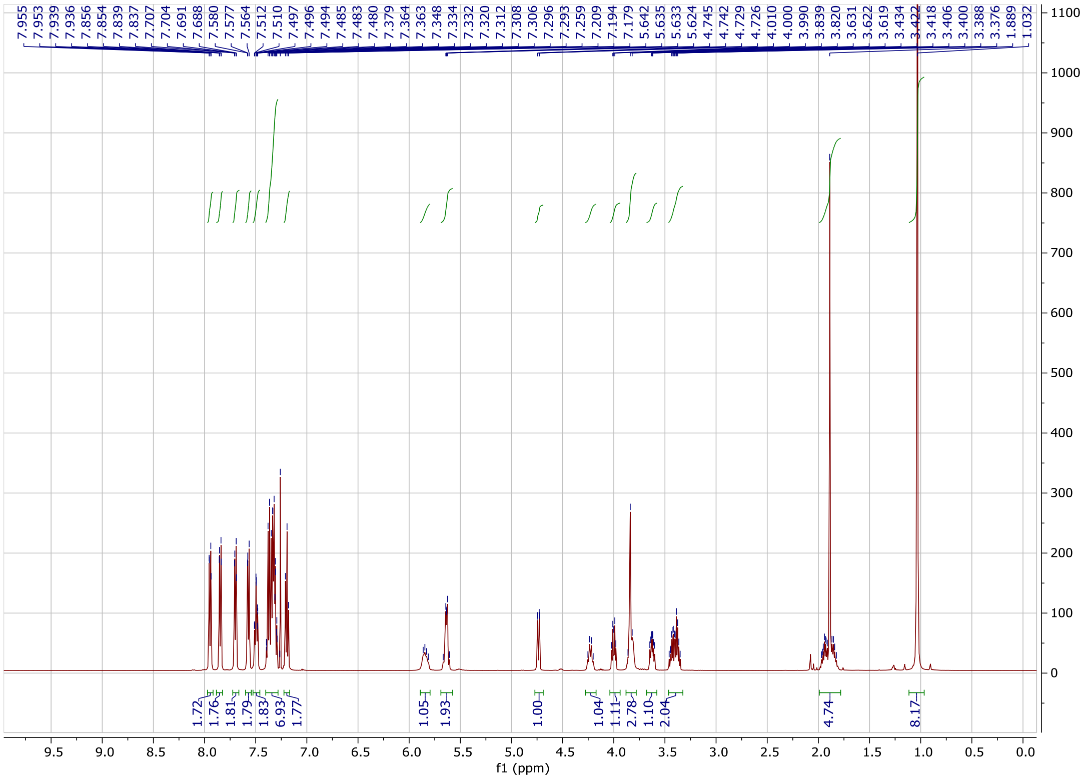

Supplementary Fig. 14. ^{13}C-NMR spectrum of compound **6**.

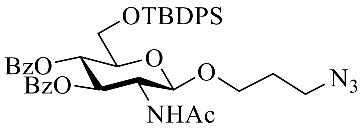

^{13}C-NMR ($CDCl_3$, 125 MHz) of **6**

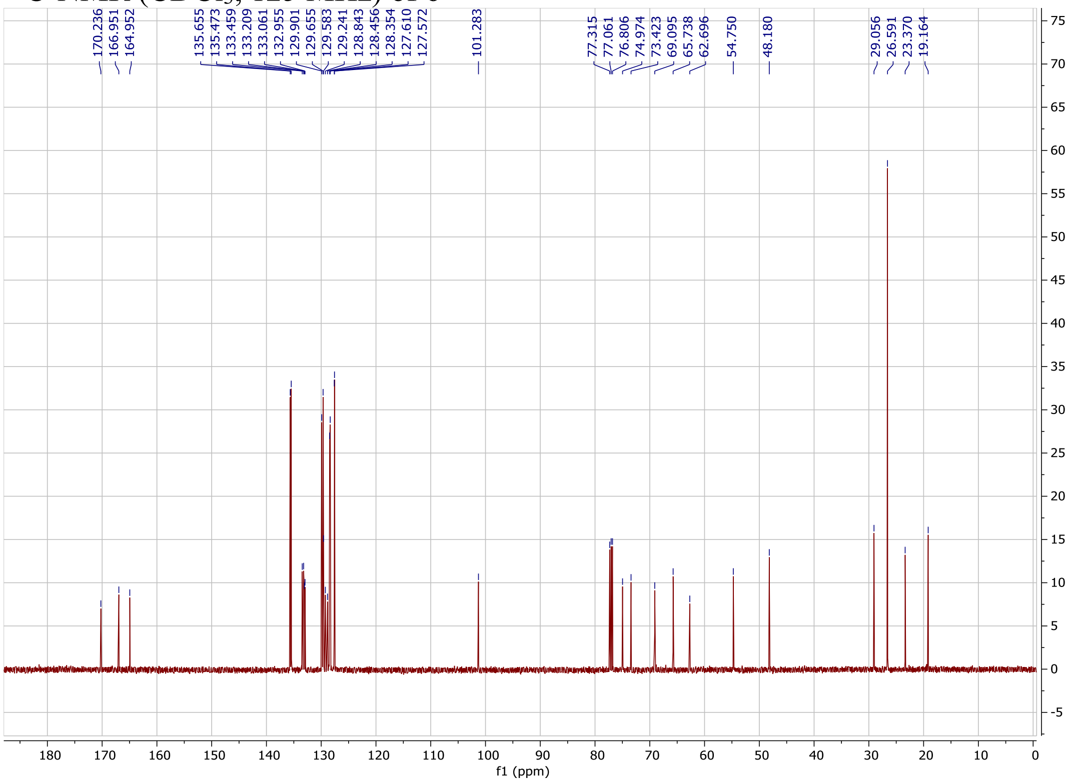

Supplementary Fig. 15. ^{1}H-NMR spectrum of compound **8**.

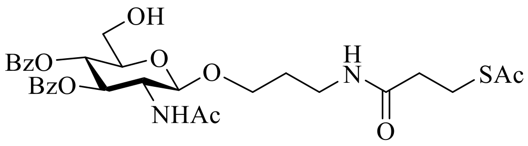

^{1}H-NMR ($CDCl_3$, 500 MHz) of **8**

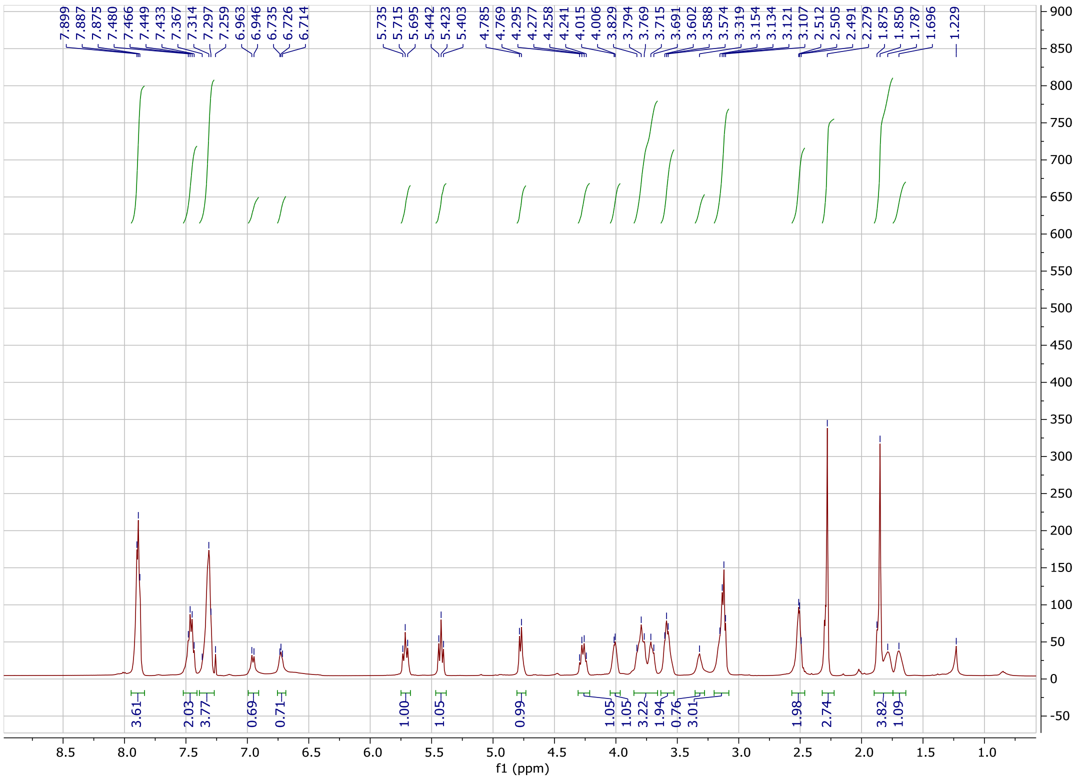

Supplementary Fig. 16. ^{13}C-NMR spectrum of compound **8**.

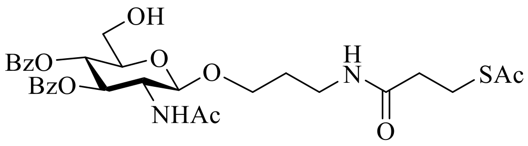

^{13}C-NMR ($CDCl_3$, 125 MHz) of **8**

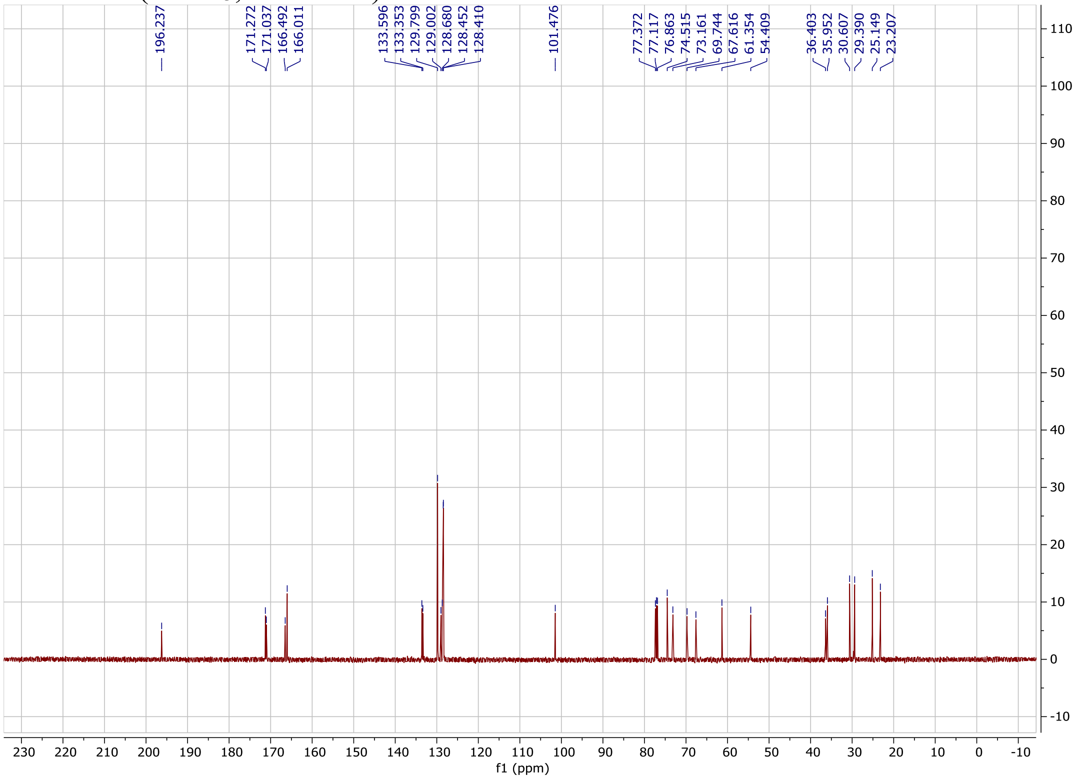

Supplementary Fig. 17. ^{1}H-NMR spectrum of compound **9**.

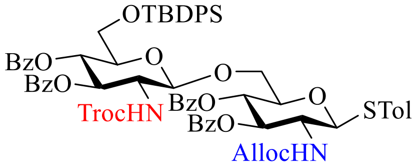

^{1}H-NMR ($CDCl_3$, 500 MHz) of **9**

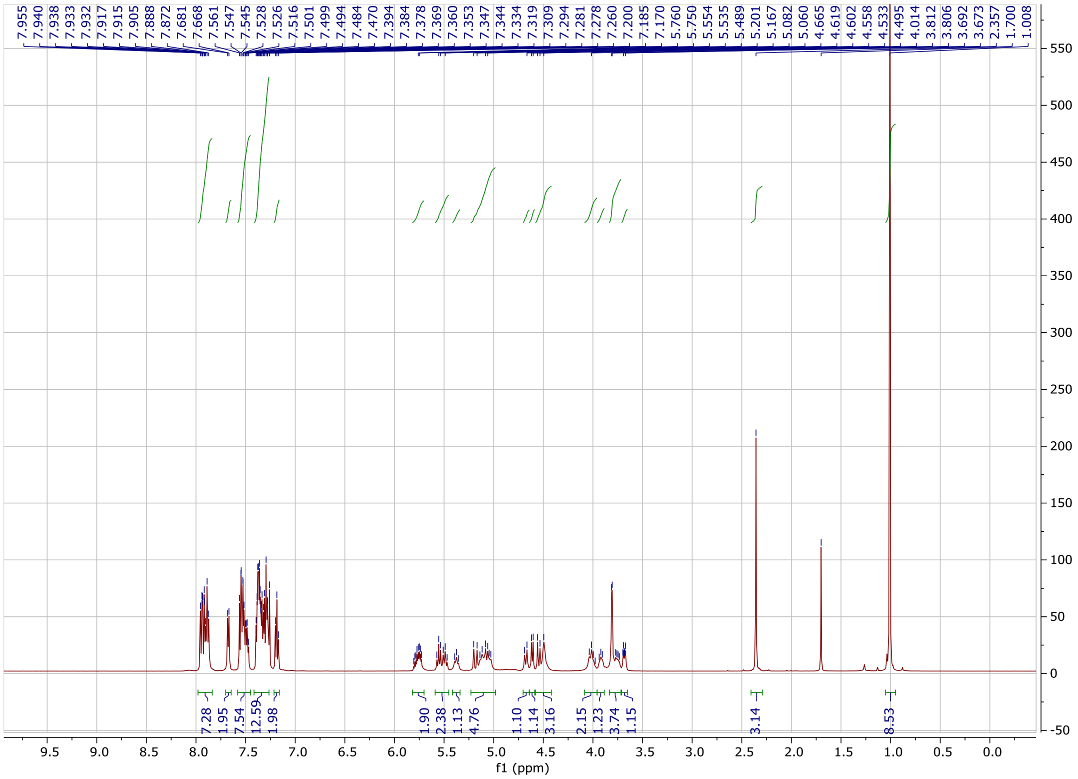

Supplementary Fig. 18. ^{13}C-NMR spectrum of compound **9**.

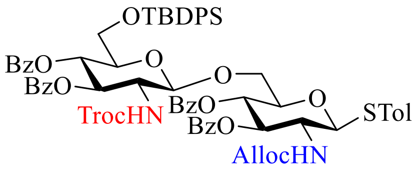

^{13}C-NMR ($CDCl_3$, 125 MHz) of **9**

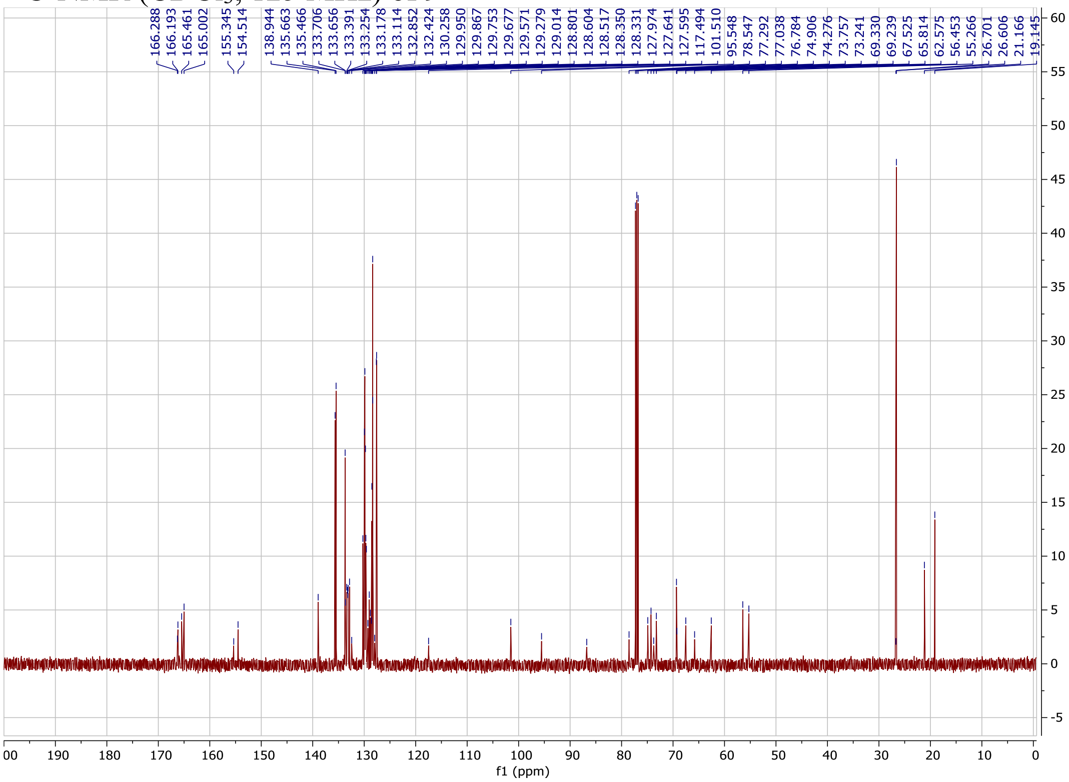

Supplementary Fig. 19. ^{1}H-NMR spectrum of compound **10**.

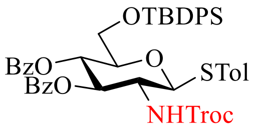

^{1}H-NMR ($CDCl_3$, 500 MHz) of **10**

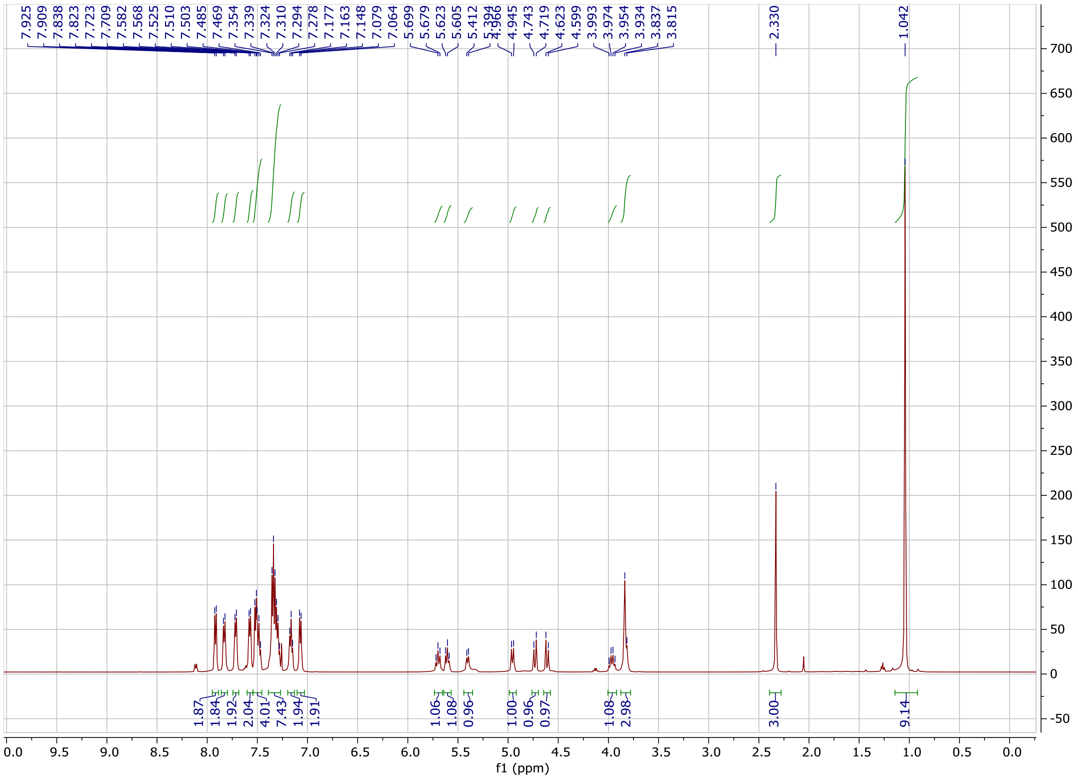

Supplementary Fig. 20. ^{13}C-NMR spectrum of compound **10**.

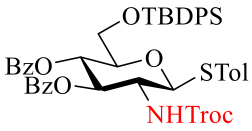

^{13}C-NMR ($CDCl_3$, 125 MHz) of **10**

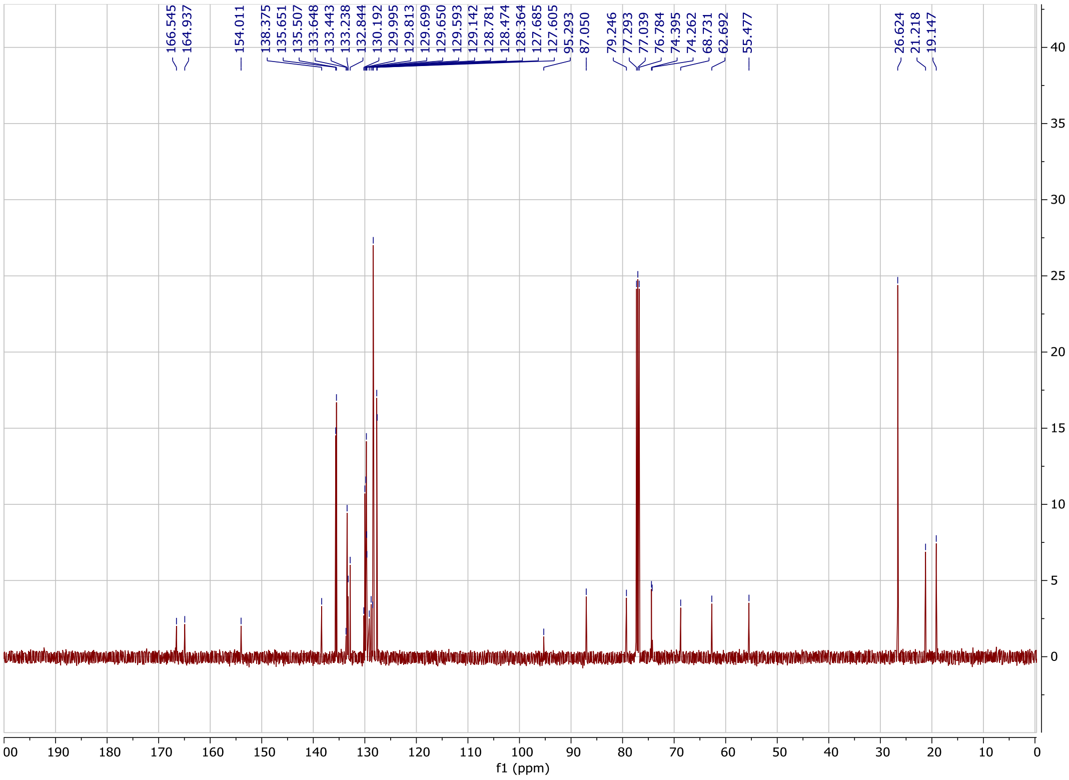

Supplementary Fig. 21. ^{1}H-NMR spectrum of compound **11**.

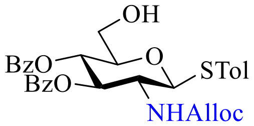

^{1}H-NMR ($CDCl_3$, 500 MHz) of **11**

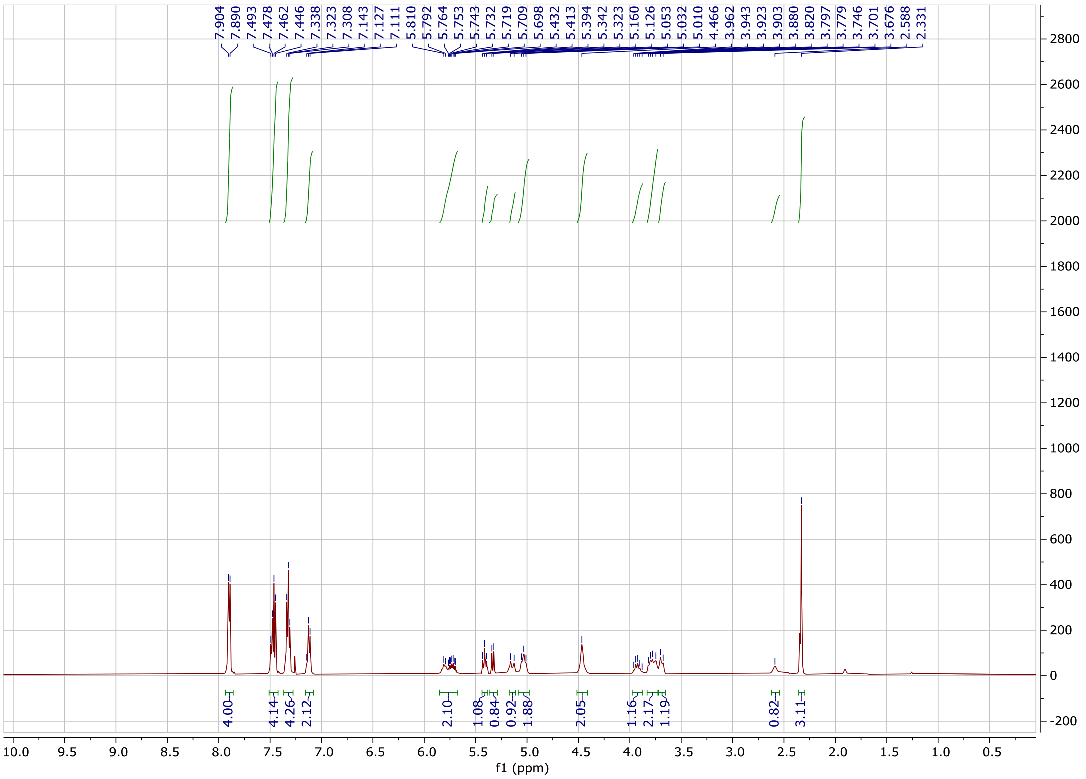

Supplementary Fig. 22. ^{13}C-NMR spectrum of compound **11**.

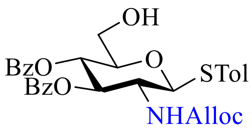

^{13}C-NMR ($CDCl_3$, 125 MHz) of **11**

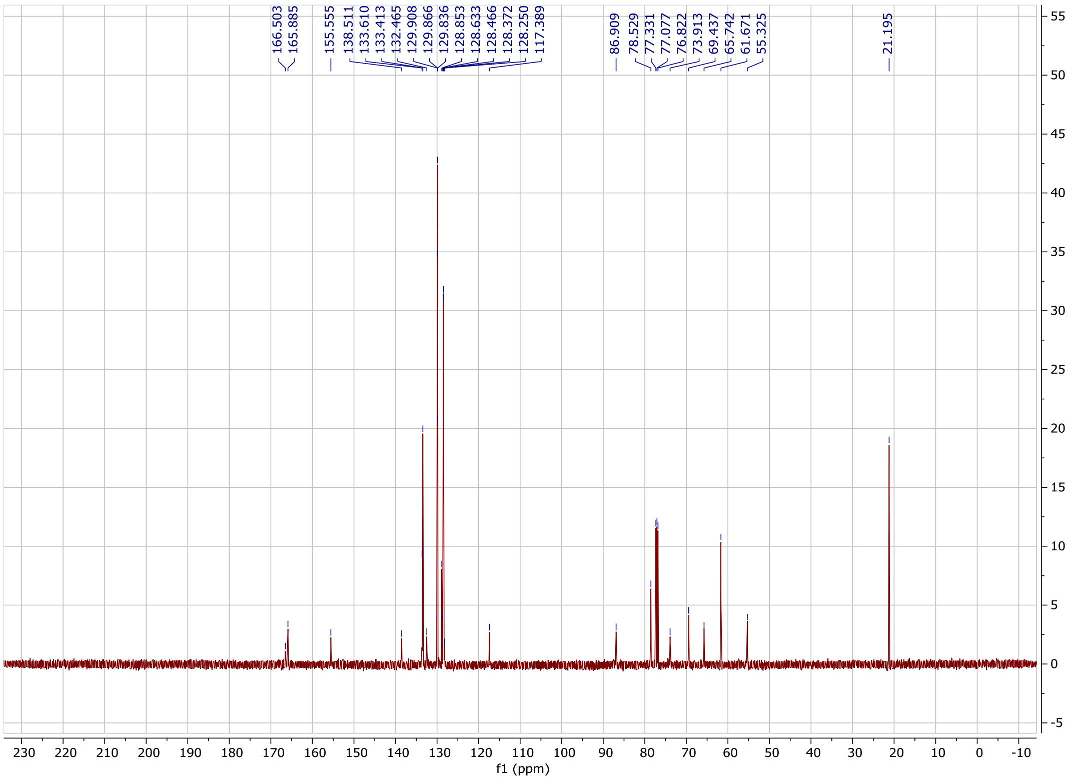

Supplementary Fig. 23. ^{1}H-NMR spectrum of compound **12**.

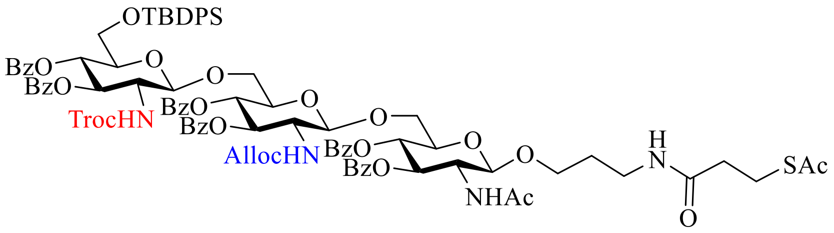

^{1}H-NMR (CDCl$_3$, 500 MHz) of **12**

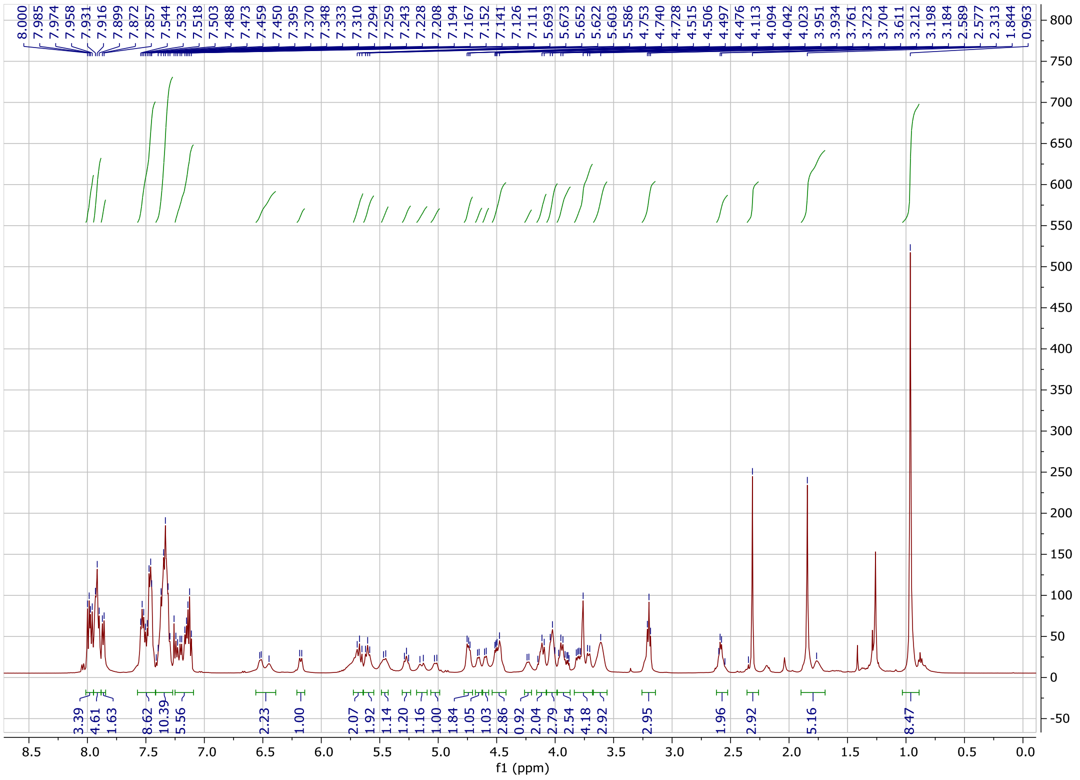

Supplementary Fig. 24. ^{13}C-NMR spectrum of compound **12**.

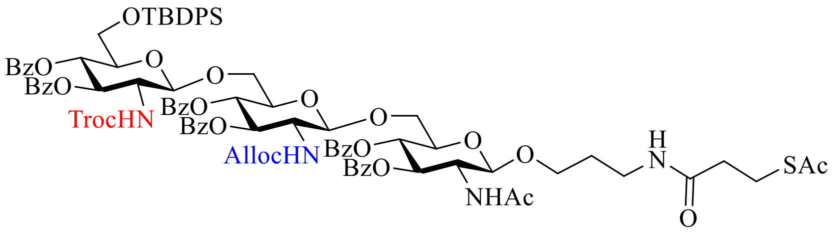

^{13}C-NMR ($CDCl_3$, 125 MHz) of **12**

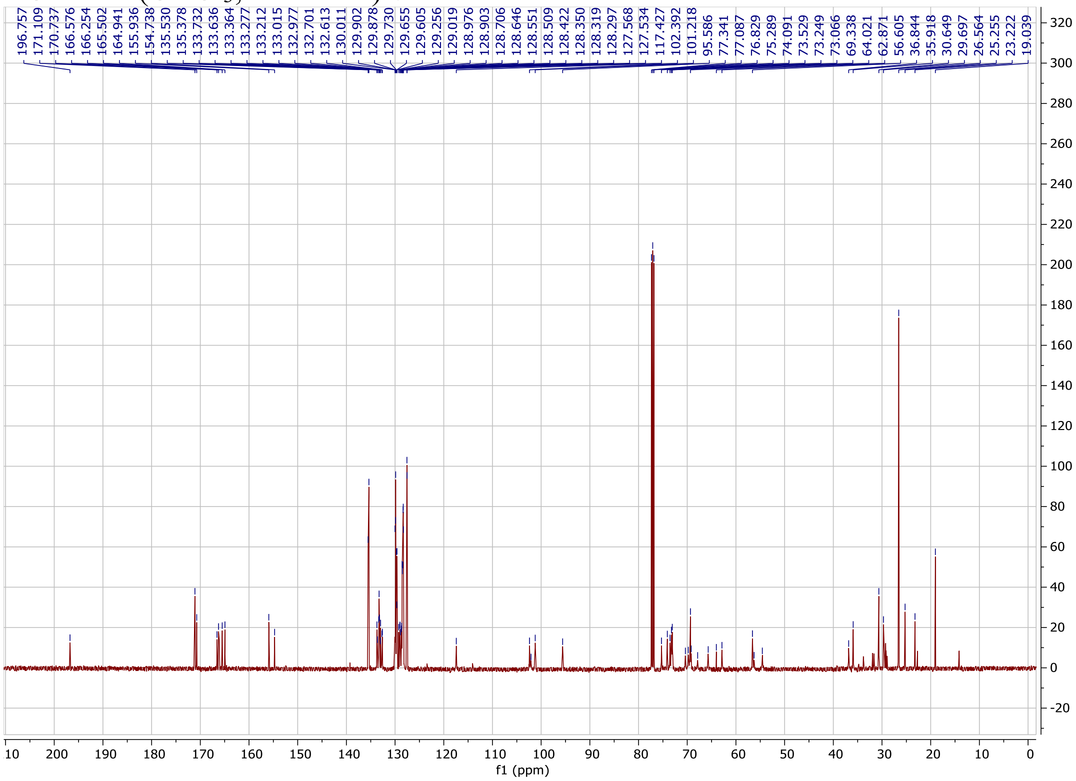

Supplementary Fig. 25. Coupled gHSQC spectrum of compound **12**.

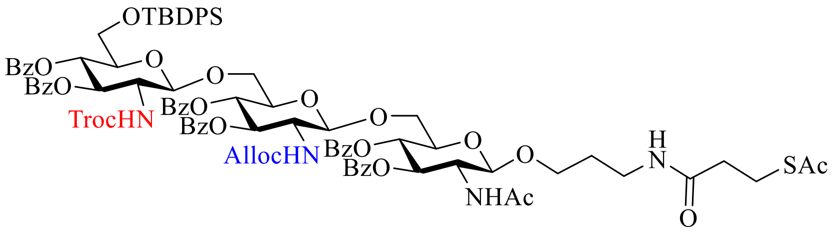

coupled gHSQC ($CDCl_3$, 500 MHz) of **12**

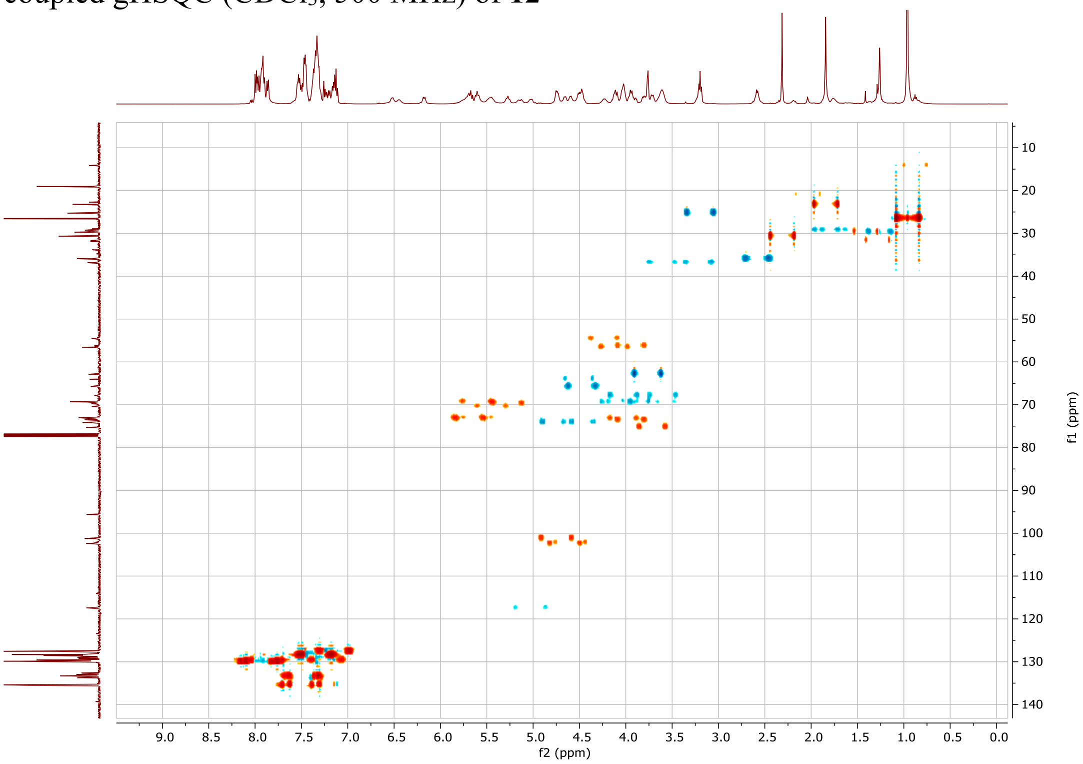

Supplementary Fig. 26. ^{1}H-NMR spectrum of compound **13**.

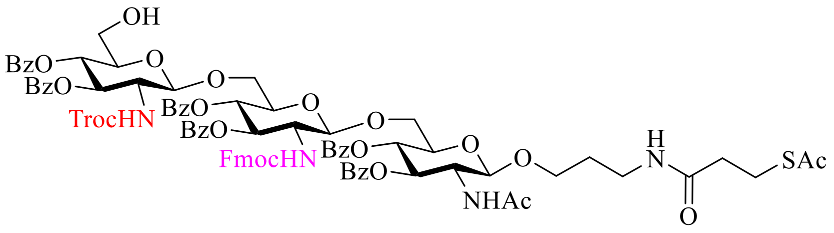

^{1}H-NMR ($CDCl_3$, 500 MHz) of **13**

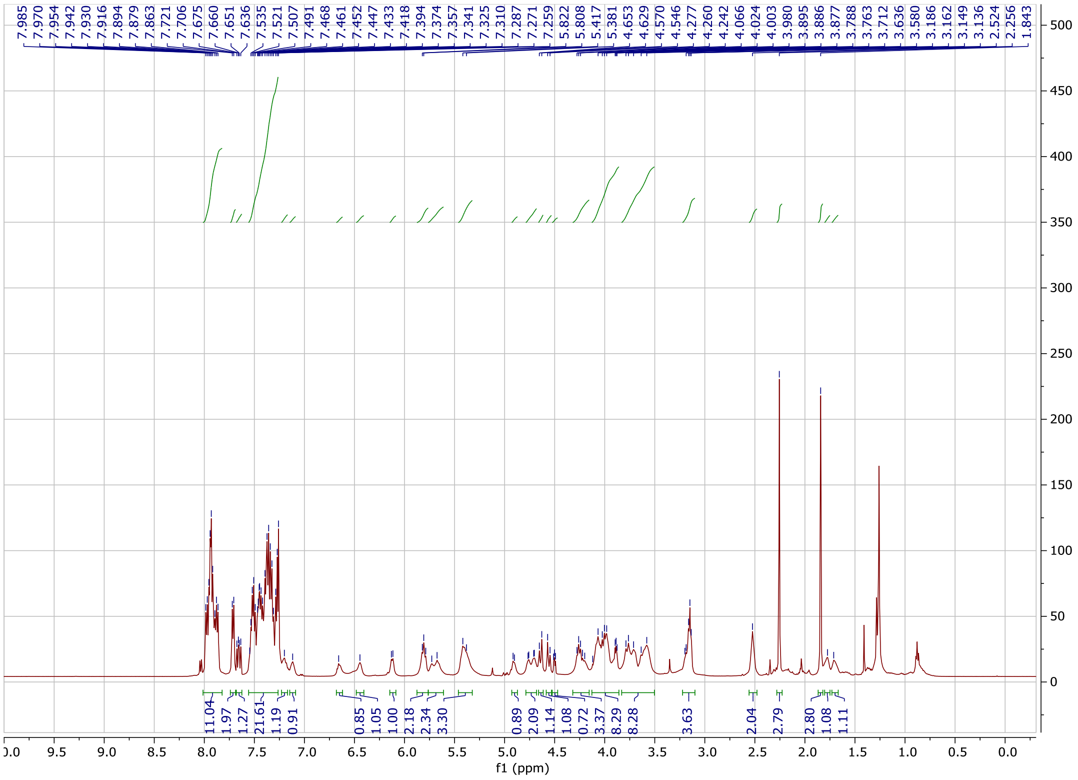

Supplementary Fig. 27. ^{13}C-NMR spectrum of compound **13**.

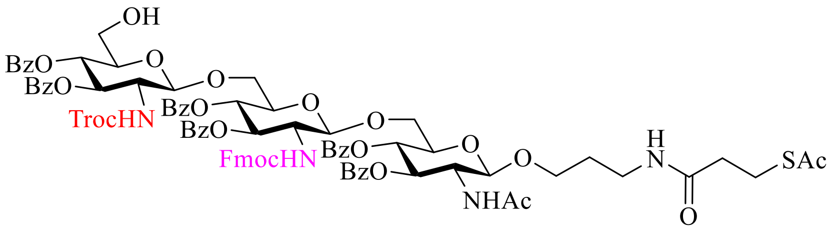

^{13}C-NMR ($CDCl_3$, 125 MHz) of **13**

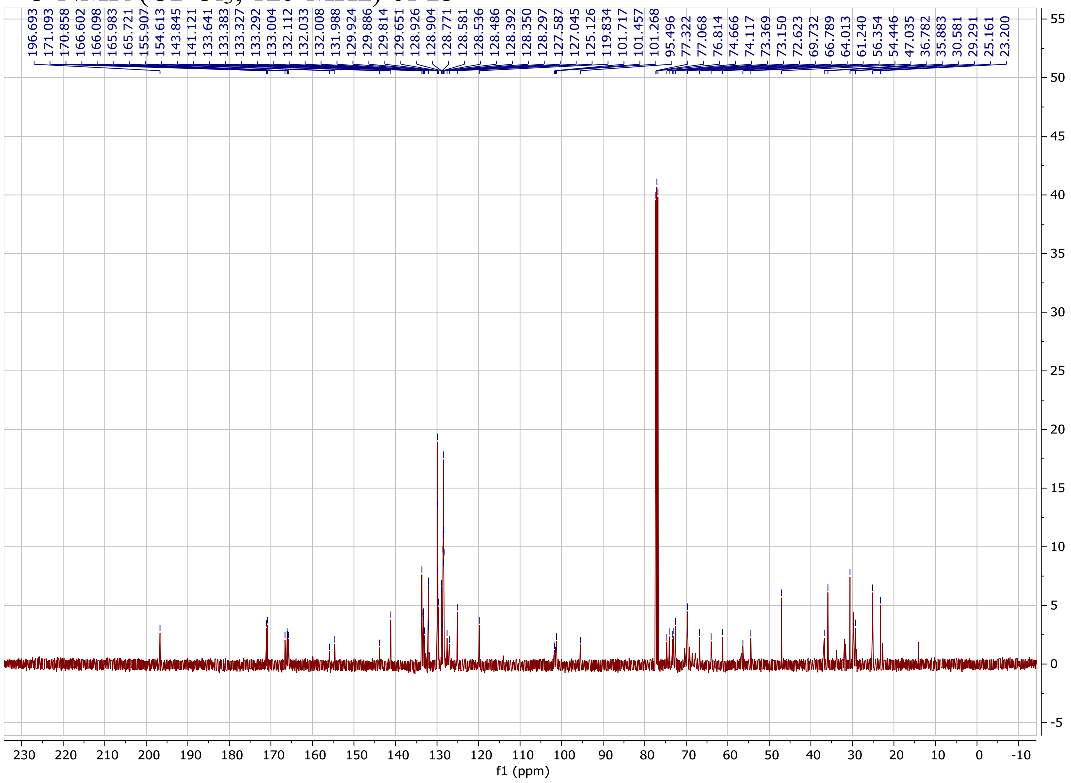

Supplementary Fig. 28. ^{1}H-NMR spectrum of compound **14**.

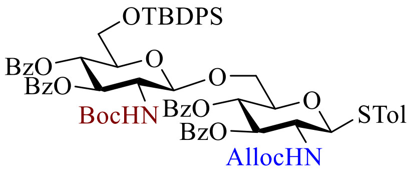

^{1}H-NMR ($CDCl_3$, 500 MHz) of **14**

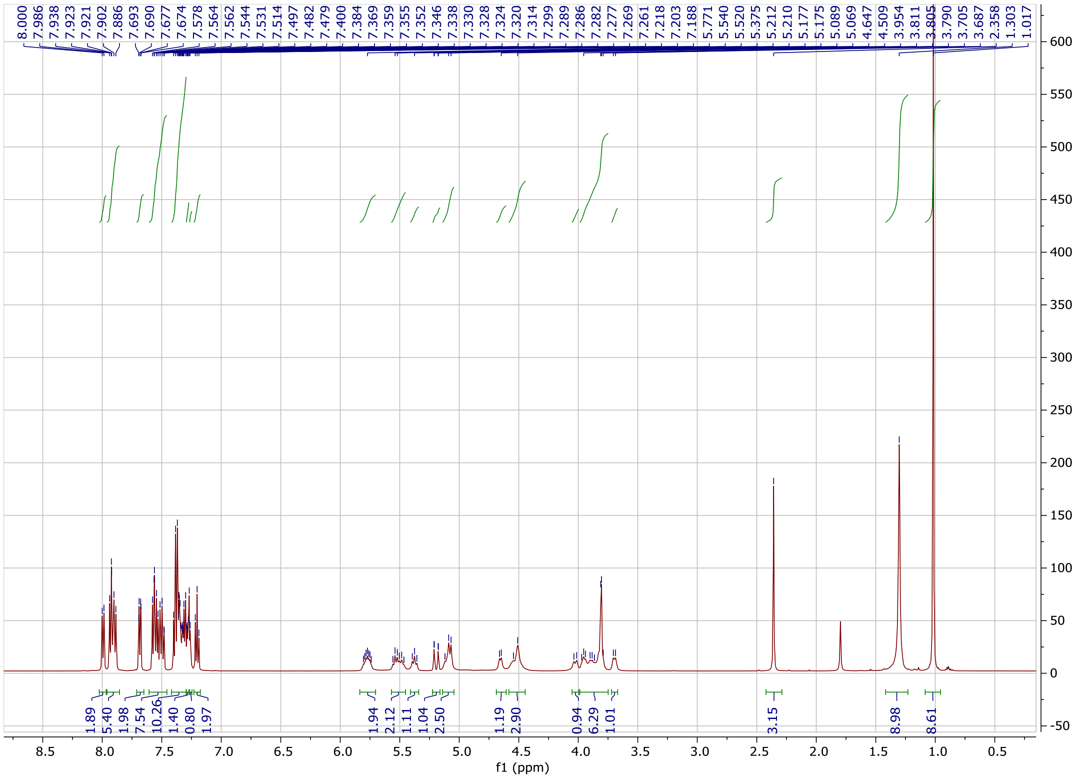

Supplementary Fig. 29. ^{13}C-NMR spectrum of compound **14**.

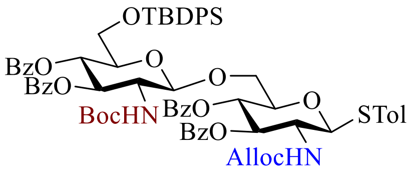

^{13}C-NMR ($CDCl_3$, 125 MHz) of **14**

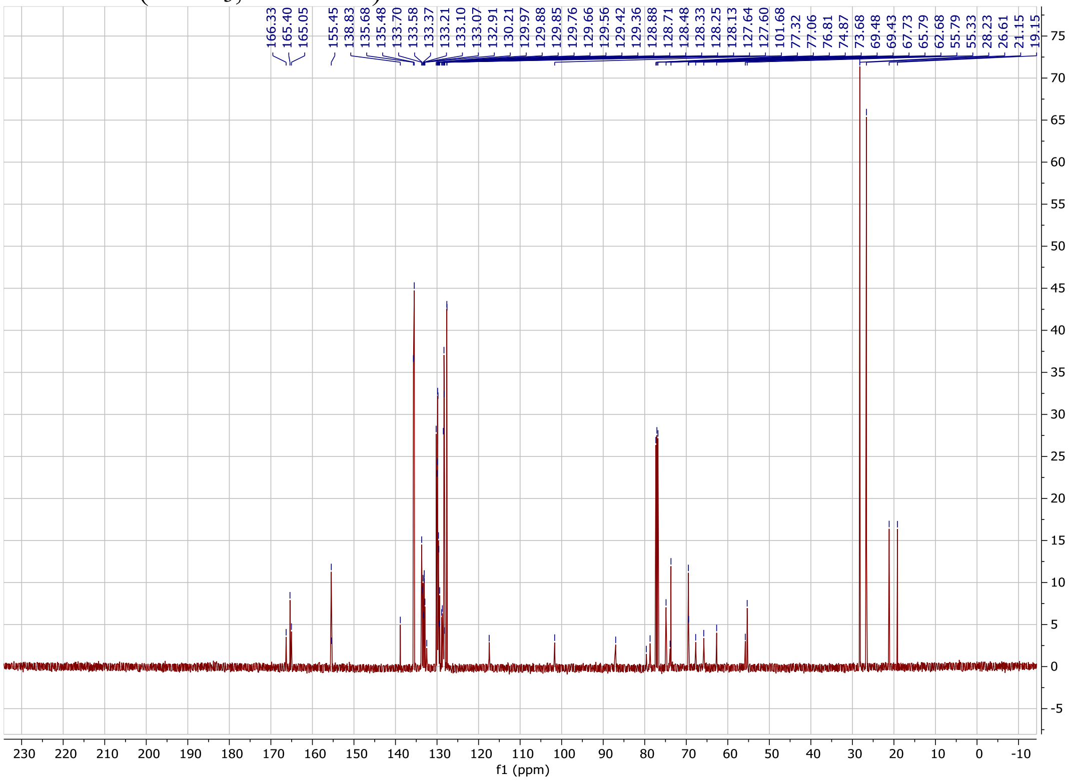

Supplementary Fig. 30. ^{1}H-NMR spectrum of compound **1**.

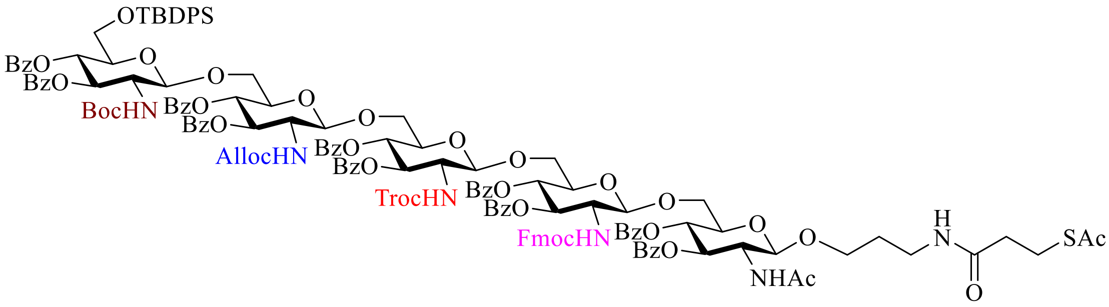

^{1}H-NMR ($CDCl_3$, 500 MHz) of **1**

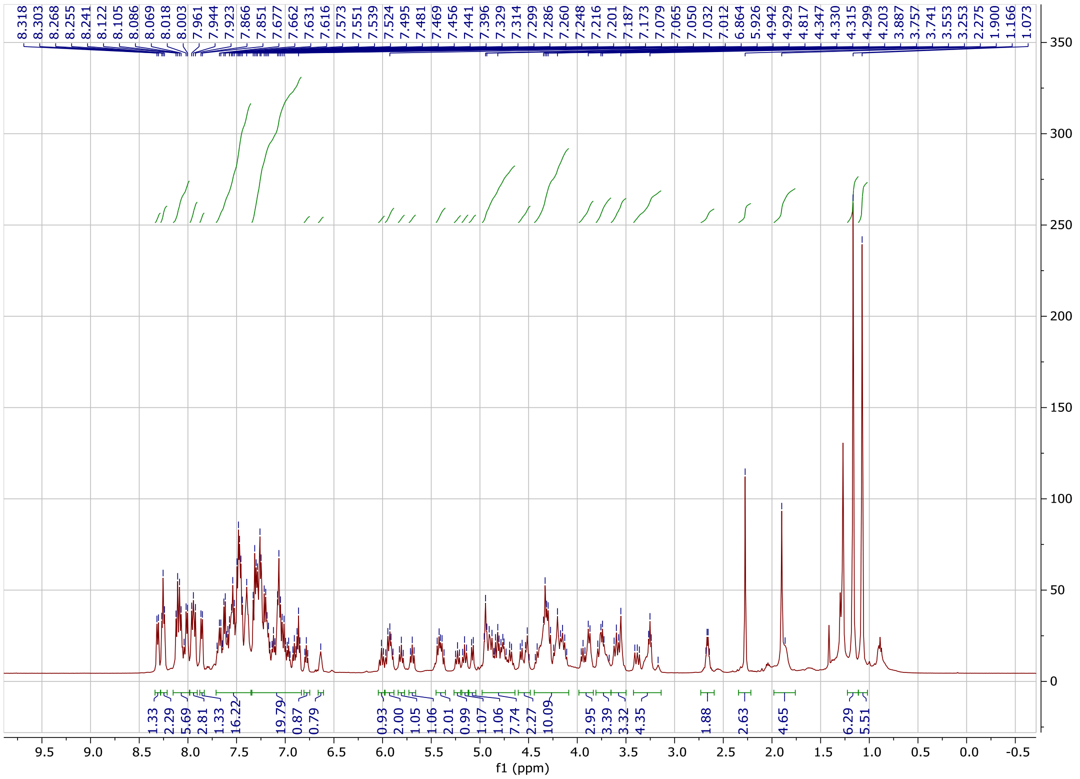

Supplementary Fig. 31. ^{13}C-NMR spectrum of compound **1**.

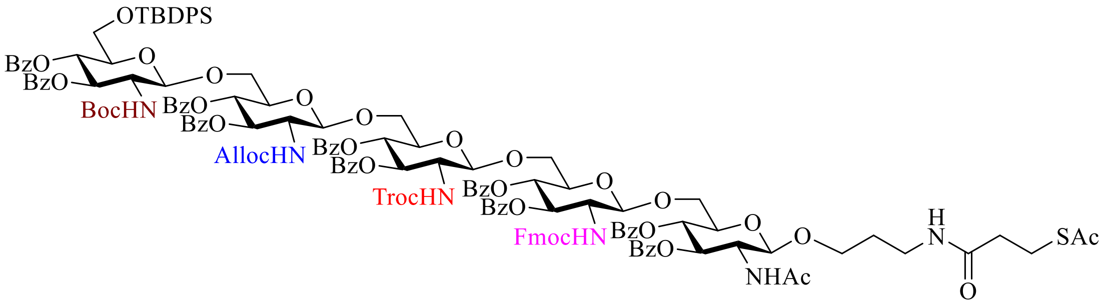

^{13}C-NMR (CDCl$_3$, 125 MHz) of **1**

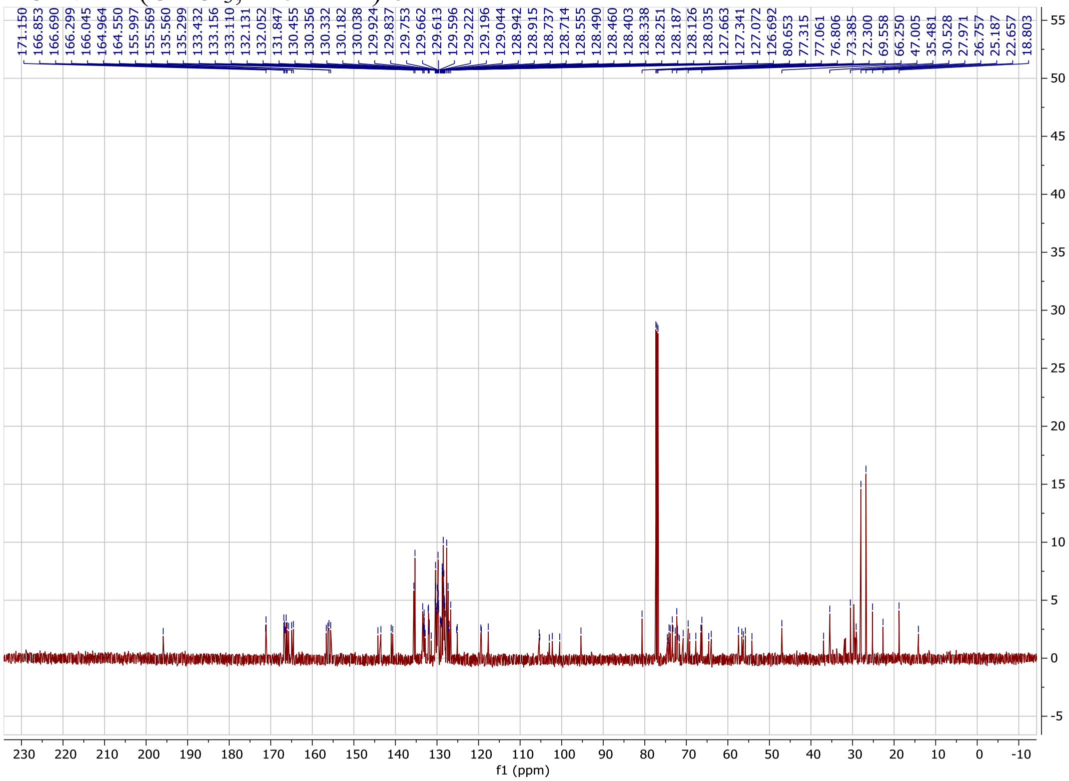

Supplementary Fig. 32. Coupled gHSQC spectrum of compound **1**.

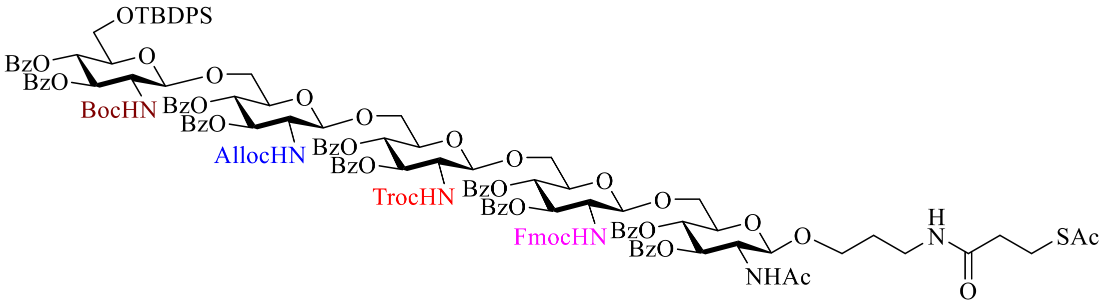

Coupled gHSQC ($CDCl_3$, 500 MHz) of **1**

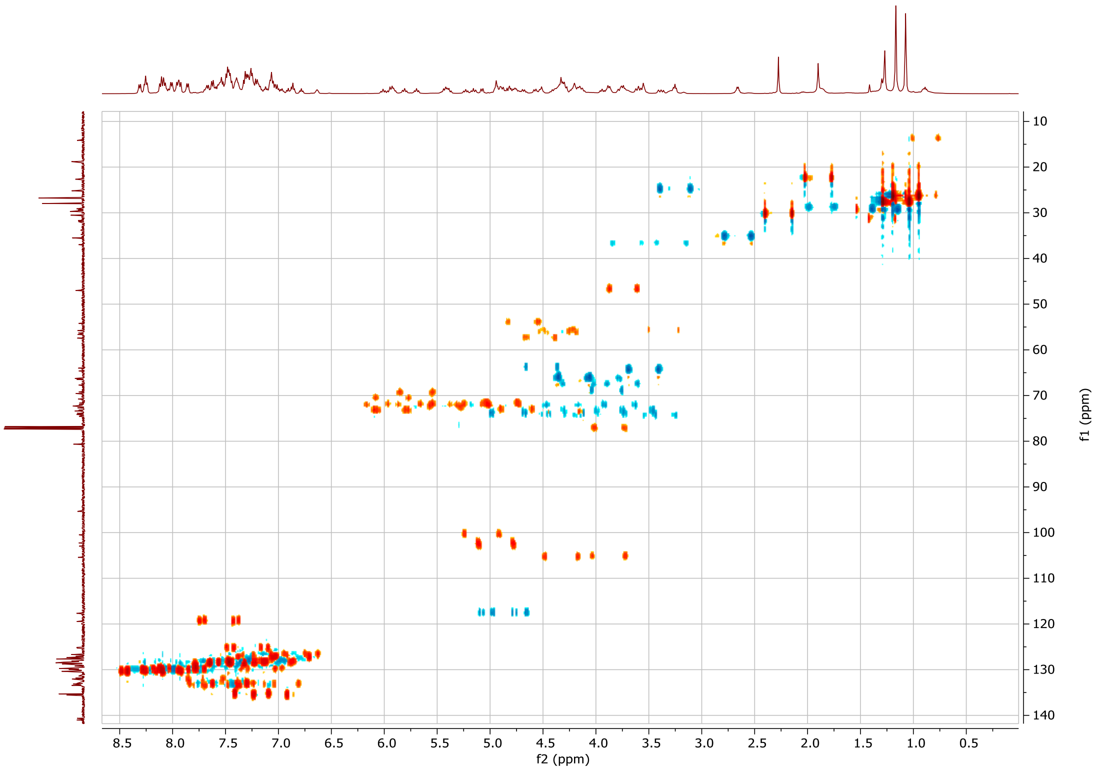

Supplementary Fig. 33. ^{1}H-NMR spectrum of compound **S3**.

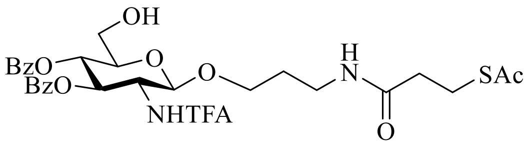

^{1}H-NMR ($CDCl_3$, 500 MHz) of **S3**

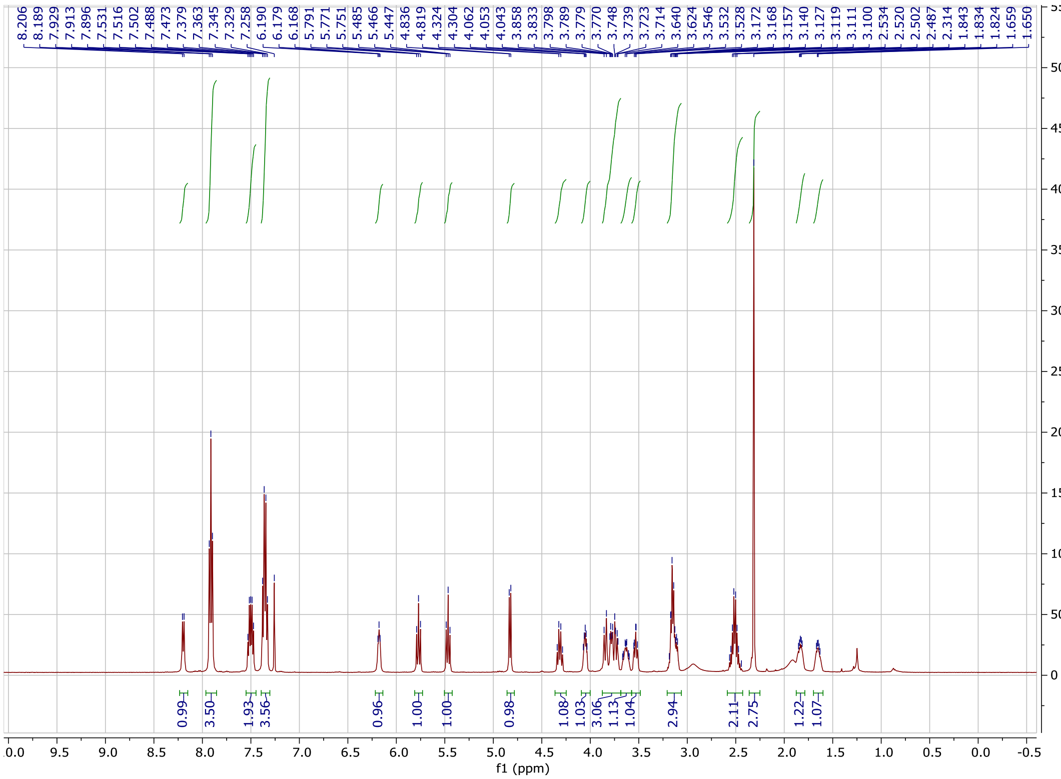

Supplementary Fig. 34. ^{13}C-NMR spectrum of compound **S3**.

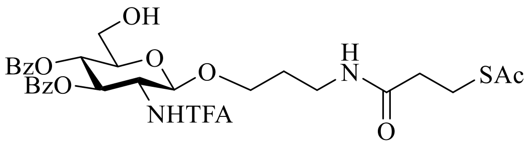

^{13}C-NMR ($CDCl_3$, 125 MHz) of **S3**

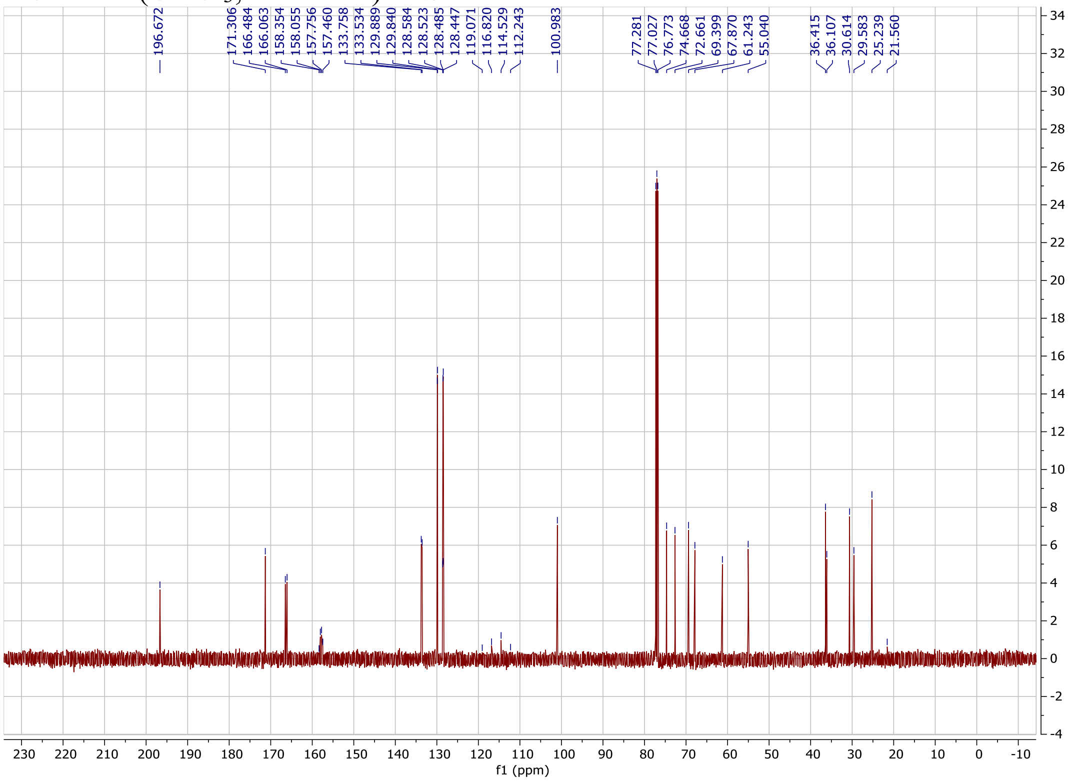

Supplementary Fig. 35. ^{1}H-NMR spectrum of compound **S4**.

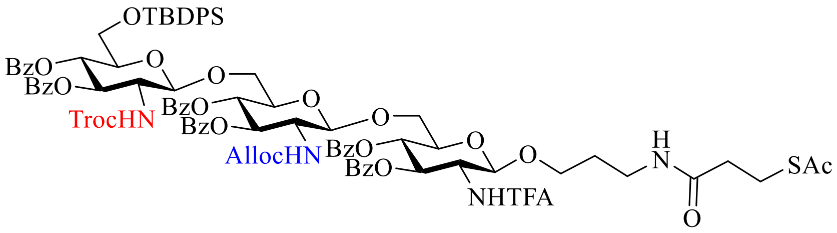

^{1}H-NMR ($CDCl_3$, 500 MHz) of **S4**

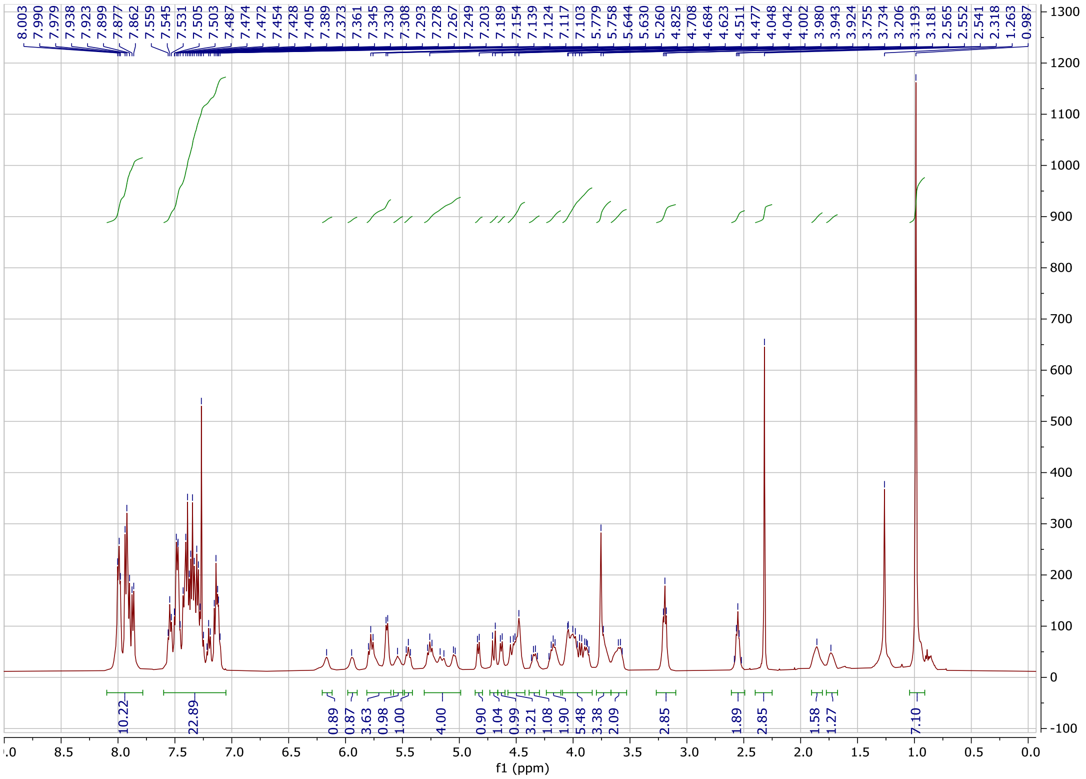

Supplementary Fig. 36. ^{13}C-NMR spectrum of compound **S4**.

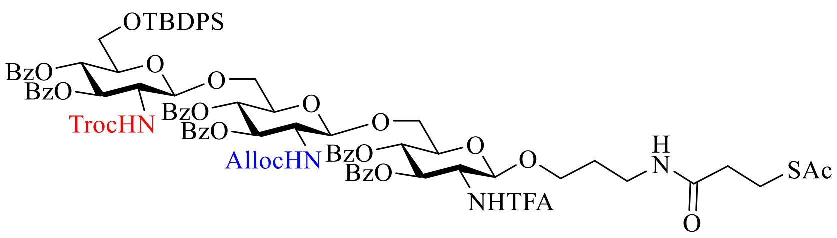

^{13}C-NMR ($CDCl_3$, 125 MHz) of **S4**

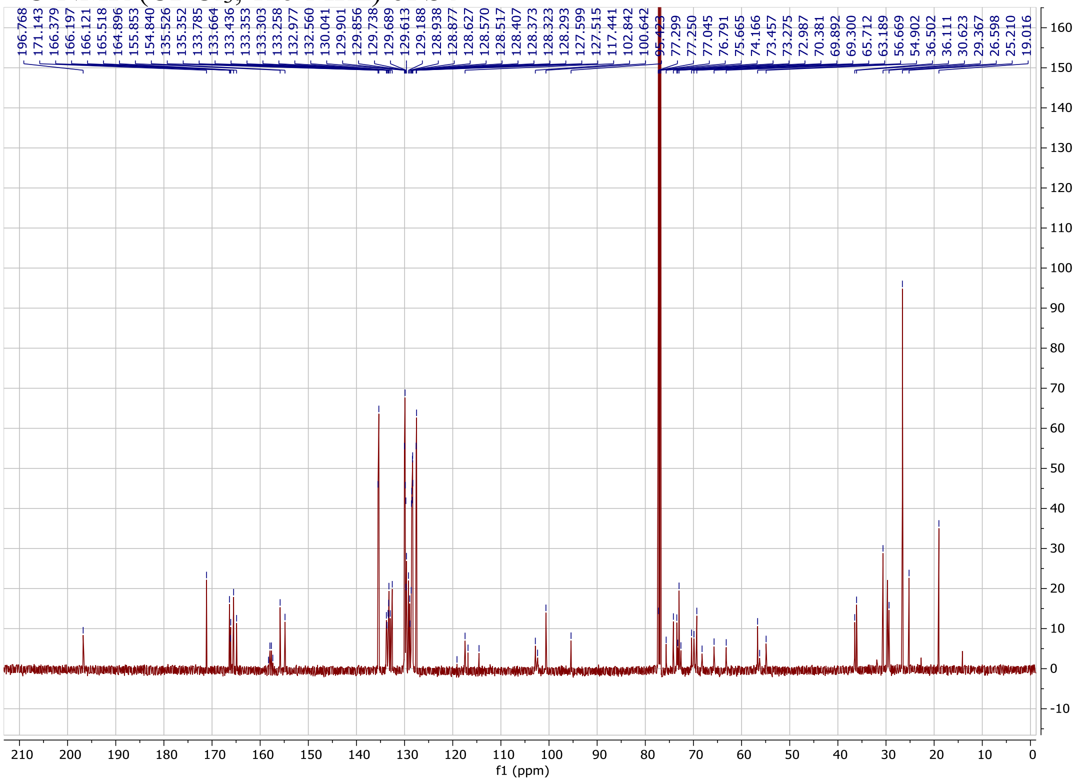

Supplementary Fig. 37. gHSQC spectrum of compound **S4**.

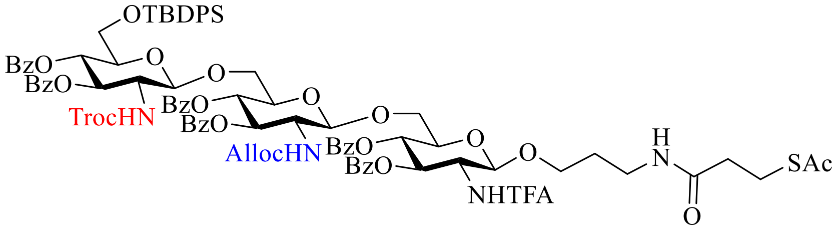

gHSQC ($CDCl_3$, 500 MHz) of **S4**

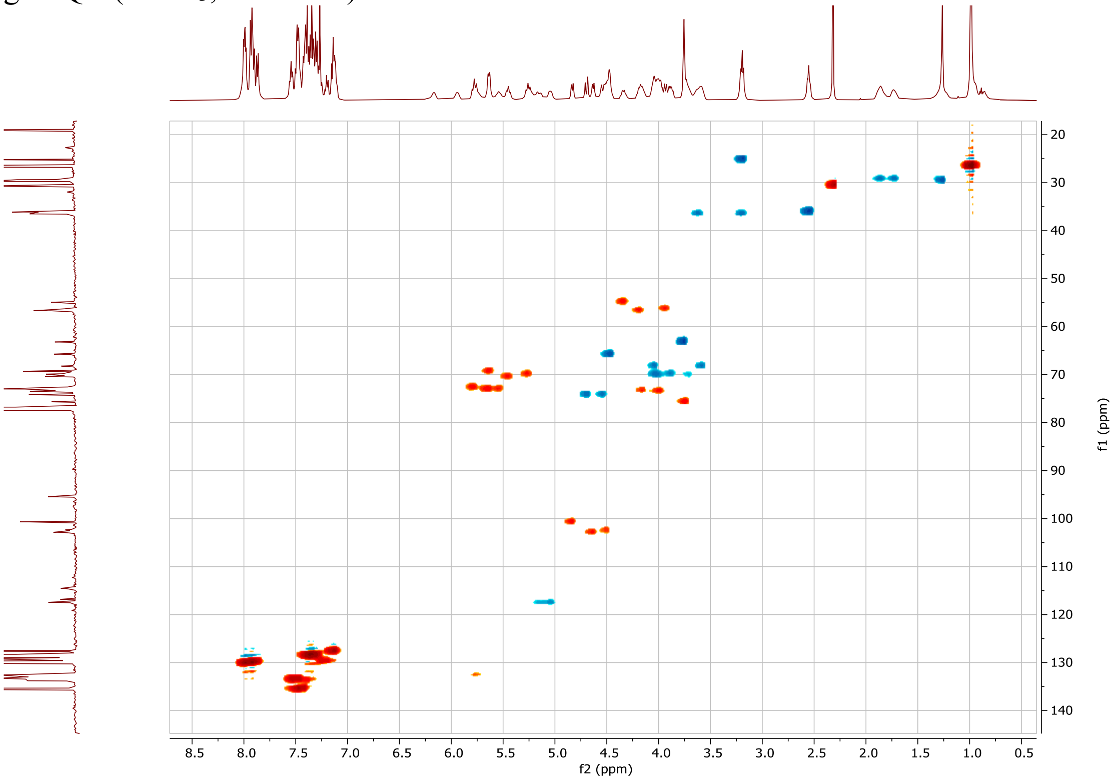

Supplementary Fig. 38. ^{1}H-NMR spectrum of compound **S5**.

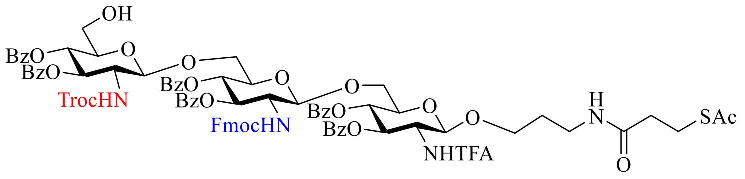

^{1}H-NMR ($CDCl_3$, 500 MHz) of **S5**

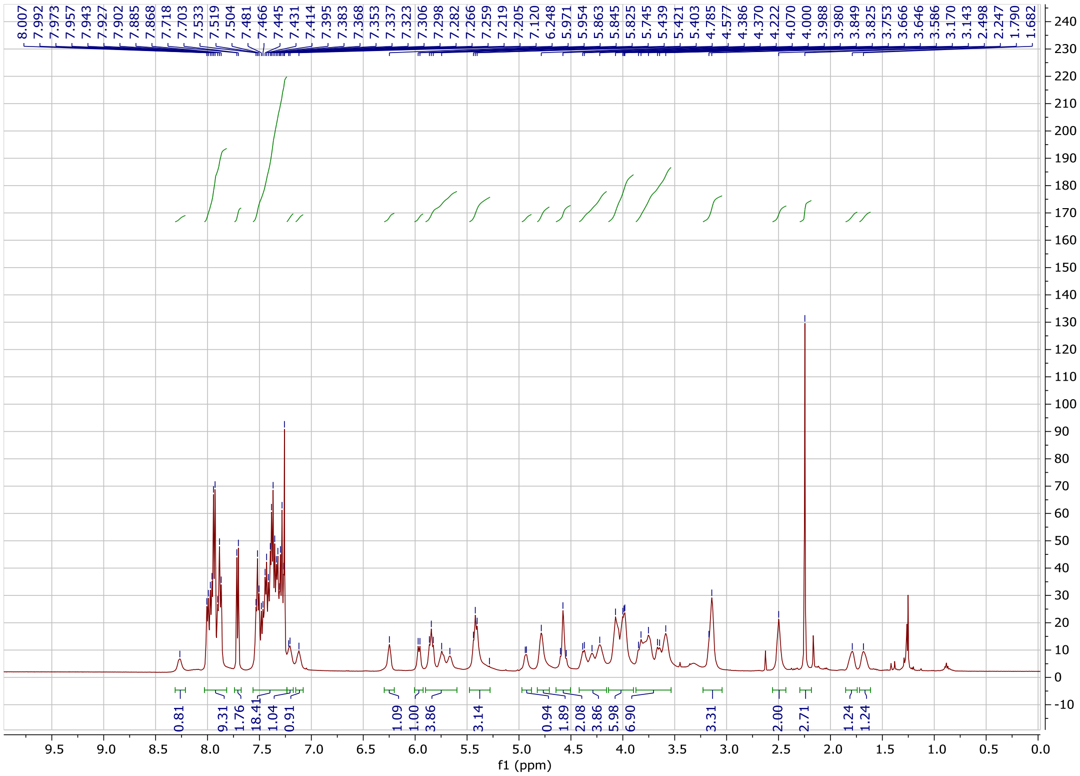

Supplementary Fig. 39. ^{13}C-NMR spectrum of compound **S5**.

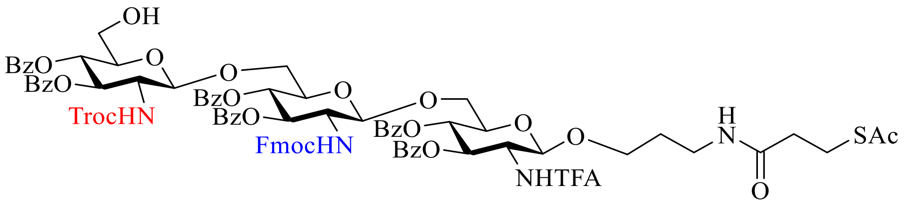

^{13}C-NMR (CDCl$_3$, 125 MHz) of **S5**

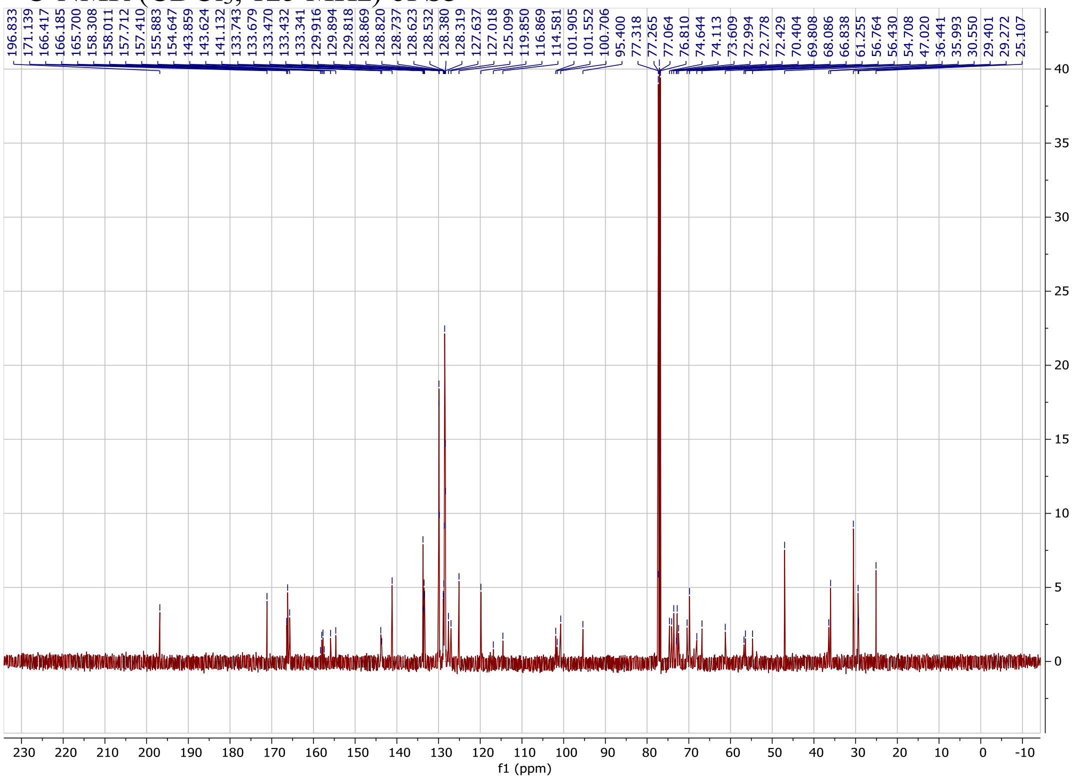

Supplementary Fig. 40. ^{1}H-NMR spectrum of compound **2**.

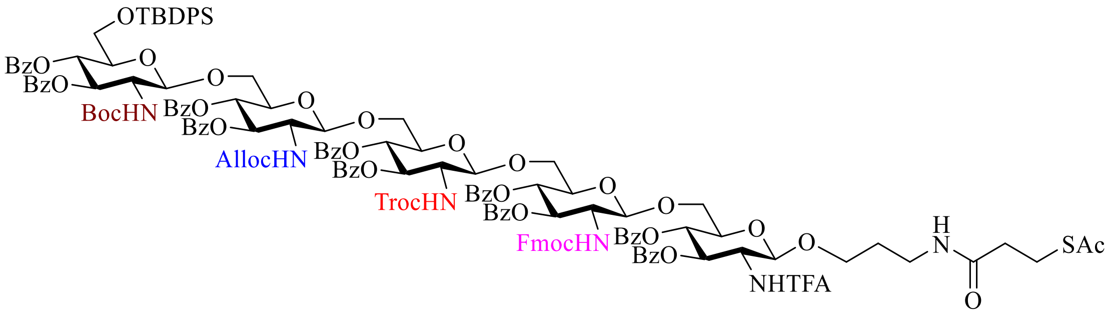

^{1}H-NMR ($CDCl_3$, 500 MHz) of **2**

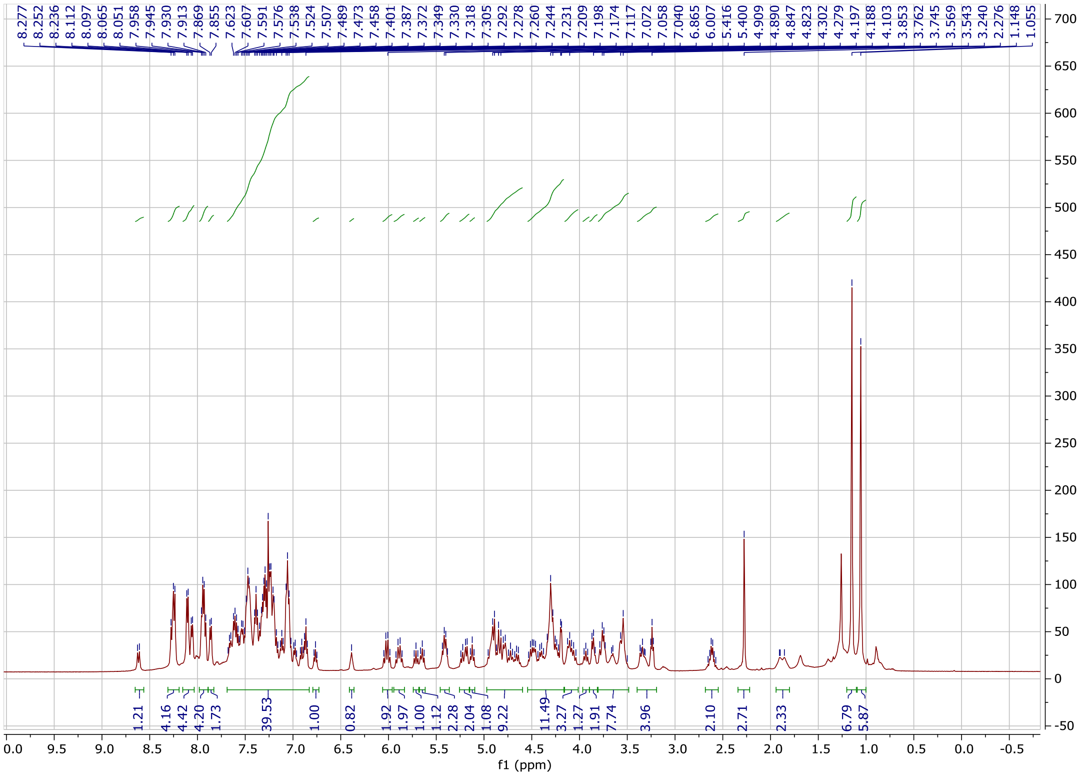

Supplementary Fig. 41. ^{13}C-NMR spectrum of compound **2**.

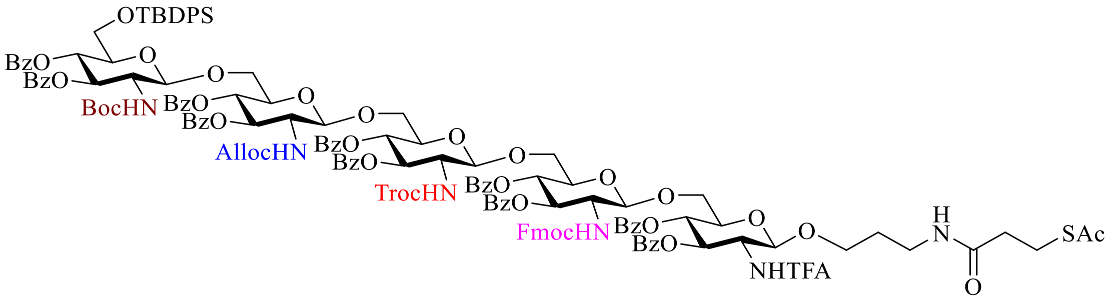

^{13}C-NMR ($CDCl_3$, 125 MHz) of **2**

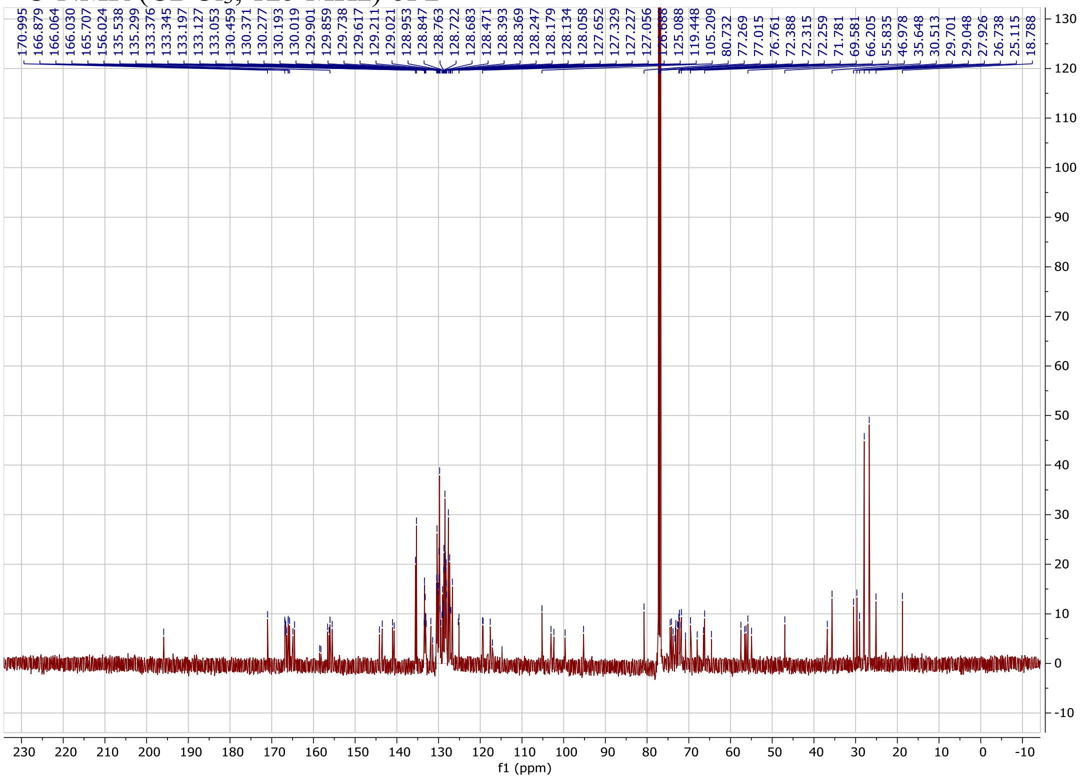

Supplementary Fig. 42. Coupled gHSQC spectrum of compound **2**.

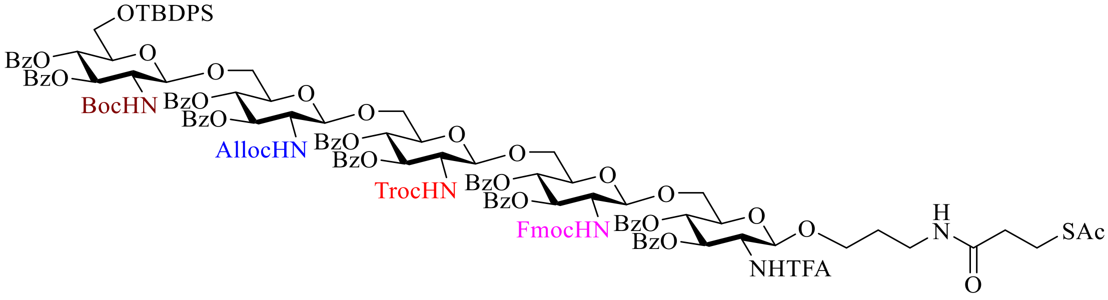

Coupled gHSQC ($CDCl_3$, 500 MHz) of **2**

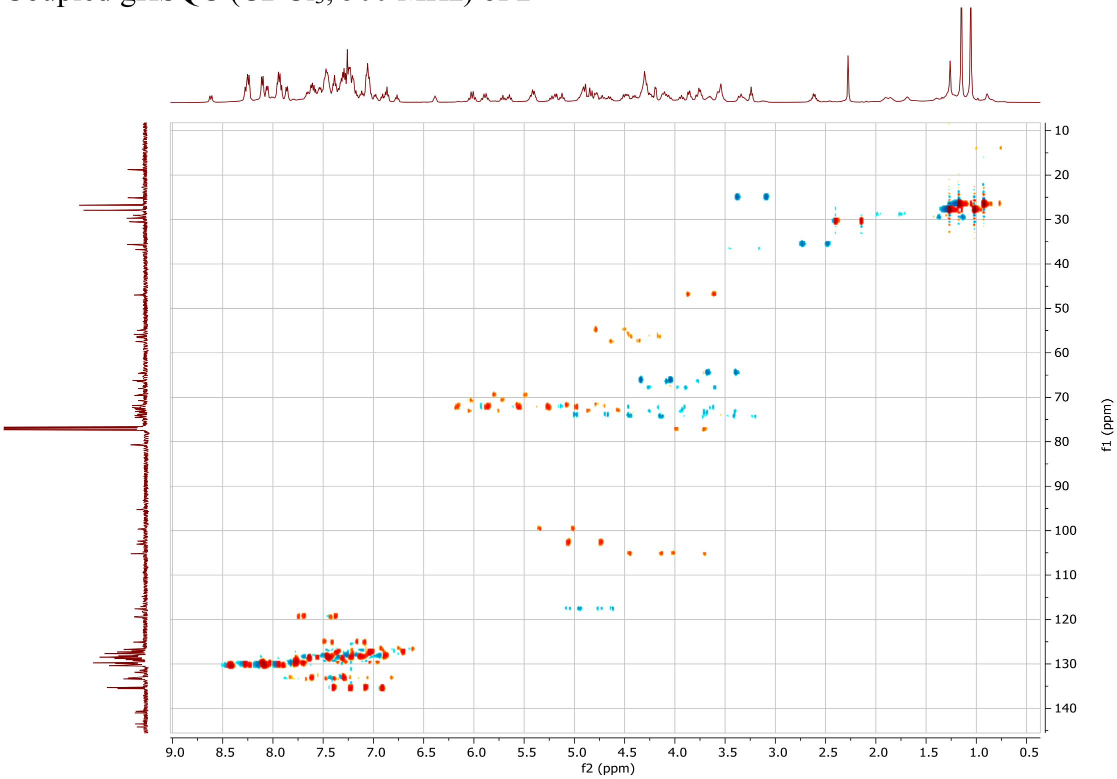

Supplementary Fig. 43. ^{1}H-NMR spectrum of compound **15**.

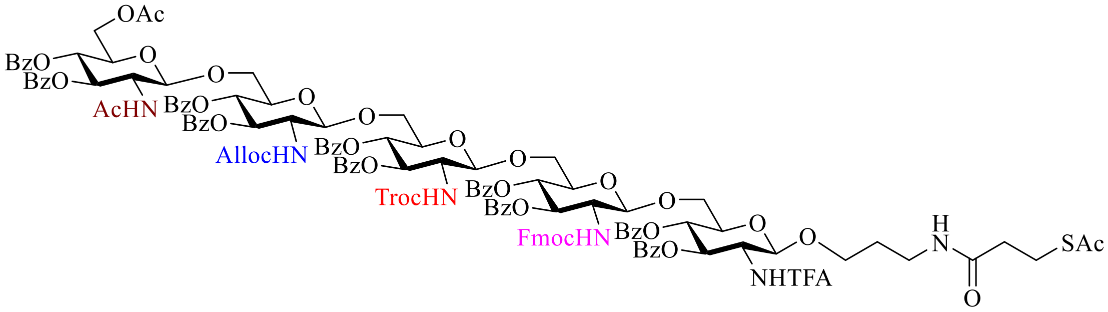

^{1}H-NMR ($CDCl_3$, 500 MHz) of **15**

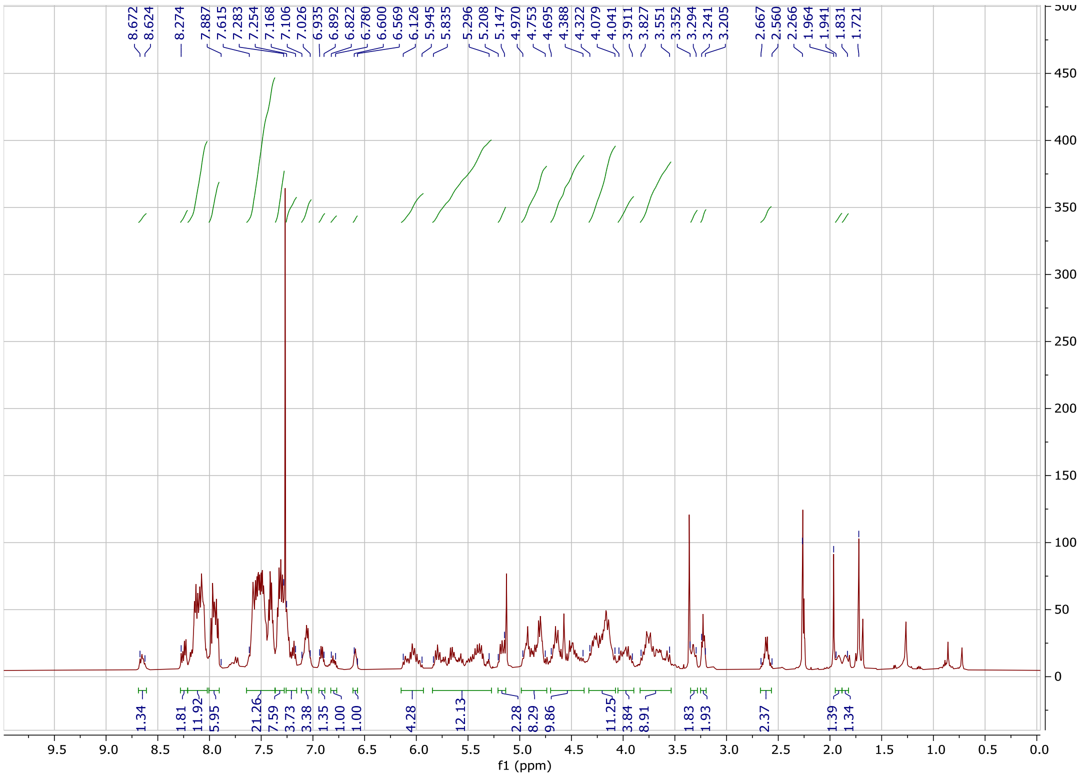

Supplementary Fig. 44. ^{13}C-NMR spectrum of compound **15**.

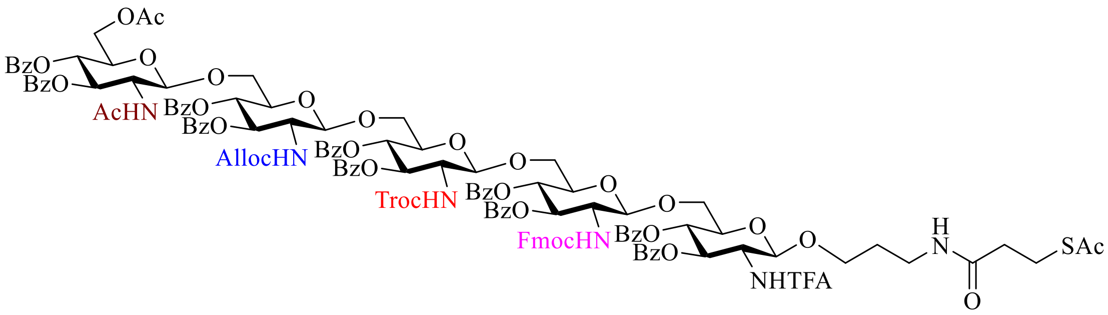

^{13}C-NMR ($CDCl_3$, 125 MHz) of **15**

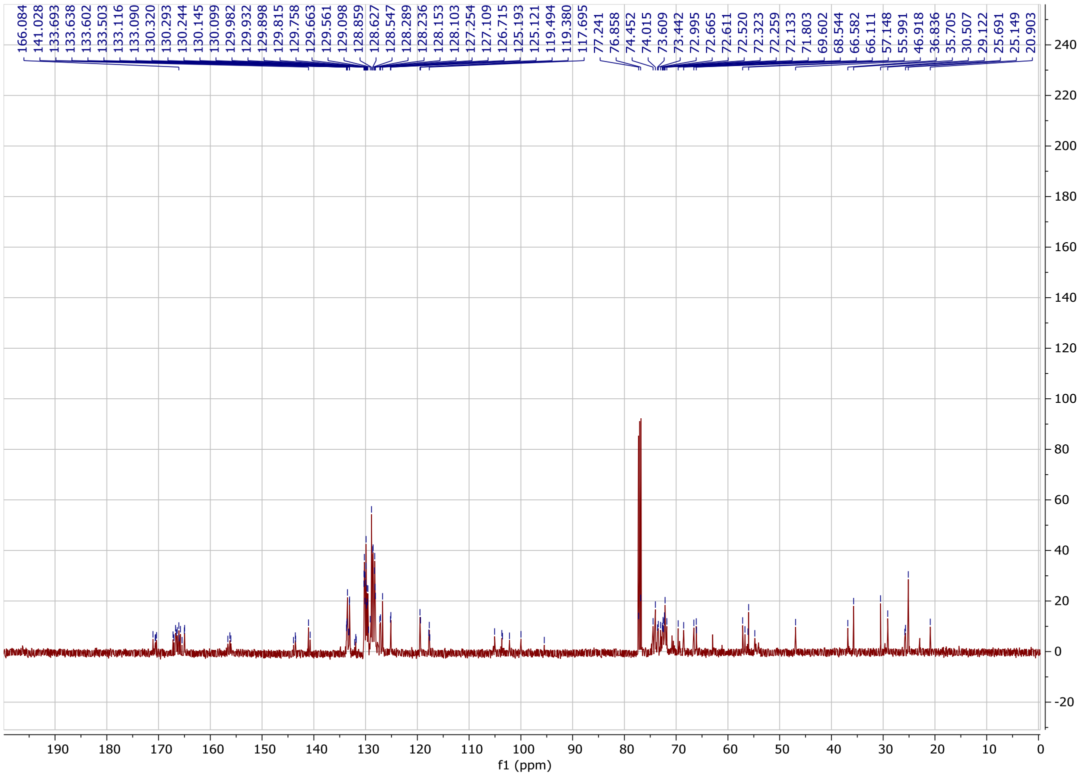

Supplementary Fig. 45. gHSQC spectrum of compound **15**.

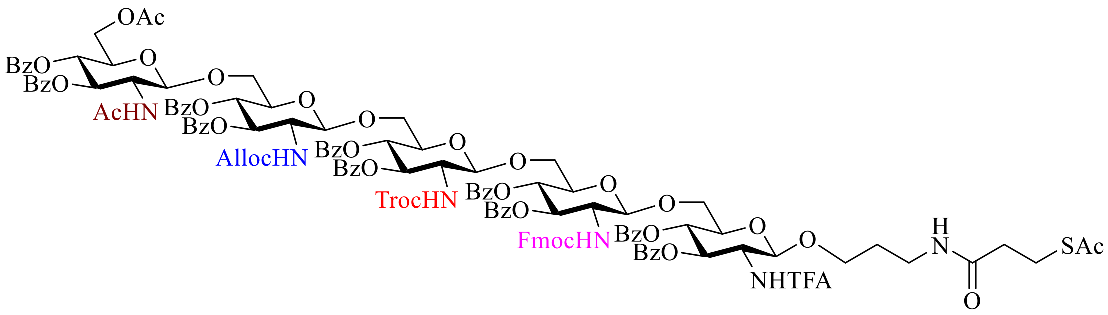

gHSQC ($CDCl_3$, 500 MHz) of **15**

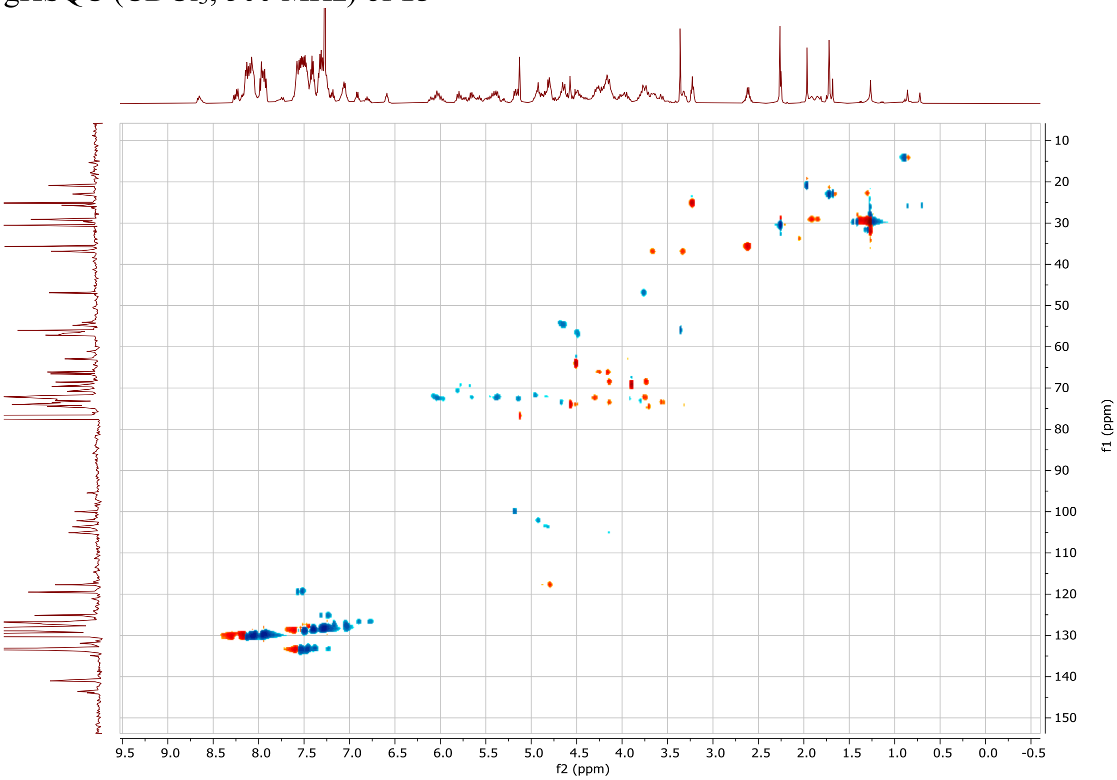

Supplementary Fig. 46. ^{1}H-NMR spectrum of compound **16**.

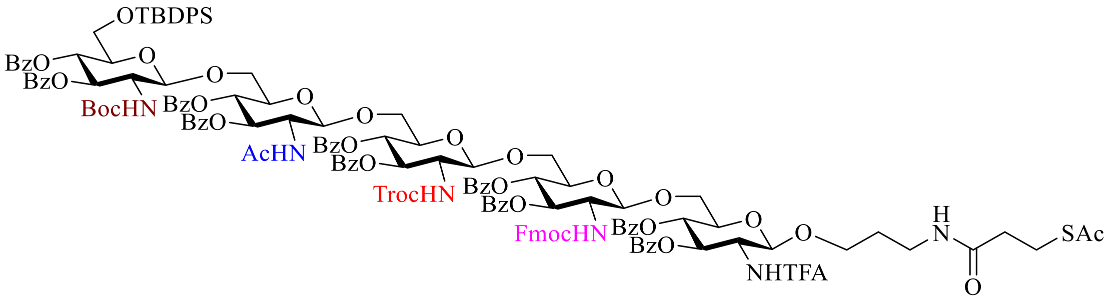

^{1}H-NMR ($CDCl_3$, 500 MHz) of **16**

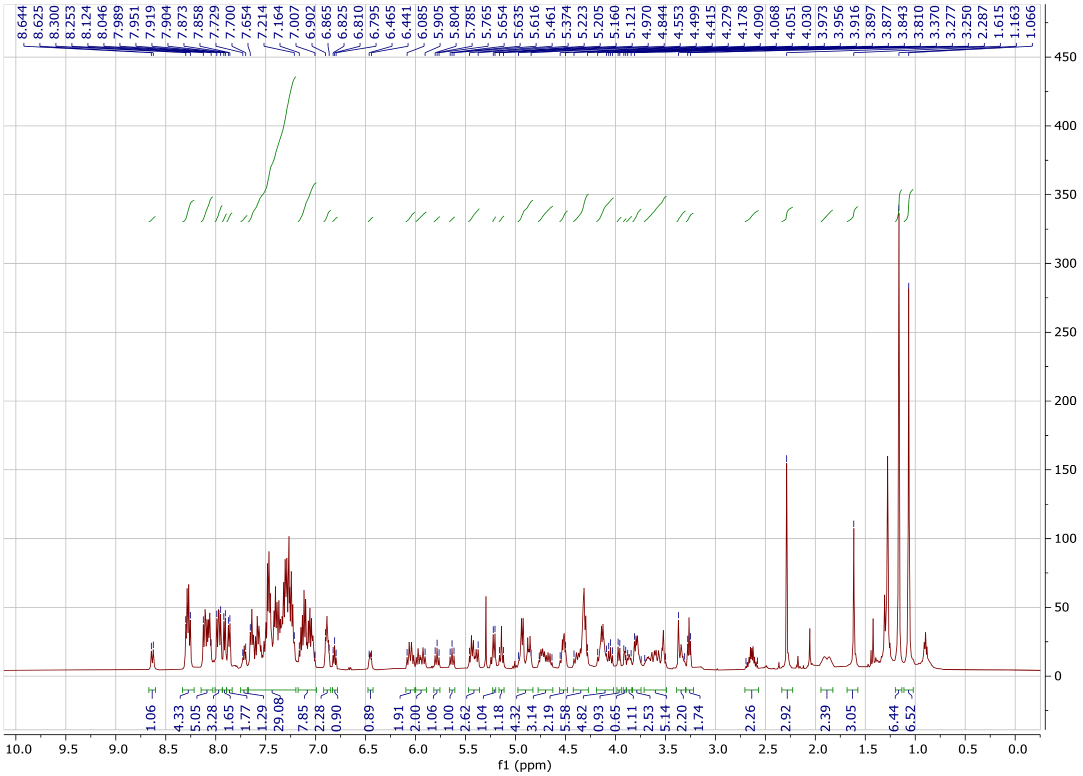

Supplementary Fig. 47. ^{13}C-NMR spectrum of compound **16**.

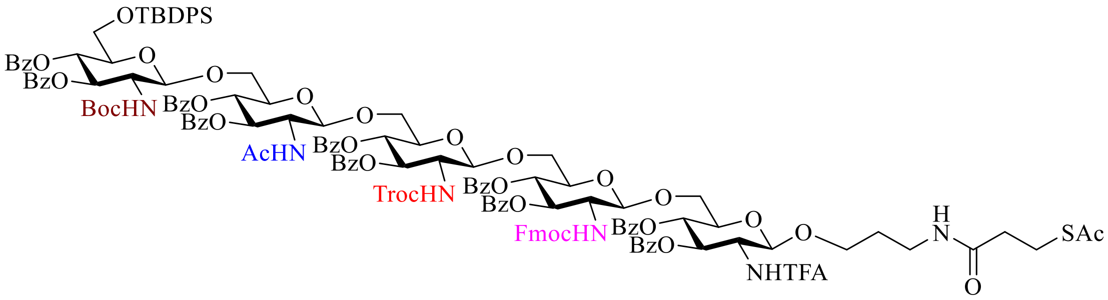

^{13}C-NMR (CDCl$_3$, 125 MHz) of **16**

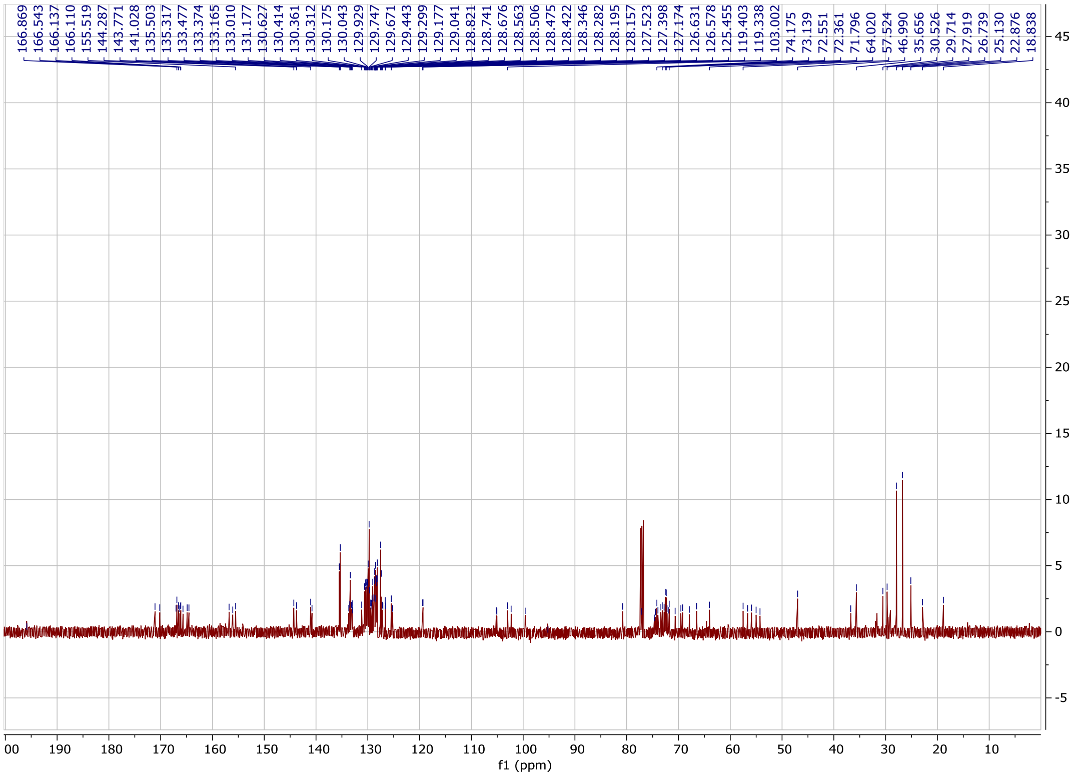

Supplementary Fig. 48. ^{1}H-NMR spectrum of compound **17**.

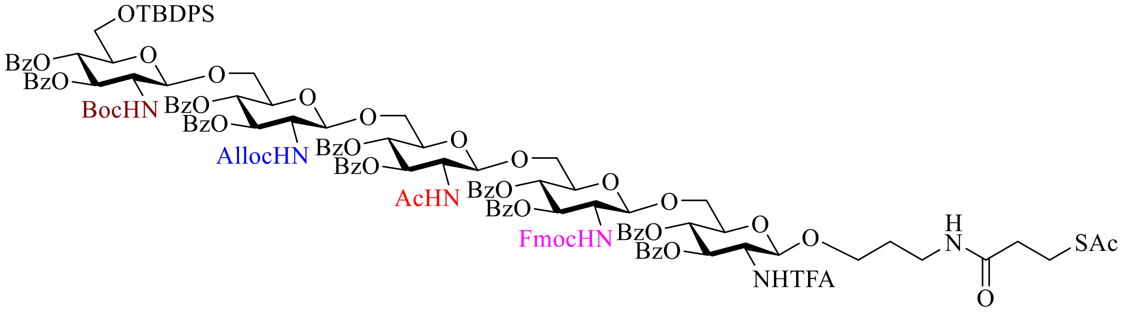

^{1}H-NMR ($CDCl_3$, 500 MHz) of **17**

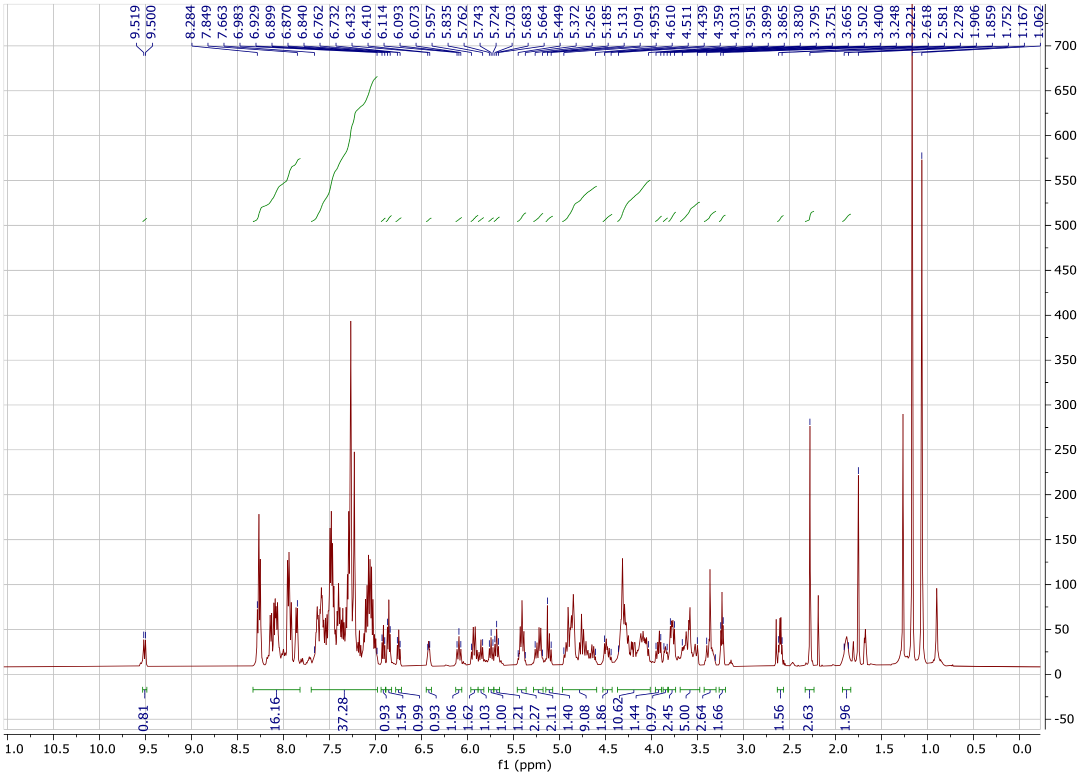

Supplementary Fig. 49. ^{13}C-NMR spectrum of compound **17**.

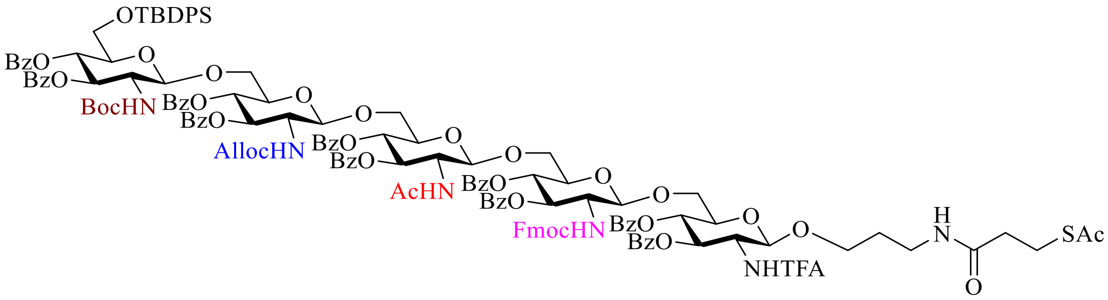

^{13}C-NMR ($CDCl_3$, 125 MHz) of **17**

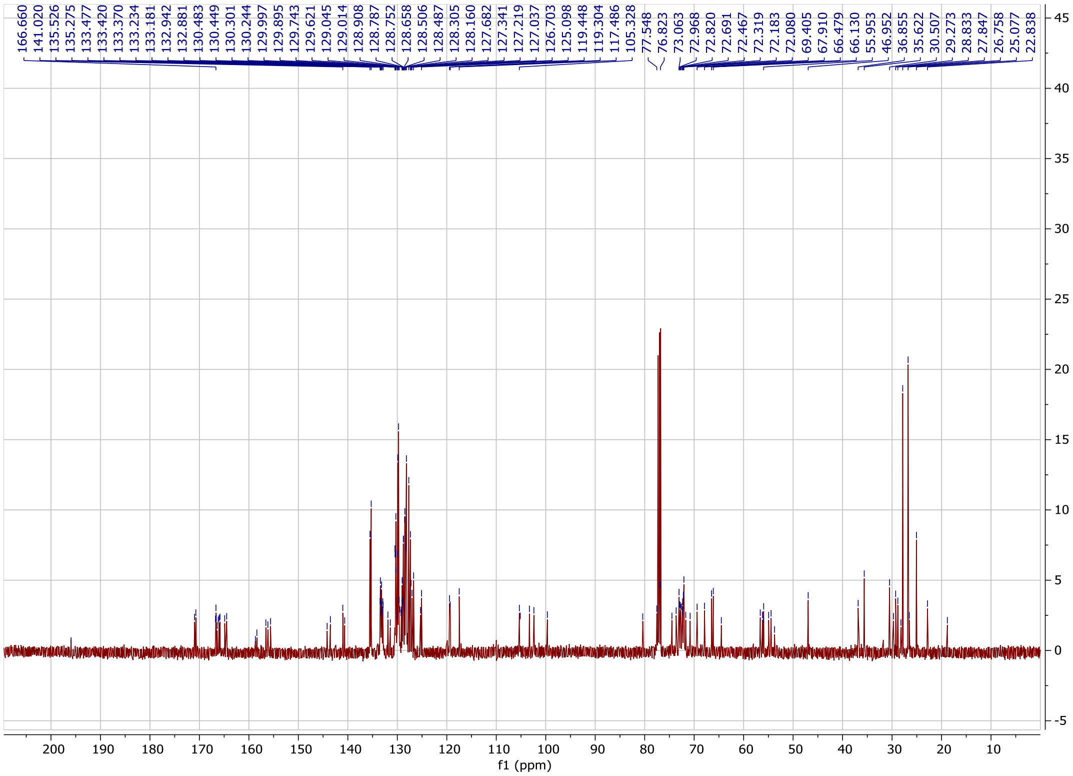

Supplementary Fig. 50. gHSQC spectrum of compound **17**.

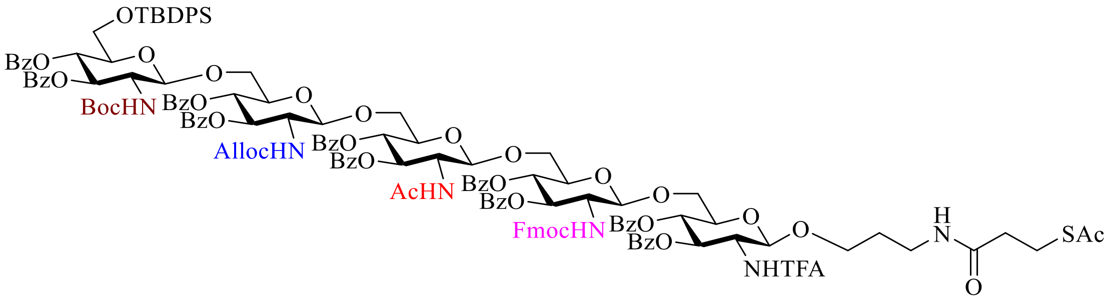

gHSQC ($CDCl_3$, 500 MHz) of **17**

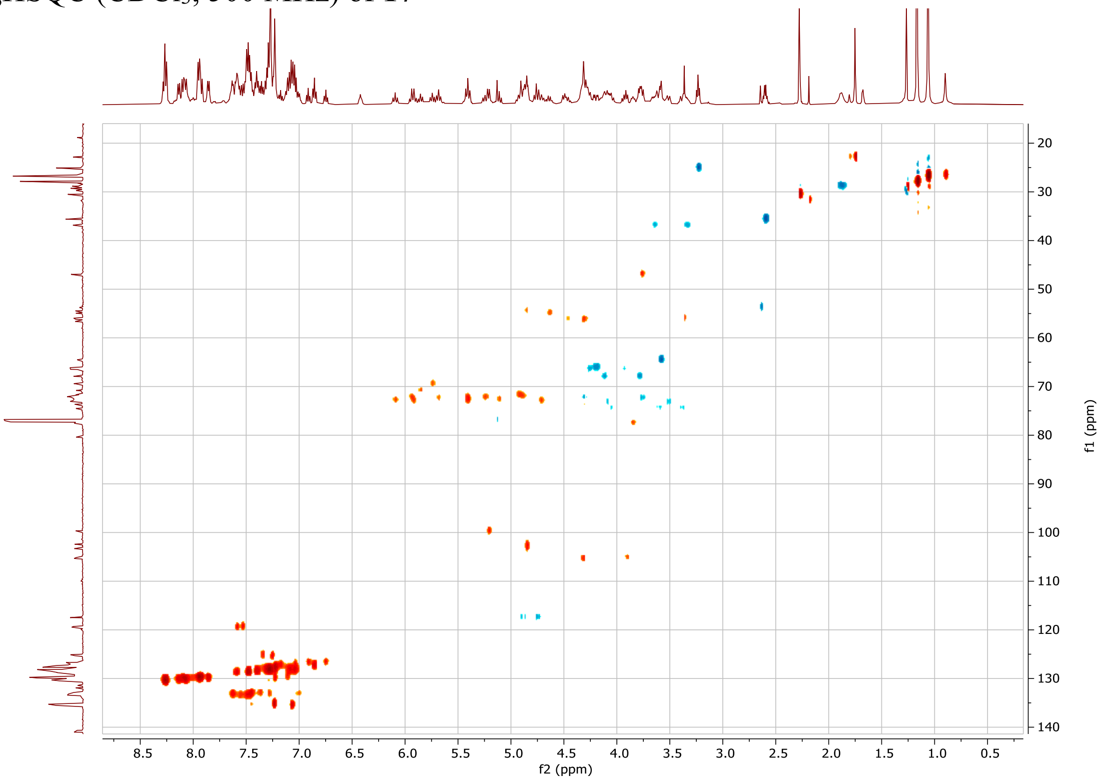

Supplementary Fig. 51. ^{1}H-NMR spectrum of compound **18**.

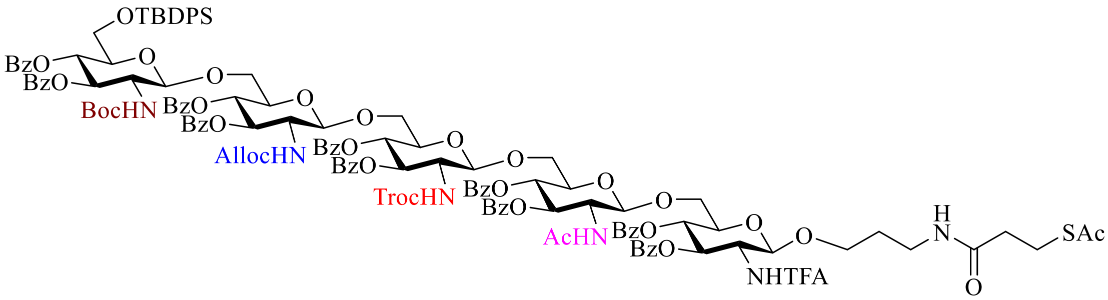

^{1}H-NMR ($CDCl_3$, 500 MHz) of **18**

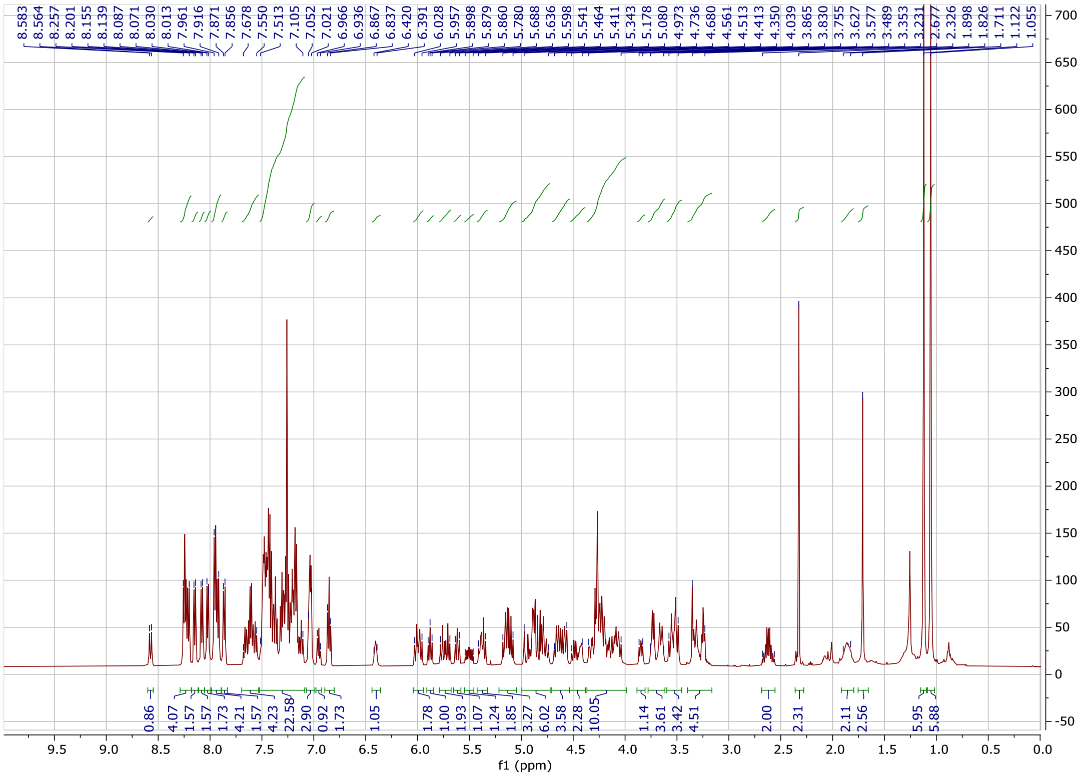

Supplementary Fig. 52. ^{13}C-NMR spectrum of compound **18**.

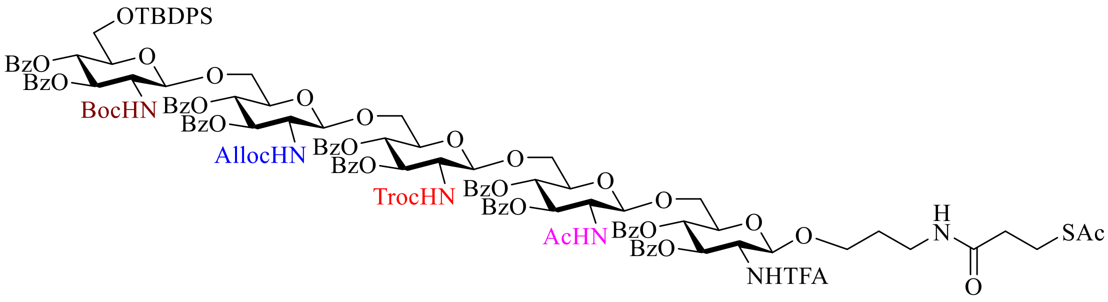

^{13}C-NMR ($CDCl_3$, 125 MHz) of **18**

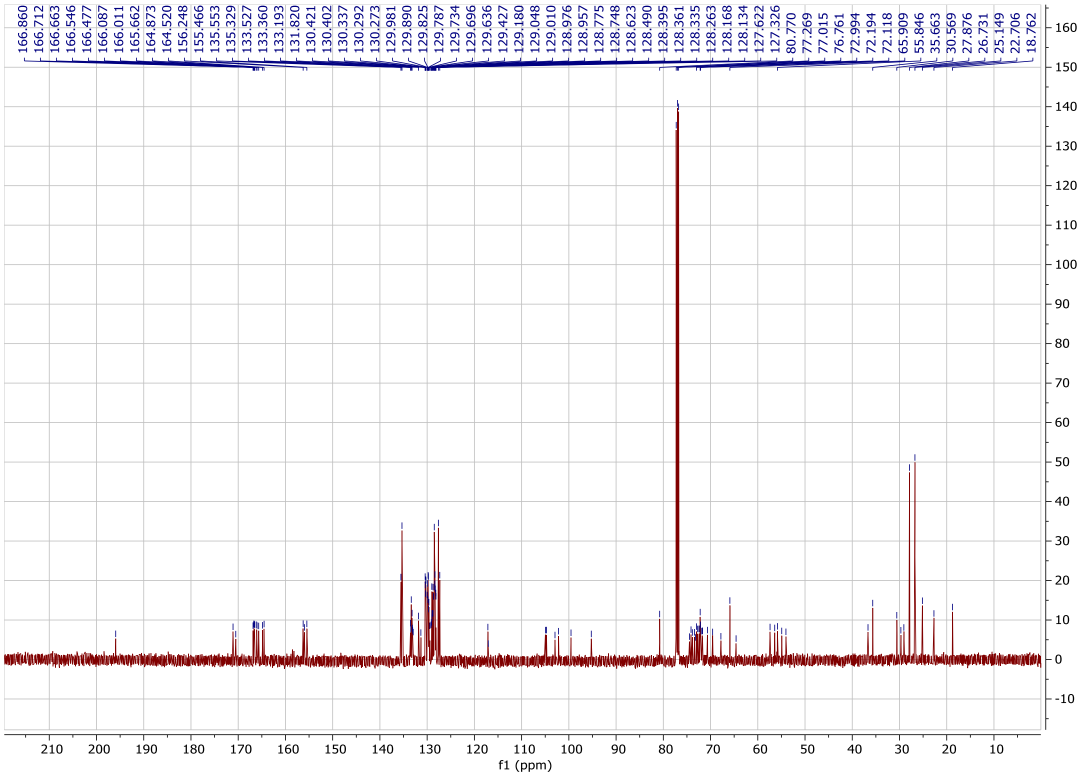

Supplementary Fig. 53. gHSQC spectrum of compound **18**.

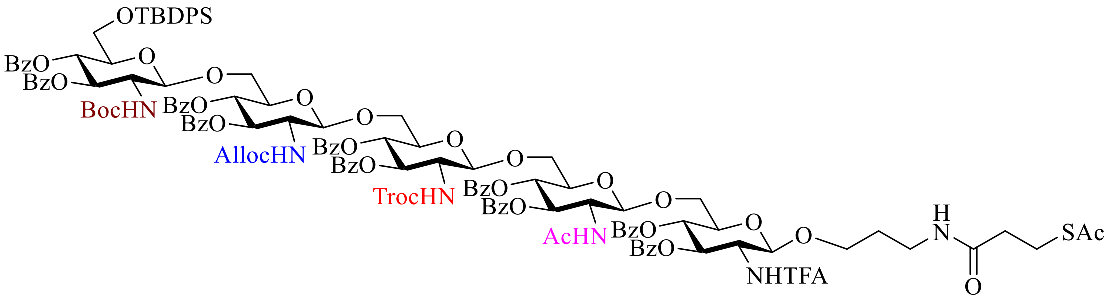

gHSQC ($CDCl_3$, 500 MHz) of **18**

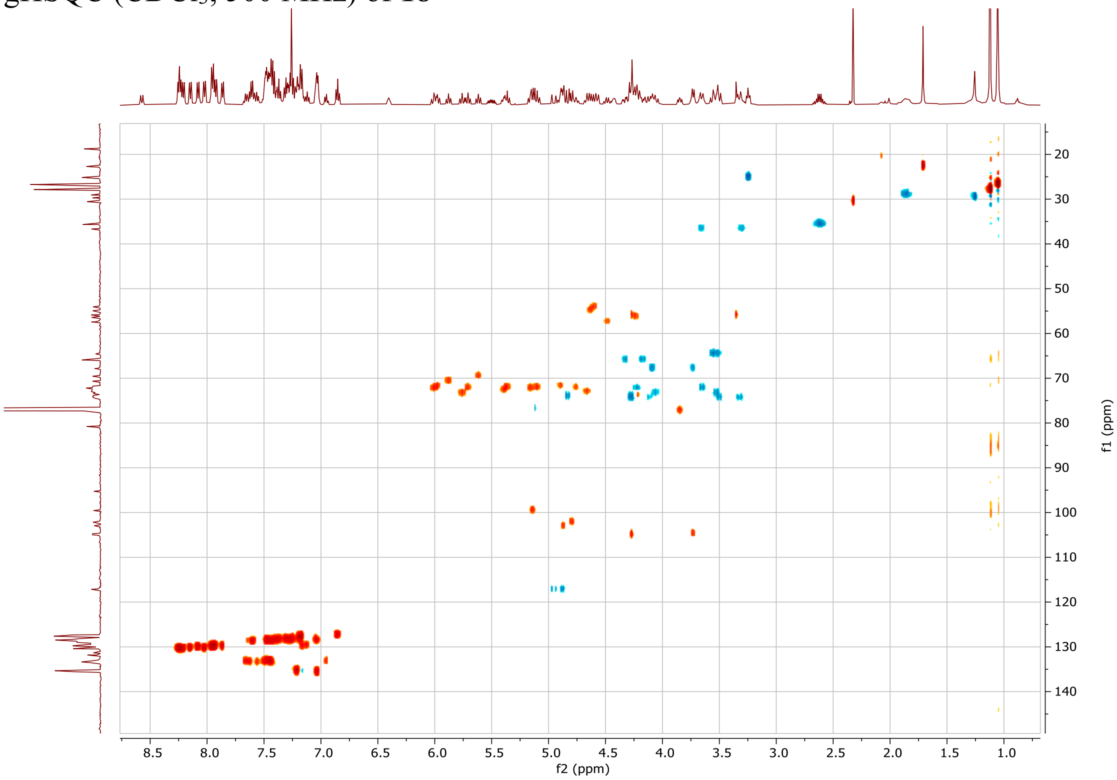

Supplementary Fig. 54. ^{1}H-NMR spectrum of compound **PNAG0**.

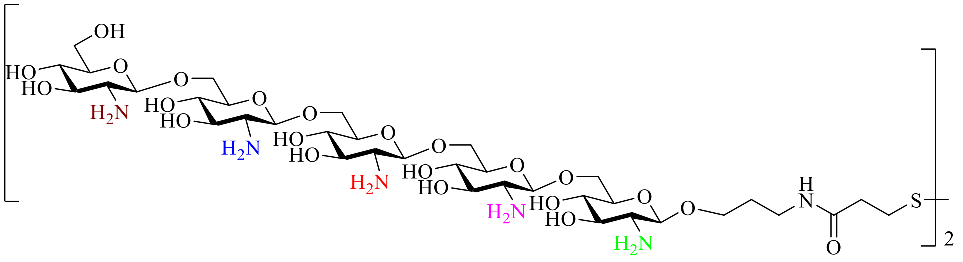

^{1}H-NMR (D_2O, 500 MHz) of **PNAG0**

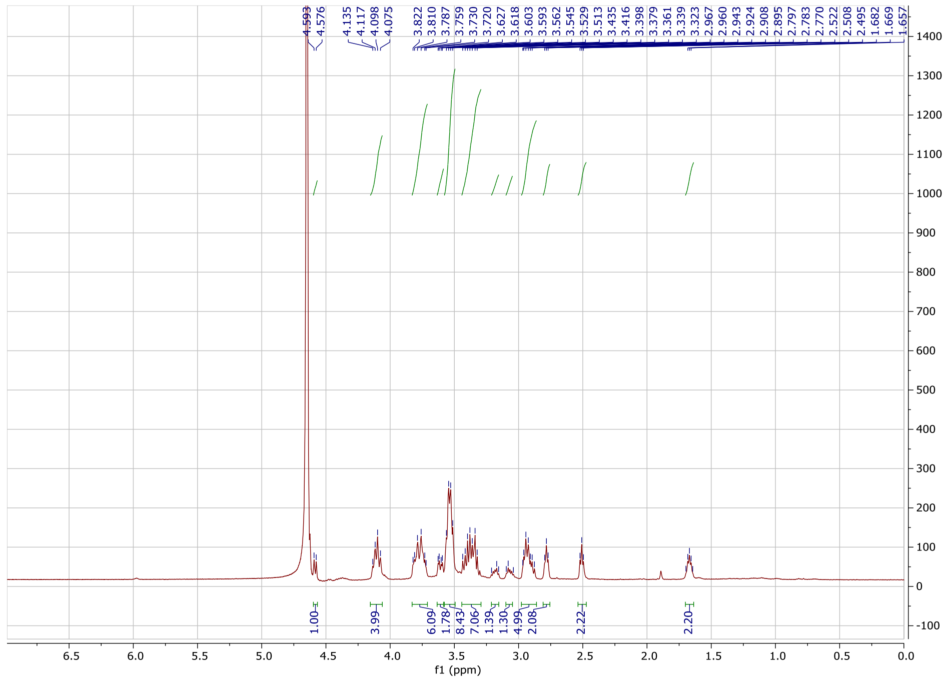

Supplementary Fig. 55. gHSQC spectrum of compound **PNAG0**.

gHSQC (D_2O, 500 MHz) of **PNAG0**

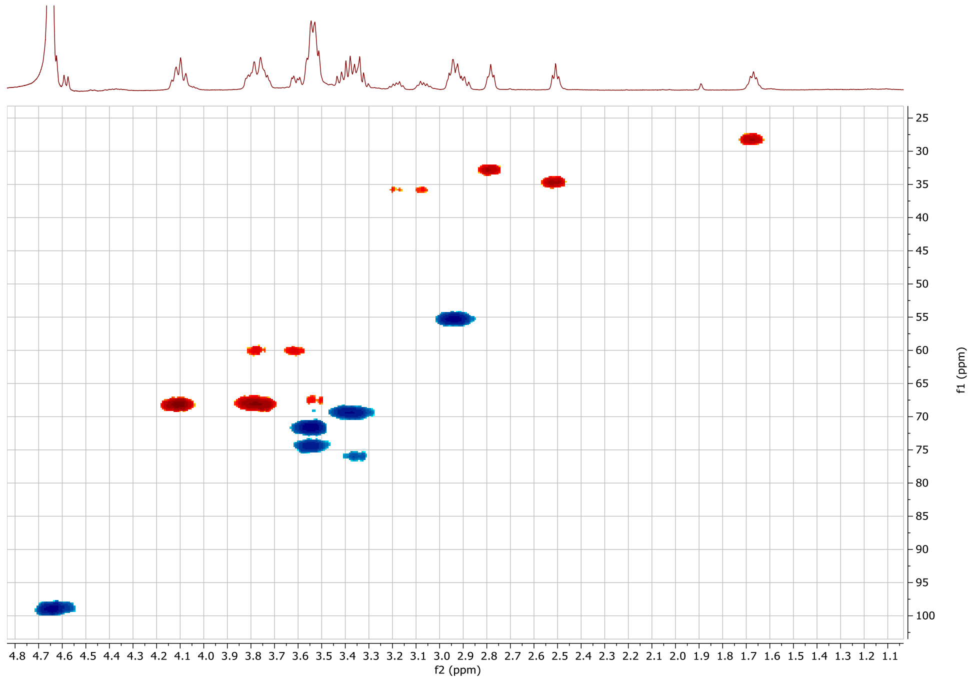

Supplementary Fig. 56. ^{1}H-NMR spectrum of compound **PNAG1**.

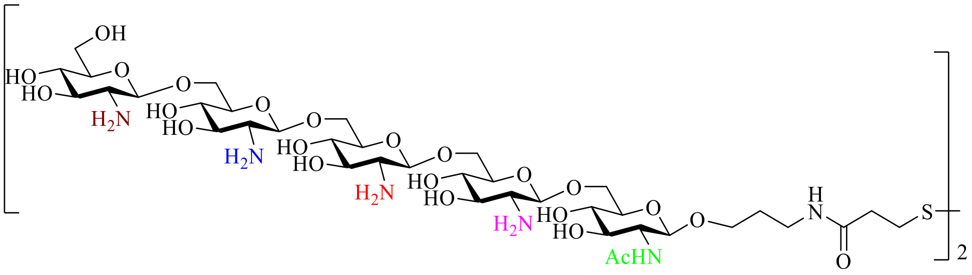

^{1}H-NMR (D_2O, 500 MHz) of **PNAG1**

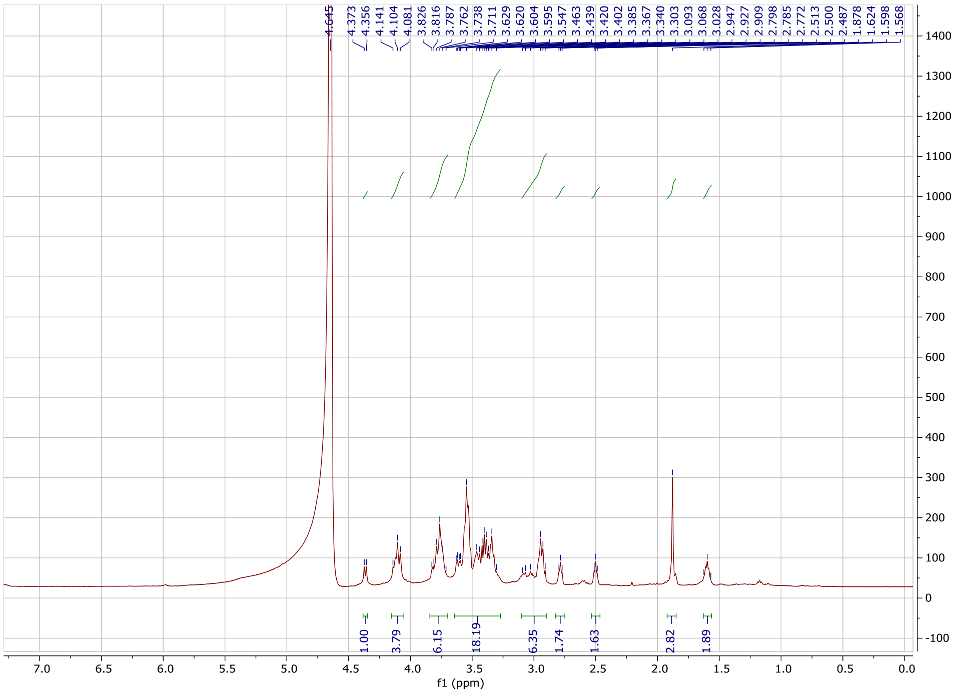

Supplementary Fig. 57. gHSQC spectrum of compound **PNAG1**.

gHSQC (D_2O, 500 MHz) of **PNAG1**

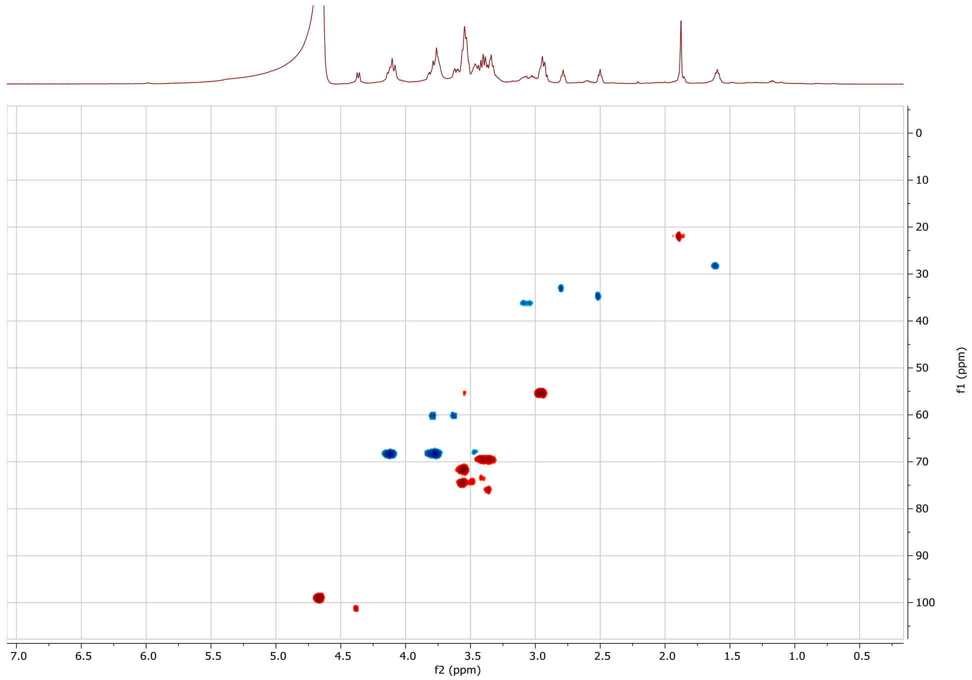

Supplementary Fig. 58. ^{1}H-NMR spectrum of compound **PNAG0**.

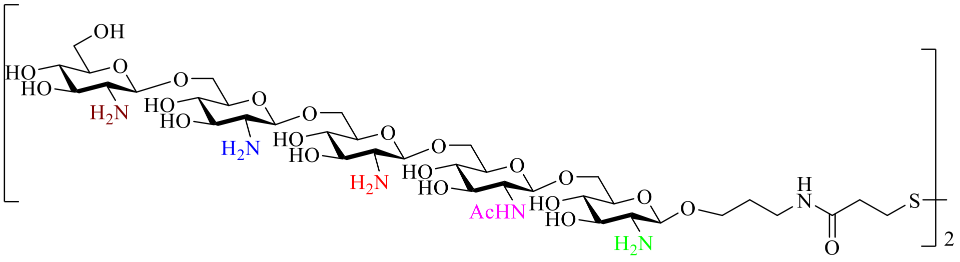

^{1}H-NMR (D_2O, 500 MHz) of **PNAG2**

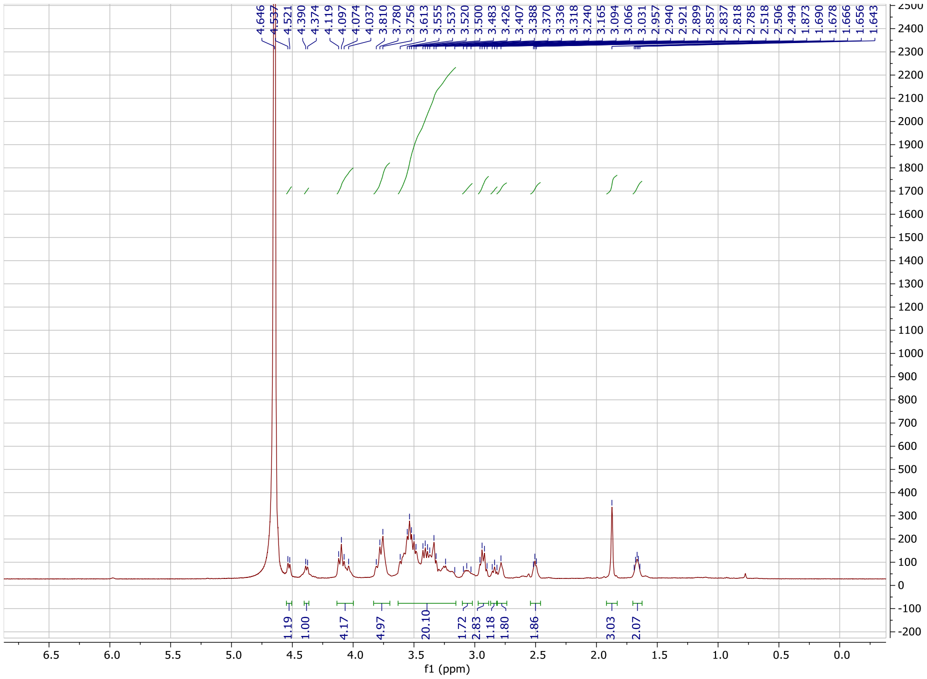

Supplementary Fig. 59. gHSQC spectrum of compound **PNAG2**.

gHSQC (D_2O, 500 MHz) of **PNAG2**

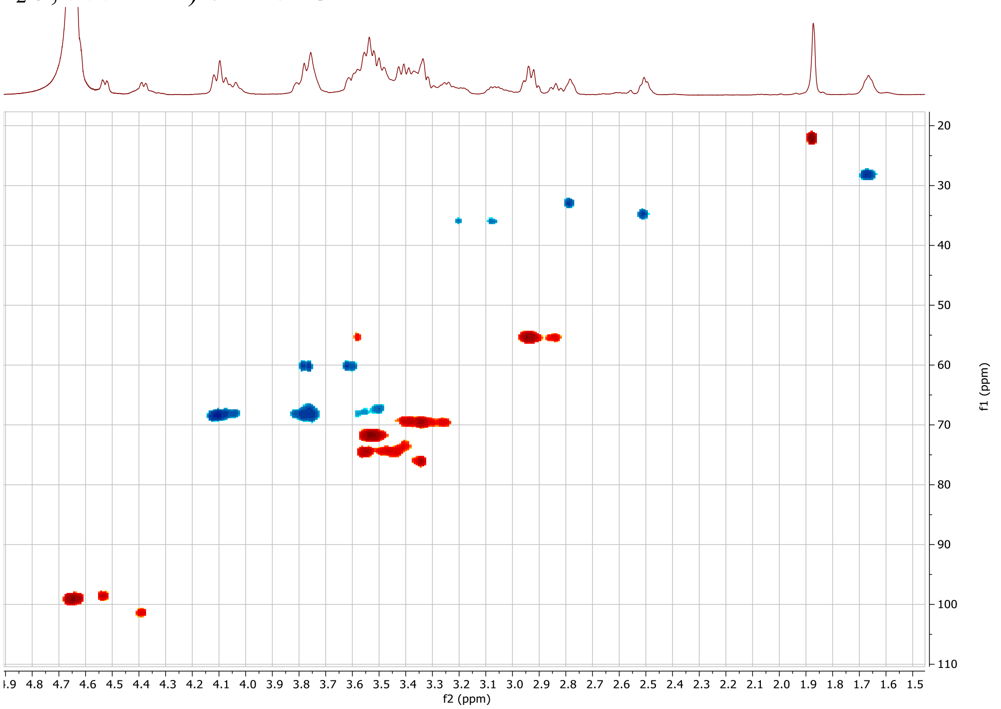

Supplementary Fig. 60. ^{1}H-NMR spectrum of compound **PNAG3**.

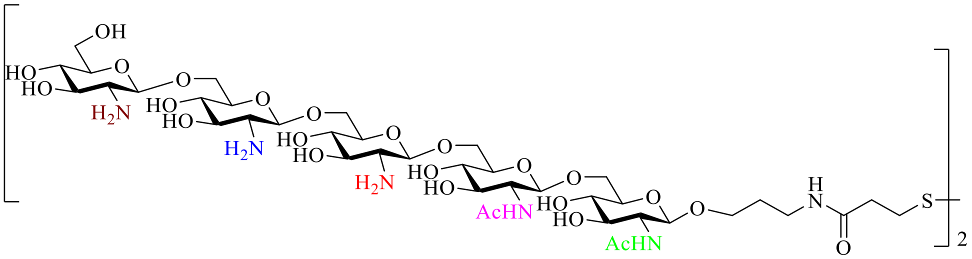

^{1}H-NMR (D_2O, 500 MHz) of **PNAG3**

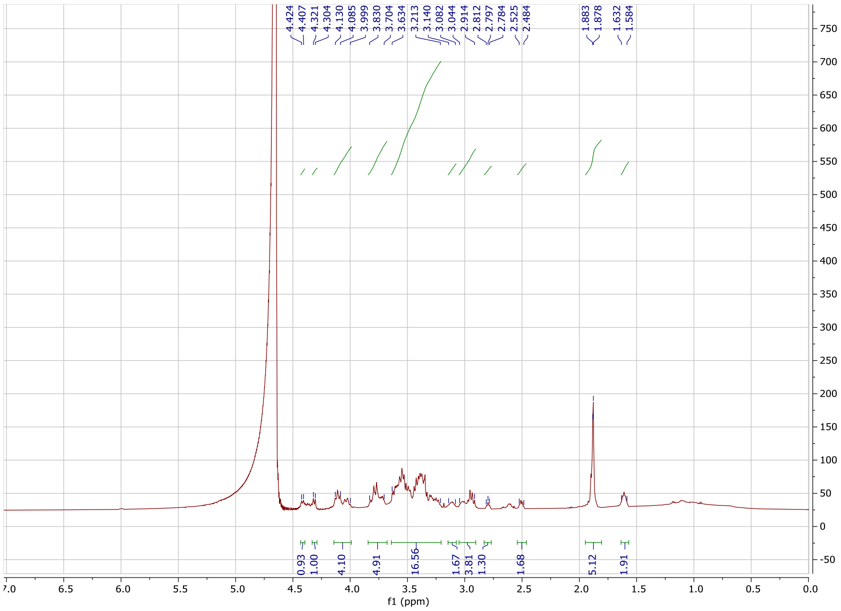

Supplementary Fig. 61. gHSQC spectrum of compound **PNAG3**.

gHSQC (D_2O, 500 MHz) of **PNAG3**

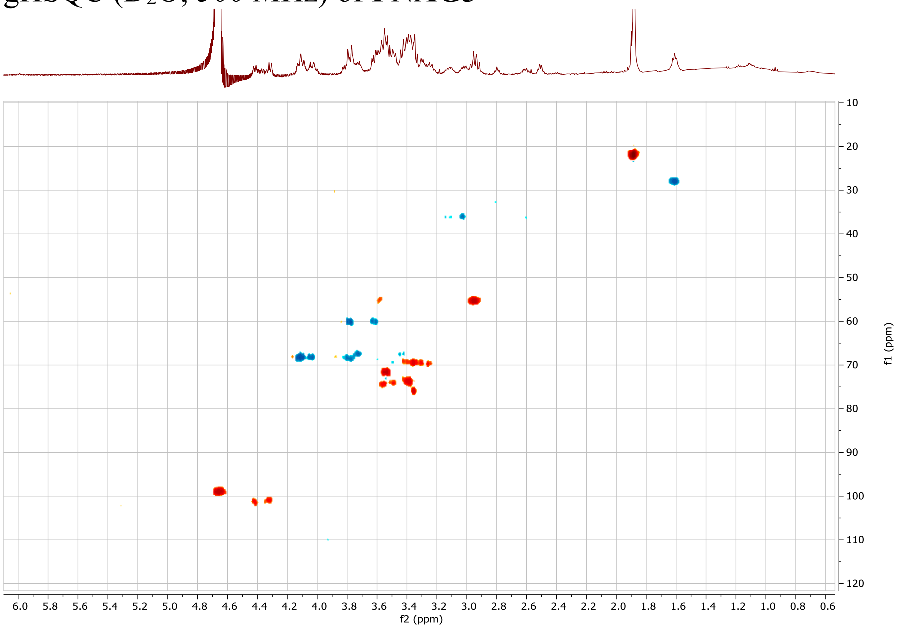

Supplementary Fig. 62. ^{1}H-NMR spectrum of compound **PNAG4**.

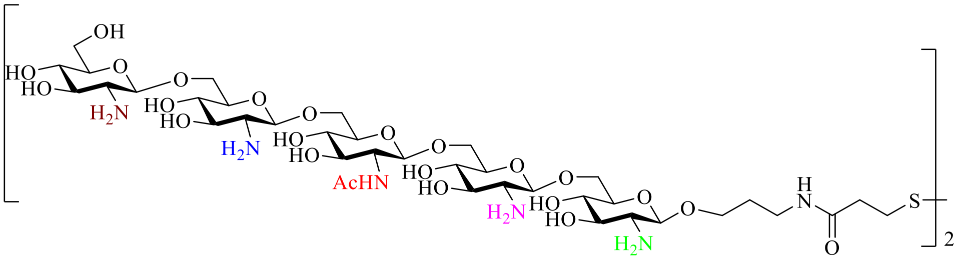

^{1}H-NMR (D_2O, 500 MHz) of **PNAG4**

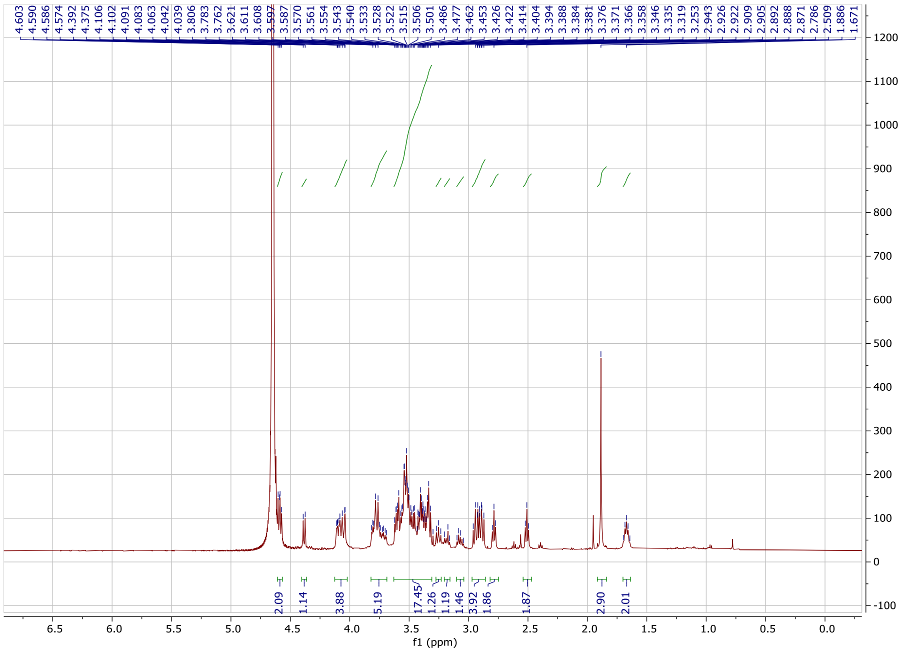

Supplementary Fig. 63. gHSQC spectrum of compound **PNAG4**.

gHSQC (D_2O, 500 MHz) of **PNAG4**

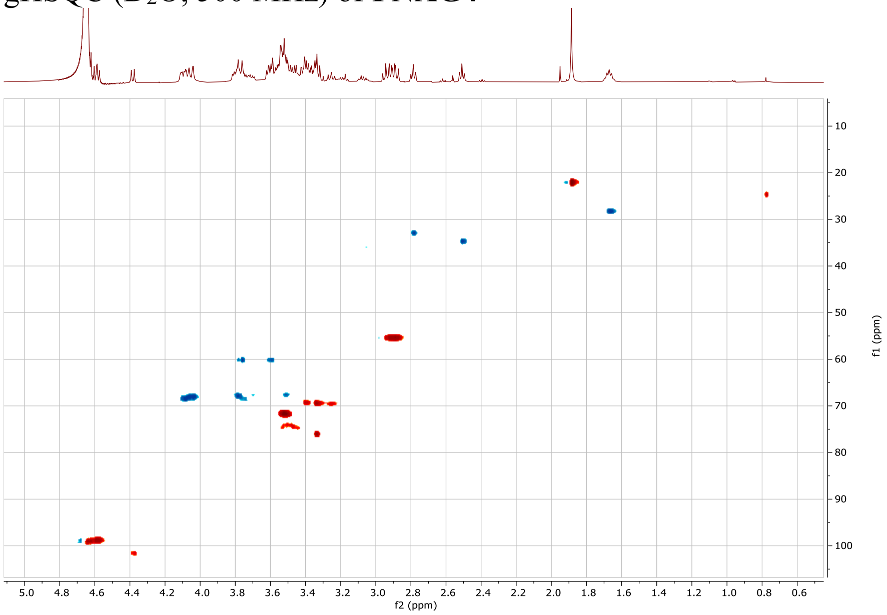

Supplementary Fig. 64. ^{1}H-NMR spectrum of compound **PNAG5**.

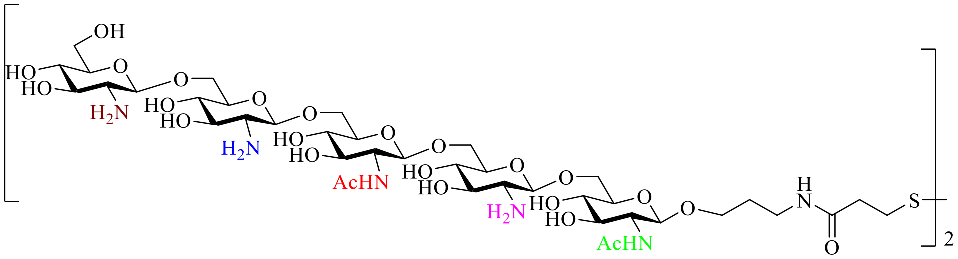

^{1}H-NMR (D_2O, 500 MHz) of **PNAG5**

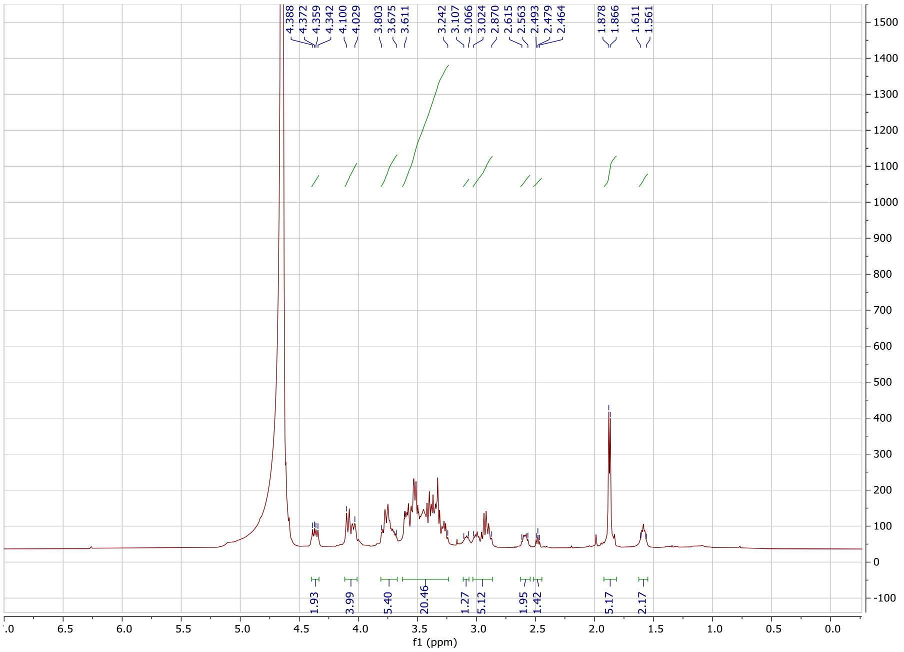

Supplementary Fig. 65. gHSQC spectrum of compound **PNAG5**.

gHSQC (D_2O, 500 MHz) of **PNAG5**

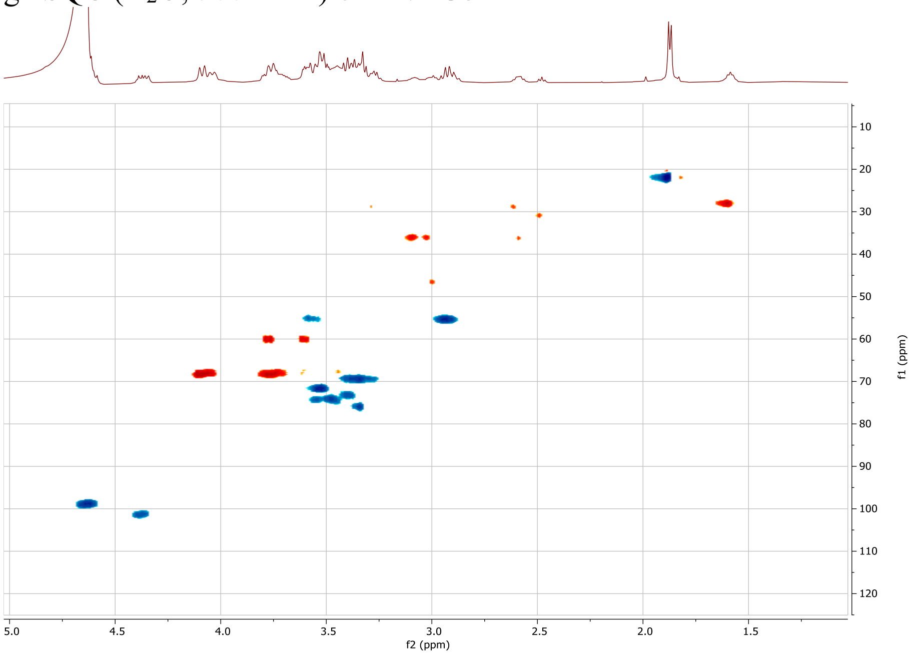

Supplementary Fig. 66. ^{1}H-NMR spectrum of compound **PNAG6**.

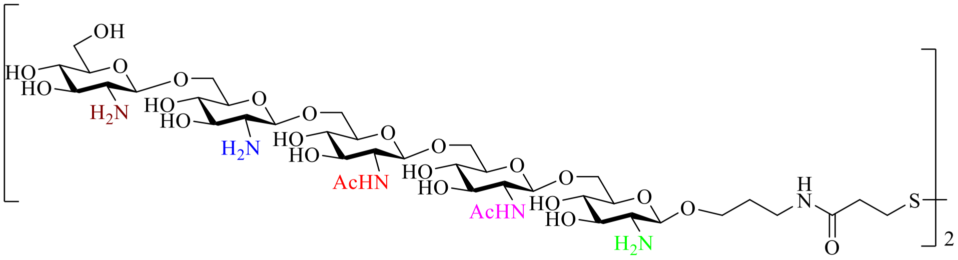

^{1}H-NMR (D_2O, 500 MHz) of **PNAG6**

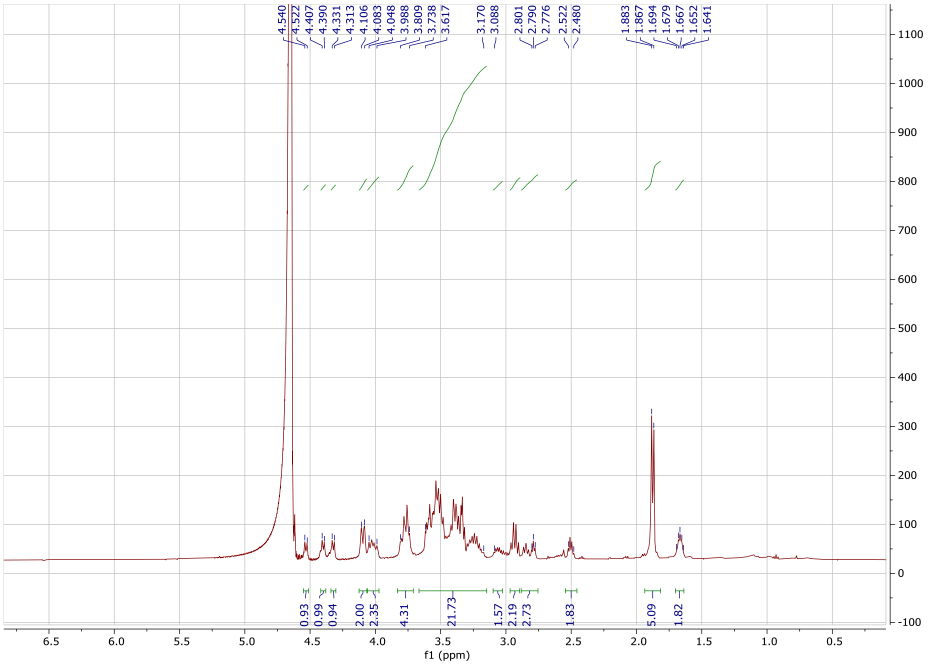

Supplementary Fig. 67. gHSQC spectrum of compound **PNAG6**.

gHSQC (D_2O, 500 MHz) of **PNAG6**

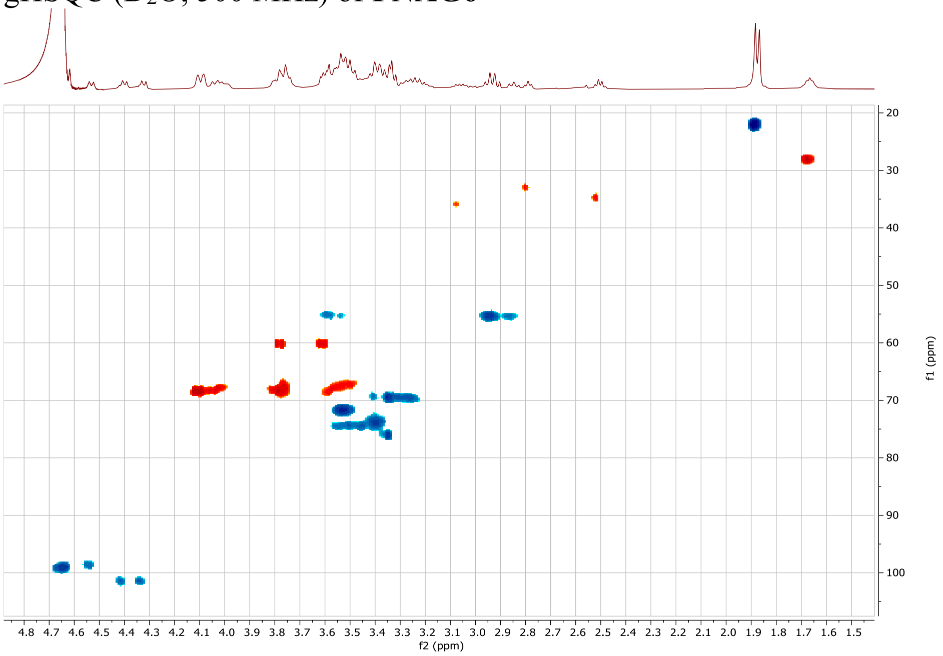

Supplementary Fig. 68. ^{1}H-NMR spectrum of compound **PNAG7**.

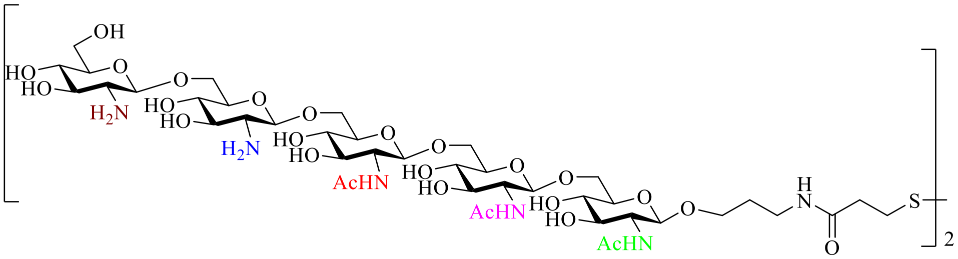

^{1}H-NMR (D_2O, 500 MHz) of **PNAG7**

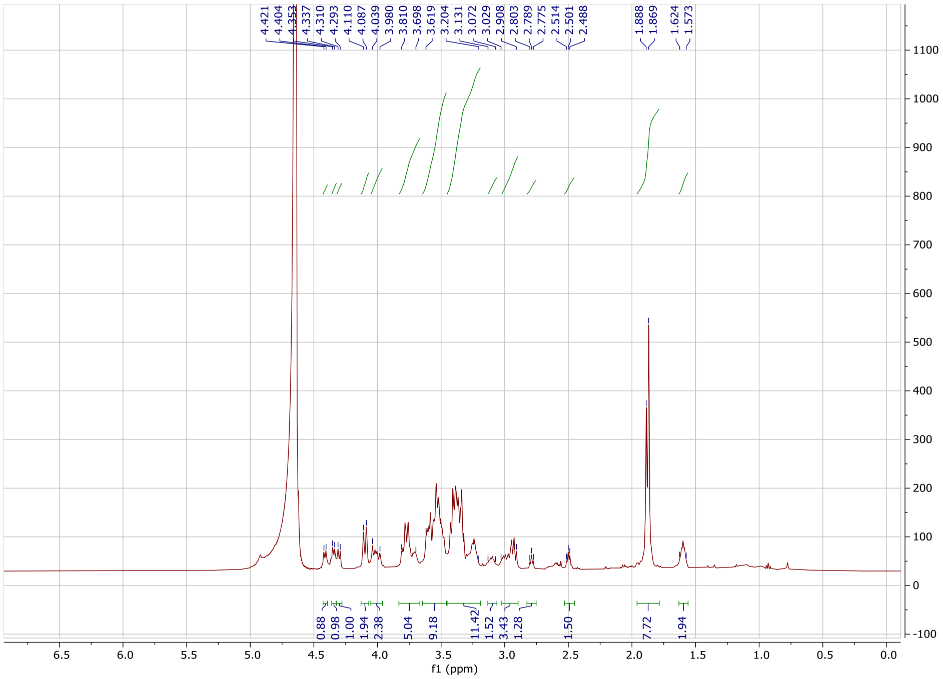

Supplementary Fig. 69. gHSQC spectrum of compound **PNAG7**.

gHSQC (D_2O, 500 MHz) of **PNAG7**

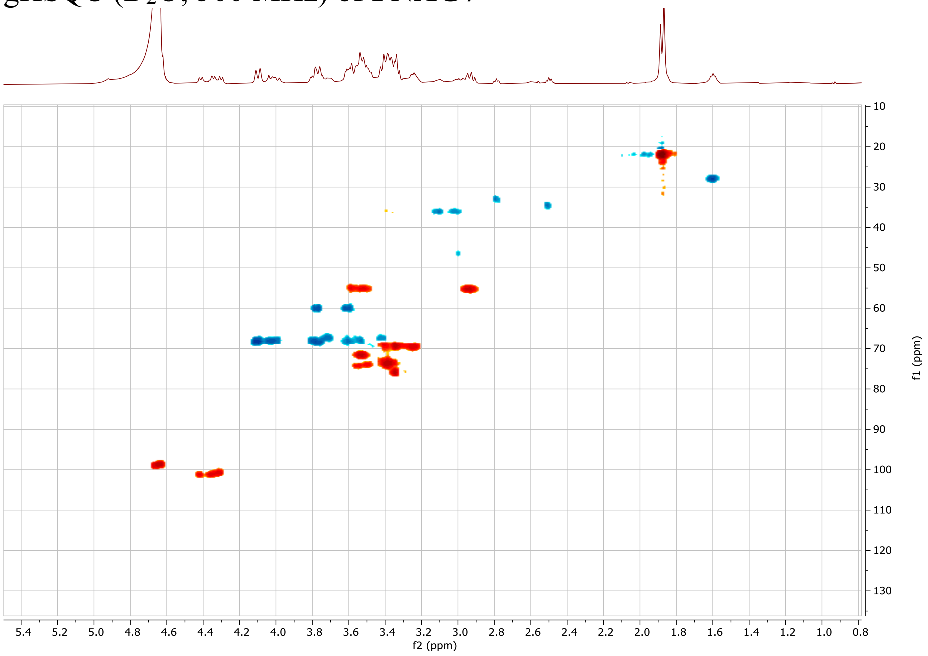

Supplementary Fig. 70. ^{1}H-NMR spectrum of compound **PNAG8**.

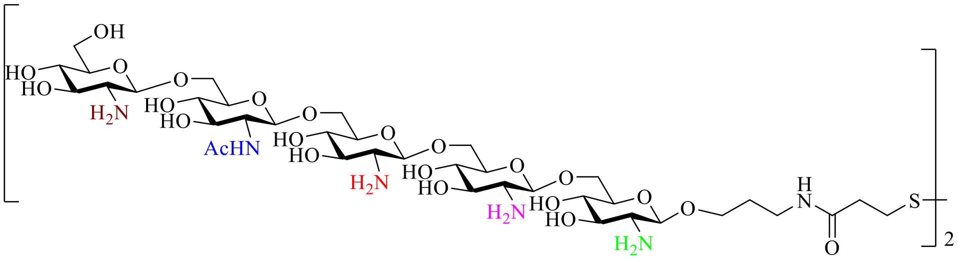

^{1}H-NMR (D_2O, 500 MHz) of **PNAG8**

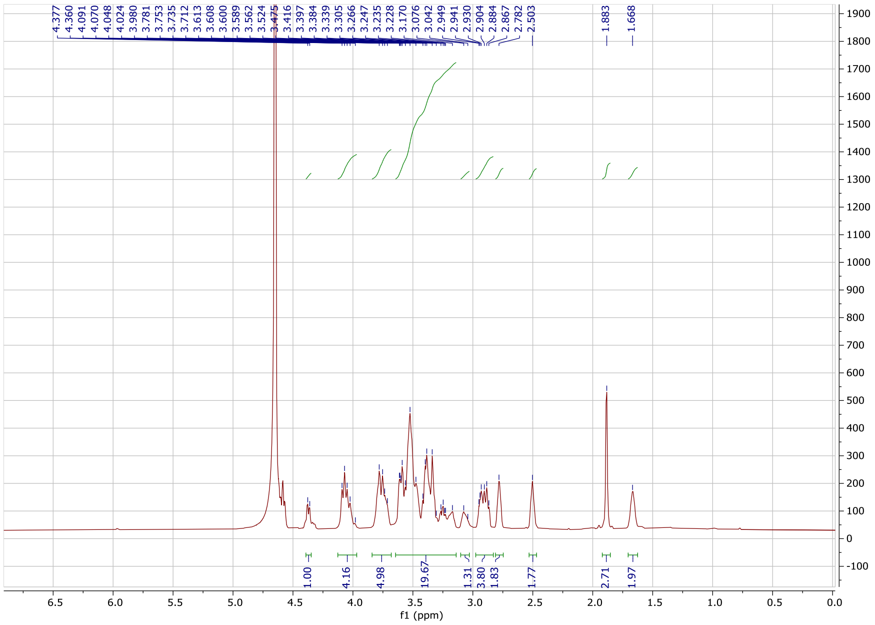

Supplementary Fig. 71. gHSQC spectrum of compound **PNAG8**.

gHSQC (D_2O, 500 MHz) of **PNAG8**

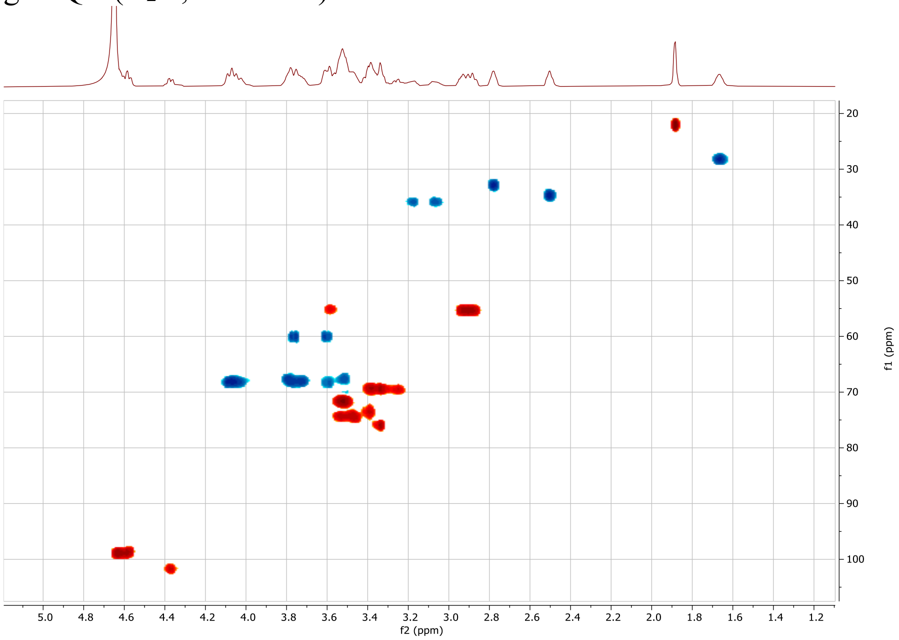

Supplementary Fig. 72. ^{1}H-NMR spectrum of compound **PNAG9**.

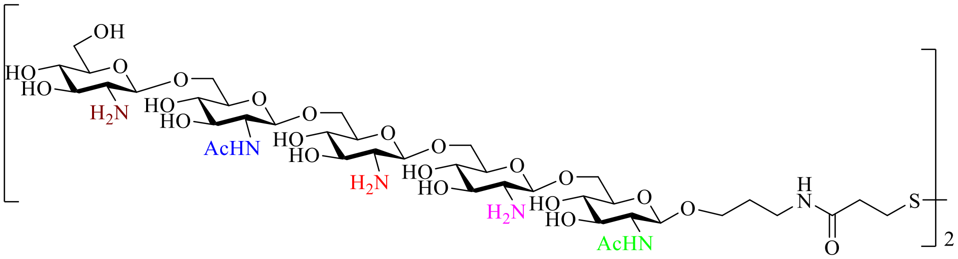

^{1}H-NMR (D_2O, 500 MHz) of **PNAG9**

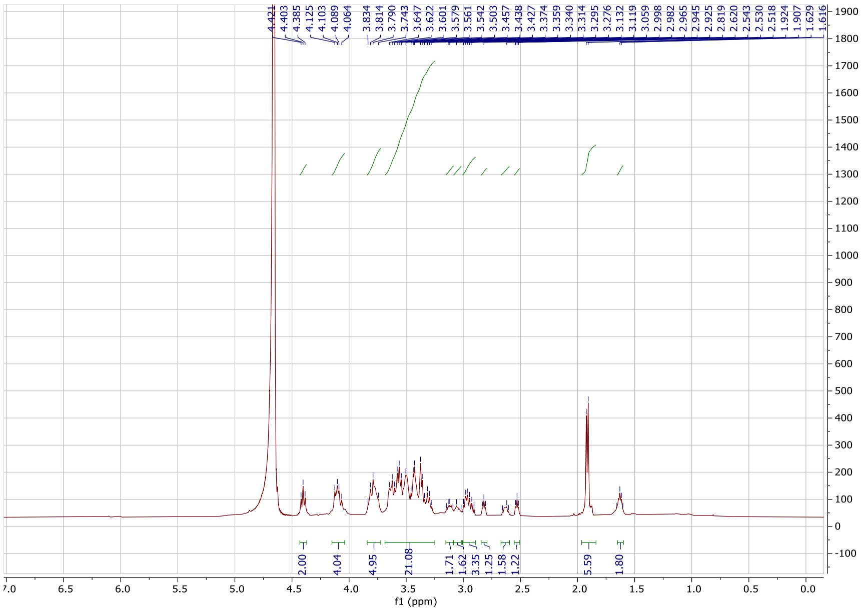

Supplementary Fig. 73. gHSQC spectrum of compound **PNAG9**.

gHSQC (D_2O, 500 MHz) of **PNAG9**

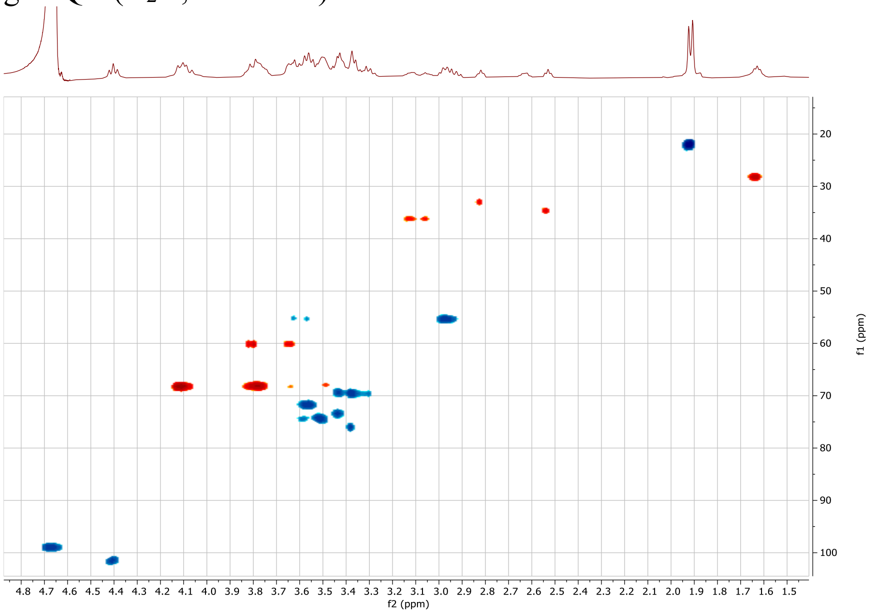

Supplementary Fig. 74. ^{1}H-NMR spectrum of compound **PNAG10**.

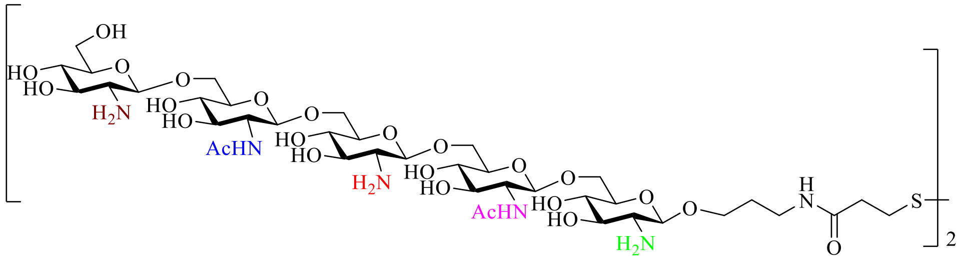

^{1}H-NMR (D_2O, 500 MHz) of **PNAG10**

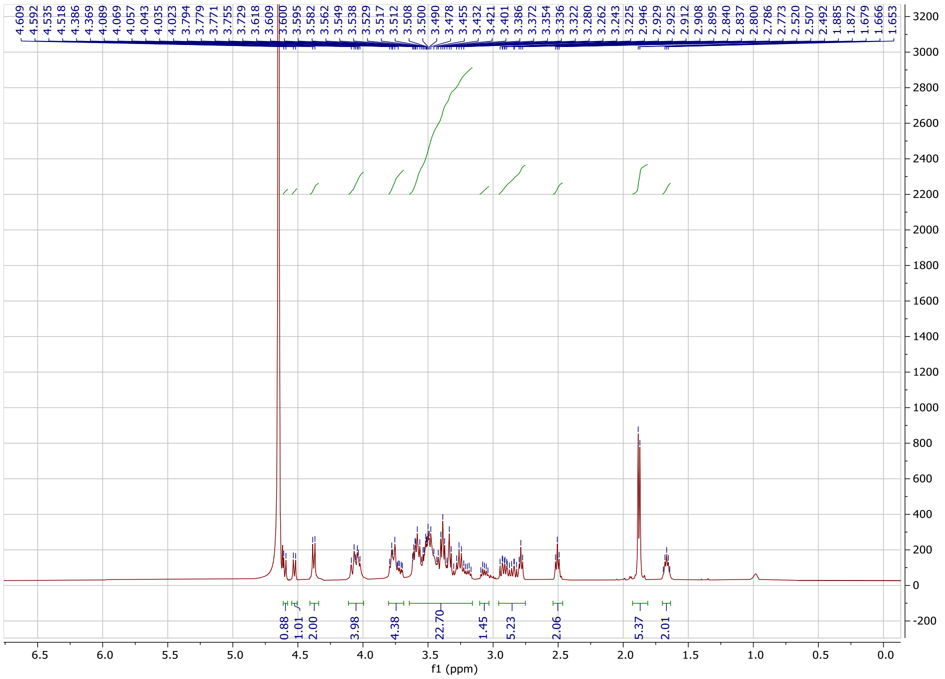

Supplementary Fig. 75. gHSQC spectrum of compound **PNAG10**.

gHSQC (D_2O, 500 MHz) of **PNAG10**

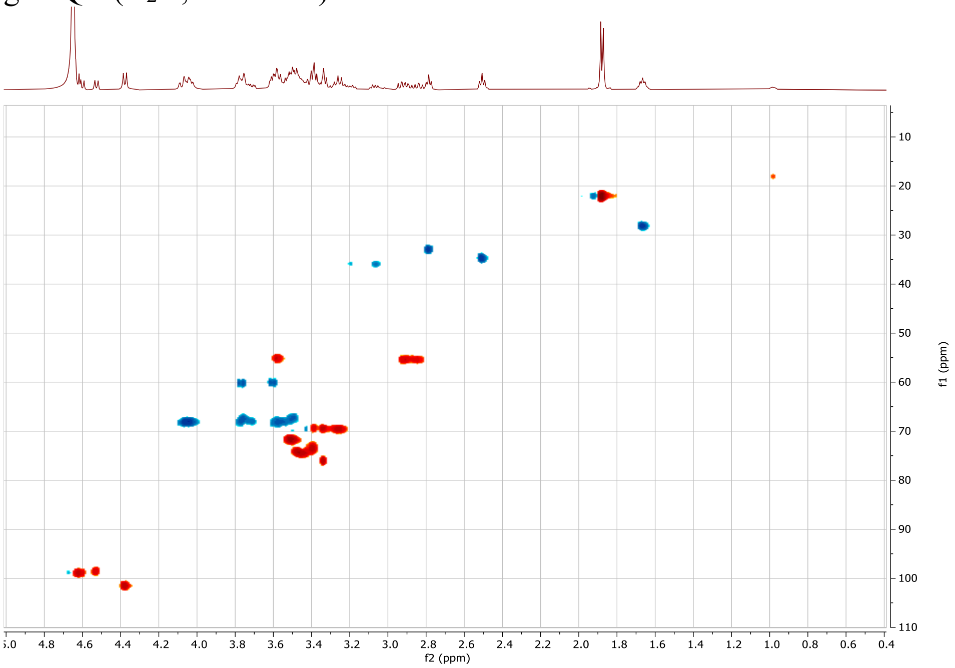

Supplementary Fig. 76. ^{1}H-NMR spectrum of compound **PNAG11**.

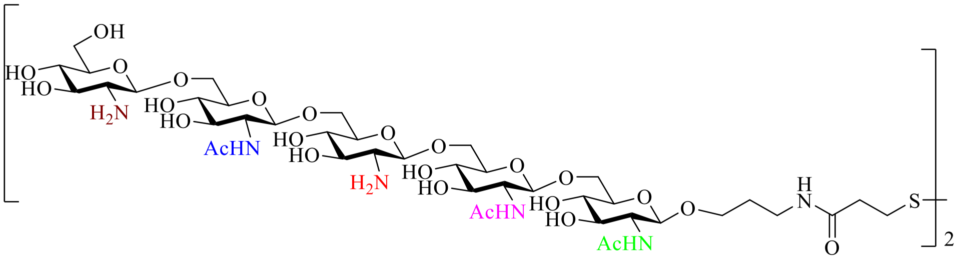

^{1}H-NMR (D_2O, 500 MHz) of **PNAG11**

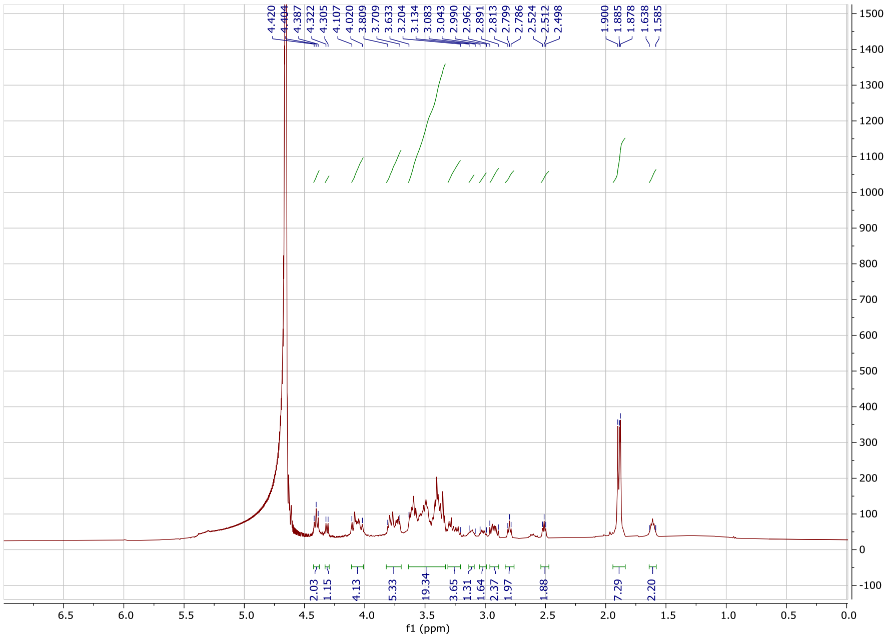

Supplementary Fig. 77. gHSQC spectrum of compound **PNAG11**.

gHSQC (D_2O, 500 MHz) of **PNAG11**

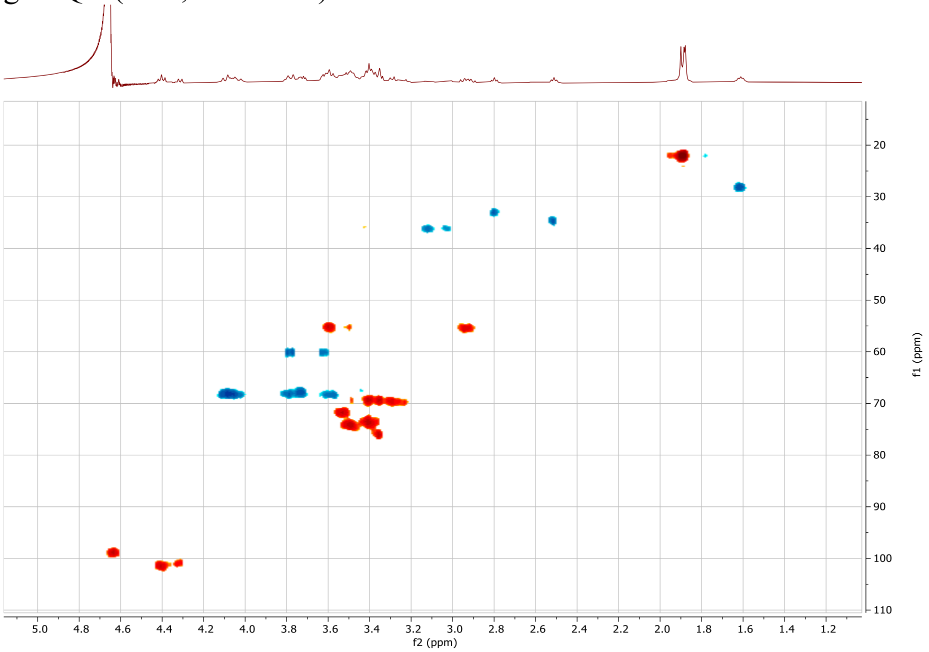

Supplementary Fig. 78. ^{1}H-NMR spectrum of compound **PNAG12**.

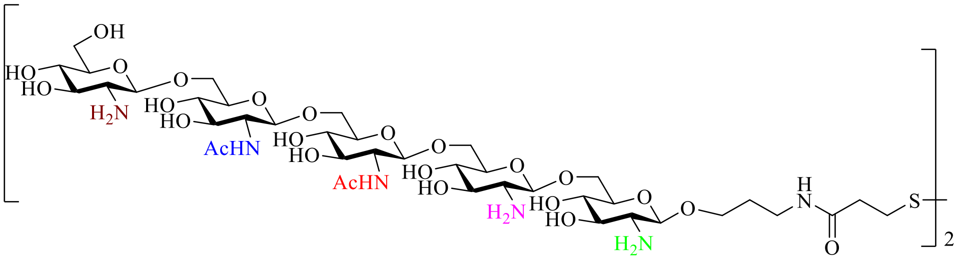

^{1}H-NMR (D_2O, 500 MHz) of **PNAG12**

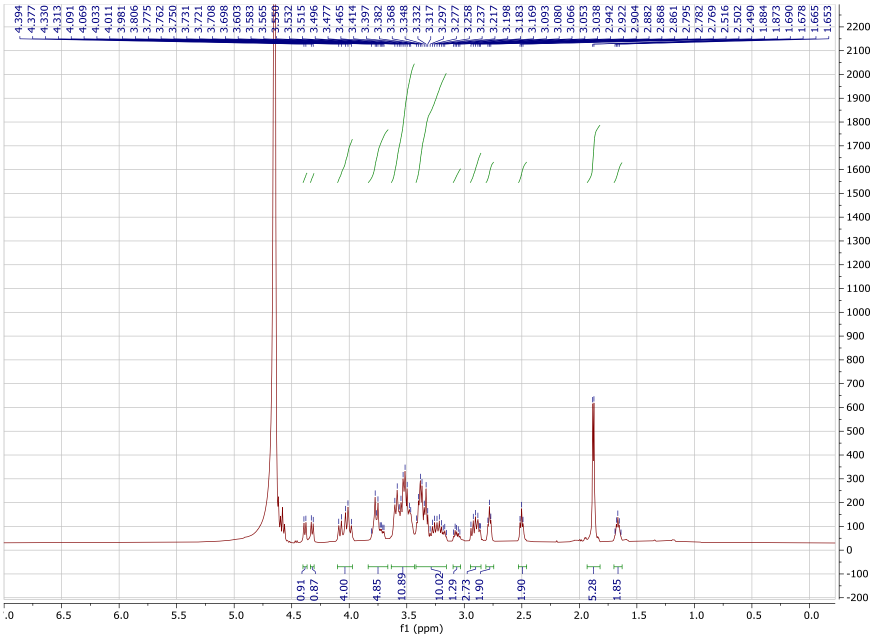

Supplementary Fig. 79. gHSQC spectrum of compound **PNAG12**.

gHSQC (D_2O, 500 MHz) of **PNAG12**

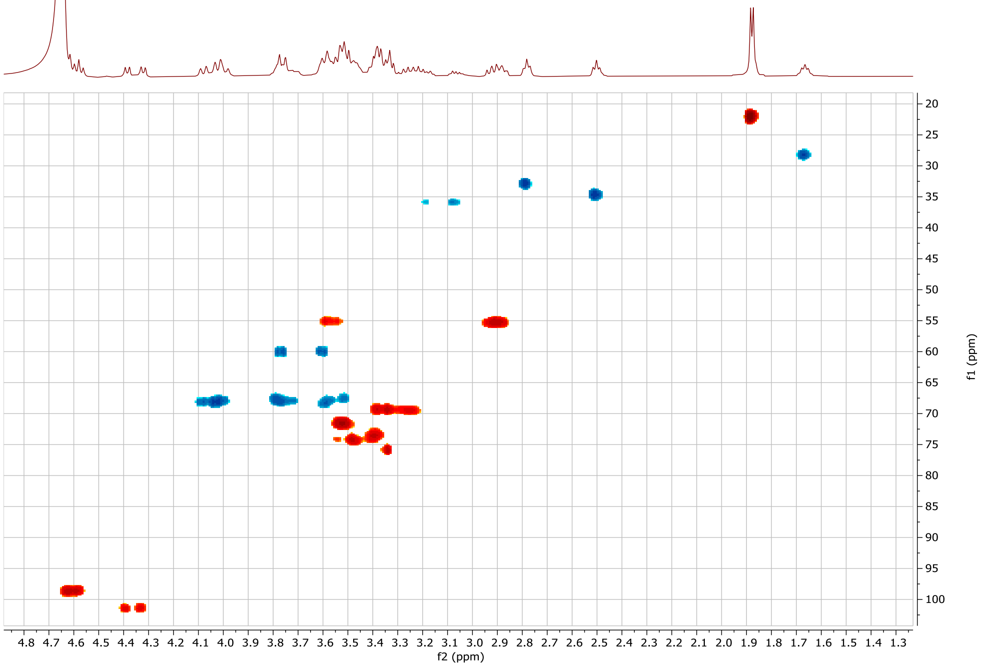

Supplementary Fig. 80. ^{1}H-NMR spectrum of compound **PNAG13**.

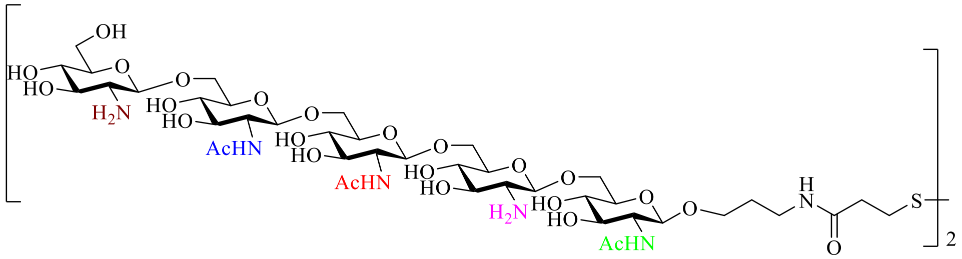

^{1}H-NMR (D_2O, 500 MHz) of **PNAG13**

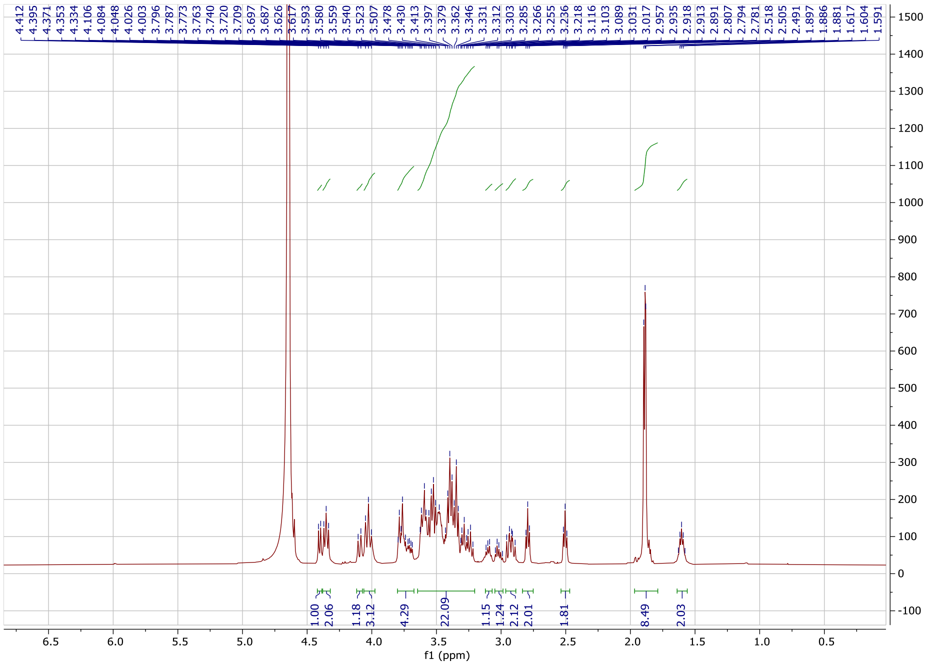

Supplementary Fig. 81. gHSQC spectrum of compound **PNAG13**.

gHSQC (D_2O, 500 MHz) of **PNAG13**

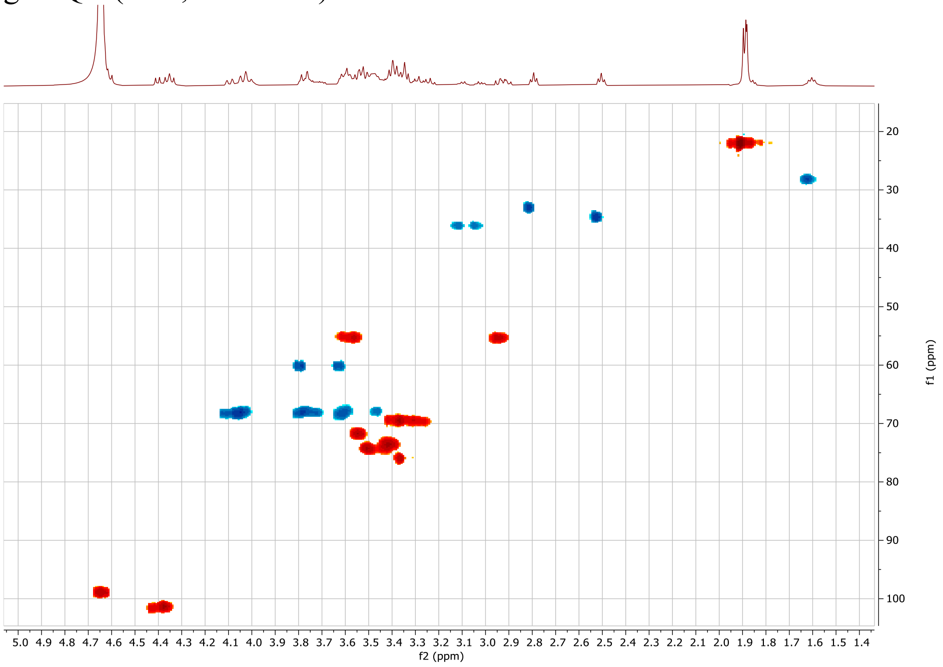

Supplementary Fig. 82. ^{1}H-NMR spectrum of compound **PNAG14**.

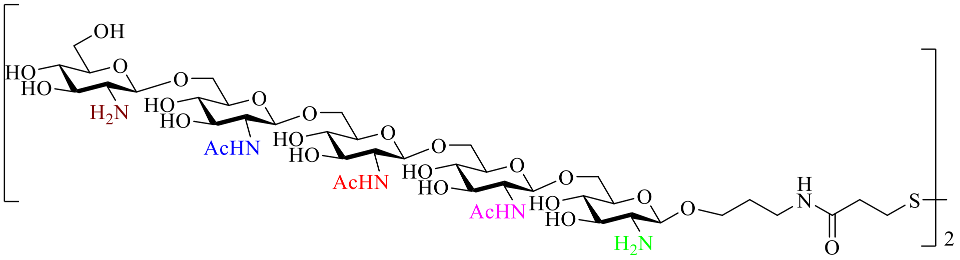

^{1}H-NMR (D_2O, 500 MHz) of **PNAG14**

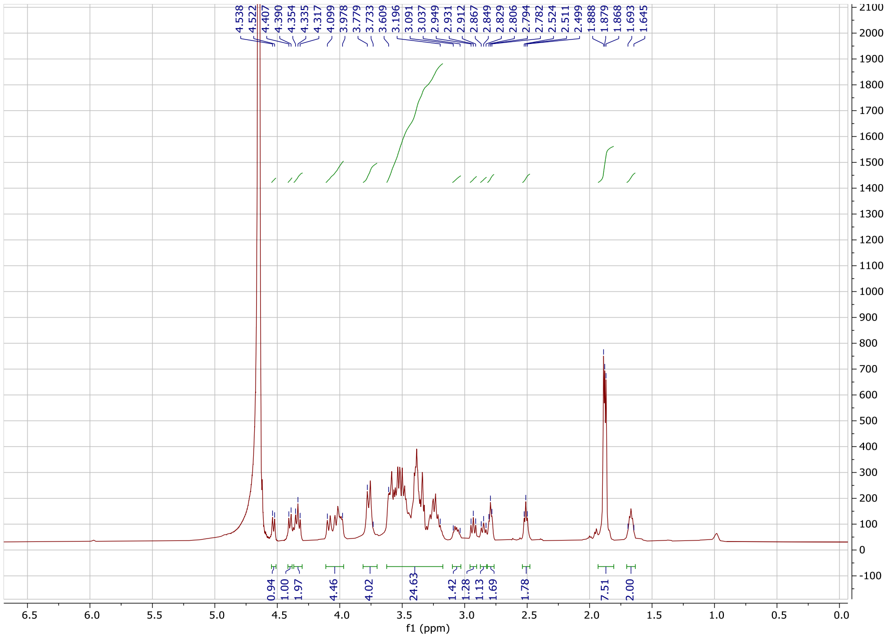

Supplementary Fig. 83. gHSQC spectrum of compound **PNAG14**.

gHSQC (D_2O, 500 MHz) of **PNAG14**

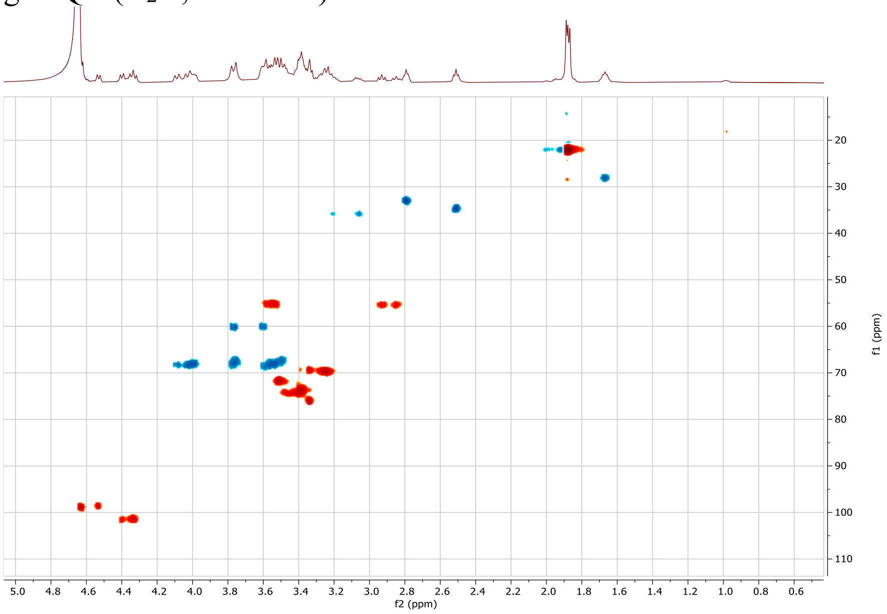

Supplementary Fig. 84. ^{1}H-NMR spectrum of compound **PNAG15**.

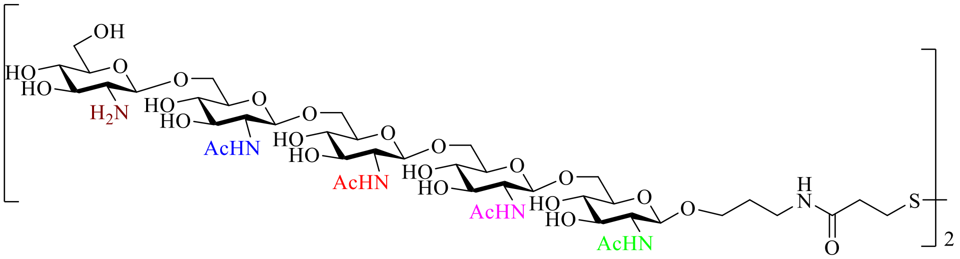

^{1}H-NMR (D_2O, 500 MHz) of **PNAG15**

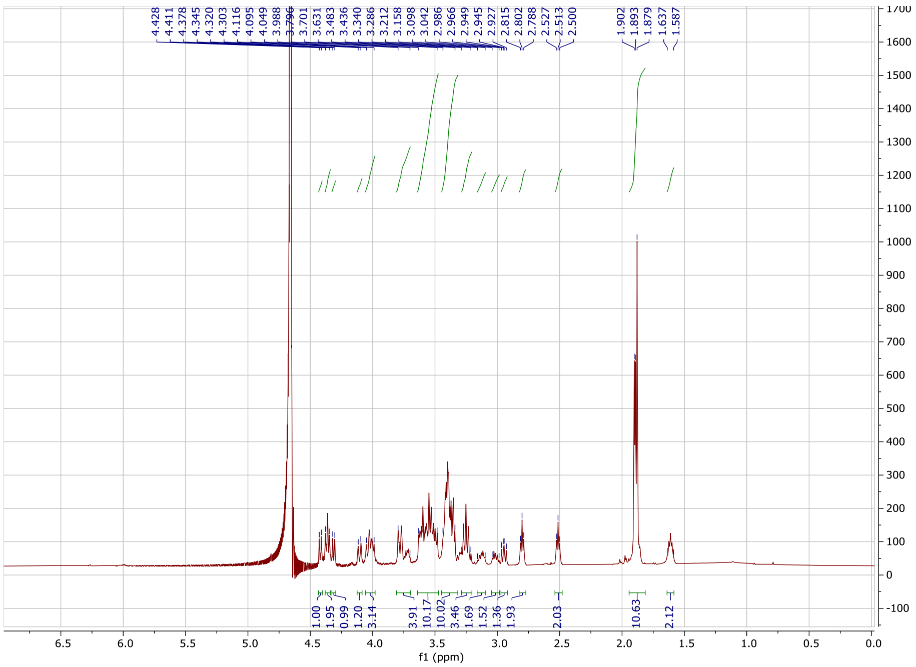

Supplementary Fig. 85. gHSQC spectrum of compound **PNAG15**.

gHSQC (D_2O, 500 MHz) of **PNAG15**

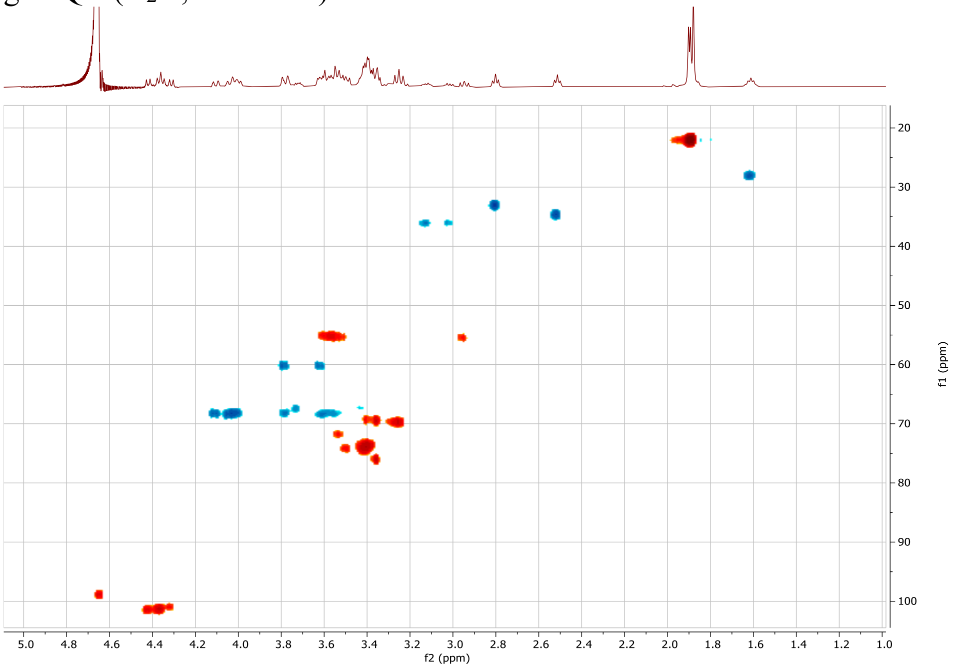

Supplementary Fig. 86. ^{1}H-NMR spectrum of compound **PNAG16**.

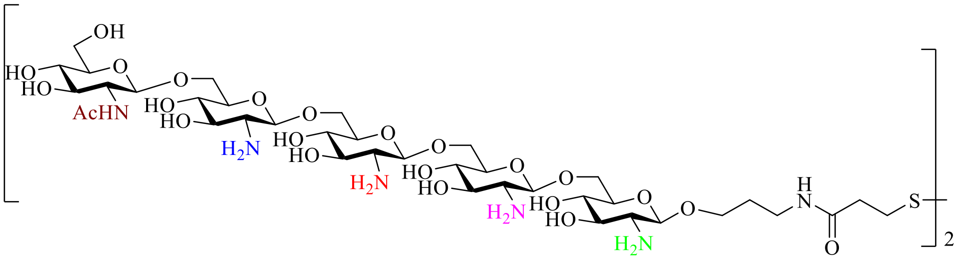

^{1}H-NMR (D_2O, 500 MHz) of **PNAG16**

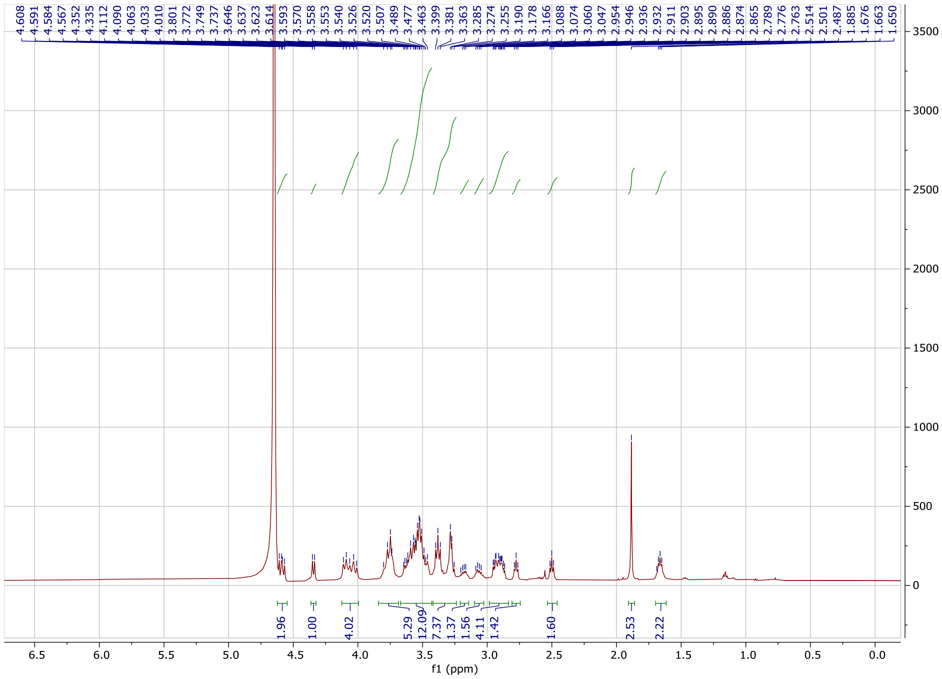

Supplementary Fig. 87. gHSQC spectrum of compound **PNAG16**.

gHSQC (D_2O, 500 MHz) of **PNAG16**

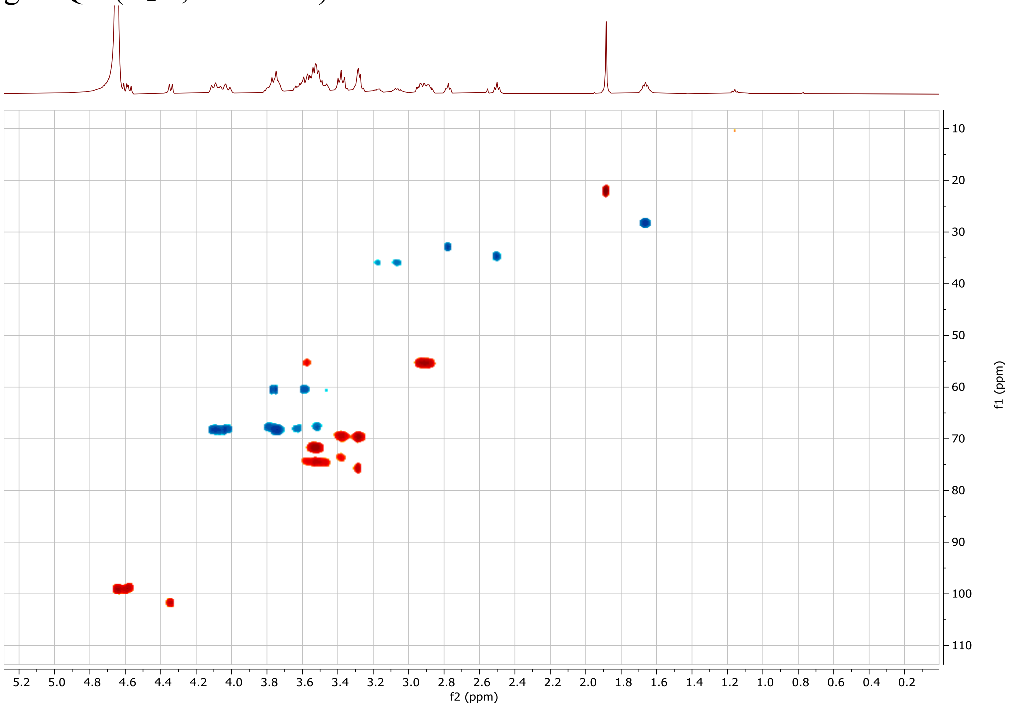

Supplementary Fig. 88. ^{1}H-NMR spectrum of compound **PNAG17**.

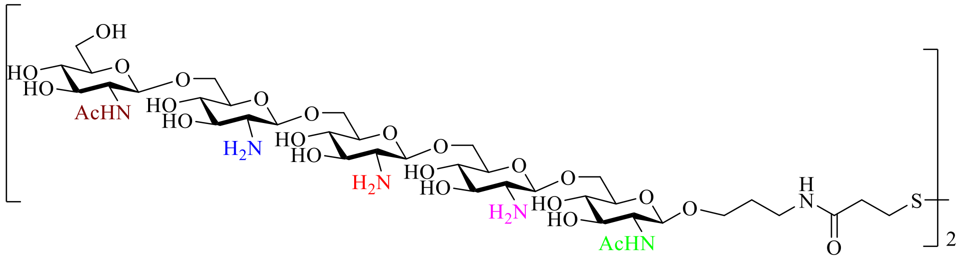

^{1}H-NMR (D_2O, 500 MHz) of **PNAG17**

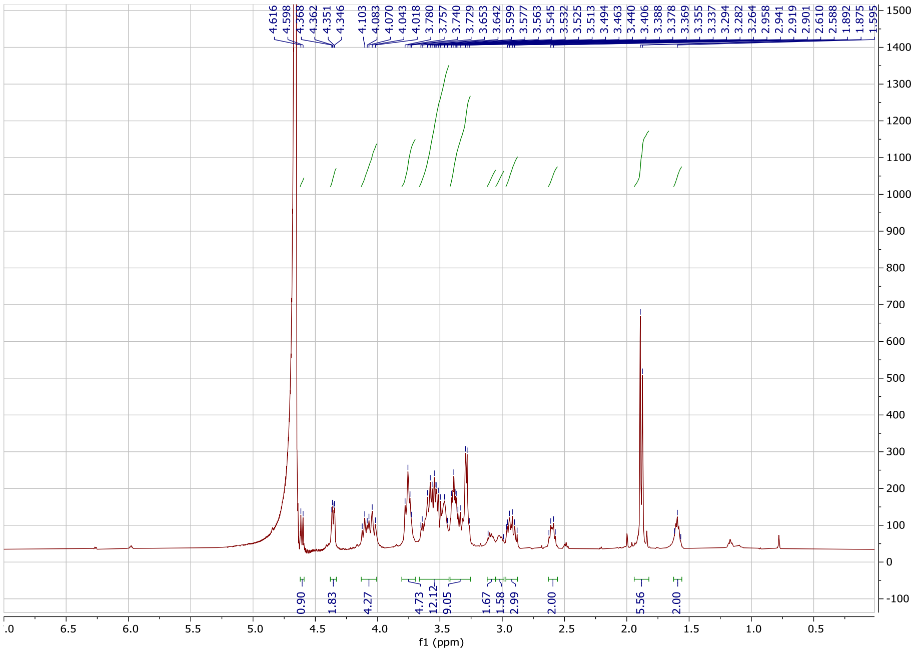

Supplementary Fig. 89. gHSQC spectrum of compound **PNAG17**.

gHSQC (D_2O, 500 MHz) of **PNAG17**

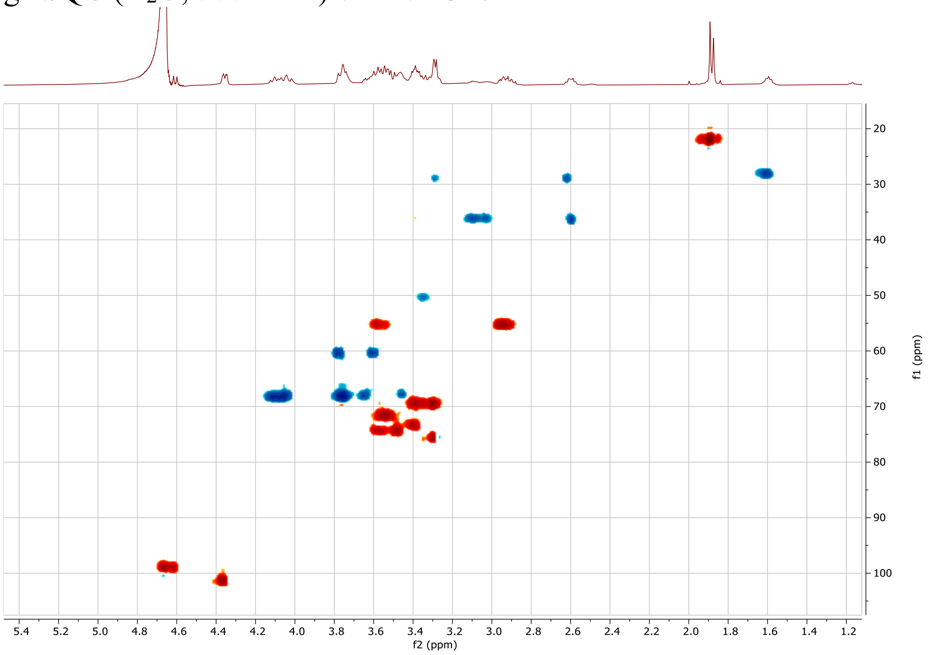

Supplementary Fig. 90. ^{1}H-NMR spectrum of compound **PNAG18**.

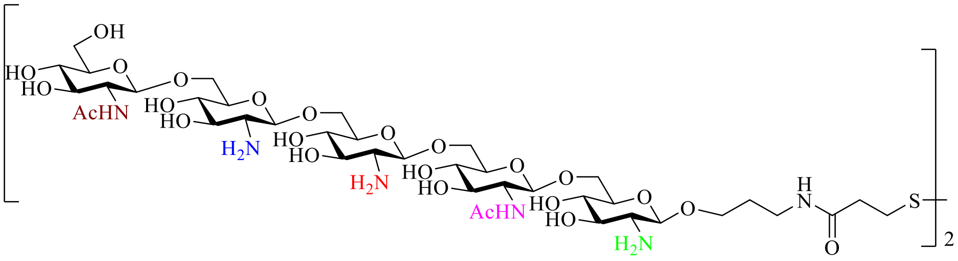

^{1}H-NMR (D_2O, 500 MHz) of **PNAG18**

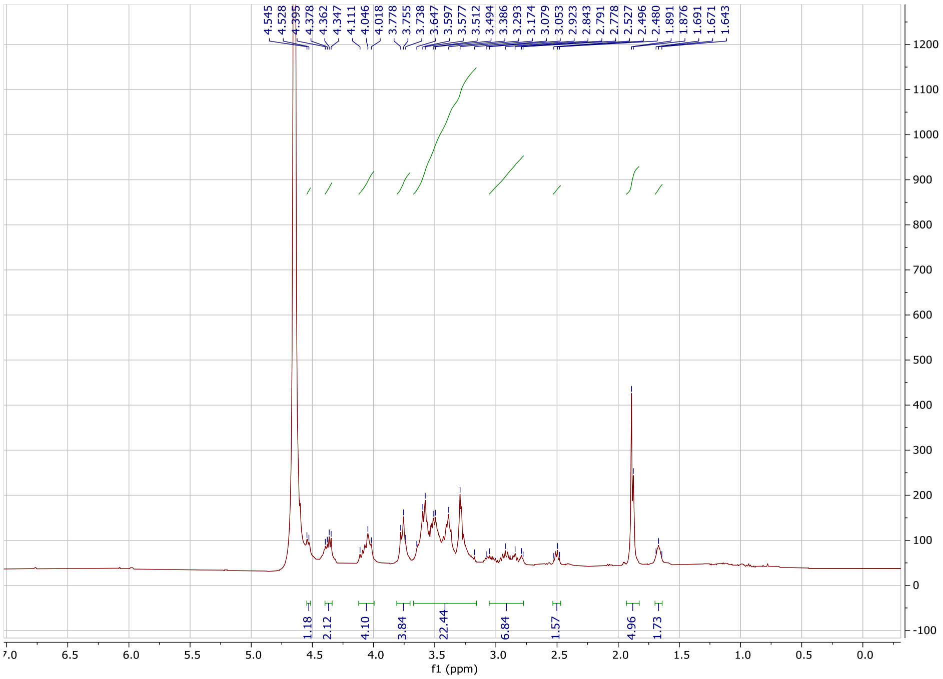

Supplementary Fig. 91. gHSQC spectrum of compound **PNAG18**.

gHSQC (D_2O, 500 MHz) of **PNAG18**

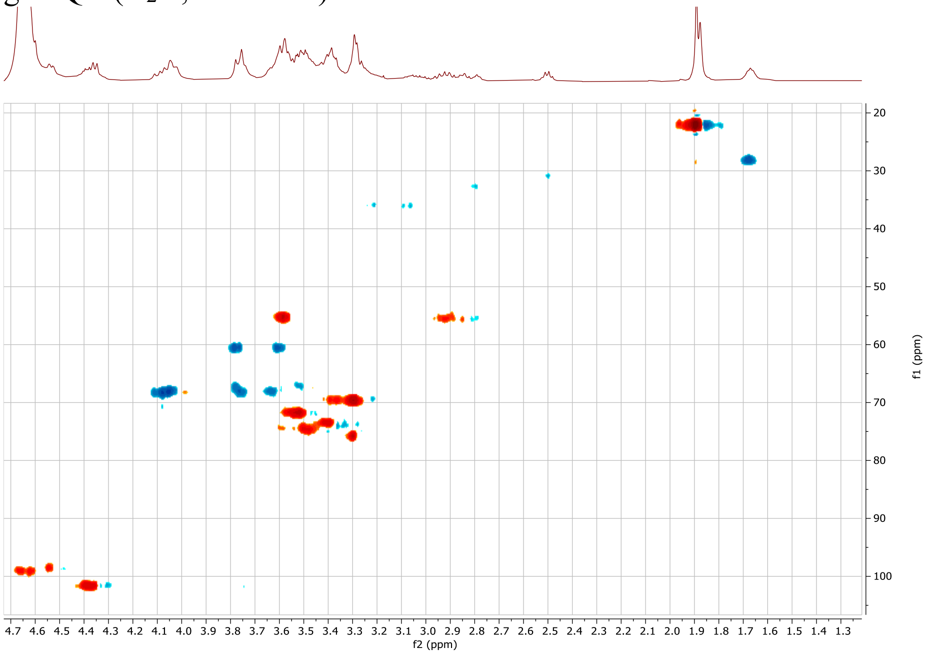

Supplementary Fig. 92. ^{1}H-NMR spectrum of compound **PNAG19**.

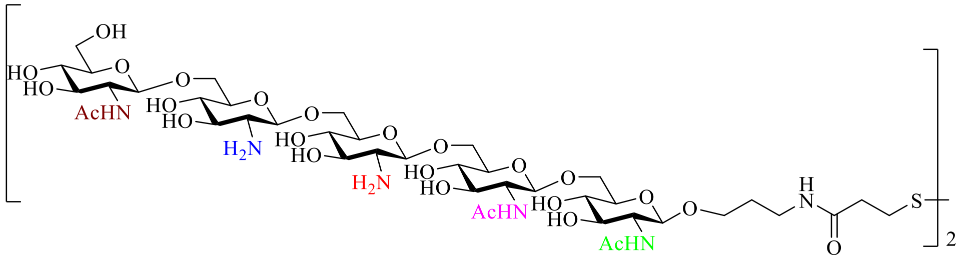

^{1}H-NMR (D_2O, 500 MHz) of **PNAG19**

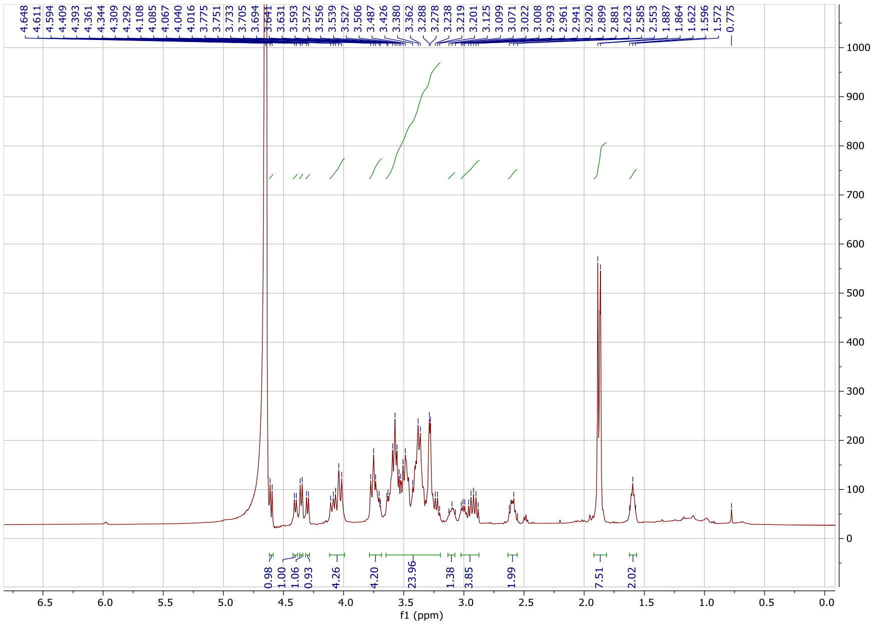

Supplementary Fig. 93. gHSQC spectrum of compound **PNAG19**.

gHSQC (D_2O, 500 MHz) of **PNAG19**

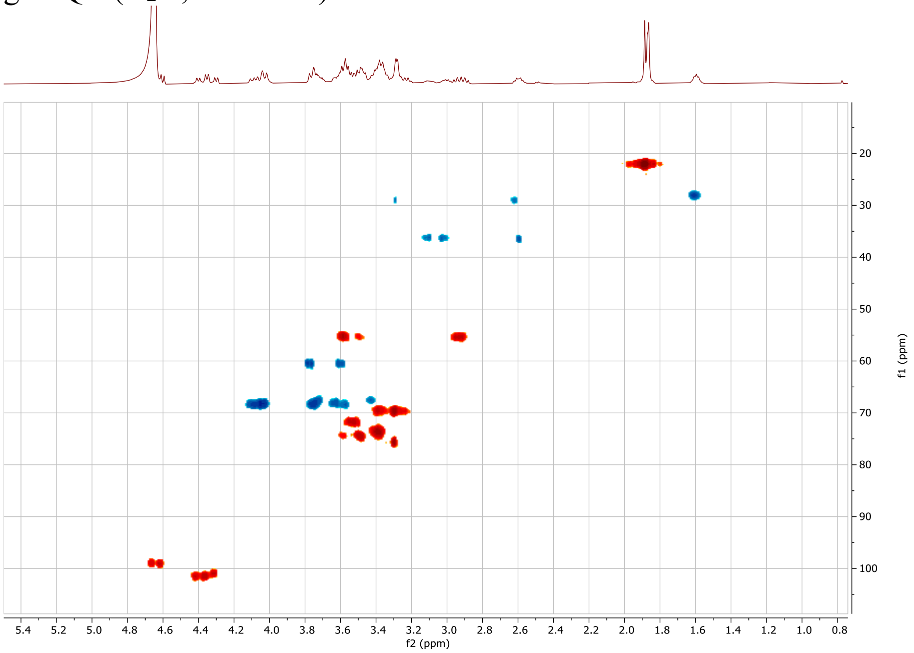

Supplementary Fig. 94. ^{1}H-NMR spectrum of compound **PNAG20**.

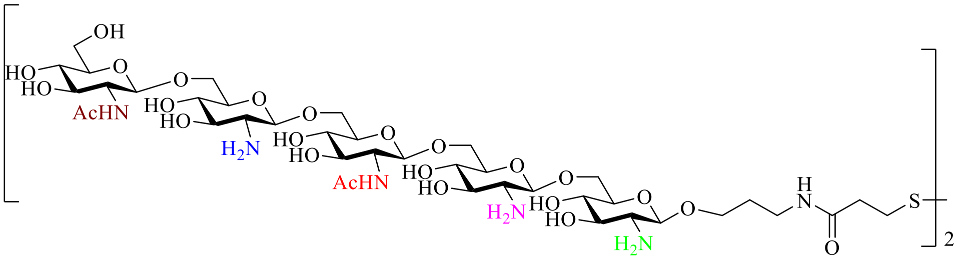

^{1}H-NMR (D_2O, 500 MHz) of **PNAG20**

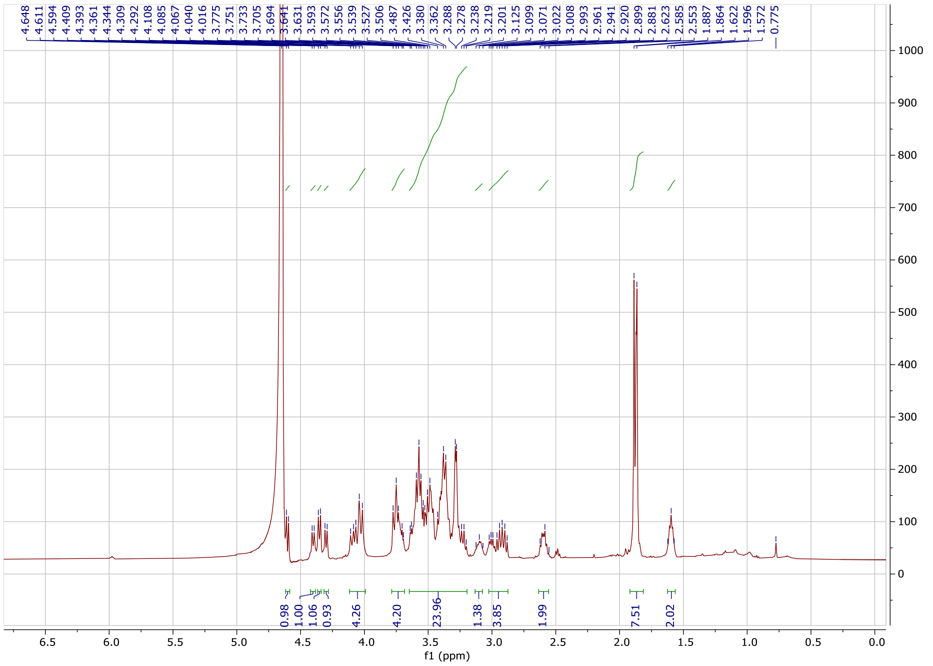

Supplementary Fig. 95. gHSQC spectrum of compound **PNAG20**.

gHSQC (D_2O, 500 MHz) of **PNAG20**

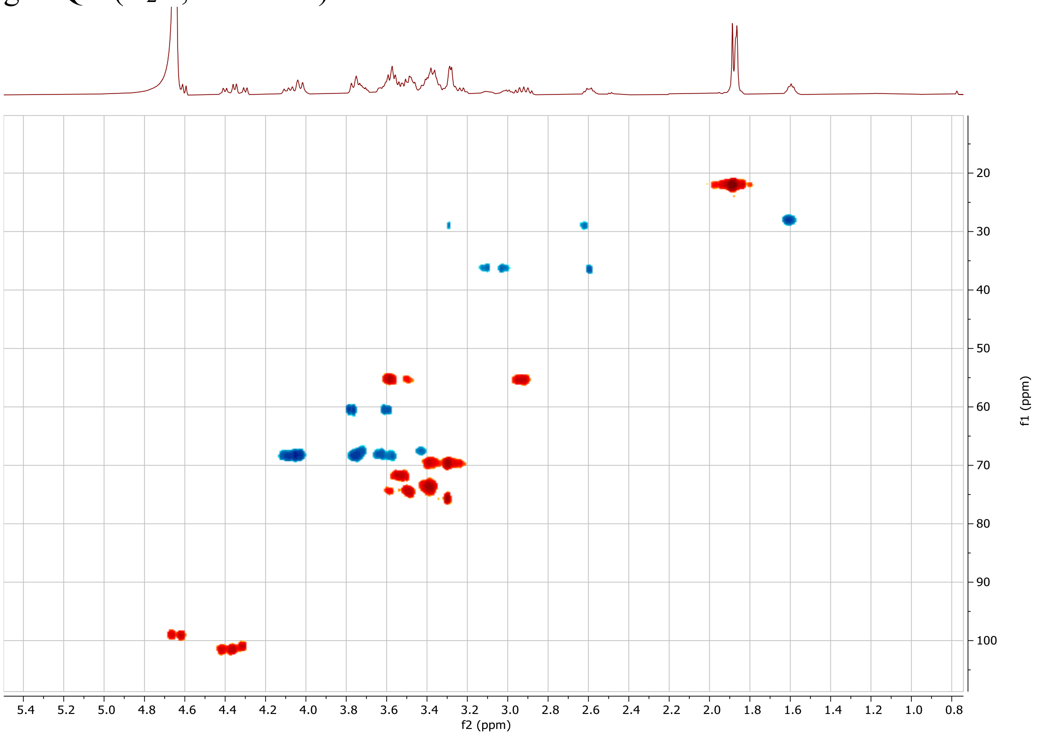

Supplementary Fig. 96. ^{1}H-NMR spectrum of compound **PNAG21**.

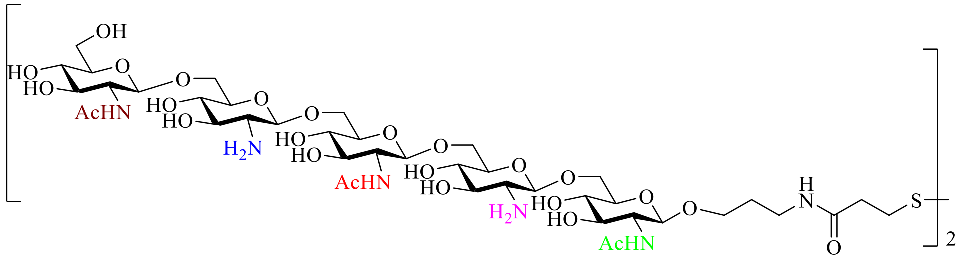

^{1}H-NMR (D_2O, 500 MHz) of **PNAG21**

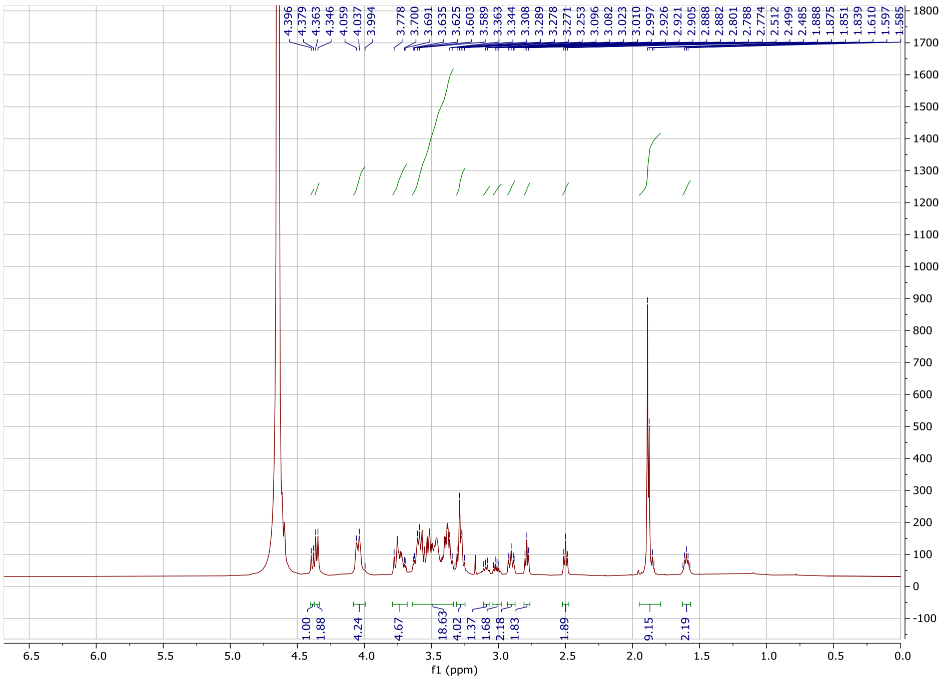

Supplementary Fig. 97. gHSQC spectrum of compound **PNAG21**.

gHSQC (D_2O, 500 MHz) of **PNAG21**

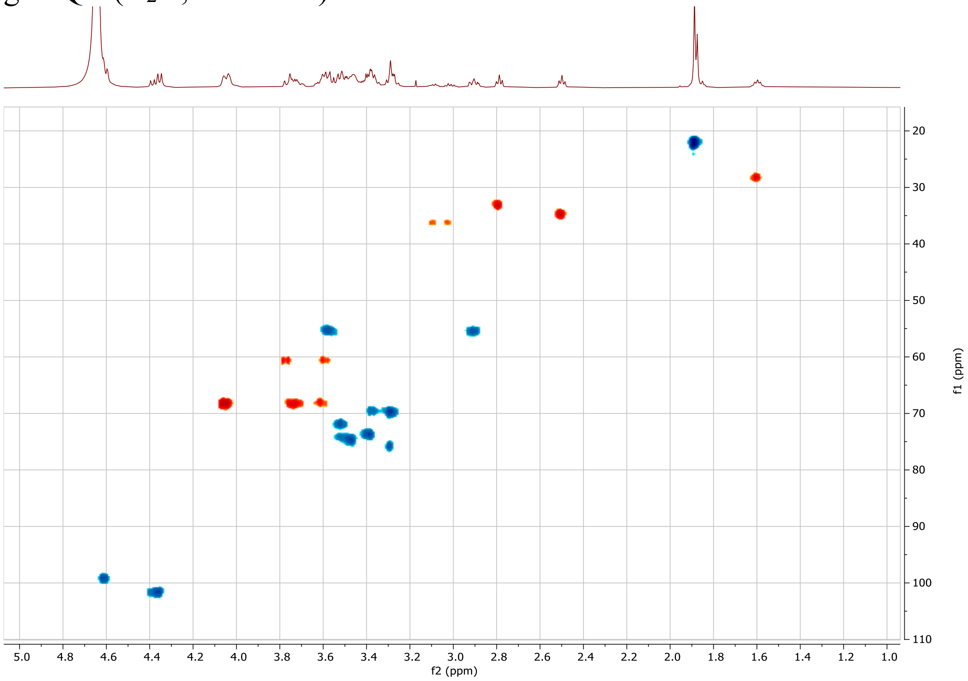

Supplementary Fig. 98. ^{1}H-NMR spectrum of compound **PNAG22**.

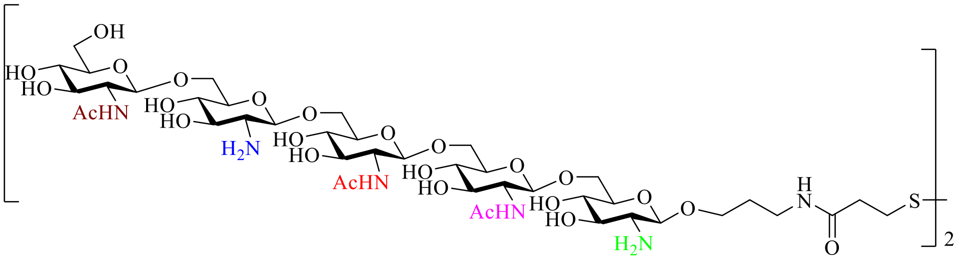

^{1}H-NMR (D_2O, 500 MHz) of **PNAG22**

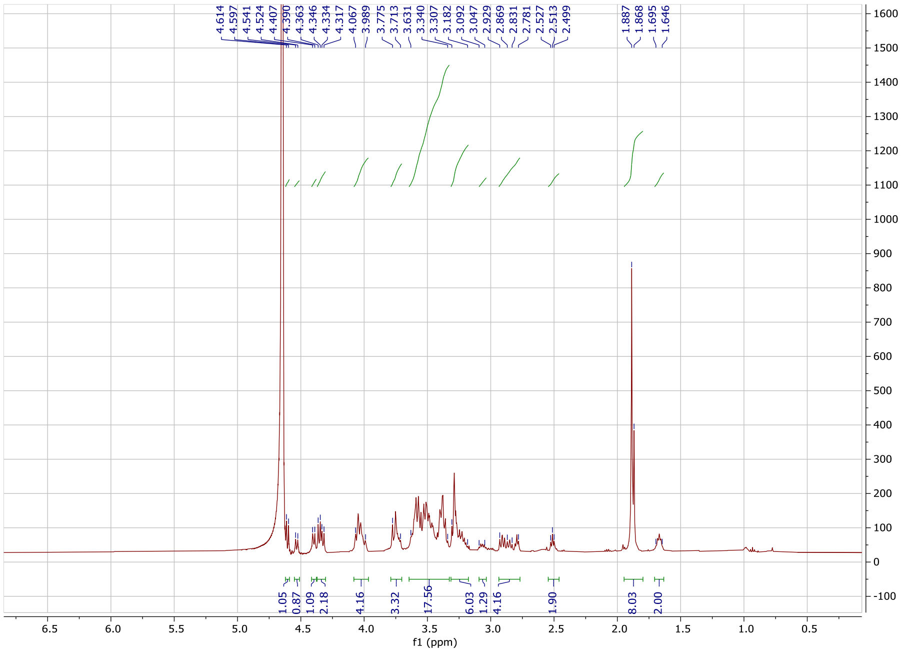

Supplementary Fig. 99. gHSQC spectrum of compound **PNAG22**.

gHSQC (D_2O, 500 MHz) of **PNAG22**

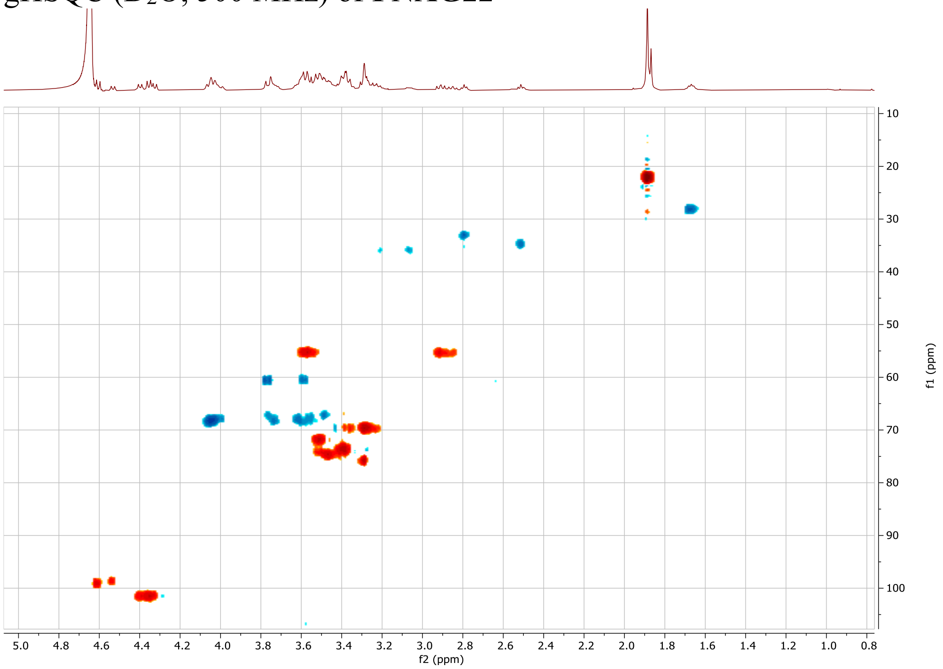

Supplementary Fig. 100. ^{1}H-NMR spectrum of compound **PNAG23**.

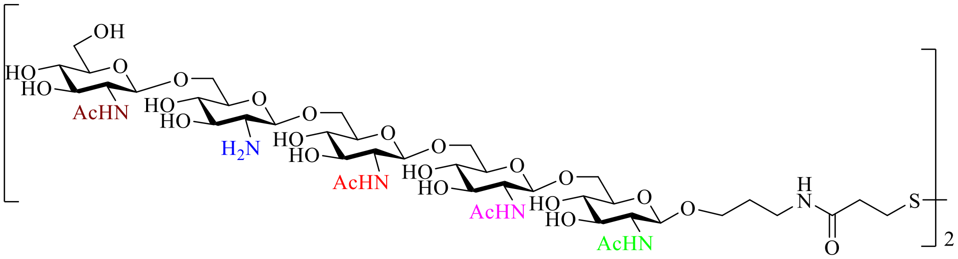

^{1}H-NMR (D_2O, 500 MHz) of **PNAG23**

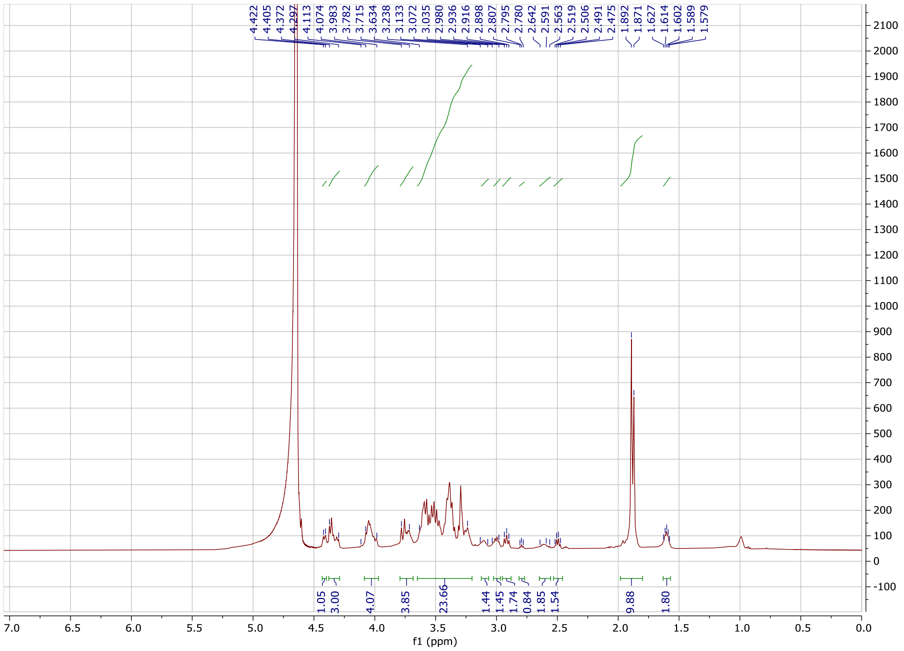

Supplementary Fig. 101. gHSQC spectrum of compound **PNAG23**.

gHSQC (D_2O, 500 MHz) of **PNAG23**

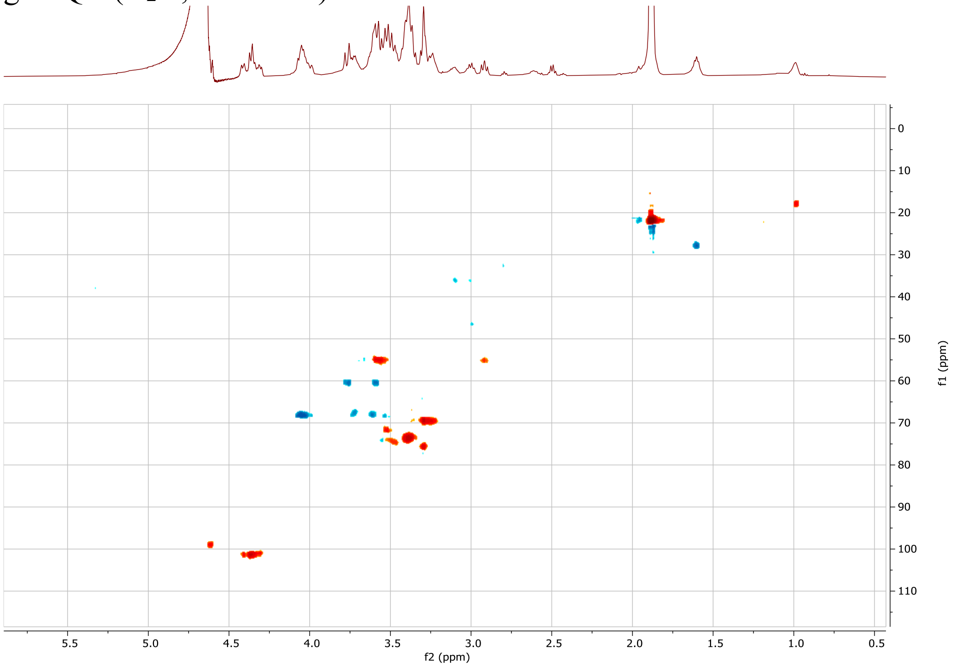

Supplementary Fig. 102. ^{1}H-NMR spectrum of compound **PNAG24**.

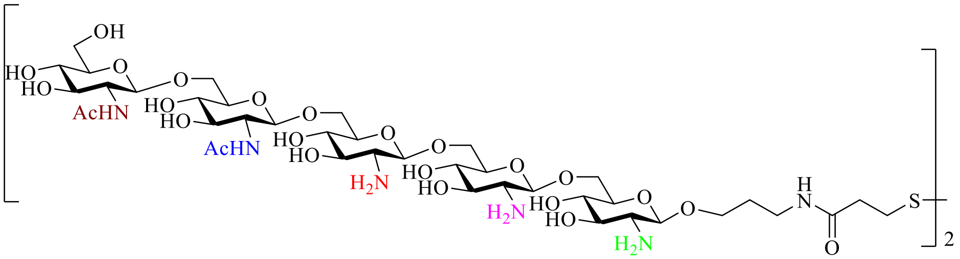

^{1}H-NMR (D_2O, 500 MHz) of **PNAG24**

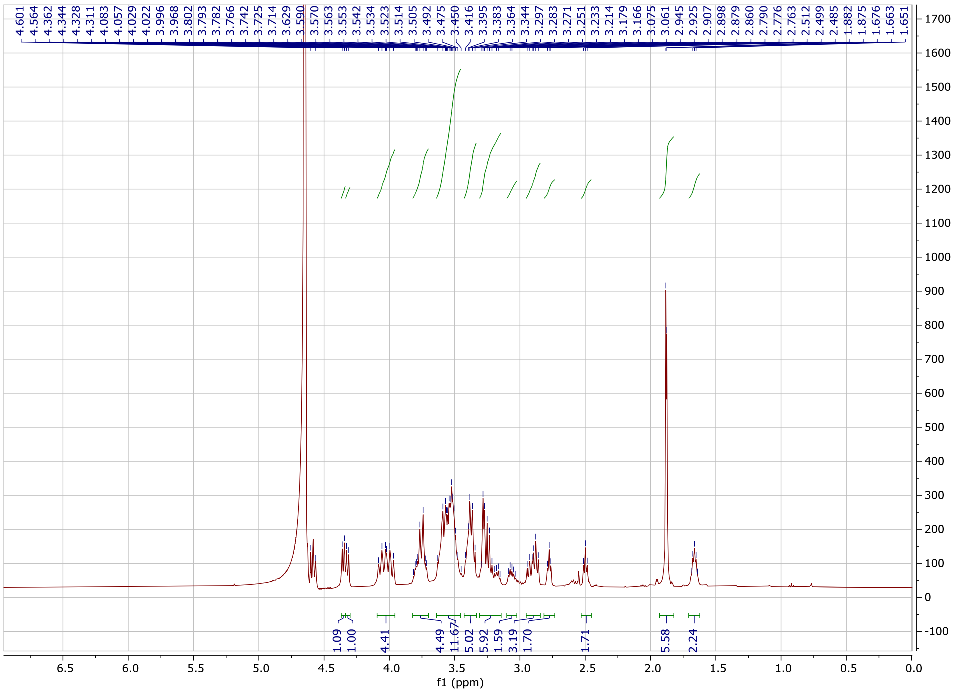

Supplementary Fig. 103. gHSQC spectrum of compound **PNAG24**.

gHSQC (D_2O, 500 MHz) of **PNAG24**

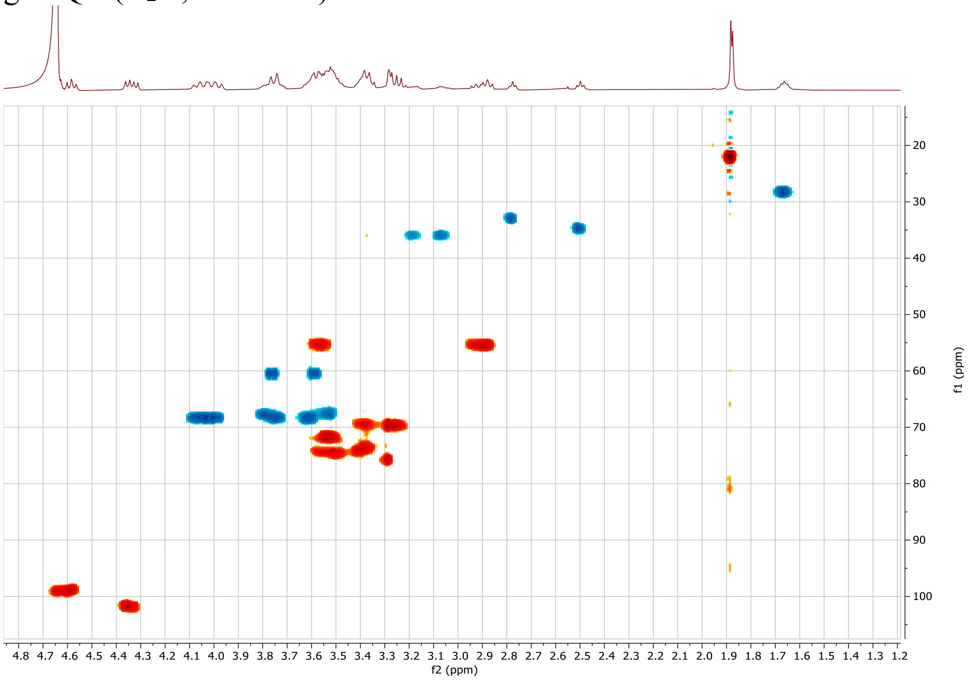

Supplementary Fig. 104. ^{1}H-NMR spectrum of compound **PNAG25**.

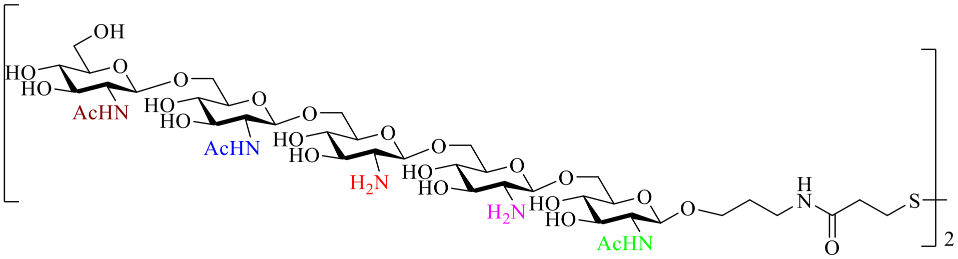

^{1}H-NMR (D_2O, 500 MHz) of **PNAG25**

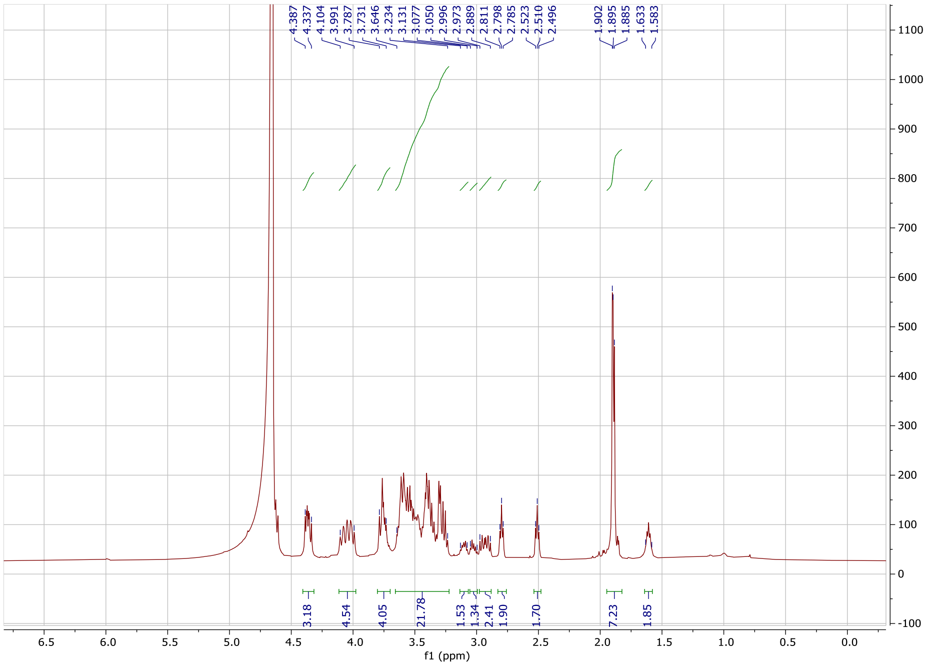

Supplementary Fig. 105. gHSQC spectrum of compound **PNAG25**.

gHSQC (D_2O, 500 MHz) of **PNAG25**

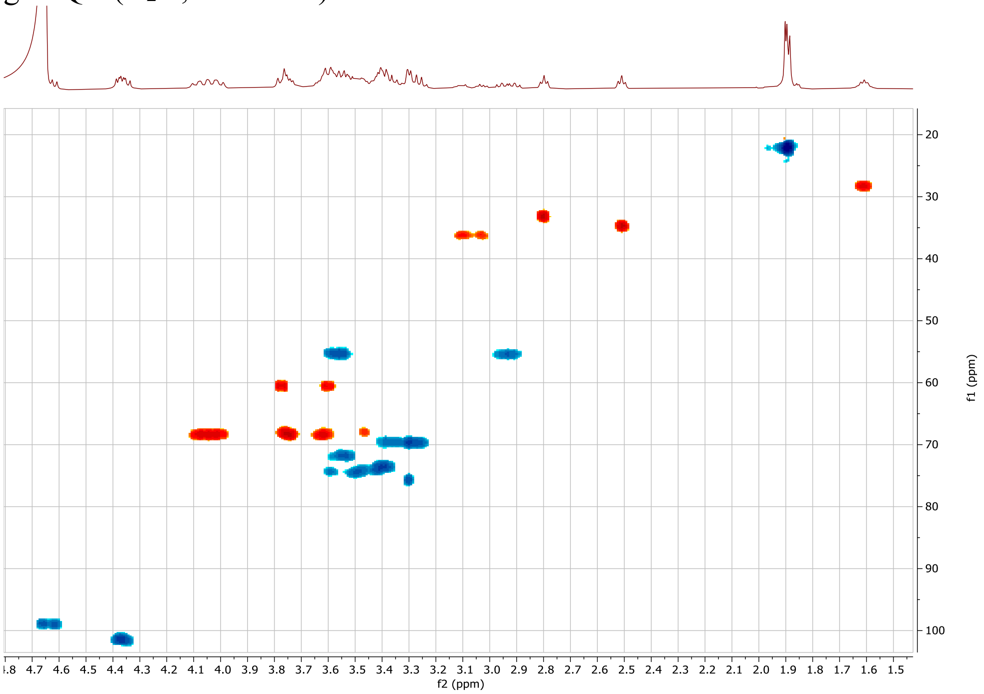

Supplementary Fig. 106. ^{1}H-NMR spectrum of compound **PNAG26**.

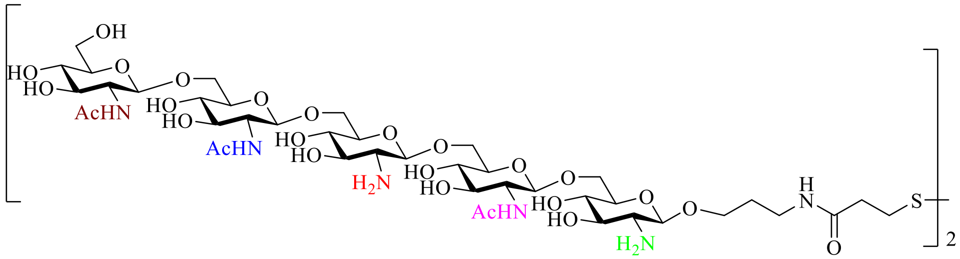

^{1}H-NMR (D_2O, 500 MHz) of **PNAG26**

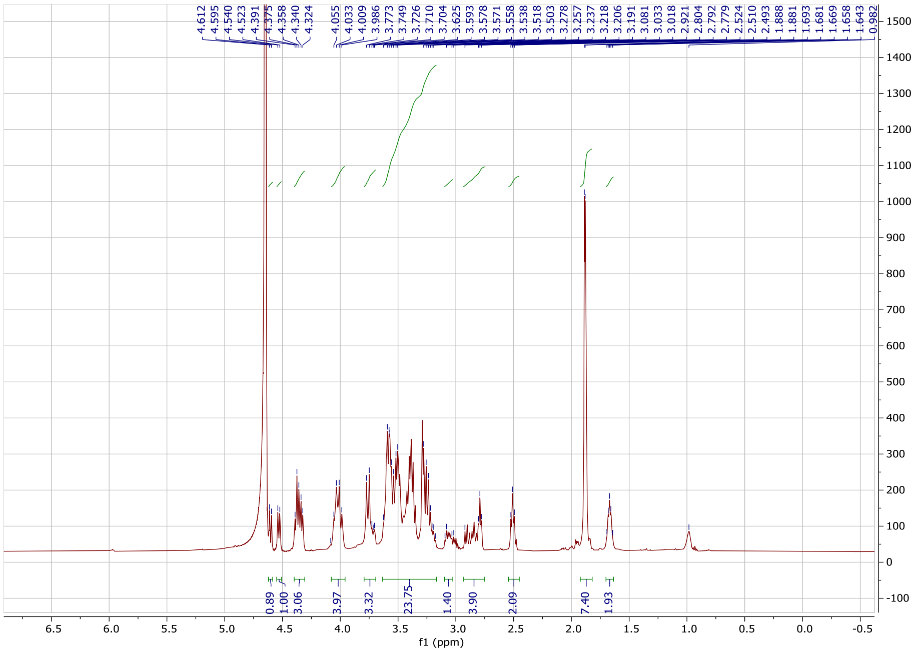

Supplementary Fig. 107. gHSQC spectrum of compound **PNAG26**.

gHSQC (D_2O, 500 MHz) of **PNAG26**

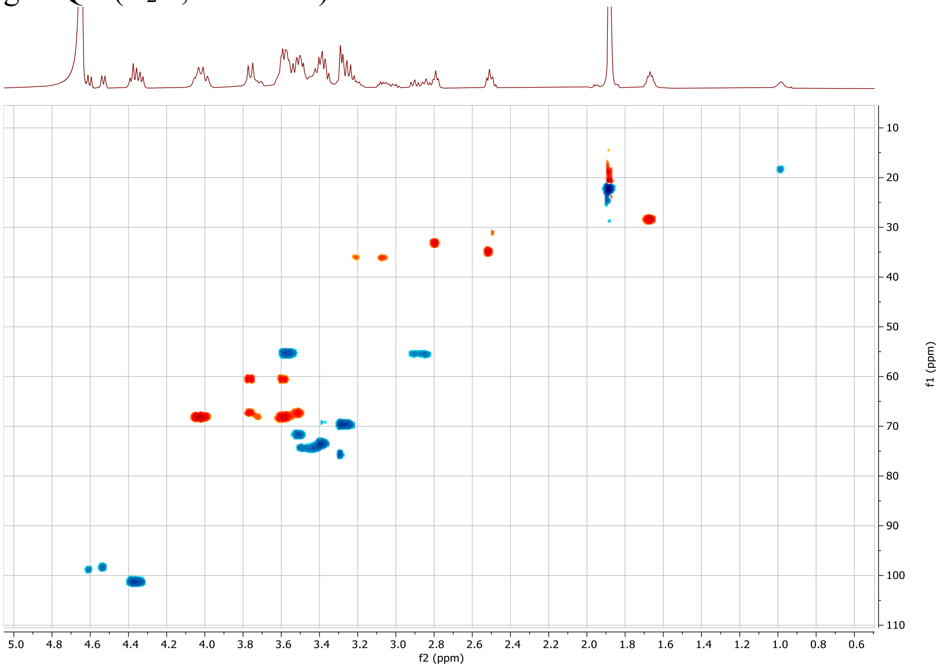

Supplementary Fig. 108. ^{1}H-NMR spectrum of compound **PNAG27**.

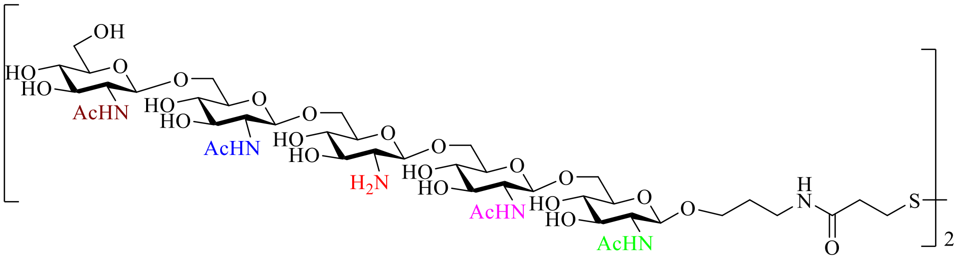

^{1}H-NMR (D_2O, 500 MHz) of **PNAG27**

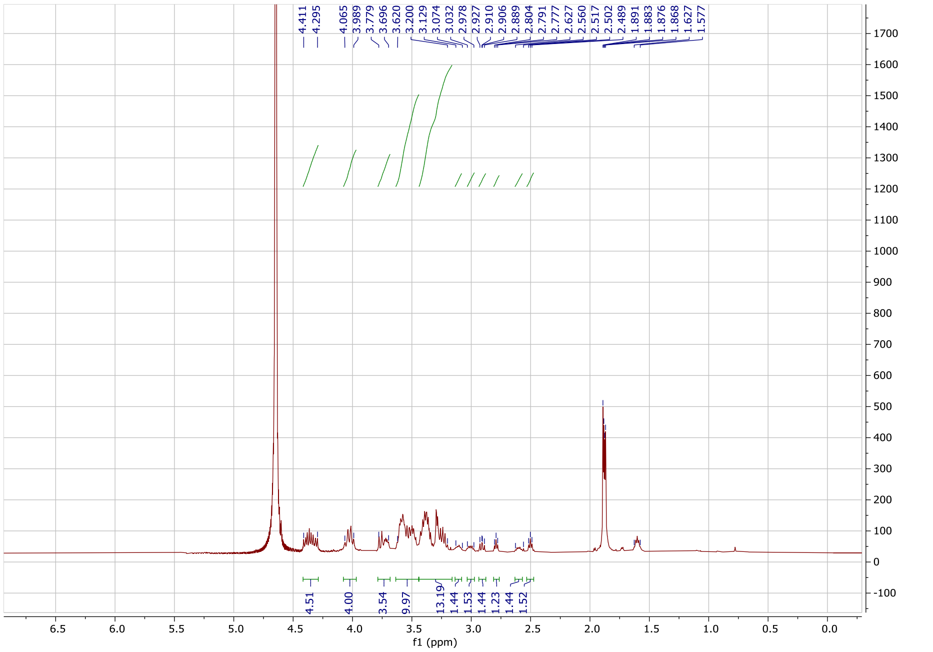

Supplementary Fig. 109. gHSQC spectrum of compound **PNAG27**.

gHSQC (D_2O, 500 MHz) of **PNAG27**

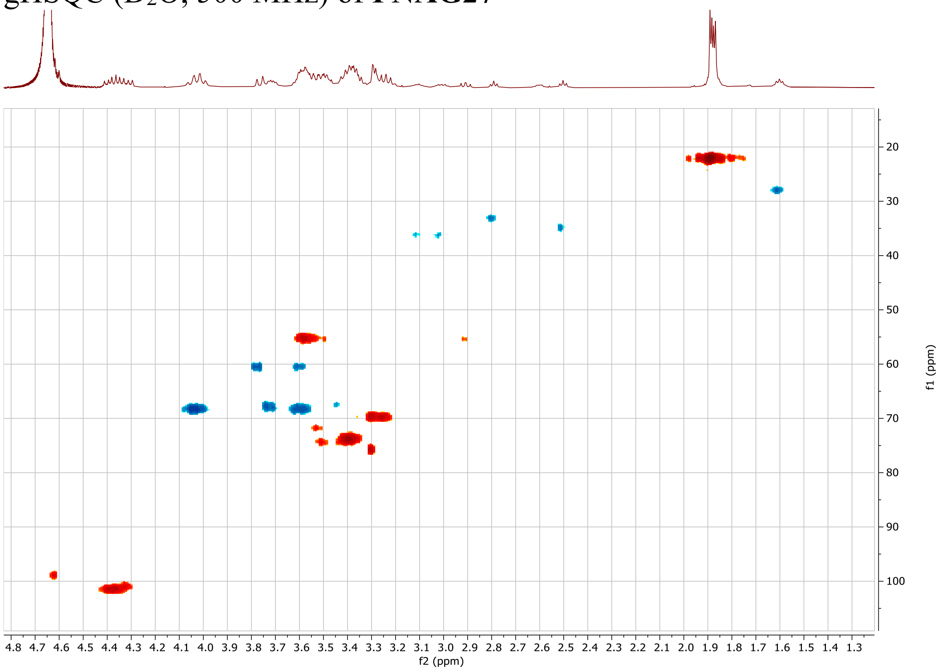

Supplementary Fig. 110. ^{1}H-NMR spectrum of compound **PNAG28**.

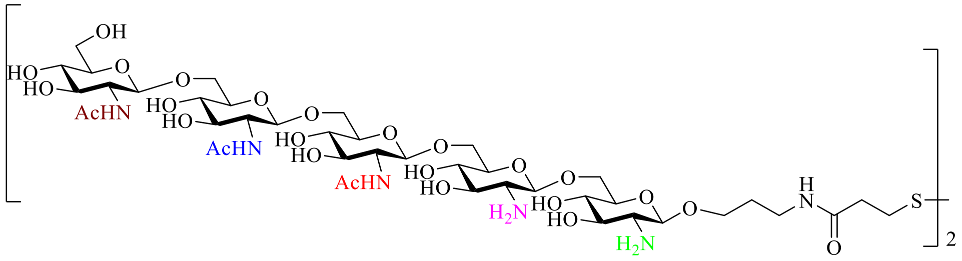

^{1}H-NMR (D_2O, 500 MHz) of **PNAG28**

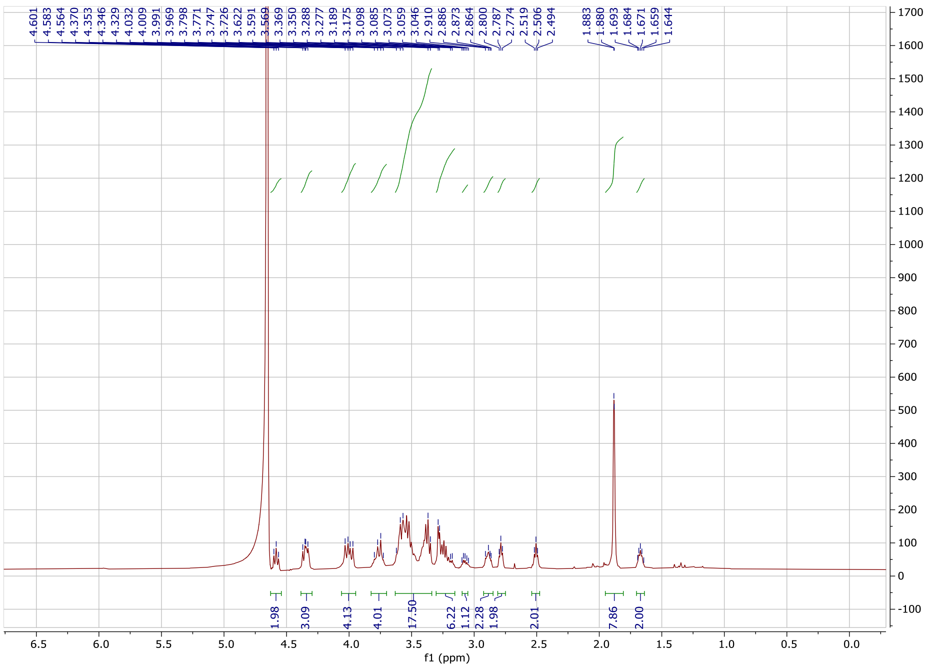

Supplementary Fig. 111. gHSQC spectrum of compound **PNAG28**.

gHSQC (D_2O, 500 MHz) of **PNAG28**

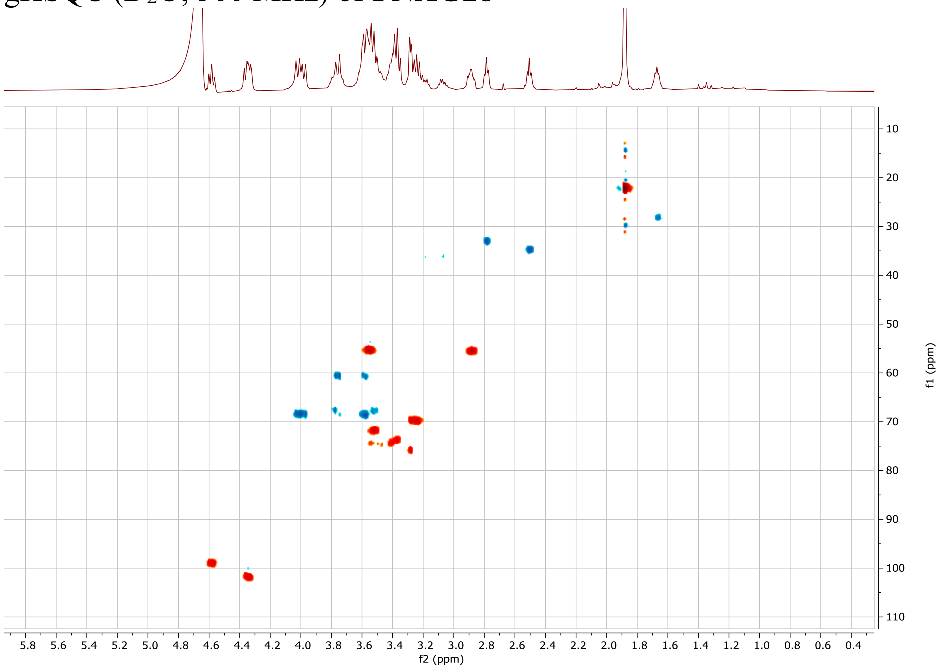

Supplementary Fig. 112. ^{1}H-NMR spectrum of compound **PNAG29**.

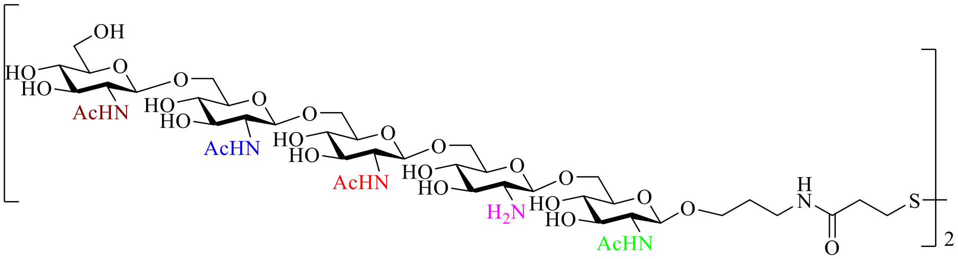

^{1}H-NMR (D_2O, 500 MHz) of **PNAG29**

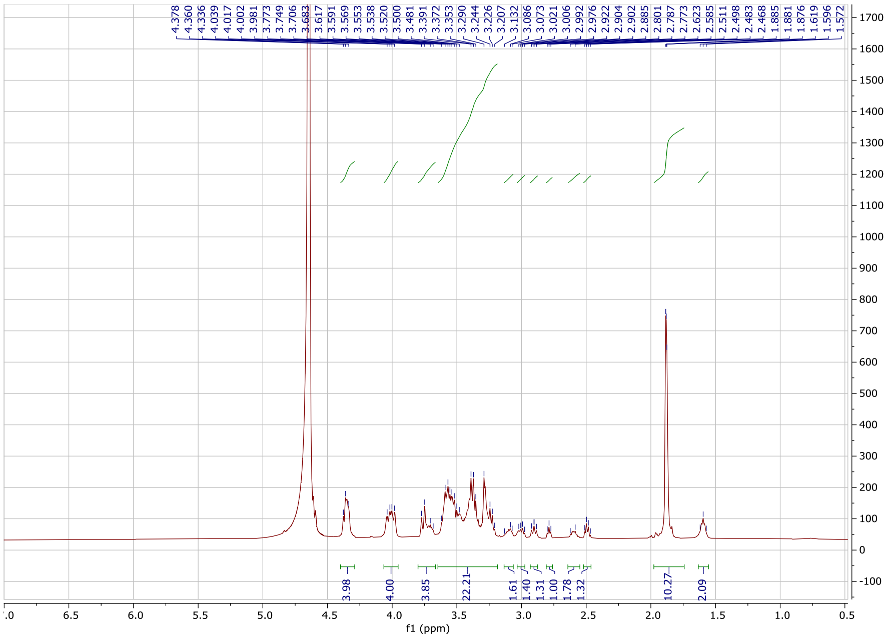

Supplementary Fig. 113. gHSQC spectrum of compound **PNAG29**.

gHSQC (D_2O, 500 MHz) of **PNAG29**

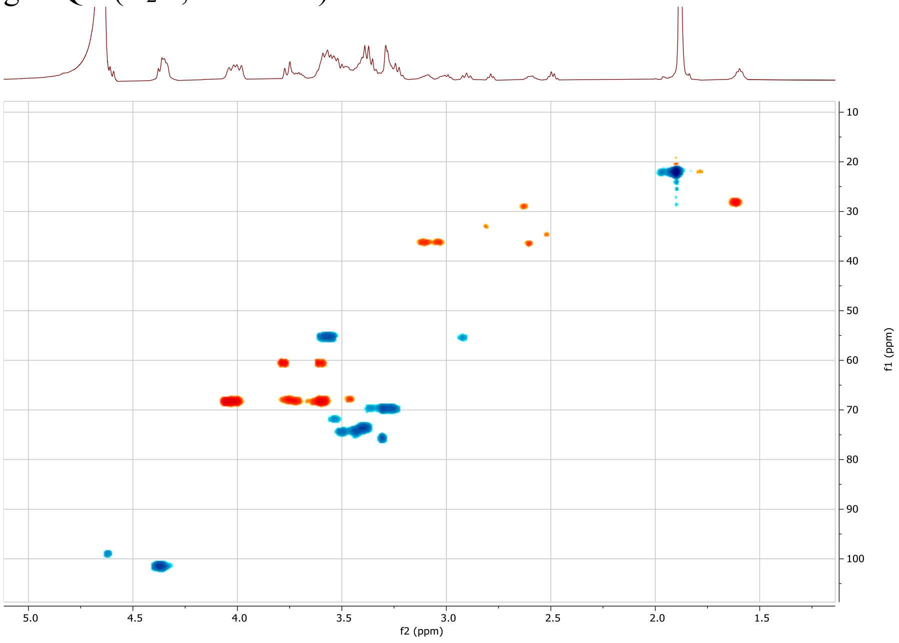

Supplementary Fig. 114. ^{1}H-NMR spectrum of compound **PNAG30**.

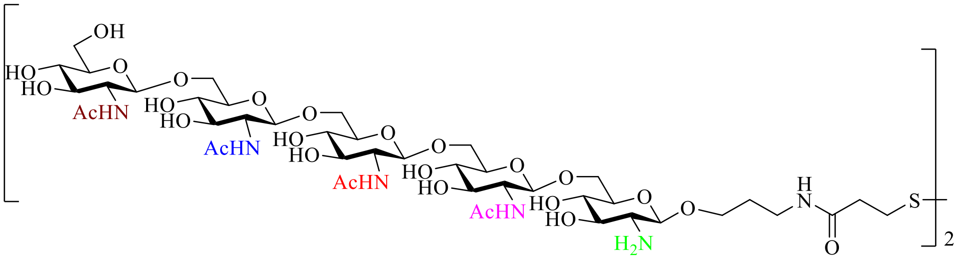

^{1}H-NMR (D_2O, 500 MHz) of **PNAG30**

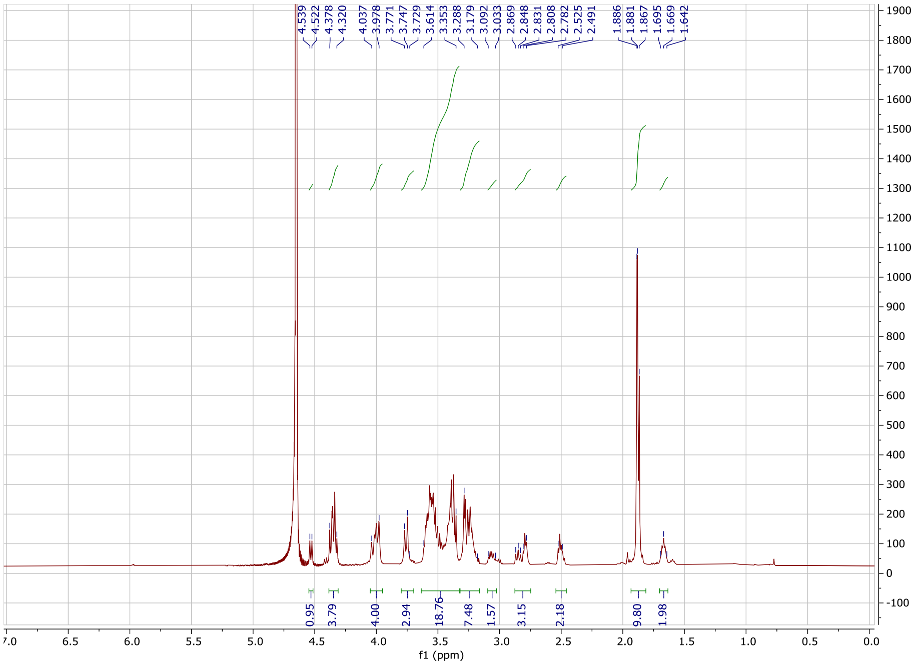

Supplementary Fig. 115. gHSQC spectrum of compound **PNAG30**.

gHSQC (D_2O, 500 MHz) of **PNAG30**

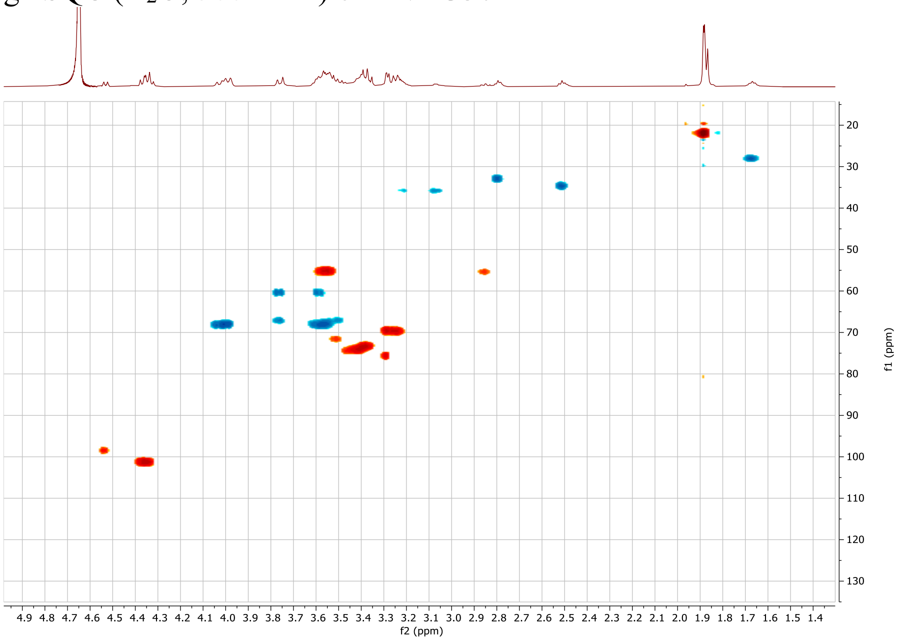

Supplementary Fig. 116. ^{1}H-NMR spectrum of compound **PNAG31**.

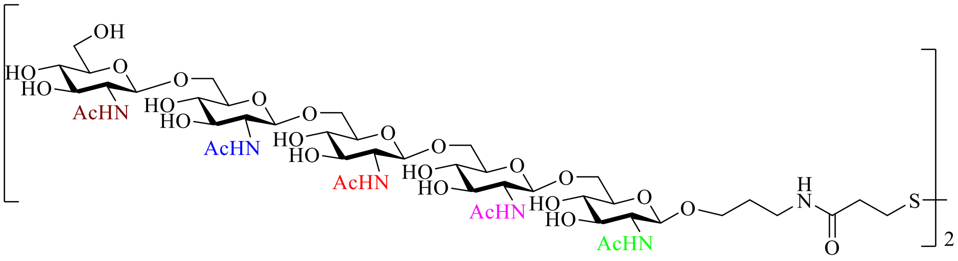

^{1}H-NMR (D_2O, 500 MHz) of **PNAG31**

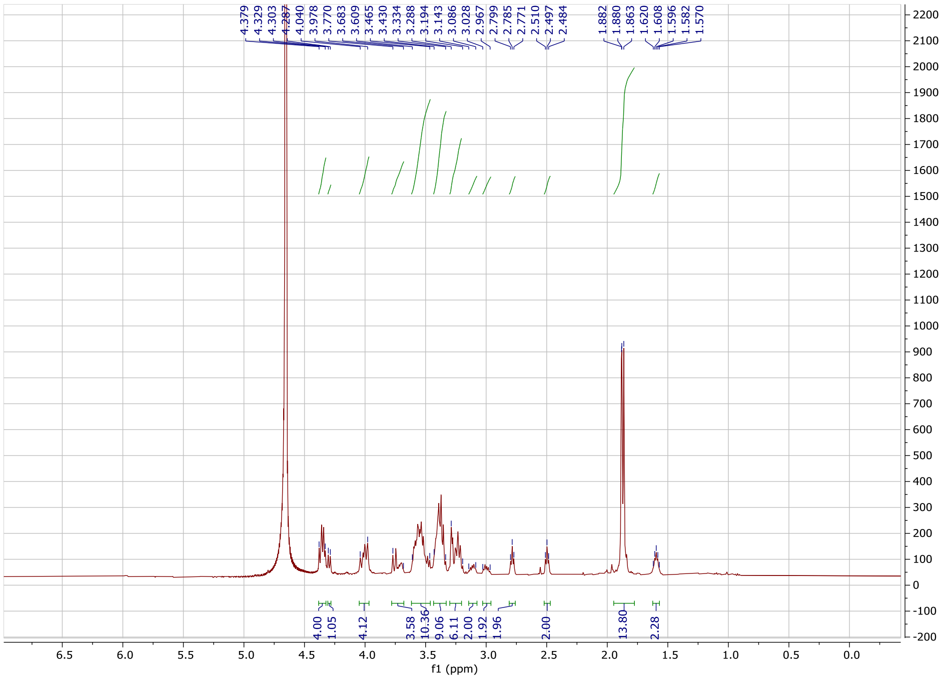

Supplementary Fig. 117. gHSQC spectrum of compound **PNAG31**.

gHSQC (D_2O, 500 MHz) of **PNAG31**

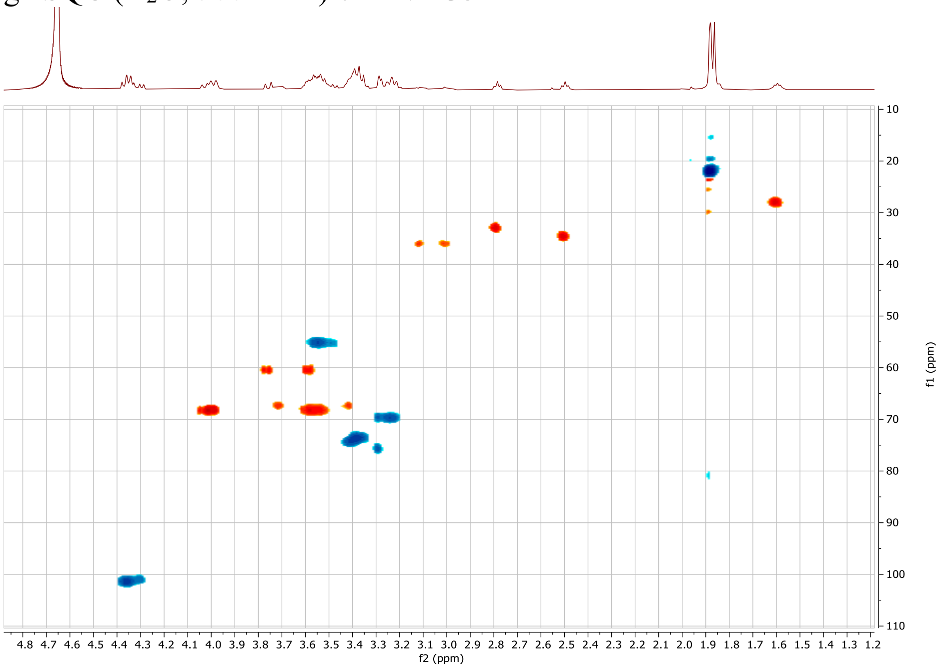

Supplementary References:

1. Hansen SG, Skrydstrup T. **Studies Directed to the Synthesis of Oligochitosans – Preparation of Building Blocks and Their Evaluation in Glycosylation Studies.** *Eur. J. Org. Chem.* **2007**; *2007*, 3392-3401.
2. Fey PD, Endres JL, Yajjala VK, Widhelm TJ, Boissy RJ, Bose JL, Bayles KW. **A genetic resource for rapid and comprehensive phenotype screening of nonessential Staphylococcus aureus genes**. *mBio* **2013**; *4*, e00537-00512.
3. Lensmire JM, Wischer MR, Kraemer-Zimpel C, Kies PJ, Sosinski L, Ensink E, Dodson JP, Shook JC, Delekta PC, Cooper CC, Havlichek DH, Jr., Mulks MH, Lunt SY, Ravi J, Hammer ND. **The glutathione import system satisfies the Staphylococcus aureus nutrient sulfur requirement and promotes interspecies competition**. *PLoS Genet.* **2023**; *19*, e1010834.
