# Supplementary material for: A comprehensive synthetic library of poly-N-acetyl glucosamines enabled vaccine against lethal challenges of Staphylococcus aureus
Source: Nat Commun. 2024 Apr 24;15:3420. doi: 10.1038/s41467-024-47457-4 (PMC11043332; doi:10.1038/s41467-024-47457-4)
Supplement: Supplementary file 3 — Reporting Summary [file 41467_2024_47457_MOESM3_ESM.pdf]

nature portfolio

Corresponding author(s): Xuefei Huang

Last updated by author(s): March 29th, 2024

Reporting Summary

Nature Portfolio wishes to improve the reproducibility of the work that we publish. This form provides structure for consistency and transparency in reporting. For further information on Nature Portfolio policies, see our Editorial Policies and the Editorial Policy Checklist.

Statistics

For all statistical analyses, confirm that the following items are present in the figure legend, table legend, main text, or Methods section.

n/a	Confirmed	
☐	☒	The exact sample size (*n*) for each experimental group/condition, given as a discrete number and unit of measurement
☐	☒	A statement on whether measurements were taken from distinct samples or whether the same sample was measured repeatedly
☐	☒	The statistical test(s) used AND whether they are one- or two-sided<br>*Only common tests should be described solely by name; describe more complex techniques in the Methods section.*
☐	☒	A description of all covariates tested
☒	☐	A description of any assumptions or corrections, such as tests of normality and adjustment for multiple comparisons
☐	☒	A full description of the statistical parameters including central tendency (e.g. means) or other basic estimates (e.g. regression coefficient) AND variation (e.g. standard deviation) or associated estimates of uncertainty (e.g. confidence intervals)
☐	☒	For null hypothesis testing, the test statistic (e.g. *F*, *t*, *r*) with confidence intervals, effect sizes, degrees of freedom and *P* value noted<br>*Give P values as exact values whenever suitable.*
☒	☐	For Bayesian analysis, information on the choice of priors and Markov chain Monte Carlo settings
☒	☐	For hierarchical and complex designs, identification of the appropriate level for tests and full reporting of outcomes
☒	☐	Estimates of effect sizes (e.g. Cohen's *d*, Pearson's *r*), indicating how they were calculated

Our web collection on statistics for biologists contains articles on many of the points above.

Software and code

Policy information about availability of computer code

Data collection	No software was used.
Data analysis	Data analysis was performed using Graph Pad Prism 6.

For manuscripts utilizing custom algorithms or software that are central to the research but not yet described in published literature, software must be made available to editors and reviewers. We strongly encourage code deposition in a community repository (e.g. GitHub). See the Nature Portfolio guidelines for submitting code & software for further information.

Data

Policy information about availability of data

All manuscripts must include a data availability statement. This statement should provide the following information, where applicable:

- Accession codes, unique identifiers, or web links for publicly available datasets
- A description of any restrictions on data availability
- For clinical datasets or third party data, please ensure that the statement adheres to our policy

The sequencing data has been submitted to Gen bank/SRA (Bioproject ID PRJNA997319). All other data are in the supporting information.

Research involving human participants, their data, or biological material

Policy information about studies with human participants or human data. See also policy information about sex, gender (identity/presentation), and sexual orientation and race, ethnicity and racism.

Reporting on sex and gender	No human participants or human data involved in this study.
Reporting on race, ethnicity, or other socially relevant groupings	N/A
Population characteristics	N/A
Recruitment	N/A
Ethics oversight	N/A

Note that full information on the approval of the study protocol must also be provided in the manuscript.

Field-specific reporting

Please select the one below that is the best fit for your research. If you are not sure, read the appropriate sections before making your selection.

☒ Life sciences ☐ Behavioural & social sciences ☐ Ecological, evolutionary & environmental sciences

For a reference copy of the document with all sections, see nature.com/documents/nr-reporting-summary-flat.pdf

Life sciences study design

All studies must disclose on these points even when the disclosure is negative.

Sample size	Sample sizes for mouse studies were based on likelihood of achieving a >65% difference with an alpha <0.05 and beta >80%. For rabbit sera, no sample size calculation was needed. It is just a demonstration that the titers achieved post-immunization were significantly higher than those of the rabbits before immunization.
Data exclusions	none
Replication	We repeated mQb-PNAG0 and TTHc-PNAG0 immunization and protection. The results were similar. All attempts at replication were successful.
Randomization	Mice were received from vendors and placed in groups in cages. All comparisons were with mice from the same group
Blinding	Investigators were blinded to the treatments used

Reporting for specific materials, systems and methods

We require information from authors about some types of materials, experimental systems and methods used in many studies. Here, indicate whether each material, system or method listed is relevant to your study. If you are not sure if a list item applies to your research, read the appropriate section before selecting a response.

Materials & experimental systems

n/a	Involved in the study	
☐	☒	Antibodies
☐	☒	Eukaryotic cell lines
☒	☐	Palaeontology and archaeology
☐	☒	Animals and other organisms
☒	☐	Clinical data
☒	☐	Dual use research of concern
☒	☐	Plants

Methods

n/a	Involved in the study	
☒	☐	ChIP-seq
☒	☐	Flow cytometry
☒	☐	MRI-based neuroimaging

Antibodies

Antibodies used	Cy3 anti-rabbit IgG (Cy'"3 AffiniPure Goat Anti-Rabbit IgG, Fc fragment specific; Jackson 111-165-046); AlexaFluor 647 Anti-rabbit IgM (Alexa Fluor® 647 labeled Goat Anti-Rabbit IgM mu chain; Abcam 150095); F598 (produced by Dr. Pier); Peroxidase AffiniPure Goat

	Anti-Mouse IgG (H+L) (Jackson 115-035-062); Peroxidase AffiniPure Goat Anti-Rabbit IgG (H+L) (Jackson 111-035-045); peroxidase-goat anti mouse IgG antibody (Jackson 115-035-003), affinity-purified goat anti-human complement C1q antibody (Cedarlane, CL7341AP); alkaline phosphatase conjugated goat anti mouse IgG antibody (Sigma-Aldrich AP308A); alkaline phosphatase conjugated goat anti rabbit IgG antibody (Sigma-Aldrich AP307A).
Validation	https://www.jacksonimmuno.com/catalog/products/111-165-046<br>https://www.abcam.com/products/secondary-antibodies/goat-rabbit-igm-mu-chain-alexa-fluor-647-ab150095.html<br>https://www.jacksonimmuno.com/catalog/products/115-035-062<br>https://www.jacksonimmuno.com/catalog/products/111-035-045<br>https://www.cedarlanelabs.com/Products/Detail/CL7341AP?lob=AllProducts<br>https://www.sigmaaldrich.com/US/en/product/mm/ap308a<br>https://www.sigmaaldrich.com/US/en/product/mm/ap307a

Eukaryotic cell lines

Policy information about cell lines and Sex and Gender in Research

Cell line source(s)	HL-60 cell, which is a promyelocytic cell line derived from peripheral blood leukocytes obtained by leukopheresis from a 36-year-old Caucasian female with acute promyelocytic leukemia.<br>https://www.atcc.org/products/ccl-240?matchtype=e&network=g&device=c&adposition=&keyword=hl60%20cell%20line&gad_source=1&gclid=CjwKCAiAx_GqBhBCIEiwAIDNAZhzUFvmZexwUzUf7wrLon5eKWK1SNMabEEgdRfpdgJDQ8V8sPhOxoCoQEQAvD_BwE
Authentication	We did not further authenticate this cell line after receiving it from ATCC
Mycoplasma contamination	This cell was tested negative for mycoplasma contamination
Commonly misidentified lines<br>(See ICLAC register)	No commonly misidentified cell lines were used

Animals and other research organisms

Policy information about studies involving animals; ARRIVE guidelines recommended for reporting animal research, and Sex and Gender in Research

Laboratory animals	Pathogen-free C57BL/6 or CD1 female mice aged 6 weeks were obtained from Charles River. Female New Zealand white rabbits aged 15 weeks were provided by ProSci.
Wild animals	This study did not involve wild animals.
Reporting on sex	We only used female mice for our study as it was easier to house female mice together. While we did not utilize male mice for the current study, previously, we have compared the antibody responses to various Qbeta-glycan conjugates of female vs male mice. Both sexes responded well with no significant differences. Female rabbits were used to be consistent with the mouse study.
Field-collected samples	This study did not involve field-collected samples.
Ethics oversight	All animal care procedures and experimental protocols have been approved by the Institutional Animal Care and Use Committee (IACUC) of Michigan State University (protocol number: 202200444).

Note that full information on the approval of the study protocol must also be provided in the manuscript.

Plants

Seed stocks	This study did not involve plants.
Novel plant genotypes	N/A
Authentication	N/A
